# Diastereo‐ and Enantioselective Access to Stereotriads through a Flexible Coupling of Substituted Aldehydes and Alkenes

**DOI:** 10.1002/anie.201900801

**Published:** 2019-03-27

**Authors:** Jing Li, Alexander Preinfalk, Nuno Maulide

**Affiliations:** ^1^ University of Vienna Institute of Organic Chemistry Währinger Strasse 38 1090 Vienna Austria

**Keywords:** aldehydes, alkenes, coupling reactions, Neopeltolide, redox-neutral reactions

## Abstract

A flexible redox‐neutral coupling of aldehydes and alkenes enables rapid access to stereotriads starting from a single stereocenter with perfect levels of enantio‐ and diastereoselectivity under mild conditions. The versatility of the method is highlighted by the installation of heteroatoms along the tether, which enables a route to structurally diverse building blocks. The formal synthesis of (+)‐neopeltolide further demonstrates the synthetic utility of this approach.

Linear reductive coupling products of aldehydes with alkenes are common structural motifs in a diverse range of biologically active natural products (Scheme 1 a).^1^, ^2^, ^3^ Traditional synthetic routes towards these scaffolds involve elaborate reagents and multiple‐step approaches, thus leading to long synthetic routes to the target compounds.^4^ In a recent example, the Krauss group elegantly achieved the homocrotylation of aldehydes using enantiopure cyclopropylboron reagents.^5^ However, preparation of the key cyclopropylmethylboronate reagent **A** requires auxiliary‐based methodology and a 5–6 step synthesis (Scheme 1 b). Thus, a more direct synthesis of such products is still in high demand. We recently disclosed a redox‐neutral coupling of aldehydes and alkenes, which selectively yields linear products **B** with perfect levels of enantio‐ and diastereoselectivity through a tethering “catch‐release” approach. This method enables for the first time the enantioselective synthesis of these compounds in a single‐step operation (Scheme 1 c).6a,6b

**Scheme 1:**
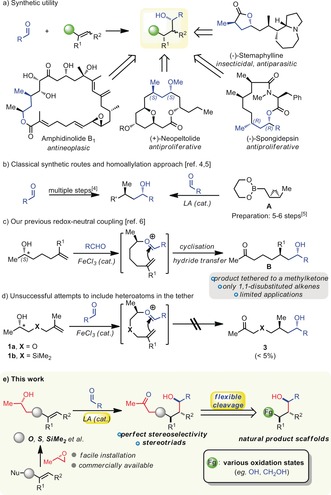
a) Synthetic utility of the coupling products of linear aldehyde and alkenes. b) Classical approaches. c) Our prior work. d) Limitations of our method. e) A flexible access to stereotriads.

A limitation of this method, however, is the necessary presence in the coupling products **B** of a long carbon chain capped by a (methyl)ketone. Cognizant of this, we became interested in studying systems carrying a heteroatom in the bridging chain with a view to employing those heteroatoms as reactive handles for chain cleavage. To our dismay, reaction of silicon‐tethered **1 a** or oxygen‐tethered **1 b** under the conditions previously developed never gave the desired products in synthetically useful yields (Scheme 1 d).6c

Furthermore, we realized that an additional substituent on the alkene present in the starting material would generate three contiguous stereogenic centres staring from the lone chiral‐epoxide‐derived stereocenter of the precursor. However, it was not clear whether any selectivity at all would be observed in this event. Herein, the development of a method for the synthesis of heteroatom‐bridged products that enables selective cleavage at every position of the bridging chain. This allows a highly flexible building‐block synthesis of complicated natural product scaffolds (Scheme 1 e). Additionally, we describe the flexible and fully stereoselective synthesis of stereotriads through redox‐neutral coupling of trisubstituted alkenes, culminating in a formal synthesis of the marine macrolide (+)‐neopeltolide.

Experiments with silicon‐tethered alcohol **1 a** and hydrocinnamaldehyde (**2 a**) quickly revealed that our previously optimised conditions employing FeCl_3_ led only to decomposition (Table 1, entry 1). Using oxygen‐tethered alcohols **1 b** and **1 c** under the same conditions resulted in similar observations (entries 2 and 3). Further optimisation on **1 a** is shown in Table 1. Increasing the catalyst loading from 5 to 50 % finally gave coupling product **3 a** but only in 10 % yield (entry 4). However, further increasing the amounts of FeCl_3_ and the temperature did not lead to any improvement (entries 5–6). We then investigated other Lewis Acids (e.g. TMSOTf and TBSOTf; entries 7–8), as well as Brønsted acids (TFA, TfOH, and Tf_2_NH; entries 9–11). Unfortunately, none of these conditions resulted in good yields of the desired coupling product. Finally, we discovered that cheap BF_3_ **⋅**Et_2_O effectively promotes the reaction (entries 12–13), with the best results obtained when one equivalent of Lewis acid was employed (entry 13). We surmise that the coordinating heteroatom in the tether is responsible for strong binding to the Lewis Acid, thus mandating the use of a full equivalent.

**Table 1:** Optimization of conditions for heteroatom‐tethered unsaturated alcohols.^[a]^ 
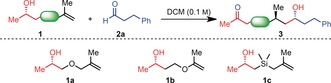

Entry	**1**	Lewis acid (%)	Yield (%)	dr	*ee* (%)
1	**1 a**	FeCl_3_ (5 %)	<5	nd	nd
2	**1 b**	FeCl_3_ (5 %)	<5	nd	nd
3	**1 c**	FeCl_3_ (5 %)	5	nd	nd
4	**1 a**	FeCl_3_ (50 %)	10	>20:1	>99
5	**1 a**	FeCl_3_ (100 %)	10	>20:1	>99
6^b^	**1 a**	FeCl_3_ (100 %)	<5	nd	nd
7	**1 a**	TMSOTf (20 %)	<5	nd	nd
8	**1 a**	TBSOTf (20 %)	<5	nd	nd
9	**1 a**	CF_3_COOH (20 %)	<5	nd	nd
10	**1 a**	TfOH (20 %)	<5	nd	nd
11	**1 a**	Tf_2_NH (20 %)	<5	nd	nd
12	**1 a**	BF_3_⋅Et_2_O (50 %)	25	>20:1	>99
13	**1 a**	BF_3_⋅Et_2_O (100 %)	50	>20:1	>99

[a] Reaction conditions: **1** (0.2 mmol), **2 a** (0.24 mmol) at rt for 1 h. [b] DCE was used as solvent at 100 °C. DCE=1,2‐dichloroethane; DCM=dichloromethane.

With suitable conditions in hand, different heteroatom‐tethered unsaturated alcohols were combined with aldehyde **2 a**. Vinylsilane‐derived substrate **1 d** reacts smoothly to product **3 d** in good yield (Table 2, entry 1). This compound can then be used for selective cleavage of the bridging chain to afford *syn*‐1,3‐diol **4 a** through modified Tamao–Fleming oxidation in excellent yield and perfect enantio‐ and diastereoselectivity.^7^ Oxygen‐tethered substrate **1 a** also reacts to the desired coupling product **3 c** and can then be cleaved to afford 1,4‐diol **4 b** by Baeyer–Villiger oxidation (entry 2).^8^ Alternatively, a thioether linker, such as in **1 e**, opens the possibility of reductive desulfuration leading to the *syn*‐methylpentanol motif (**4 c**).^9^ For the sake of completeness, we also show that coupling product **3 f** can be converted into the corresponding 1,5‐diol **4 d** by Saegusa oxidation^10^ and ozonolysis. Cleavage at either of the ketone positions (α or α′) is easily achieved by Baeyer–Villiger oxidation or Lieben haloform reaction to afford the corresponding 1,6‐diol **4 e** or carboxylic acid **4 f**, respectively.^11^

**Table 2:** Flexible asymmetric synthesis of the addition products of aldehydes with alkenes.

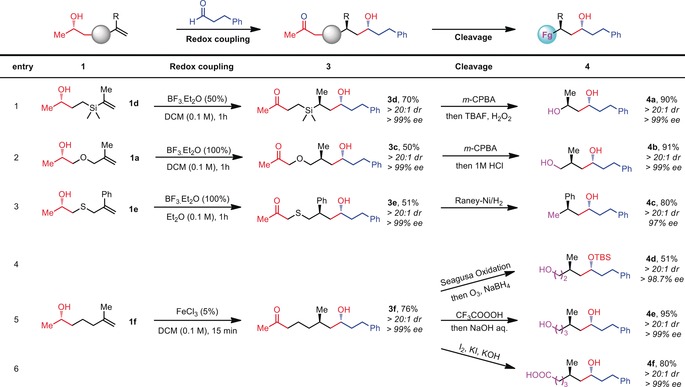

We then turned our attention towards using trisubstituted alkenes as substrates for the coupling reaction. In the event, we found that FeCl_3_ is an efficient catalyst to promote these reactions. As shown in Table 3, dimethyl‐substituted alkene **5 a** resulted in coupling product **6 a** in good yield and with perfect levels of enantio‐ and diastereoselectivity. Interestingly, cyclic systems perform particularly well (**6 b**–**6 e**). On the aldehyde component, α‐branched substrates and other carbonyls such as esters (cf. **6 g**) are tolerated. By using readily available α‐chiral aldehydes, it is possible to access products bearing four stereogenic centres (demonstrated by the stereotetrads **6 h**,**i**) with high diastereo‐ and enantioselectivity even for the labile 2‐phenylpropanal. Additionally, by using BF_3_⋅Et_2_O as the Lewis acid, aldol products can be employed as electrophiles with similarly high stereoselectivity (**6 j**).

**Table 3:** Scope of the redox‐neutral coupling of alkene‐bearing alcohols with aldehydes using Lewis acid catalysis.^[a]^

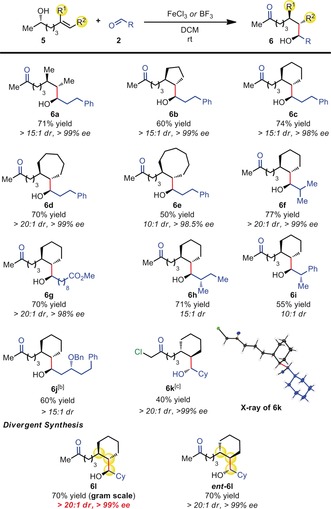

[a] Reactions were performed with **5** (0.2 mmol), aldehyde **2** (0.24 mmol), FeCl_3_ (20 % mol), r.t., 10 minutes. [b] for **6 j** the reaction conducted with BF_3_⋅Et_2_O (50 % mol) at r.t. for 30 minutes. [c] (*S*)‐Epichlorohydrin was used to make starting materials **5 j**.

Naturally, one is not restricted to unsaturated alcohols derived from propylene oxide. In order to unambiguously determine the absolute configuration of our products, we prepared a chiral unsaturated alcohol nucleophile from commercially available (*S*)‐epichlorohydrin. This resulted in the chlorinated coupling product **6 k**, which gave crystals suitable for X‐ray diffraction analysis. The advantage of using cheap and readily available propylene oxide, of which both enantiomers are commercially available at similar prices, is expressed in the possibility to rapidly access both enantiomers of a given coupling product as demonstrated for **6 l** and *ent*‐**6 l**. The preparation of **6 l** was achieved on a gram scale.

To demonstrate the synthetic utility of this coupling reaction, we first applied it to the stereoselective synthesis of 1,3,5‐triol **8** (Scheme 2 a), a polyol motif often found in bioactive compounds.^12^ To access this compound, we carried out ring‐opening of propylene oxide with silyl‐Grignard **7** to deliver the nucleophilic partner **1 d**. Redox‐neutral coupling with the aldol product **2 g** gives **8**, which can be directly converted into the target skipped triol **9** by Tamao–Fleming oxidation and benzyl‐deprotection. Finally, we sought to test our approach in the formal synthesis of (+)‐neopeltolide (Scheme 2 b), a marine macrolide with potent in vitro cytotoxicity towards a range of cancer cell lines.^13^ Interestingly, most of the reported total syntheses proceed through a common precursor (key fragment **11**, Scheme 2 b) carrying different O‐protecting groups. Starting from inexpensive, readily available starting materials, the redox‐neutral coupling described herein allows access to intermediate **11** 13c in only five operations from aldehyde **2 h**.

**Scheme 2:**
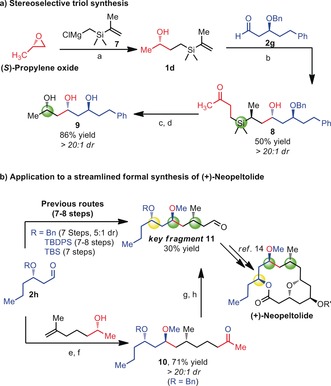
A) Stereoselective triol synthesis. Reaction conditions: a) Grignard reagent, CuCN (10 mol %); b) **1 g** (1 equiv), **2 h** (1.2 equiv), BF_3_⋅Et_2_O (50 mol %) in DCM (0.1 m), r.t.; c) **7** ( 1 equiv), *m*‐CPBA (1.5 equiv), then TBAF and H_2_O_2_; d) Pd/C (10 %) in MeOH; B) Formal synthesis of (+)‐Neopeltolide: e) **1 f** (1 equiv), **2 g** (1.2 equiv), BF_3_⋅Et_2_O (50 mol %) in DCM (0.1 m), r.t.; f) Me_3_OBF_4_ ( 4 equiv.), proton sponge (5 equiv) in DCM (0.1 m), r.t.; g) **9** (1 equiv), TBSOTf (2 equiv), lutidine (3 equiv), Et_2_O (0.3 m), r.t., then Pd(OAc)_2_ (10 mol %), O_2_ (1 atm), DMSO (0.1 m); 80 °C; h) O_3_, DCM (0.03 m), rt, then DMS. *m*‐CPBA=*m*‐chloroperoxybenzoic acid, TBAF=tetra‐*n*‐butylammonium fluoride, TBS=*tert*‐butyldimethylsilyl, OTf=triflate, DMS=dimethyl sulfide.

In summary, we have presented a flexible method for the enantioselective redox‐neutral coupling of aldehydes and alkenes. By installing heteroatoms in the bridging carbon chain, it is possible to access a wide variety of structures for the synthesis of chiral building blocks. We also showed that the use of trisubstituted alkenes enables the preparation of products bearing stereotriads with very high enantio‐ and diastereopurity in a single‐step operation. Application of this method to the synthesis of enantiopure triols and to the formal synthesis of (+)‐neopeltolide showcases the utility of these coupling products.

## Conflict of interest

The authors declare no conflict of interest.

## Supporting information

As a service to our authors and readers, this journal provides supporting information supplied by the authors. Such materials are peer reviewed and may be re‐organized for online delivery, but are not copy‐edited or typeset. Technical support issues arising from supporting information (other than missing files) should be addressed to the authors.

Supplementary
