# Supplementary material for: Diastereo‐ and Enantioselective Access to Stereotriads through a Flexible Coupling of Substituted Aldehydes and Alkenes
Source: Angew Chem Int Ed Engl. 2019 Mar 27;58(18):5887–90. doi: 10.1002/anie.201900801 (PMC6492014; doi:10.1002/anie.201900801)
Supplement: Supplementary file 1 — Supplementary [file ANIE-58-5887-s001.pdf]

Angewandte Chemie
Eine Zeitschrift der Gesellschaft Deutscher Chemiker

Supporting Information

# Diastereo- and Enantioselective Access to Stereotriads through a Flexible Coupling of Substituted Aldehydes and Alkenes

*Jing Li[+], Alexander Preinfalk[+], and Nuno Maulide**

anie_201900801_sm_miscellaneous_information.pdf

## Table of Contents


| | |
|---|---|
| 1. General Information | Page 2 |
| 2. Preparation of Starting Materials | Page 3-11 |
| 3. Synthesis and Functionalisation of the Coupling Products | Page 12-22 |
| 4. Synthesis of Stereotriads | Page 23-30 |
| 5. Applications | Page 31-37 |
| 6. Assignment of Absolute Configuration | Page 38-39 |
| 7. References | Page 40 |
| 8. NMR and HPLC Spectra | Page 41-98 |

# 1. General Information

Unless otherwise stated, all glassware was flame-dried before use and all reactions were performed under an atmosphere of argon. All solvents were distilled from appropriate drying agents prior to use. All reagents were used as received from commercial suppliers unless otherwise stated. All aldehydes were distilled or purified *via* flash column chromatography before use. Reaction progress was monitored by thin layer chromatography (TLC) performed on aluminium plates coated with silica gel $F_{254}$ with 0.2 mm thickness. Chromatograms were visualized by fluorescence quenching with UV light at 254 nm or by staining using potassium permanganate. Flash column chromatography was performed using silica gel 60 (230-400 mesh, Merck and co.). Neat infra-red spectra were recorded using a Perkin-Elmer Spectrum 100 FT-IR spectrometer. Wavenumbers ($\nu_{max}$) are reported in $cm^{-1}$. Mass spectra were obtained using a Finnigan MAT 8200 or (70 eV) or an Agilent 5973 (70 eV) spectrometer, using electrospray ionization (ESI). All $^{1}H$ NMR and $^{13}C$ NMR spectra were recorded using a Bruker AV-400 or AV-600 spectrometer at 300K. Chemical shifts were given in parts per million (ppm, δ), referenced to the solvent peak of $CDCl_3$, defined at δ = 7.26 ppm ($^{1}H$ NMR) and δ = 77.16 ($^{13}C$ NMR). Coupling constants are quoted in Hz (*J*). $^{1}H$ NMR splitting patterns were designated as singlet (s), doublet (d), triplet (t), quartet (q), pentet (p). Splitting patterns that could not be interpreted or easily visualized were designated as multiplet (m) or broad (br).

# 2. Preparation of Starting Materials

## 2.1 Synthesis of unsaturated alcohol substrates (Method A)

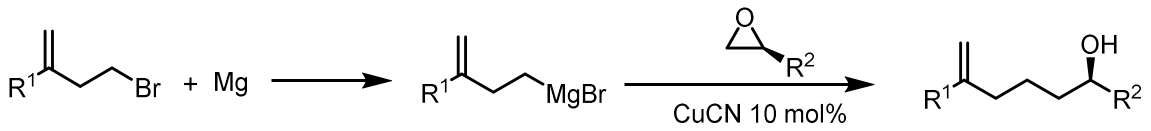

To a flame-dried Schlenk flask containing a suspension of Mg powder (15 mmol, 1.5 equiv.) in dry $Et_2O$ (10.0 mL) were added 5 drops 1, 2-dibromoethane and 5 drops of alkyl bromide at ambient temperature and the mixture was stirred for 5 minutes. Then alkyl bromide (12.0 mmol) was slowly added and the reaction was kept stirring at room temperature for 30 minutes.

The Grignard reagent was added to a suspension of (*S*)-(-)-Propylene Oxide (10 mmol) and CuCN (10 mol%) in THF (20 mL) at -78° C. The reaction mixture was allowed to warm to room temperature over 3 h. Then *sat.* $NH_4Cl$ solution was slowly added, extracted with ether and the combined organic phases were dried with anhydrous $MgSO_4$ and filtered. The solvent was removed under reduced pressure and the crude product was purified by column chromatography (heptane/ethyl acetate 4:1).

### (*S*)-6-methylhept-6-en-2-ol (1f)

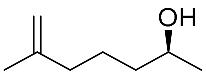

**1f** was prepared according to the general procedure and obtained as colorless oil in 78% yield.

**$^1$H NMR** (400 MHz, $CDCl_3$) δ 4.70 (s, 1H), 4.68 (s, 1H), 3.89 – 3.73 (m, 1H), 2.03 (t, *J* = 6.2 Hz, 2H), 1.71 (s, 3H), 1.61 – 1.39 (m, 4H), 1.36 (s, 1H), 1.19 (d, *J* = 6.2 Hz, 3H).

**$^{13}$C NMR** (100 MHz, $CDCl_3$) δ 145.9, 110.1, 68.2, 39.0, 37.8, 23.8, 23.7, 22.5.

**HRMS** (GC-MS): *m/z* calcd. for $(M-H_2O)^+$ 110.1096; found 110.1094.

$[\alpha]_D^{20}$ = 5.5 (C = 0.66, $CHCl_3$)

**FT-IR** (neat): 3360 (br), 2966, 2926, 2875, 2857, 1453, 1122 $cm^{-1}$

**(*S*)-1-chloro-5-(cyclohex-1-en-1-yl)pentan-2-ol (1k)**

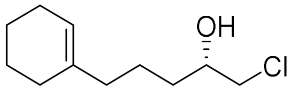


**1k** was prepared according to the general procedure and obtained as colorless oil in 70% yield.

**$^1$H NMR** ($CDCl_3$ 400 MHz): δ 5.33 (m, 1H), 3.73-3.74 (m, 1H), 3.57 (dd, *J* = 3.2, 10.8Hz, 1H), 3.41 (dd, *J* = 7.2 Hz, 11.2 Hz, 1H), 2.04, 2.06 (m, 1H), 1.82-1.92 (m, 6H), 1.37-1.57 (m, 8H).

**$^{13}$C NMR** ($CDCl_3$ 100 MHz): δ 137.2, 12.4, 71.4, 50.6, 37.8, 33.8, 28.2, 25.2, 23.5, 23.0, 22.6.

**HRMS** (EI): *m/z* calcd. for $(M - H_2O - HCl)^+$ 148.1252; found: 148.1243.

**FT-IR** (neat): 3377, 2926, 2862, 1657, 1434, 1327, 1275, 1261, 1194, 1133, 1065, 915, 750, 701 $cm^{-1}$.

$[\alpha]_D^{20}$ = 2.6 (C = 1.0, $CHCl_3$)

**(*S*)-5-(cyclohex-1-en-1-yl)pentan-2-ol (1l)**

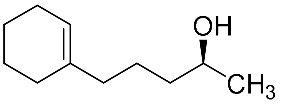


**1l**was prepared according to the general procedure and obtained as colorless, sticky oil in 78% yield.

**$^1$H NMR** ($CDCl_3$ 400 MHz): δ 5.23-5.24 (m, 1H), 3.69-3.73 (m, 1H), 2.17-2.22 (m, 2H), 2.10-2.14 (m, 2H), 1.99 (t, *J* = 6.0 Hz, 2H), 1.71-1.79 (m, 2H), 1.30-1.48 (m, 4H), 1.26 (b s, 1H), 1.09(d, *J*= 6.4 Hz, 3H).

**$^{13}$C NMR** ($CDCl_3$ 100 MHz): δ 137.6, 121.0, 68.2, 39.0, 37.9, 28.2, 25.2, 23.8, 23.5, 23.0, 22.6.

**HRMS** (EI) m/z calcd for $C_{10}H_{17}O$ $(M-CH_4)^+$ 153.1274; found: 153.1275.

**FT-IR** (neat): 3338, 2923, 2857, 2834, 1439, 1372, 1276, 1262, 1125, 1090, 939, 917, 837, 800, 764, 750 cm-1.

$[\alpha]_D^{20}$ = 6.9 (C = 1.0, $CHCl_3$)

## 2.2 Synthesis of unsaturated alcohol substrates (Method B)

**(*S*)-tert-butyl((5-iodopentan-2-yl)oxy)dimethylsilane (S-1)**

S-1 was prepared in 55% yield according to a known procedure.[1]

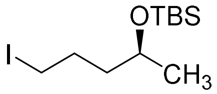


**S-1**

**General procedure for the synthesis of trisubstituted alkene-substrates**

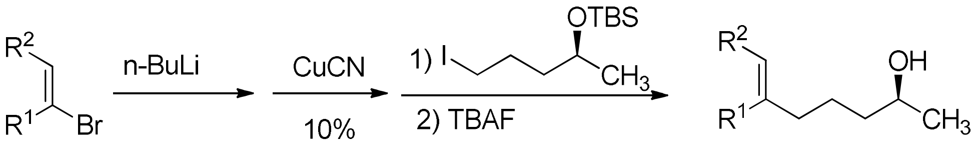


The required vinyl bromides are commercially available or were synthesized based on a previously reported procedure.[2]

Vinyl bromide (15 mmol, 1.5 equiv.) and dry THF (20.0 mL) were added to a flame dried Schlenk flask. The flask was placed in a -78 °C dry ice-acetone bath, n-BuLi was slowly added and the reaction was kept at the same temperature for 30 minutes. Then CuCN (10 mol%) was added in one portion, the reaction was allowed to warm to r.t. and stirred at this temperature for 1h. **S-1** was added in one portion at -78 °C, the reaction was stirred at the same temperature for 2 h and quenched with *sat.* $NH_4Cl$ solution. The biphasic mixture was extracted with ether, the combined organic phased were dried with anhydrous MgSO4, filtered and the solvent was removed under reduced pressure to afford the crude products. Purification by silica gel chromatography (Heptane/Ethyl acetate = 3/1) gave the desired products.

**(*S, E*)-6-methyloct-6-en-2-ol (1g)**

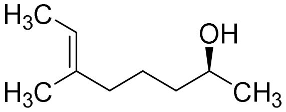


**1g** was prepared according to the general procedure and obtained as colorless, sticky oil in 85% yield.

**$^1$H NMR** ($CDCl_3$ 400 MHz): δ 5.13-5.18 (m, 1H), 3.72-3.76 (m, 1H), 1.90-1.98 (m, 2H), 1.53-1.54 (m, 3H), 1.50-1.53 (m, 3H), 1.32-1.48 (m, 4H), 1.14 (d, *J* = 6.0 Hz, 3H).

**$^{13}$C NMR** ($CDCl_3$ 150 MHz): δ 135.6, 118.5, 68.1, 39.5, 38.9, 24.0, 23.5, 15.5, 13.3.

**HRMS** (EI): *m/z* calcd. for $(M\text{-}CH_4)^+$ 127.1117; found: 127.1111.

**FT-IR** (neat): 3335, 2966, 2928, 2861, 1454, 1375, 1129, 970, 938, 815, 776, 735 $cm^{-1}$.

$[\alpha_D^{20}]$ = 4.1 (C = 1.0, $CHCl_3$)

**(*S*)-5-(cyclopent-1-en-1-yl)pentan-2-ol (1h)**

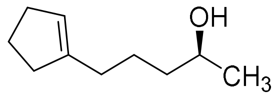


**1h** was prepared according to the general procedure and obtained as colorless, sticky oil in 50% yield.

**$^1$H NMR** ($CDCl_3$ 400 MHz): δ 5.22-5.25 (m, 1H), 3.67-3.75 (m, 1H), 2.17-2.22 (m, 2H), 2.10-2.15 (m, 2H), 1.99 (t, *J* = 6.0 Hz, 2H), 1.71-1.79 (m, 2H), 1.31-1.48 (m, 4H), 1.25 (m, 1H), 1.09 (d, *J* = 6.4 Hz, 3H).

**$^{13}$C NMR** ($CDCl_3$ 100 MHz): δ 144.6, 123.4, 68.1, 39.4, 31.1, 23.9, 23.5, 23.4.

**HRMS** (ESI): *m/z* calcd. for C10H19O (M + H)+ 155.1430; found: 155.1428.

**FT-IR** (neat): 3367, 2932, 2846, 1457, 1374, 1123, 1081, 1035, 764, 750 $cm^{-1}$.

$[\alpha_D^{20}]$ = 3.0 (C = 1.0, $CHCl_3$)

**(*S*)-5-(cyclohept-1-en-1-yl)pentan-2-ol (1i)**

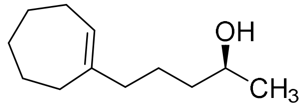


**1i** was prepared according to the general procedure and obtained as colorless, sticky oil in 85% yield.

**$^1$H NMR** ($CDCl_3$ 400 MHz): δ 5.48-5.51 (m, 1H), 3.74-3.78 (m, 1H), 2.00-2.06 (m, 4H), 1.92-1.95 (m, 2H), 1.05-1.71 (m, 2H), 1.35-1.45 (m, 10H), 1.14 (d, *J* = 6.0 Hz, 3H).

**$^{13}$C NMR** ($CDCl_3$ 150 MHz): δ 144.5, 126.1, 68.1, 40.1, 38.9, 32.7, 32.6, 28.3, 27.4, 26.8, 24.0, 23.5.

**HRMS** (EI): *m/z* calcd. for $C_{12}H_{22}O$ $(M)^+$ 182.1671; found: 182.1662.

**FT-IR** (neat): 3341, 2917, 2847, 1446, 1373, 1275, 1129, 1092, 1067, 987, 941, 845, 764, 750 $cm^{-1}$.

$[\alpha_D^{20}]$ = 5.3 (C = 1.0, $CHCl_3$)

**(*S*, *E*)-5-(cyclooct-1-en-1-yl)pentan-2-ol (1j)**

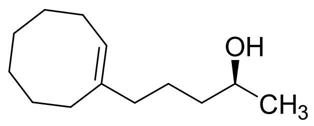


**1j** was prepared according to the general procedure and obtained as colorless, sticky oil in 75% yield.

**$^1$H NMR** ($CDCl_3$ 400 MHz): δ 5.23-5.29 (m, 1H), 3.72-3.76 (m, 1H), 2.05-2.10 (m, 2H), 1.99-2.04 (m, 2H), 1.90-1.94 (m, 2H), 1.32-1.49 (m, 12H), 1.12 (d, $J$ = 6.0Hz, 3H).

**$^{13}$C NMR** ($CDCl_3$ 100 MHz): δ 140.6, 123.9, 68.2, 39.2, 37.4, 30.0, 28.8, 28.8, 26.5, 26.3, 26.3, 25.9, 24.2, 23.5.

**HRMS** (EI): *m/z* calcd. for $(M)^+$ 196.1827; found: 196.1817.

**FT-IR** (neat): 3367, 2932, 2846, 1457, 1374, 1123, 1081, 1035,764 $cm^{-1}$.

$[\alpha_D^{20}]$ = 6.5 (C = 1.0, $CHCl_3$)

## 2.3 Synthesis of heteroatom-bridged substrates

### (*S*)-4-(dimethyl(prop-1-en-2-yl)silyl)butan-2-ol (1d)

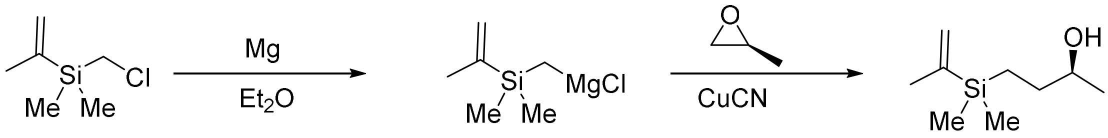


To a flame-dried Schlenk flask containing a suspension of Mg powder (15 mmol, 1.5 equiv.) in dry $Et_2O$ (10.0 mL) were added 5 drops 1, 2-dibromoethane and 5 drops of (chloromethyl)dimethyl(prop-1-en-2-yl)silane and the mixture was stirred for 5 minutes at ambient temperature. Then (chloromethyl)dimethyl(prop-1-en-2-yl)silane (12.0 mmol) was slowly added and the reaction was kept stirring at room temperature for 30 minutes to give ((dimethyl(prop-1-en-2-yl)silyl)methyl)magnesium chloride (**7**). Grignard reagent **7** was added to a suspension of (*S*)-(-)-Propylene Oxide (10 mmol) and CuCN (10 mol%) in $Et_2O$ (20 mL) at -78° C. The reaction mixture was allowed to warm to room temperature over 3 h. Then *sat.* $NH_4Cl$ solution was slowly added, the mixture was extracted with ether and the combined organic phases were dried with anhydrous $MgSO_4$ and filtered. The solvent was removed under reduced pressure and the crude product was purified by column chromatography (heptane/ethyl acetate 4:1) to give the product as colorless oil in 90% yield.

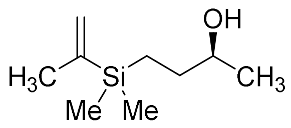


**$^{1}$H NMR** ($CDCl_3$ 400 MHz): δ 5.57-5.59 (m, 1H), 5.24-5.25 (m,1H), 3.68-3.73 (m,1H), 1.81 (t, *J* = 1.6 Hz, 3H), 1.18 (d, *J* = 6.0 Hz,3H), 1.37-1.45 (m, 2H), 0.64-0.72 (m,1H), 0.48-0.56 (m, 1H), 0.08 (s, 6H).

**$^{13}$C NMR** ($CDCl_3$ 100 MHz): δ 146.8, 125.2, 70.4, 33.4, 22.8, 22.6, 10.2, -3.9, -4.0.

**HRMS** (EI): *m/z* calcd. for $(M\text{-}C_3H_7)^+$ 129.0736; found: 129.0728.

**FT-IR** (neat): 3333, 2957, 2926, 1448, 1275, 1255, 1180, 1119, 1069, 1019, 920, 836, 767, 750 $cm^{-1}$.

$[\alpha_D^{20}]$ = 4.9 (C = 1.0, $CHCl_3$)

**(*S*)-1-((2-methylallyl)oxy)propan-2-ol (1a)**

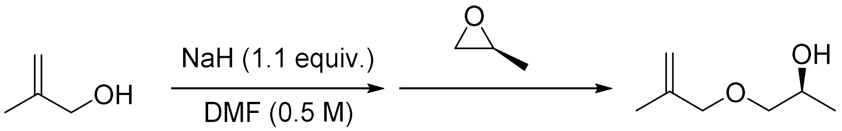

To a flame-dried Schlenk flask containing a suspension of NaH powder (33 mmol, 1.1 equiv.), which was previously washed with pentane (50 mL), DMF (50 mL) was added. Allylic alcohol (30 mmol) was added dropwise. After completion of addition of allyl alcohol, the reaction mixture was further stirred for 30 minutes and HMPA (5 equiv.) was added. Then (S)-(-)-Propylene Oxide (35 mmol) was added in one portion at r.t. and the reaction mixture was stirred at 50 °C for 12 h. Upon completion of the reaction, s*at.* $NH_4Cl$ solution was added and extracted with pentane. The combined organic phases were dried with anhydrous $MgSO_4$, filtered and the solvent was removed under reduced pressure to afford the crude product. Purification by silica gel chromatography (Pentane/DCM = 1/1) afforded the clean product as colorless oil in 50% yield.

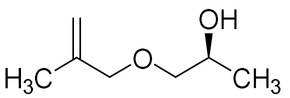

**$^1$H NMR** ($CDCl_3$ 400 MHz): δ 4.98-4.99 (m, 1H), 4.92-4.93 (m, 1H), 3.96-4.04 (m. 1H), 3.95 (s, 2H), 3.43 (dd, *J* = 3.2, 9.4 Hz, 1H), 3.23 (dd, *J* = 8.0, 9.4 Hz, 1H), 1.77 (s, 3H), 1.80 (d, J = 6.4 Hz, 3H).

**$^{13}$C NMR** ($CDCl_3$ 100 MHz): δ 141.9, 112.3, 75.4, 75.1, 66.4, 19.4, 18.6.

**HRMS** (EI): *m/z* calcd. for $(M\text{-}CH_3)^+$ 115.0754; found: 115.0748.

**FT-IR** (neat): 3425, 2972, 2859, 1659, 1451, 1374, 1260, 1091, 984, 898, 750 $cm^{-1}$.

$[\alpha]_D^{20}$ = 28.9 (C = 1.0, $CHCl_3$)

**(*S*)-1-((2-phenylallyl)thio)propan-2-ol (1e)**

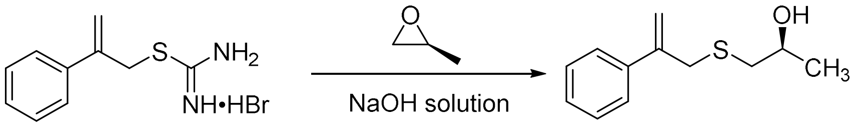


2-phenylallyl carbamimidothioate salt was dissolved in water, solid NaOH (5 equiv.) was slowly added followed by (*S*)-2-methyloxirane (1.2 equiv.), which was added in one portion. Then the reaction mixture was stirred for 2 h and extracted three times with $Et_2O$. The organic phase was dried with anhydrous MgSO4, filtered and the solvent was removed under reduced pressure to afford the crude product. Purification by silica gel chromatography (Heptane: Ethyl acetate = 4;1) afforded the pure product as sticky oil (95% yield).

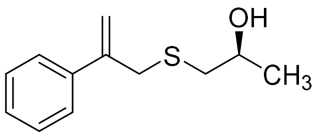


**$^1$H NMR** ($CDCl_3$ 600 MHz): δ 7.48-7.49 (m, 2H), 7.32-7.39 (m, 3H), 5.32 (s, 1H), 5.24 (s, 1H), 3.86-3.89 (m, 1H), 3.65 (s, 2H), 2.70 (dd, *J* = 3.0, 12 Hz, 1H), 2.42-2.45 (m, 2H), 1.25 (d, J = 5.2 Hz, 3H).

**$^{13}$C NMR** ($CDCl_3$ 150 MHz): δ 143.5, 139.0, 128.4, 128.0, 126.3, 115.4, 65.3, 40.6, 36.5, 22.0.

**HRMS** (ESI): *m/z* calcd. for $(M)^+$ 208.0922; found: 208.0917.

**FT-IR** (neat): 3366, 2968, 2911, 1623, 1494, 1445, 1407, 1302, 1124, 1069, 1038, 902, 777, 698 $cm^{-1}$.

$[\alpha_D^{20}]$ = 62.8 (C = 1.0, $CHCl_3$)

## 2.4. Synthesis of aldehyde 2f

### (R)-3-(benzyloxy)-5-phenylpentanal (2f)

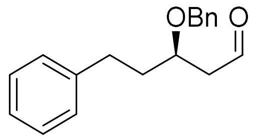

The aldehyde was prepared based on a previously reported procedure in 75% yield as colorless sticky oil.[3]

**$^1$H NMR** ($CDCl_3$ 600 MHz): δ 9.8 (t, *J* = 2.0 Hz, 1H), 7.30-7.30 (m, 7H), 7.19-7.25 (m, 3H), 4.58 (s, 2H), 3.98-4.04 (m, 1H), 2.60-2.82 (m, 4H), 1.89-2.09 (m, 2H).

**$^{13}$C NMR** ($CDCl_3$ 100 MHz): δ 141.5, 138.1, 128.5, 128.5, 128.4, 127.9, 127.8, 126.0, 73.7, 71.4, 48.3, 36.1, 31.4.

$[\alpha]_D^{20}$ = 2.0 (C = 1.0, $CHCl_3$)

# 3. Synthesis and Functionalisation of the Coupling Products

**GP 1**: Alcohol (0.2 mmol) was diluted with 2 mL 1,2-dichloroethane and brought to the reaction temperature using an oil bath. Aldehyde (0.24 mmol, 1.2 eq.) and $FeCl_3$ (0.01 mmol, 5 mol%) were successively added and the reaction was heated for 5 minutes. Water (0.2 mL) was added and the reaction was allowed to cool to room temperature with stirring. The reaction mixture was filtered over silica, eluted with dichloromethane and the solvent was removed under reduced pressure to obtain crude sticky product. Purification by flash column chromatography afforded the pure products.

**GP 2**: Alcohol (0.2 mmol) was diluted with 2 mL dichloromethane. Aldehyde (0.24 mmol, 1.2 eq.) and $FeCl_3$ (0.04 mmol, 20 mol%) were successively added and the reaction was stirred at r.t. until Alcohol was consumed. Water (0.2 mL) was added and the reaction was allowed to cool to room temperature with stirring. The reaction mixture was filtered over silica, eluted with dichloromethane and the solvent was removed under reduced pressure to obtain crude sticky product. Purification by flash column chromatography afforded the pure products.

**GP 3**: Alcohol (0.2 mmol) was diluted with 2 mL dichloromethane. Aldehyde (0.24 mmol, 1.2 eq.) and $BF_3 \cdot Et_2O$ were successively added and the reaction was stirred at r.t. for 2 h. Water (0.2 mL) was added and the reaction was allowed to cool to room temperature with stirring. The reaction mixture was filtered over silica, eluted with dichloromethane and the solvent was removed under reduced pressure. Purification by flash column chromatography afforded the pure products.

**Caution**! To make stereotriads, the reactions are sensitive to the purity of the aldehydes. In order to obtain the reported results, the aldehyde must always be distilled or purified by column chromatography before use. If an impurity is present in the starting materials, the reaction does not reach full conversion. In this case, another dose of catalyst can be added without dramatically influencing the yield and *ee*-values.

**4-(((2*S*,4*R*)-4-hydroxy-6-phenylhexan-2-yl)dimethylsilyl)butan-2-one** (**3d**)

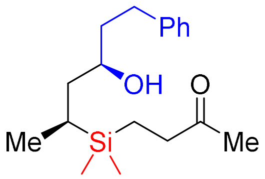

Compound **3d** was prepared according to the **GP3** (50% $BF_3\,Et_2O$) at room temperature and obtained in 70% yield as colorless oil after column chromatography (2:1 of heptane:EtOAc).

$^1$H NMR spectroscopic analysis of the unpurified reaction mixture indicated >**20:1 dr**.

HPLC analysis (Column: Chiralcel OD-H 250×4.6 mm; Solvent System: n-Heptane + 0.1% IPA: EtOH =95:5); Flow: 0.70 mL/min, 254 nm) indicated > **99% ee** ($t_{minor}$ = 12.5 min, $t_{major}$ = 14.8 min)

**$^1$H NMR** ($CDCl_3$ 600 MHz): δ 7.27-7.30 (m, 2H), 7.17-7.21 (m, 3H), 3.75 (m, 1H), 2.78-2.83 (m, 1H), 2.66-2.71 (m, 1H), 2.39 (t, *J* = 5.6 Hz, 2H), 2.15 (s, 3H), 1.71-1.80 (m, 2H), 1.46-1.50 (m, 1H), 0.93 (b s, 3H), 0.92-0.96 (m, 1H), 0.74-0.80 (m, 2H), -0.04 (s, 3H), -0.05 (s, 3H).

**$^{13}$C NMR** ($CDCl_3$ 150 MHz): δ 209.9, 142.1, 128.4, 125.8, 68.2, 40.0, 39.0, 38.2, 32.3, 29.3, 13.9, 13.3, 6.9, -5.5, -5.5.

**HRMS** (ESI): *m/z* calcd. for $C_{18}H_{30}NaO_2Si$ $(M + Na)^+$ 329.1907; found: 329.1906.

**FT-IR** (neat): 2947, 2865, 1712, 1454, 1412, 1358, 1065, 839, 764, 700cm$^{-1}$.

$[\alpha]_D^{20}$= 37.5 (C = 0.8, $CHCl_3$)

**Synthesis of (2*S*,4*R*)-6-phenylhexane-2,4-diol (4a)**

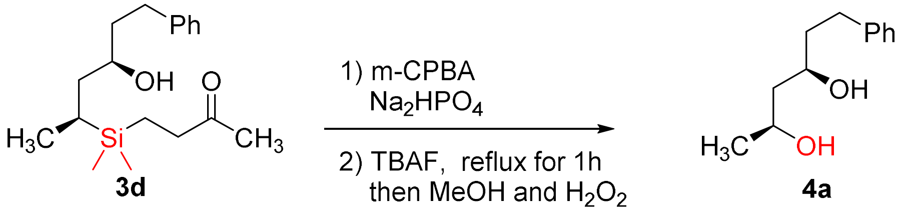


**Procedure:** To a 25-mL, round-bottomed flask was added **3t** (1.0 mmol), $Na_2HPO_4$ (2.0 mmol, 2.0 eq.) and dichloromethane (10 mL). The suspension was stirred vigorously at room temperature, then *m*-CPBA solid was slowly added by several portion. The solution was stirred at r.t. for 12. The reaction was quenched with $Na_2S_2O_3$ (3.2 mmol, 16 eq.) and concentrated in vacuo. The crude residue was redissolved in THF (5 mL) and 3 mL TBAF was added and reflex for 1h. Then 37% H2O2 (64 equiv.) was added at r.t., which

further stirred for 8 h at the same temperature. The reaction was quenched with $Na_2S_2O_3$ (3.2 mmol, 16 eq.) extracted with ether and dried over $MgSO_4$. Purification by flash column chromatography (1:1 heptane/EtOAc) afforded (2*S*,4*R*)-6-phenylhexane-2,4-diol10 as colorless oil in 90% yield.

$^1$H NMR spectroscopic analysis of the unpurified reaction mixture indicated >20:1 dr.

HPLC analysis (Column: Chiralcel OD-H 250×4.6 mm; Solvent System: n-Heptane + 0.1% IPA:EtOH = 95:5; Flow: 1.0 mL/min, 210 nm; indicated > 99% ee ($t_{minor}$ = 21.7 min, $t_{major}$ = 18.5 min)

**$^1$H NMR** ($CDCl_3$ 400 MHz): δ 7.17-7.22 (m, 2H), 7.08-7.12 (m, 3H), 3.91-3.99 (m, 1H), 3.76-3.82 (m, 1H), 3.37 (*b* s, 2H), 2.55-2.72 (m, 2H), 1.62-1.77 (m, 2H), 1.41-1.54 (m, 2H), 1.12 (d, J = 6.0 Hz, 3H).

**$^{13}$C NMR** ($CDCl_3$ 100 MHz): δ 142.0, 128.4, 125.9, 72.2, 69.1, 44.6, 39.8, 31.7, 24.3.

**HRMS** (ESI): *m/z* calcd. for C12H18NaO2 (M + Na)+ 217.1199; found: 217.1198.

$[\alpha]_D^{20}$= 22.4 (c= 1.0, $CHCl_3$)

**FT-IR** (neat): 3311, 29312860, 1495, 1453, 1320, 1276, 1132, 1076, 932, 838, 748, 698 cm$^{-1}$.

**<u>1-(((2*S*,4*R*)-4-hydroxy-2-methyl-6-phenylhexyl)oxy)propan-2-one</u>** (**3c**)

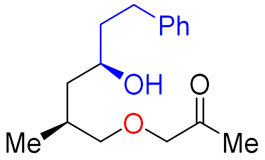


Compound **3c** was prepared according to the **GP3** at r.t. and obtained in 50% yield as colorless oil after column chromatography (2:1 heptane:EtOAc).

$^1$H NMR spectroscopic analysis of the unpurified reaction mixture indicated **> 20: 1 dr**.

HPLC analysis (AD-H column, 3% iPrOH/hexanes, 0.80 mL/min, 254 nm) indicated **> 99% ee** ($t_{minor}$ = 29.8 min, $t_{major}$ = 34.1 min).

**$^1$H NMR** ($CDCl_3$ 600 MHz): δ 7.26-7.29 (m, 2H), 7.16-7.21 (m, 3H), 4.04 (s, 2H), 3.69-3.73 (m, 1H), 3.37 (dd, J = 5.4, 7.2 Hz, 1H), 3.32 (dd, *J*= 7.2, 7.3 Hz, 1H), 2.78-2.82 (m, 1H), 2.66-2.71 (m, 1H), 2.14 (s, 3H), 1.98-2.03 (m, 1H), 1.72-1.81 (m, 2H), 1.54-1.59(m, 1H), 1.36-1.40 (m, 1H), 0.93 (d, J = 6.6 Hz).

$^{13}$**C NMR** ($CDCl_3$ 100 MHz) δ 206.6, 142.2, 128.4, 128.3, 125.6, 77.7, 76.3, 69.5, 42.7, 40.0, 32.1, 31.2, 26.2, 17.5.

**HRMS** (ESI): *m/z* calcd. for $C_{16}H_{24}NaO_3$ $(M + Na)^+$ 287.1618; found: 287.1613.

**FT-IR** (neat): 3721, 3006, 2990, 1726, 1459, 1123 $cm^{-1}$

$[\alpha]_D^{20}$= -5.0 (C = 1.0, $CHCl_3$)

**Synthesis of (2*S*,4*R*)-2-methyl-6-phenylhexane-1,4-diol (4b)**

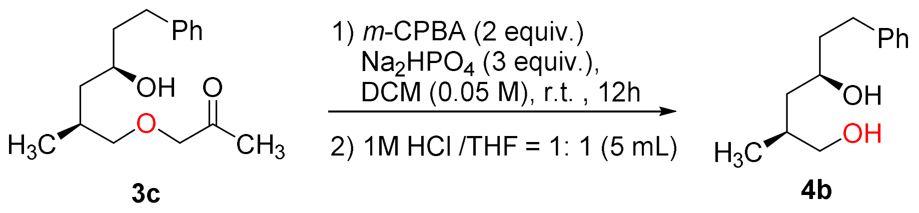


**Procedure:** To a 25-mL, round-bottomed flask was added **3c** (1.0 mmol), $Na_2HPO_4$ (2.0 mmol, 2.0 eq.) and dichloromethane (10 mL). The suspension was stirred vigorously at room temperature, then *m*-CPBA solid was slowly added in several portions. The solution was stirred for 12 h at r.t. °C, by which time $^1$H NMR analysis had indicated consumption of the starting material. The reaction was quenched with $Na_2S_2O_3$ (3.2 mmol, 16 eq.) and concentrated in vacuo. The crude residue was redissolved in THF : 1M HCl = 1:1 (5 mL). The reaction was stirred at r.t. for 3 h, then extracted with ether and dried over $MgSO_4$. Purification by flash column chromatography (1:1 heptane/EtOAc) afforded (2*S*,4*R*)-2-methyl-6-phenylhexane-1,4-diol **4b** as off-yellow oil in 91% yield.

$^1$H NMR spectroscopic analysis of the unpurified reaction mixture indicated >20:1 dr.

HPLC analysis (Column: Chiralpak IC 250×4.6 mm; Solvent System: n-Heptane + 0.1% $^i$PrOH: $^i$PrOH =95:5); Flow: 1 mL/min, 254 nm) indicated > 99% ee ($t_{minor}$ = 17.3 min, $t_{major}$ = 19.9 min)

$^1$**H NMR** ($CDCl_3$ 600 MHz): δ 7.19-7.23 (m, 2H), 7.09-7.14 (m, 2H), 3.62-3.68 (m, 1H), 3.51 (dd, J = 4.4, 10.8 Hz, 1H), 3.32 (dd, *J* = 7.6 Hz, 10.4 Hz, 1H), 2.58-2.76 (m, 2H), 2.76 (*b* s, 2H), 1.69-1.80 (m, 3H), 1.36-1.47 (m, 2H), 0.84 (d, *J* = 6.8 Hz, 3H).

$^{13}$**C NMR** ($CDCl_3$ 150 MHz): δ 206.6, 142.2, 128.3, 128.3, 125.6, 77.7, 76.3, 69.5, 42.7, 40.0, 32.1, 31.2, 26.2, 17.5.

**HRMS** (ESI): *m/z* calcd. for $C_{13}H_{20}NaO_2$ $(M + Na)^+$ 231.1356; found: 231.1353.

**FT-IR** (neat): 3319, 2927, 1455, 1276.1261, 1033, 764,750 $cm^{-1}$.

$[\alpha]_D^{20}$= -7.0 (C = 0.7, $CHCl_3$)

**<u>1-(((2*R*,4*R*)-4-hydroxy-2,6-diphenylhexyl)thio)propan-2-one</u>** (**3e**)

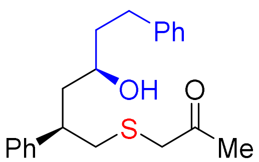


Compound **3e** was prepared according to the **GP3** at r.t. in diethyl ether and obtained in 51% yield as colorless oil after column chromatography (2:1 heptane:EtOAc).

$^{1}$H NMR spectroscopic analysis of the unpurified reaction mixture indicated **>20:1 dr**.

HPLC analysis (Column: Chiralcel OD-H 250×4.6 mm; Solvent System: (*n*-Heptane + 0.1% *i*-PrOH) : *i*-PrOH = 8:2; Flow: 1.0 mL/min, 210 nm; indicated **> 99% ee** ($t_{minor}$ = 15.2 min, $t_{major}$ = 13.2 min)

**$^{1}$H NMR** ($CDCl_3$ 600 MHz): δ 7.33-7.36 (m, 2H), 7.17-7.27 (m, 6H), 7.12-7.13 (m, 2H), 3.37-3.40 (m, 1H), 3.11-3.16 (m, 1H), 3.13 (*d*, J = 6Hz, 2H), 2.78 (*d*, J = 6Hz, 2H), 2.67-2.72 (m, 1H), 2.55-2.60 (m, 1H), 2.25 (s, 3H), 1.93-1.98 (m, 1H), 1.78-1.83 (m, 1H), 1.71-1.74 (m, 2H), 1.47 (d, *J* = 1.2Hz, 1H).

**$^{13}$C NMR** ($CDCl_3$ 150 MHz): δ 204.0, 142.9, 141.9, 128.6, 128.3, 128.2, 127.7, 126.8, 125.7, 69.1, 42.7, 42.2, 41.9, 40.0, 39.3, 32.0, 27.7.

**HRMS** (ESI): *m/z* calcd. for $C_{21}H_{26}NaO_2S$ $(M + Na)^+$ 365.1546; found: 365.1541.

**FT-IR** (neat): 3423, 3026, 2929, 2858, 1708, 1494, 1453, 1408, 1360, 1276, 1159, 1030, 750, 700 $cm^{-1}$.

$[\alpha]_D^{20}$= 5.8 (C = 1.0, $CDCl_3$)

**<u>Synthesis of (3*R*,5*R*)-1,5-diphenylhexan-3-ol (4c)</u>**

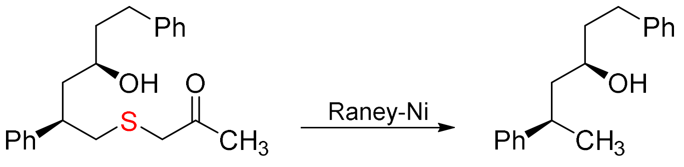


**3e** **4c**

**Procedure:** To a 25-mL, round-bottomed flask was added **3e** (1.0 mmol) in MeOH (10 mL) under $H_2$ balloon. The solution was stirred vigorously at room temperature, then

Raney-Ni (23 equiv.) solid was added by one-portion. The solution was stirred at r.t. until the starting materials was consumed. The reaction was filtered via celite concentrated in vacuo. The crude residue was purified by flash column chromatography (5:1 heptane/EtOAc) afforded (3*R*,5*R*)-1,5-diphenylhexan-3-ol **9** as sticky colorless oil in 80% yield.

$^1$H NMR spectroscopic analysis of the unpurified reaction mixture indicated >20:1 dr. HPLC analysis analysis of the Fmoc-derivatized analog (Column: Chiralcel OD-H 250× 4.6 mm; Solvent System: (n-Heptane + 0.1% i-PrOH):i-PrOH = 99:1; Flow: 0.7 mL/min, 210 nm; indicated 97% ee ($t_{minor}$ = 9.5 min, $t_{major}$ = 16.8 min)

**$^1$H NMR** ($CDCl_3$ 400 MHz): δ 7.02-7.24 (m, 10H), 3.30-3.36 (m, 1H), 2.88-2.97 (m, 1H), 2.57-2.64 (m, 1H), 2.45-2.52 (m, 1H), 1.60-1.74 (m, 4H), 1.19 (d, J = 7.0 Hz, 3H).

**$^{13}$C NMR** ($CDCl_3$ 100 MHz): δ 146.7, 142.1, 128.5, 128.4, 128.3, 127.1, 126.1, 126.8, 69.5, 45.8, 39.9, 36.5, 32.0, 23.3.

**HRMS** (ESI): *m/z* calcd. for $C_{18}H_{22}NaO$ $(M + Na)^+$ 277.1563; found: 277.1559.

**FT-IR** (neat): 3352, 2924, 2866, 1602, 1493, 1452, 1276,910,762, 749, 697 $cm^{-1}$.

$[\alpha]_D^{20}$= -3.8 (C = 1.0, $CHCl_3$)

**<u>(6*R*,8*R*)-8-hydroxy-6-methyl-10-phenyldecan-2-one</u> ((*R*, *R*)-3f)**

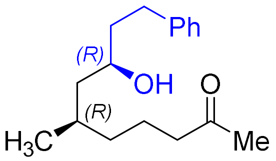


Compound **(*R*, *R*)-3f** was prepared according to the **GP1** at 100°C and obtained in 76% yield as colorless oil after column chromatography (3:1 heptane:EtOAc).

$^1$H NMR spectroscopic analysis of the unpurified reaction mixture indicated **> 20:1 dr**.

HPLC analysis (Chiralcel OD-H 250×4, 6 mm, (n-Heptane+0.1% i-PrOH):EtOH = 95:5, 0.70 mL/min, 210 nm) indicated **> 99% ee** ($t_{minor}$ = 13.93 min, $t_{major}$ = 15.84 min).

**$^1$H NMR** ($CDCl_3$ 400 MHz): δ 7.28-7.32 (m, 2H), 7.12-7.23 (m, 3H), 3.67-3.82 (m, 1H), 2.77-2.85 (m,1H), 2.65-2.73 (m, 1H), 2.42 (t, *J* = 7.2 Hz, 2H), 2.14 (s, 3H), 1.74-1.80 (m, 2H), 1.48-1.71 (m, 4H),1.14-1.33 (m, 4H), 0.91 (d, J = 6.4 Hz, 3H).

$^{13}$**C NMR** ($CDCl_3$ 150 MHz): δ 209.3, 142.2, 128.4, 125.8, 69.1, 44.8, 43.9, 40.1, 37.2, 32.1, 29.9, 29.1, 21.2, 19.2.

**HRMS** (ESI): *m/z* calcd. for $C_{17}H_{26}NaO_2$ $(M + Na)^+$ 327.2295; Found: 327.2291.

**FT-IR** (neat): 3423, 2966, 1710, 1495, 1454, 1409, 1361, 1165, 1054, 750, 700 $cm^{-1}$.

$[\alpha]_D^{20}$ = -5.6 (C = 1.0, $CHCl_3$)

**Synthesis of (6*R*,8*R*,*E*)-8-hydroxy-6-methyl-10-phenyldec-3-en-2-one**

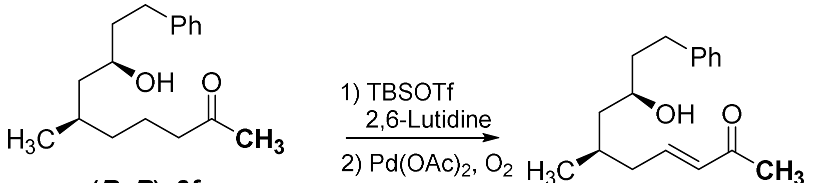


To a solution of **(*R*, *R*)-3f** (0.3 mmol) in 1 mL THF was added lutidine (1.2 mmol, 4 eq.) at room temperature. After stirring for 10 minutes, *tert*-butyldimethylsilyl trifluoromethanesulfonate (0.9 mmol, 3 eq.) was added dropwise and the reaction mixture was stirred for 13 hours. The reaction was quenched with saturated aqueous ammonium chloride (3 mL), extracted with ether and dried over $Na_2SO_4$. The solvent was removed under reduced pressure giving the product as 2:1 mixture of diastereoisomers. Remaining lutidine was removed using filtration over celite, layered with a small pad of silica. The eluent (heptane + 1% trimethylamine) was cooled to -78° C before using.

Previously obtained silyl enol ether and $Pd(OAc)_2$ (0.03 mmol, 10 mol%) were dissolved in 3 mL DMSO, frozen in a bath of liquid nitrogen, evacuated and backfilled with pure oxygen using a balloon. The Schlenk tube was directly taken out of the nitrogen bath, warmed to room temperature and heated at 80° C for 13 h. Upon completion, the reaction mixture was diluted with water and extracted with dichloromethane three times. The combined organic phases were dried over $Na_2SO_4$ and the solvent removed under reduced pressure. The crude product was purified using flash column chromatography (20:1-9:1 heptane/EtOAc) to give **(6*R*,8*R*,*E*)-8-hydroxy-6-methyl-10-phenyldec-3-en-2-one** in 57% yield over 2 steps.

$^1$H NMR spectroscopic analysis of the unpurified reaction mixture indicated **> 20: 1 dr**.

HPLC analysis (Chiralpak IA, (n-heptane + 0.1% $^i$PrOH) : $^i$PrOH = 98.5:1.5, 0.70 mL/min, 210 nm) indicated **> 99% ee** ($t_{minor}$ = 7.9 min, $t_{major}$ = 7.4 min).

**$^1$H NMR** (400 MHz, $CDCl_3$) δ 7.33 – 7.23 (m, 2H), 7.18 (dd, *J* = 10.0, 4.4 Hz, 3H), 6.76 (dt, *J* = 15.8, 7.3 Hz), 6.08 (dt, *J* = 15.9, 1.2 Hz, 1H), 3.79 (td, *J* = 9.6, 5.4 Hz, 1H), 2.63 (dd, *J* = 9.7, 6.9 Hz, 2H), 2.24 (s, 3H, H1), 2.23–2.14 (m, 1H), 2.14–2.06 (m, 1H), 1.91–1.73 (m, 2H), 1.59–1.51 (m, 1H), 1.33–1.20 (m, 2H), 0.93–0.91 (m, 12H), 0.06 (s, 3H), 0.04 (s, 3H).

**$^{13}$C NMR** (100 MHz, $CDCl_3$) δ 198.6, 147.0, 142.6, 132.7, 128.5(2C), 128.5 (2C), 125.9, 69.8, 44.1, 40.7, 39.9, 31.5, 29.1, 27.1, 26.1 (3C), 19.9, 18.2, -4.0 (TBS), -4.3 (TBS).

**HRMS** (ESI): *m/z* calcd. for $[M+Na]^+$ 397.2533, found 397.2535.

**FT-IR** (neat): 2954, 2928, 1676, 1628, 1459, 1361, 1253, 1064, 980, 835, 774, 748, 700 $cm^{-1}$.

$[\alpha]_D^{20}$ = -12.2 (C = 1, $CHCl_3$).

**Synthesis of (3*R*,5*R*)-5-((tert-butyldimethylsilyl)oxy)-3-methyl-7-phenylheptan-1-ol (4d)**

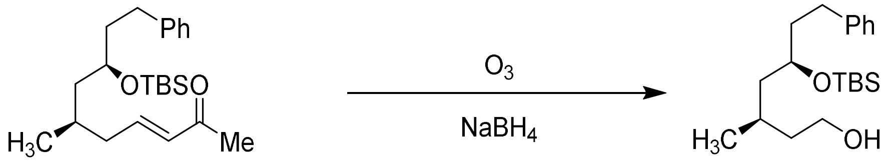

**4d**

**(6*R*,8*R*,*E*)-8-((tert-butyldimethylsilyl)oxy)-6-methyl-10-phenyldec-3-en-2-one**(0.7 mmol) was dissolved in 7 mL dichloromethane and cooled to -78° C. Ozone was bubbled through the solution until a blue color was observed (ca. 5 minutes). The ozone generator was turned off and oxygen was bubbled through until the solution became colorless. Sodium borohydride (10.7 mmol, 15 eq.) in methanol (7 mL) was added, the reaction mixture was slowly warmed up to 0°C and stirred at this temperature for 13 h. The reaction was quenched with saturated aqueous $NaHCO_3$, extracted with ether, dried over $Na_2SO_4$ and the solvent removed under reduced pressure. Purification by flash column chromatography (9:1 heptane/EtOAc) afforded (3R,5R)-5-((tert-butyldimethylsilyl)oxy)-3-methyl-7-phenylheptan-1-ol **4d** as colorless oil (89% yield).

$^1$H NMR spectroscopic analysis of the unpurified reaction mixture indicated **> 20: 1 dr**.

HPLC analysis (Lux-Cellulose1 (Chiralcel OD-H), (n-heptane + 0.1% $^i$PrOH) : / $^i$PrOH = 92:8, 0.70 mL/min, 210 nm) indicated > **99% ee** ($t_{minor}$ = 8.0 min, $t_{major}$ = 6.4 min).

**$^{1}$H NMR** (400 MHz, $CDCl_3$) δ 7.37 – 7.25 (m, 2H), 7.23 – 7.11 (m, 3H), 3.84 – 3.78 (m, 1H), 3.75 – 3.57 (m, 2H), 2.65 (dd, *J* = 10.8, 5.8 Hz, 2H), 1.93 – 1.69 (m, 3H), 1.64 – 1.16 (m, 5H), 0.93 (s, 3H), 0.91 (s, 9H), 0.15 – -0.02 (m, 6H).

**$^{13}$C NMR** (100 MHz, $CDCl_3$) δ 142.8, 128.5 (4C), 125.8, 67.0, 61.3, 44.6, 40.6, 39.9, 31.6, 26.2, 26.1 (3C), 20.2, 18.3, -4.0, -4.3.

**HRMS** (ESI): *m/z* calcd. for $[M+Na]^+$ 359.2377, found 359.2374

**FT-IR** (neat): 3333 (br), 2952, 2928, 2856, 1459, 1377, 1254, 1091, 1056, 1030cm$^{-1}$.

$[\alpha]_D^{20}$= -23.1 (c = 1.17, $CHCl_3$)

**Synthesis of (4R,6R)-4-methyl-8-phenyloctane-1,6-diol (4e)**

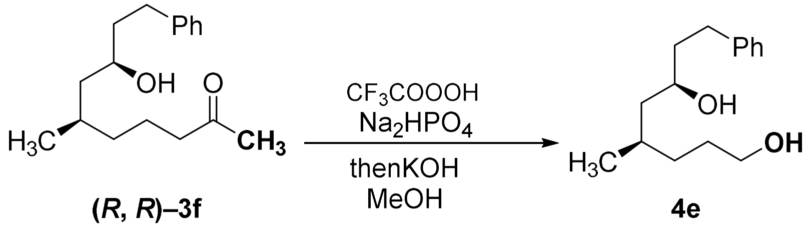


**Procedure: Compound 4e** was prepared with slight modifications according to a reported procedure.[4]

An approximately 1M solution of trifluoroperacetic acid was prepared according to the following procedure: to a 25-mL, round-bottomed flask was added urea hydrogen peroxide (5 mmol) and anhydrous 1, 2-dichloroethane (5.0 mL). The suspension was cooled to 0 °C in an ice/water bath and trifluoroacetic anhydride (5.5 mmol) added dropwise by syringe. The solution was stirred at 0°C for 1 h, then the ice bath removed and stirred at room temperature for 1 h, by which time the white suspension had changed into a biphasic mixture. Stirring was stopped to allow the layers to separate. To achieve more reproducible results, the biphasic mixture was placed in a -20°C freezer for 1 h to freeze trifluoroacetic acid before addition.

To a 25-mL, round-bottomed flask was added **(*R*, *R*)-3f** (0.2 mmol), $HNa_2PO_4$ (1.2 mmol, 6 eq.) and 1, 2-dichloroethane (10 mL). The suspension was stirred vigorously at room temperature, then trifluoroperacetic acid (1M solution in 1, 2-DCE, as prepared above, ~4 eq) was added dropwise by syringe. The solution was stirred for 4 h at 20 °C, by which time TLC analysis had indicated consumption of the starting material. The

reaction was quenched with $Na_2S_2O_3$ (3.2 mmol, 16 eq.) and concentrated in vacuo [**Caution!** Trifluoroperacetic acid, like other organic peroxides, is potentially explosive and should be used with caution. Although no incidents involving this peroxide were encountered during these studies, rotary evaporation was conducted behind a blast shield as a precaution].

The crude residue was redissolved in methanol and potassium hydroxide (3 mmol, 15 eq.) was added. The reaction was refluxed for 3 h, quenched with saturated aqueous $NaHCO_3$, extracted with ether and dried over $MgSO_4$. Purification by flash column chromatography (1:1 heptane/EtOAc) afforded (4*R*,6*R*)-4-methyl-8-phenyloctane-1,6-diol **4e** as off-yellow oil in 95% yield.

$^1$H NMR spectroscopic analysis of the unpurified reaction mixture indicated **> 20: 1 dr**.

HPLC analysis (Lux-Cellulose1( Chiralcel OD-H), (*n*-heptane + 0.1% *i*-PrOH) : / *i*-PrOH = 85:5, 0.70 mL/min, 210 nm) indicated **> 99% ee** ($t_{minor}$ = 13.9 min, $t_{major}$ = 15.3 min).

**$^1$H NMR** (400 MHz, $CDCl_3$) δ 7.40 – 7.30 (m, 2H, H9/10), 7.26–7.2 (m, 3H, H9/10+11), 3.85–3.73 (m, 1H), 3.66 (t, *J* = 6.6 Hz, 2H), 2.92–2.79 (m, 1H), 2.76–2.69 (m, 1H), 2.03 (bs, 2H, OH), 1.89–1.48 (m, 6H), 1.47–1.35 (m, 1H), 1.35–1.17 (m, 2H), 0.95 (d, *J* = 6.6 Hz, 3H).

**$^{13}$C NMR** (100 MHz, $CDCl_3$) δ 142.3, 128.5, 125.9, 69.3, 63.1, 44.9, 40.2, 33.9, 32.2, 30.1, 29.2, 19.5.

**HRMS** (ESI): *m/z* calcd. for $[M+Na]^+$ 259.1669, found 259.1673.

**FT-IR** (neat): 3310 (br), 3026, 2927, 2866, 1495, 1454, 1377, 1114, 1054, 919, 746, 699 $cm^{-1}$.

$[\alpha]_D^{20}$= -5.6 (C = 1.07, $CHCl_3$)

Synthesis of **(5*R*,7*R*)-7-hydroxy-5-methyl-9-phenylnonanoic acid (4f)**

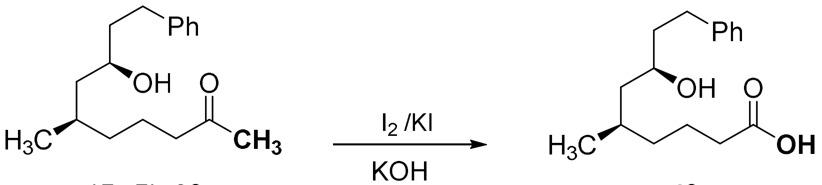


**(*R*, *R*)–3f** **4f**

**Procedure:** **(*R*, *R*)-3f** (0.2 mmol) was dissolved in dioxane (2 mL) at room temperature. This solution was vigorously stirred as a solution of potassium hydroxide (2 mmol, 10 eq.) in $H_2O$ (2.0 mL) and a solution of iodine (0.6 mmol, 3 eq.) and potassium iodide (2.4 mmol, 12 eq.) in $H_2O$ (2.0 mL) were added *via* syringe at similar rates over 10 min. This mixture was stirred for 2 h and the reaction was quenched with 1M aqueous $Na_2SO_3$ (2 mL). The pH of the mixture was adjusted to 14 with 1M aqueous potassium hydroxide, diluted with water (5 mL) and washed with dichloromethane. The pH of the aqueous layer was adjusted to 3 with 1M aqueous $NaHSO_4$ and extracted with ether (2x20 mL). The combined organic layers were dried over $MgSO_4$, filtered and concentrated. Purification by flash column chromatography (2:1 heptane/EtOAc + 1% acetic acid) afforded (5R,7R)-7-hydroxy-5-methyl-9-phenylnonanoic acid **4f** as white solid in 80% yield.

$^1$H NMR spectroscopic analysis of the unpurified reaction mixture indicated **> 20: 1 dr**.

HPLC analysis (Chiralpak IA column, Chirapak IA, n-heptane + 1% ethanol + 5% isopropanol + 0.1% trifluoroacetic acid, 0.70 mL/min, 210 nm) indicated **> 99% ee** ($t_{minor}$ = 35.0 min, $t_{major}$ = 30.1 min).

**$^1$H NMR** (400 MHz, $CDCl_3$) δ 7.30 – 7.21 (m, 2H), 7.20 – 7.08 (m, 3H), 6.13 (bs, 1H, OH), 3.79 – 3.62 (m, 1H), 2.83–2.70 (m, 1H), 2.68–2.60 (m, 1H), 2.29 (t, *J* = 7.4 Hz, 2H), 1.83 – 1.54 (m, 5H), 1.47 (ddd, *J* = 13.7, 9.5, 4.1 Hz), 1.37 – 1.06 (m, 3H), 0.87 (d, *J* = 6.6 Hz, 3H).

**$^{13}$C NMR** (100 MHz, $CDCl_3$) δ 179.4, 142.2, 128.5 (2C), 128.5 (2C), 126.0, 69.4, 44.8, 40.1, 37.2, 34.3, 32.2, 29.1, 22.2, 19.3.

**HRMS** (ESI): *m/z* calcd. for $[M+Na]^+$ 287.1618, found:287.1617.

**FT-IR** (neat): 3430 (br), 3028, 2931, 2877, 1709, 1455, 1412, 1377, 1251, 1175, 1107, 1050, 746, 700 $cm^{-1}$.

$[\alpha]^{20}_{D}$= -6.0 (C = 1.0, $CHCl_3$)

# 4. Synthesis of Stereotriads

**(6*R*,7*S*,8*R*)-8-hydroxy-6,7-dimethyl-10-phenyldecan-2-one (6a)**

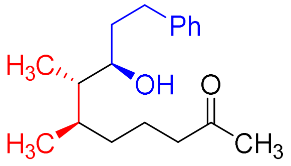


Compound **6a** was prepared according to the **GP2** at r.t. and obtained in 71% yield as colorless oil after column chromatography (2:1 heptane:EtOAc).

$^1$H NMR spectroscopic analysis of the unpurified reaction mixture indicated dr > 15: 1 dr.

HPLC analysis (Chiralcel OD-H 250×4, 6 mm, (n-Heptane+0.1% i-PrOH):i-PrOH = 95:5, 1.00 mL/min, 210 nm) indicated > 99% ee ($t_{minor}$ = 14.1 min, $t_{major}$ = 17.6 min).

**$^1$H NMR** ($CDCl_3$ 400 MHz): δ 7.20-7.25 (m, 2H), 7.11-7.17 (m, 3H), 3.40-3.45 (m, 1H), 2.78-2.85 (m, 1H), 2.58-2.68 (m, 1H), 2.34 (t, *J* = 7.6 Hz, 2H), 2.08 (s, 3H), 1.76-1.88 (m, 2H), 1.40-1.64 (m,5H), 1.27 (m,1H), 1.11-1.19 (m, 2H), 0.73 (d, *J* = 6.8 Hz, 3H), 0.68 (d, *J* = 6.8 Hz, 3H).

**$^{13}$C NMR** ($CDCl_3$ 100 MHz): δ 209.1, 142.4, 128.5, 128.4, 125.8, 73.3, 43.9, 42.5, 36.6, 35.1, 32.2, 32.0, 29.9, 21.7, 14.1, 10.1.

**HRMS** (ESI): *m/z* calcd. for $C_{18}H_{28}NaO_2$ (M + Na)+ 299.1982; found: 299.1979.

**FT-IR** (neat): 3440, 2955, 1710, 1454, 1361, 1276, 1261,1167, 1045, 764, 750, 701 $cm^{-1}$.

[ $\alpha_D^{20}$]= -1.4 (C= 1.0, $CHCl_3$).

**5-((1*R*,2*S*)-2-((*R*)-1-hydroxy-3-phenylpropyl)cyclopentyl)pentan-2-one (6b)**

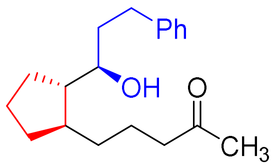


Compound **6b** was prepared according to the **GP2** at r.t. and obtained in 60% yield as colorless oil after column chromatography (2:1 heptane:EtOAc).

$^1$H NMR spectroscopic analysis of the unpurified reaction mixture indicated dr > 15: 1 dr.

HPLC analysis analysis of the Fmoc-derivatized analog (IA column, 0.1% iPrOH + Heptane/hexanes = 92:8, 0.70 mL/min, 254 nm) indicated > 99% ee ($t_{minor}$ = 13.8 min, $t_{major}$ = 12.5 min).

**$^1$H NMR** ($CDCl_3$ 600 MHz): δ 7.19-7.23 (m, 2H), 7.11-7.15 (m, 3H), 3.65-3.41 (m, 1H), 2.77-2.82 (m, 1H), 2.56-2.61 (m, 1H), 2.30-2.40 (m, 2H), 2.06 (s, 3H), 1.74-1.78 (m, 1H),

**$^{13}$C NMR** ($CDCl_3$ 150 MHz): δ 209.5, 142.3, 128.5, 128.4, 125.8, 75.4, 51.7, 43.8, 41.60, 37.3, 36.3, 32.5, 32.3, 29.9, 29.3, 25.1, 22.6.

**HRMS** (ESI): *m/z* calcd. for $C_{19}H_{28}NaO_2$ $(M + Na)^+$ 311.1982; found: 311.1980.

**FT-IR** (neat): 2947, 2863, 1738, 1713, 1450, 1386, 1255, 1008, 968, 746, 700 $cm^{-1}$.

$[\alpha_D^{20}]$= 11.6 (C = 0.25, $CHCl_3$)

**5-((1*R*,2*S*)-2-((*R*)-1-hydroxy-3-phenylpropyl)cyclohexyl)pentan-2-one** (**6c**)

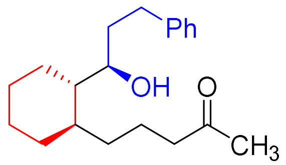


Compound **6c** was prepared according to the **GP2** at r.t. and obtained in 74% yield as colorless oil after column chromatography (2:1 heptane:EtOAc).

$^1$H NMR spectroscopic analysis of the unpurified reaction mixture indicated > **20: 1 dr**.

HPLC analysis (AD-H column, 3% iPrOH/hexanes, 0.80 mL/min, 254 nm) indicated > **99% ee** ($t_{minor}$ = 25.8 min, $t_{major}$ = 15.0 min).

**$^1$H NMR** ($CDCl_3$ 400 MHz): δ 7.28-7.32 (m, 2H7.18-7.25 (m, 3H), 3.84 (m, 1H), 2.89-2.96 (m, 1H), 2.62-2.69 (m, 1H), 2.21-2.35 (m, 2H), 2.12 (s, 3H), 1.87 (d, *J* = 12.4, 1H), 1.56-1.75 (m, 7H),1.33-1.44 (m, 2H), 1.13-1.24 (m, 4H),0.95-1.07 (m, 3H).

**$^{13}$C NMR** ($CDCl_3$ 100 MHz): δ 209.1, 142.4, 128.6, 128.4, 125.8, 70.6, 48.0, 44.0, 38.4, 33.0, 32.8, 32.1, 31.6, 29.9, 26.2, 26.0, 24.8, 20.2.

**HRMS** (ESI): *m/z* calcd. for $C_{20}H_{30}NaO_2$ $(M + Na)^+$ 325.2138; found: 325.2130

**FT-IR** (neat): 3424, 2920, 2853, 1709, 1495, 1451, 1359, 1300, 1163, 1035, 924, 947, 700 $cm^{-1}$.

$[\alpha_D^{20}]$= 69.0 (C = 1.4, $CHCl_3$)

**5-((1*R*,2*S*)-2-((*R*)-1-hydroxy-3-phenylpropyl)cycloheptyl)pentan-2-one** (**6d**)

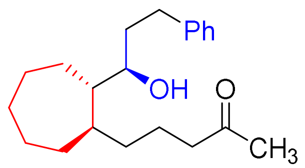


Compound **6d** was prepared according to the **GP2** at r.t. and obtained in 77% yield as colorless oil after column chromatography (2:1 heptane:EtOAc).

$^1$H NMR spectroscopic analysis of the unpurified reaction mixture indicated **> 20: 1 dr**.

HPLC analysis (Chiralpak IC 250×4 column, (n-Heptane+0.1% i-PrOH):EtOH = 8:2, 0.70 mL/min, 210 nm) indicated **> 99% ee** ($t_{minor}$ = 7.4 min, $t_{major}$ = 8.2 min).

**$^1$H NMR** ($CDCl_3$ 400 MHz): δ 7.19-7.23 (m, 2H), 7.08-7.15 (m, 3H), 3.51 (m, 1H), 2.77-2.85 (m, 1H), 2.52-2.60 (m, 1H), 2.18-2.30 (m, 2H), 2.04 (s, 3H), 1.48-1.73 (m, 7H), 1.11-1.45 (m, 13H).

**$^{13}$C NMR** ($CDCl_3$ 150 MHz): δ 209.3, 142.3, 128.5, 128.4, 125.8, 74.1, 51.8, 43.8, 39.2, 38.1, 34.9, 34.2, 32.9, 30.7, 29.9, 29.9, 28.7, 26.4, 23.9, 21.7.

**HRMS** (ESI): *m/z* calcd. for $C_{21}H_{32}NaO_2$ $(M + Na)^+$ 339.2295; found 339.2292.

**FT-IR** (neat): 3423, 2919, 2854, 1709, 1454, 1359, 1276, 1261, 1166, 1032, 750, 700 $cm^{-1}$.

$[\alpha]_D^{20}$ = 37.5 (C = 0.8, $CHCl_3$)

**5-((1*R*,2*S*)-2-((*R*)-1-hydroxy-3-phenylpropyl)cyclooctyl)pentan-2-one** (**6e**)

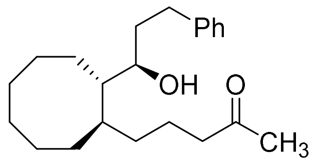


Compound **6e** was prepared according to the **GP2** at r.t. and obtained in 50% yield as colorless oil after column chromatography (2:1 heptane:EtOAc).

$^1$H NMR spectroscopic analysis of the unpurified reaction mixture indicated = **10: 1 dr.**

HPLC analysis (Chiralpak IC 250×4 column, (n-Heptane+0.1% i-PrOH):EtOH = 9:1, 1.00 mL/min, 210 nm) indicated **> 99% ee** ($t_{minor}$ = 6.7 min, $t_{major}$ = 7.8 min).

**$^1$H NMR** ($CDCl_3$ 400 MHz): δ 7.28-7.32 (m, 2H), 7.18-7.24 (m, 3H), 3.67-3.72 (m, 1H), 2.7-2.94 (m, 1H), 2.61-2.68 (m, 1H), 2.25-2.38 (m, 2H), 2.14 (s, 3H), 1.75-1.83 (m, 2H), 1.55-1.72 (m, 5H), 1.18-1.45 (m, 13H)

$^{13}$**C NMR** ($CDCl_3$ 150 MHz): 209.2, 142.3, 128.6, 128.4, 125.8, 73.5, 50.0, 43.9, 37.4, 34.9, 32.9, 32.6, 31.6, 29.9, 26.3, 26.2, 24.7, 24.0, 21.7.

**HRMS (ESI):** m/z calcd. for $C_{22}H_{34}NaO_2$ $(M + Na)^+$ 353.2451; found: 353.2450.

**FT-IR** (neat): 2924, 2856, 1711, 1541, 1276, 1261, 750 $cm^{-1}$.

$[\alpha]_D^{20}$ = 53.6 (C = 0.7, $CHCl_3$)

**<u>5-((1R,2S)-2-((R)-1-hydroxy-2-methylpropyl)cyclohexyl)pentan-2-one</u> (6f)**

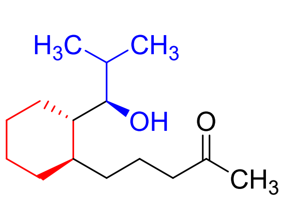


Compound **6f** was prepared according to to the **GP2** at r.t. and obtained in 77% yield as colorless oil after column chromatography (2:1 heptane:EtOAc).

$^1$H NMR spectroscopic analysis of the unpurified reaction mixture indicated **> 20: 1 dr**.

HPLC analysis analysis of the Fmoc-derivatized analog (AD-H column, (0.1% iPrOH + hexanes): iPrOH = 9:1, 1.0 mL/min, 254 nm) indicated **> 99% ee** ($t_{minor}$ = 8.8 min, $t_{major}$ = 7.6 min).

$^1$**H NMR** ($CDCl_3$ 600 MHz): δ 3.55-3.57 (m, 1H), 2.38-2.47 (m, 2H), 2.13 (s, 3H), 1.81-1.87 (m, 1H), 1.61-1.72 (m, 4H), 1.19-1.55 (m, 11H), 0.98 (d, $J$ = 6.6Hz, 3H), 0.84 (d, $J$ = 6.6 Hz, 3H).

$^{13}$**C NMR** ($CDCl_3$ 150 MHz): δ 209.6, 76.5, 44.0, 43.9, 36.0, 32.6, 29.9, 29.5, 28.2, 25.3, 24.0, 23.3, 21.3, 20.9, 15.3.

**HRMS** (ESI): *m/z* calcd. for $C_{15}H_{28}NaO_2$ $(M + Na)^+$ 263.1982; found: 263.1978.

**FT-IR** (neat):3458, 2928, 2858, 1711, 1462, 1364, 1276, 1261, 1166, 990, 750 $cm^{-1}$.

$[\alpha]_D^{20}$ = 8.4 (C = 1.0, $CHCl_3$).

**<u>(*R*)-methyl 10-hydroxy-10-((1*S*,2*R*)-2-(4-oxopentyl)cyclohexyl)decanoate</u> (6g)**

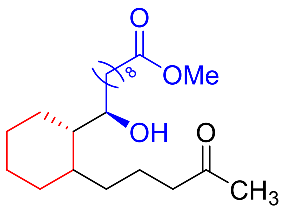


Compound **6g** was prepared according to to the **GP2** at r.t. and obtained in 70% yield as colorless oil after column chromatography (2:1 heptane:EtOAc).

$^1$H NMR spectroscopic analysis of the unpurified reaction

mixture indicated > **20: 1 dr**.

HPLC analysis analysis of the Fmoc-derivatized analog (Chiralpak IA column, 0.1% $^{i}$PrOH/hexanes : $^{i}$PrOH = 92:8, 0.70 mL/min, 210 nm) indicated **> 99% ee** ($t_{minor}$ = 12.6 min, $t_{major}$ = 10.9 min).

**$^{1}$H NMR** ($CDCl_3$ 600 MHz): δ 3.81-3.83 (m, 1H), 3.66 (s, 3H), 2.34-2.44 (m, 2H), 2.29 (t, *J* = 7.8 Hz, 2H), 2.13 (s, 3H), 1.59-1.80 (m, 6H), 1.12-1.54 (m, 20H), 0.99-1.05 (m, 2H).

**$^{13}$C NMR** ($CDCl_3$ 150 MHz): δ 209.2, 174.4, 71.7, 51.5, 47.8, 44.1, 38.2, 34.1, 32.2, 31.4, 31.0, 29.8, 29.7, 29.4, 29.2, 29.1, 26.6, 26.1, 25.9, 24.9, 24.7, 20.2.

**HRMS** (ESI): *m/z* calcd. for $C_{22}H_{40}NaO_4$ $(M + Na)^+$ 391.2819; found: 3982815.

**FT-IR** (neat): 2924, 2854, 1738, 1716, 1439, 1361, 1276, 1261, 1197, 1167, 750 $cm^{-1}$.

$[\alpha]_D^{20}$= 40.3 (C = 1.0, $CHCl_3$)

<u>**5-((1R,2S)-2-((1R,2S)-1-hydroxy-2-methylbutyl)cyclohexyl)pentan-2-one**</u> (**6h**)

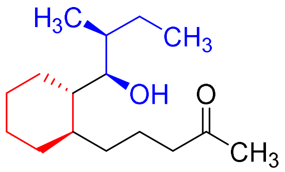


Compound **3g** was prepared according to the to the **GP2** at r.t. and obtained in 71% yield as colorless oil after column chromatography (2:1 heptane:EtOAc).

$^{1}$H NMR spectroscopic analysis of the unpurified reaction mixture indicated > **20: 1 dr**.

**$^{1}$H NMR** ($CDCl_3$ 600 MHz): δ 3.76-3.77 (m, 1H), 2.41-2.49 (m, 2H), 2.16 (s, 3H), 1.27-1.73 (m, 16H), 1.78-1.79 (m, 1H), 0.95 (t, *J* = 7.8 Hz, 3H), 0.84 (d, J = 6.6 Hz, 3H).

**$^{13}$C NMR** ($CDCl_3$ 150 MHz): δ 209.6, 73.9, 44.0, 43.1, 36.0, 35.5, 32.5, 29.9, 27.7, 27.4, 24.3, 23.5, 22.7, 21.5, 12.1, 12.0.

**HRMS** (ESI): *m/z* calcd. for $C_{16}H_{30}NaO_2$ $(M + Na)^+$ 277.2138; found: 277.2134.

**FT-IR** (neat): 3459, 2927, 2858, 1711, 1460, 1361, 1166, 993, 954 $cm^{-1}$.

$[\alpha]_D^{20}$=13.6 (C = 1.0, $CHCl_3$)

**5-((1R,2S)-2-((1S,2S)-1-hydroxy-2-phenylpropyl)cyclohexyl)pentan-2-one** (**6i**)

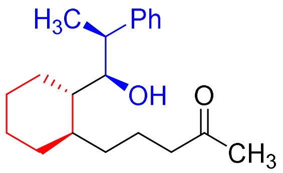


Compound **6i** was prepared according to the **GP2** at r.t. and obtained in 55% yield as colorless oil after column chromatography (2:1 heptane:EtOAc).

$^1$H NMR spectroscopic analysis of the unpurified reaction mixture indicated = 10: 1 dr.

**$^1$H NMR** ($CDCl_3$ 600 MHz): δ 7.30-7.33 (m, 2H), 7.22-7.27 (m, 3H), 2.94-2.99 (m, 1H), 2.36-2.45 (m, 2H), 2.13 (s, 3H), 1.76-1.78 (m, 1H), 1.58-1.67 (m, 4H), 1.44-1.52 (m,2H), 1.25-1.34 (m, 6H), 1.32 (d, *J* = 7.2 Hz, 3H), 1.21-1.22 (m, 1H), 1.09-1.14 (m, 1H).

**$^{13}$C NMR** ($CDCl_3$ 150 MHz): δ 209.4, 143.8, 128.5, 128.4, 126.6, 76.7, 44.7, 44.0, 42.9, 36.5, 32.8, 29.9, 29.6, 26.7, 24.9, 24.2, 21.0, 19.5.

**HRMS** (ESI): *m/z* calcd. for $C_{20}H_{30}NaO_2$ $(M + Na)^+$ 325.2138; found: 325.2126.

**FT-IR** (neat): 3483, 2924, 2855, 1711, 1494, 1361, 1276, 1261, 764, 750, 703 $cm^{-1}$.

$[\alpha]_D^{20}$ = 32.3 (C = 0.8, $CHCl_3$)

**5-((1*R*,2*S*)-2-((1*R*,3*R*)-3-(benzyloxy)-1-hydroxy-5-phenylpentyl)cyclohexyl)pentan-2-one** (**6j**)

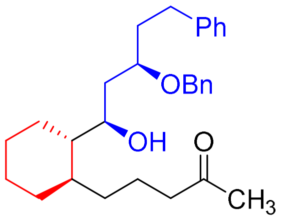


Compound **6j** was prepared according to the **GP2** (50% $BF_3$ $Et_2O$) at r.t. for 30 min and obtained in 60% yield as colorless oil after column chromatography (2:1 heptane:EtOAc).

$^1$H NMR spectroscopic analysis of the unpurified reaction mixture indicated **> 15: 1 dr**.

**$^1$H NMR** ($CDCl_3$ 600 MHz): δ 7.35-7.38 (m, 4H), 7.30-7.33 (m, 3H), 7.20-7.23 (m, 3H), 4.58 (dd, J = 11.4, 34.02 Hz, 2H), 4.22-4.24 (m, 1H), 3.76-3.78 (m, 1H), 2.66-2.77 (m, 2H), 2.28-2.33 (m, 2H), 2.08 (s, 3H), 1.84-1.91 (m, 2H), 1.60-1.79 (m, 6H), 1.51-1.55 (m, 1H), 1.36-1.48 (m,3H), 1.14-1.29 (m,4H), 0.98-1.09 (m, 2H).

**$^{13}$C NMR** ($CDCl_3$ 150 MHz): δ 209.2, 142.0, 138.4,128.4, 128.4, 128.3, 127.9, 127.7, 125.8, 75.4, 67.6, 47.1, 44.1, 38.1, 35.4, 33.9, 32.1, 31.9, 31.9, 29.8, 26.1, 25.9, 24.8, 20.1.

**HRMS** (ESI): *m/z* calcd. for $(M + Na)^+$ 459.2870; found: 459.2865.

**FT-IR** (neat): 3465, 2920, 2853, 1710, 1495, 1452, 1354, 1163, 1605,735, 697 $cm^{-1}$.

[ $\alpha_D^{20}$]= 34.2 (C = 1.0, $CHCl_3$)

**<u>1-chloro-5-((1*S*,2*R*)-2-((*S*)-cyclohexyl(hydroxy)methyl)cyclohexyl)pentan-2-one</u>** (**6k**)

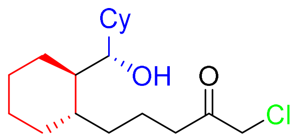


Compound **6k** was prepared according to the **GP2** at r.t. and obtained in 40% yield as colorless oil after column chromatography (2:1 heptane:EtOAc).

$^1$H NMR spectroscopic analysis of the unpurified reaction mixture indicated dr > 20:1 dr. The compound was protectd with Fmoc for HPLC analysis (Chiralcel OD-H 250×4 column, (n-Heptane+0.1% i-PrOH) : i-PrOH = 95:5, 1 mL/min, 210 nm) indicated > 99% ee ($t_{minor}$ = 9.9 min, $t_{major}$ = 9.0 min).

**$^1$H NMR** ($CDCl_3$ 600 MHz): δ 4.08 (s, 2H), 3.50-3.51 (m, 1H), 2.56-2.59 (m, 2H), 1.74-1.76 (m, 2H), 1.50-1.62 (m, 3H), 1.44-1.49 (m, 3H), 1.34-1.41 (m, 2H), 1.24-1.30 (m, 6H), 1.11-1.22 (m, 3H), 1.00-1.07 (m, 1 H).

**$^{13}$C NMR** ($CDCl_3$ 150 MHz): δ 202.9, 76.4, 48.3, 43.4, 40.0, 39.9, 36.0, 32.5, 31.1, 28.3, 26.6, 26.5, 26.2, 26.0, 25.6, 24.1, 23.4, 21.1.

**HRMS** (ESI): *m/z* calcd. for $C_{18}H_{31}ClNaO$ $(M + Na)^+$ 337.1905; found: 337.1893.

**FT-IR** (neat): 3377, 2926, 1710, 1657, 1434, 1275, 1261, 1065,750, 701 $cm^{-1}$.

[ $\alpha_D^{20}$]= -17.7 (C = 1.0, $CHCl_3$)

After X-ray analysis, we reduced **6k** to compare the optical rotation with **6l**.

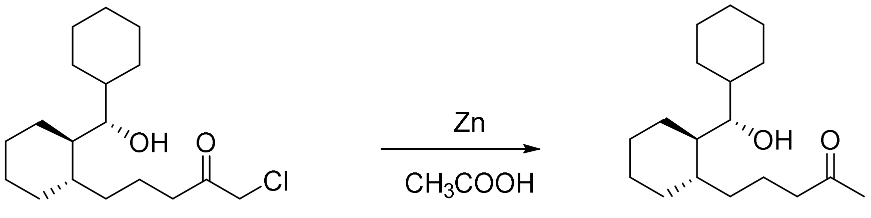


***ent-6l***

Yield > 95%, white solid.

[ $\alpha_D^{20}$]= -16.7 (C = 1.0, $CHCl_3$)

**5-((1*R*,2*S*)-2-((*R*)-cyclohexyl(hydroxy)methyl)cyclohexyl)pentan-2-one** (6l)

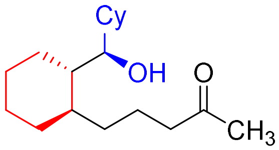


Compound **4f** was prepared according to the **GP2** at r.t. and obtained in 70% yield as colorless oil after column chromatography (2:1 heptane:EtOAc).

$^1$H NMR spectroscopic analysis of the unpurified reaction mixture indicated > **20:1 dr**.

The compound was protectd with Fmoc for HPLC analysis (Chiralcel OD-H 250×4 column, (n-Heptane+0.1% i-PrOH) : i-PrOH = 95:5, 1 mL/min, 210 nm) indicated > **99% ee** ($t_{minor}$ = 9.1 min, $t_{major}$ = 10.0 min).

**$^1$H NMR** ($CDCl_3$ 600 MHz): δ 3.50-3.52 (m, 1H), 2.36-2.44 (m, 2H), 2.12 (s, 3H), 1.00-1.76 (m, 25H).

**$^{13}$C NMR** ($CDCl_3$ 150 MHz): δ 209.5, 76.3, 44.0, 43.5, 40.0, 36.0, 32.6, 31.1, 29.9, 28.4, 26.7, 25.5, 26.2, 26.1, 25.5, 24.2, 23.4, 21.3.

**HRMS** (ESI): *m/z* calcd. for $C_{18}H_{32}NaO_2$ $(M + Na)^+$ 303.2295; found: 303.2293.

**FT-IR** (neat): 3454, 2902, 2852, 1711, 1450, 1361, 1262, 1163, 984, 750, 699 $cm^{-1}$.

$[\alpha]_D^{20}$= 16.4 (C = 1.0, $CHCl_3$)

# 5. Applications

**Synthesis of 4-(((2*S*,4*R*,6*S*)-6-(benzyloxy)-4-hydroxy-8-phenyloctan-2-yl)dimethyl silyl)butan-2-one (8)**

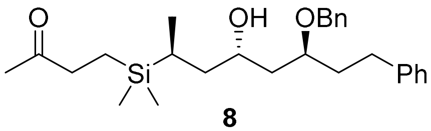

**8**

**Procedure:** Alcohol (0.2 mmol) was diluted with 2 mL dichloromethane. Then aldehyde (0.24 mmol, 1.2 eq.) and $BF_3$·EtO were successively added and the reaction was stirred at r.t. for 2 h. Water (0.2 mL) was added and the reaction was allowed to cool to room temperature with stirring. The reaction mixture was filtered over silica, eluted with dichloromethane and the solvent was removed under reduced pressure. Purification by flash column chromatography afforded the desired pure product **7** in 50% yield.

$^1$H NMR spectroscopic analysis of the unpurified reaction mixture indicated **>20:1 dr**.

**$^1$H NMR** ($CDCl_3$ 600 MHz): δ 7.28-7.37 (m, 7H), 7.18-7.22 (m, 3H), 4.56 (dd, $J$ = 1.2, 2.4 Hz, 2H), 4.02-4.05 (m, 1H), 3.73-3.77 (m, 1H), 2.65-2.75 (m, 2H), 2.56 (*b* s, 1H), 2.38-2.41 (m, 2H), 2.14 (s, 3H), 2.02-2.08 (m, 1H), 1.84-1.90 (m, 1H), 1.72-1.77 (m, 1H), 1.59-1.63 (m, 1H), 1.49-1.53 (m, 1H), 1.08-1.13 (m, 1H), 0.96-1.01 (m, 1H), 0.94 (d, $J$ = 6.0 Hz, 3H), 0.76-0.79 (m, 2H), 0.04 (s, 6H).

**$^{13}$C NMR** ($CDCl_3$ 150 MHz): δ 210.0, 138.2, 128.4, 128.4, 128.3, 127.9, 127.8, 125.8, 76.3, 65.2, 40.8, 39.0, 38.1, 35.4, 31.6, 29.2, 13.6, 13.2, 6.9, -5.5, -5.6.

**HRMS** (ESI): m/z calcd. for $C_{27}H_{40}NaO_3Si$ $(M + Na)^+$ 463.2639; found: 463.2634.

**FT-IR** (neat): 2947, 2865, 1715, 1454, 1412, 1356, 1195, 1065, 1029, 839, 700 $cm^{-1}$.

$[\alpha]_D^{20}$= -2.3 (C = 1.0, $CHCl_3$)

**Synthesis of (2*S*,4*S*,6*S*)-6-(benzyloxy)-8-phenyloctane-2,4-diol**

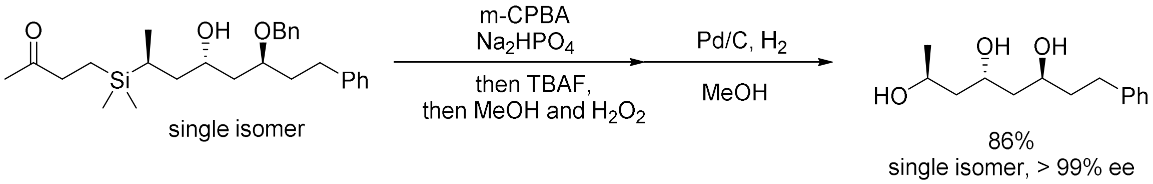


**Procedure:** To a 25-mL, round-bottomed flask was added **8** (1.0 mmol), $Na_2HPO_4$ (2.0 mmol, 2.0 eq.) and dichloromethane (10 mL). The suspension was stirred vigorously at room temperature, then *m*-CPBA solid was slowly added in several portions. The solution was stirred at r.t. for 12. The reaction was quenched with $Na_2S_2O_3$ (3.2 mmol, 16 eq.) and concentrated in vacuo. The crude residue was redissolved in THF (5 mL) and 3 mL TBAF was added and reflex for 1h. Then 37% $H_2O_2$ (64 equiv.) was added at r.t., which further stirred for 8 h at the same temperature. The reaction was quenched with $Na_2S_2O_3$ (3.2 mmol, 16 eq.) Extracted with ether and dried over $MgSO_4$, then the solvent was removed to afford crude product. The crude product was directly dissolved in MeOH, 10% Pd/C were added and the reaction was stirred under $H_2$ atmosphere for 2 h. After that, Pd/C was filtered off and the solvent was removed. Purification by flash column chromatography (1:1 heptane/EtOAc) afforded **8** as colorless oil in 86% yield.

$^1$H NMR spectroscopic analysis of the unpurified reaction mixture indicated **>20:1** dr.

**$^1$H NMR** ($CDCl_3$ 600 MHz): δ 7.26-7.38 (m, 2H), 7.14-7.26 (m, 3H), 4.20-4.33 (m, 1H), 4.14-4.15 (m, 1H), 4.10-4.12 (m, 1H), 4.01-4.02 (m, 1H), 3.05 (*br* s, 1H), 3.02 (*br* s, 1H), 2.80-2.85 (m, 1H), 2.67-2.72 (m, 1H), 1.86-1.93 (m, 1H), 1.77-1.80 (m, 1H), 1.64-1.80 (m, 4H), 1.24 (d, J = 6.6 Hz, 3H).

**$^{13}$C NMR** ($CDCl_3$ 150 MHz): δ 141.9, 128.4, 128.4, 125.8, 70.8, 69.4, 68.7, 44.2, 42.6, 39.1, 32.1, 24.4.

**HRMS** (ESI): *m/z* calcd. for $C_{14}H_{22}NaO_3$ (M + Na)+ 261.1461; found: 261.1461.

**FT-IR** (neat): 3331, 2931, 1454, 1375, 1323, 1276, 1086,760, 700 cm$^{-1}$.

$[\alpha]_D^{20}$= 4.3 (C = 1.0, $CDCl_3$)

**Synthesis of (6*R*,8*S*,10*S*)-10-(benzyloxy)-8-methoxy-6-methyltridecan-2-one (10)**

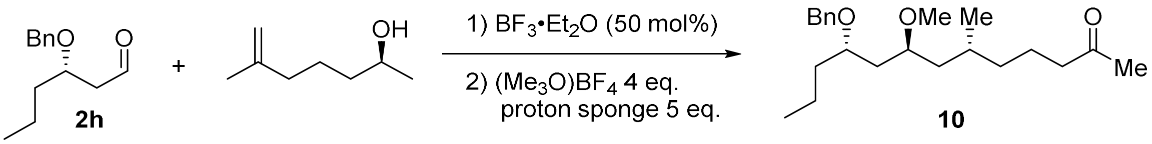

Alcohol (1 mmol) was diluted with 10 mL dichloromethane. Aldehyde (1.2 mmol, 1.2 eq.) and $BF_3 \cdot Et_2O$ (50%) were successively added and the reaction was stirred at r.t. for 2 h. Water (0.2 mL) was added and the reaction was allowed to cool to room temperature with stirring. The reaction mixture was filtered over silica, eluted with dichloromethane and the solvent was removed under reduced pressure. The crude reaction mixture was dissolved in 5 mL dichloromethane and cooled to 0° C. Proton sponge (2.5 mmol, 5 eq.) and trimethyloxonium tetrafluoroborate (2 mmol, 4 eq.) were added successively and stirred at 0° C for 3h. The reaction was quenched with saturated aqueous $Na_2CO_3$ and extracted with ether. The combined organic layers were dried over MgSO4, filtered and concentrated. Purification by column chromatography (9:1 heptane/EtOAc) afforded the product **9** as colorless oil (71% yield).

$^1$H NMR spectroscopic analysis of the unpurified reaction mixture indicated **>20:1 dr**.

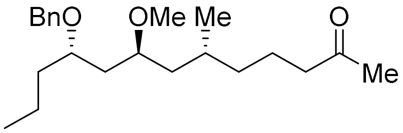

**$^1$H NMR** (400 MHz, $CDCl_3$) δ 7.37 – 7.30 (m, 4H), 7.29 – 7.23 (m, 1H), 4.58 (d, *J* = 11.4 Hz, 1H), 4.46 (d, *J* = 11.5 Hz, 1H), 3.69 – 3.50 (m, 1H), 3.49 – 3.36 (m, 1H), 3.26 (s, 3H), 2.45 – 2.27 (m, 2H), 2.11 (s, 3H), 1.69 – 1.33 (m, 9H), 1.33 – 1.03 (m, 4H), 0.95 – 0.89 (m, 6H).

**$^{13}$C NMR** (101 MHz, $CDCl_3$) δ 209.2, 139.2, 128.5 (2C), 127.9 (2C), 127.6, 76.1, 76.1, 71.2, 56.5, 44.1, 42.1, 40.5, 37.0, 36.7, 30.0, 29.4, 21.3, 20.0, 18.5, 14.5.

**HRMS** (ESI): *m/z* calcd. for $[M+Na]^+$ 371.2557, found 371.2557

**FT-IR** (neat): 2953, 2927, 2871, 1715, 1455, 1360, 1186, 1161, 1091, 1068, 1028, 735, 698 $cm^{-1}$.

$[\alpha_D^{20}]$= 27.2 (C = 1, $CHCl_3$)

**Synthesis of (6*R*,8*S*,10*S*,*E*)-10-(benzyloxy)-8-methoxy-6-methyltridec-3-en-2-one**

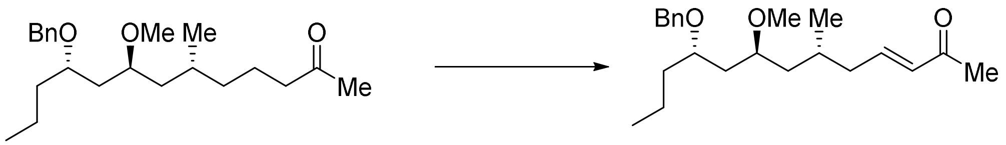


**Procedure**: To a solution of **10** (0.15 mmol) in 0.5 mL diethyl ether was added lutidine (0.45 mmol, 3 eq.) at room temperature. After stirring for 10 minutes, *tert*-butyldimethylsilyl trifluoromethanesulfonate (0.3 mmol, 2 eq.) was added dropwise and the reaction mixture was stirred for 13 hours. The reaction was quenched with saturated aqueous ammonium chloride (3 mL), extracted with ether and dried over $Na_2SO_4$. The solvent was removed under reduced pressure giving the product as 2:1 mixture of diastereoisomers which was used directly for the next step.

Previously obtained silyl enol ether and $Pd(OAc)_2$ (0.015 mmol, 10 mol%) were dissolved in 1.5 mL DMSO, frozen in a bath of liquid nitrogen, evacuated and backfilled with pure oxygen using a balloon. The Schlenk tube was directly taken out of the nitrogen bath, warmed to room temperature and heated at 80° C for 13 h. Upon completion, the reaction mixture was diluted with water and extracted with dichloromethane three times. The combined organic phases were dried over $Na_2SO_4$ and the solvent removed under reduced pressure. The crude product was purified using flash column chromatography (20:1-9:1 heptane/EtOAc) to give a colorless oil (35% yield over 2 steps).

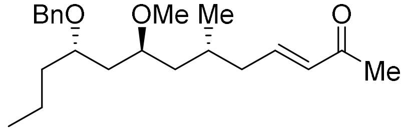


**$^1$H NMR** (400 MHz, $CDCl_3$) δ 7.37 – 7.24 (m, 5H), 6.75 (dt, *J* = 15.8, 7.4 Hz, 1H), 6.06 (dt, *J* = 15.9, 1.3 Hz, 1H), 4.59 (d, *J* = 11.4 Hz, 1H), 4.45 (d, *J* = 11.4 Hz, 1H), 3.62 – 3.55 (m, 1H), 3.48-3.42 (m, 1H) 3.26 (s, 3H), 2.35 – 2.14 (m, 4H), 2.10 – 2.03 (m, 1H), 1.86 – 1.79 (m, 1H), 1.70 – 1.09 (m, 8H), 1.00 – 0.86 (m, 6H).

**$^{13}$C NMR** (101 MHz, $CDCl_3$) δ 198.6, 147.0, 139.1, 132.9, 128.5 (2C), 128.0 (2C), 127.7, 76.1, 76.0, 71.2, 56.7, 41.9, 40.5, 40.4, 36.6, 29.3, 27.0, 20.1, 18.4, 14.5.

**HRMS** (ESI): *m/z* calcd. for $[M+Na]^+$ 369.2400, found 369.2401.

**FT-IR** (neat): 2958, 2921, 1699, 1673, 1632, 1457, 1254, 1095, 1069 $cm^{-1}$.

$[\alpha_D^{20}]$= 24.7 (C = 1, $CHCl_3$)

**Synthesis of (3*S*,5*S*,7*S*)-7-(benzyloxy)-5-methoxy-3-methyldecanal (11)**

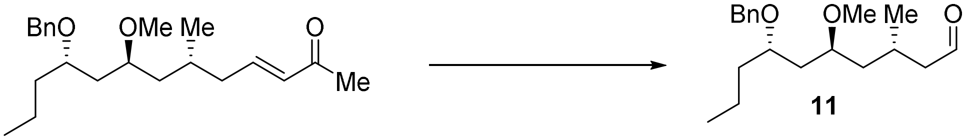


**Procedure:** Unsaturated ketone (0.023 mmol) was dissolved in 1 mL dichloromethane and cooled to -78° C. Ozone was bubbled through the solution until a blue color was observed (ca. 5 minutes). The ozone generator was turned off and oxygen was bubbled through until the solution became colorless. The reaction was quenched with dimethyl sulfide (0.05 mL), slowly warmed up and stirred at room temperature for 5 h. The solvent and excess DMS were removed using a high vacuum pump with a liquid nitrogen cooling trap. The remaining yellow oil was dissolved in 3 mL diethyl ether, 3 mL saturated aqueous $Na_2CO_3$ was added and the biphasic mixture was vigurously stirred at room temperature for 3 h. The mixture was extracted with ether, dried over $Na_2SO_4$ and the solvent removed under reduced pressure. Purification by flash column chromatography (20:1 – 9:1 heptane/EtOAc) afforded the product as colorless oil (85% yield).

**$^1$H NMR** (700 MHz, $CDCl_3$) δ 9.72 (s, 1H), 7.37 – 7.22 (m, 5H), 4.58 (d, *J* = 11.4 Hz, 1H), 4.44 (d, *J* = 11.4 Hz, 1H), 3.58 (td, *J* = 9.1, 5.6 Hz, 1H), 3.51 – 3.38 (m, 1H), 3.26 (s, 3H), 2.40 (dd, *J* = 19.1, 8.5 Hz, 1H), 2.24 (dd, *J* = 10.7, 2.5 Hz, 2H), 1.70 – 1.48 (m, 5H), 1.43 – 1.36 (m, 2H), 1.32 – 1.36 (m, 1H), 0.99 (d, *J* = 6.2 Hz, 3H), 0.93 (t, *J* = 7.3 Hz, 3H).

**$^{13}$C NMR** (176 MHz, $CDCl_3$) δ 202.9, 139.0, 128.5 (2C), 128.0 (2C), 127.7, 76.0, 76.0, 71.2, 56.5, 51.5, 41.9, 40.2, 36.6, 25.2, 20.4, 18.4, 14.5.

**FT-IR** (neat): 2957, 2929, 2873, 1724, 1456, 1379, 1092, 1028, 736, 698

## Comparison of $^{1}$H NMR data with reported values[5]

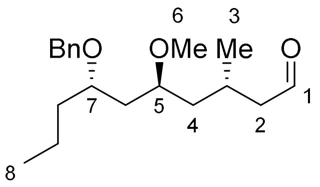


| **$^{1}$H NMR** (500 MHz, $CDCl_3$) (lit) ppm | **$^{1}$H NMR** (700 MHz, $CDCl_3$) (**11**) ppm |
|---|---|
| 0.93 ppm (t, $J$ = 7.2 Hz, 3 H) | 0.93 (t, $J$ = 7.3 Hz, 3H) |
| 0.99 (d, $J$ = 6.0 Hz, 3 H) | 0.99 (d, $J$ = 6.2 Hz, 3H) |
| 1.32–1.26 (m, 1 H) | 1.32 – 1.36 (m, 1H) |
| 1.43–1.37 (m, 2 H) | 1.43 – 1.36 (m, 2H) |
| 1.67–1.46 (m, 5 H) | 1.70 – 1.48 (m, 5H) |
| 2.27–2.20 (m, 2 H) | 2.24 (dd, $J$ = 10.7, 2.5 Hz, 2H) |
| 2.43–2.37 (m, 1 H) | 2.40 (dd, $J$ = 19.1, 8.5 Hz, 1H) |
| 3.26 (s, 3 H) | 3.26 (s, 3H) |
| 3.47–3.42 (m, 1 H) | 3.51–3.38 (m, 1H) |
| 3.60–3.55 (m, 1 H) | 3.58 (td, $J$ = 9.1, 5.6 Hz, 1H) |
| 4.44 (ABq, $J_{AB}$ = 11.2 Hz, 2H) | 4.44 (d, $J$ = 11.4 Hz, 1H) |
| 4.58 and 4.44 (ABq, $J_{AB}$ = 11.2 Hz, 2H) | 4.58 (d, $J$ = 11.4 Hz, 1H) |
| 7.34–7.26 (m, 5 H) | 7.37–7.22 (m, 5H) |
| 9.72 (t, $J$ = 2.0 Hz, 1 H) | 9.72 (s, 1H) |

| $^{13}$**C NMR** (126 MHz, $CDCl_3$) (lit) ppm | $^{13}$**C NMR** (176 MHz, $CDCl_3$) (**11**) ppm |
|---|---|
| 14.6 | 14.5 |
| 18.5 | 18.4 |
| 20.5 | 20.4 |
| 25.3 | 25.2 |
| 36.7 | 36.6 |
| 40.3 | 40.2 |
| 42.0 | 41.9 |
| 51.6 | 51.5 |
| 56.6 | 56.5 |
| 71.3 | 71.2 |
| 76.1 | 76.0 |
| 76.1 | 76.0 |
| 127.7 | 127.7 |
| 128.1 | 128.0 |
| 128.6 | 128.5 |
| 139.1 | 139.0 |
| 202.8 | 202.9 |

**Literature:** $[a]_D^{27}$ **+22.4** (C = **1.00, $CHCl_3$**)

**Our synthesis of 14:** $[\alpha]_D^{20}$ **= +6.0 (C = 0.67, $CHCl_3$)**

# 6. Assignment of Absolute Configuration

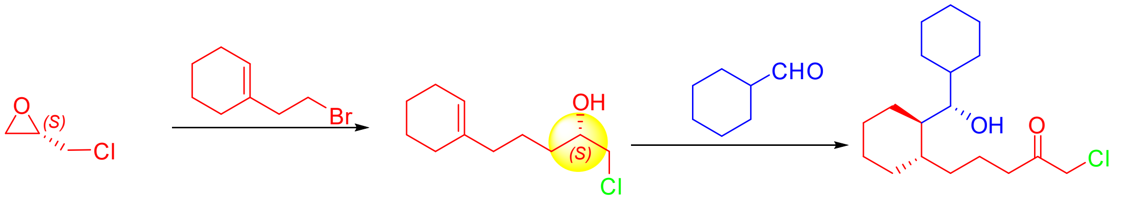


**6k**

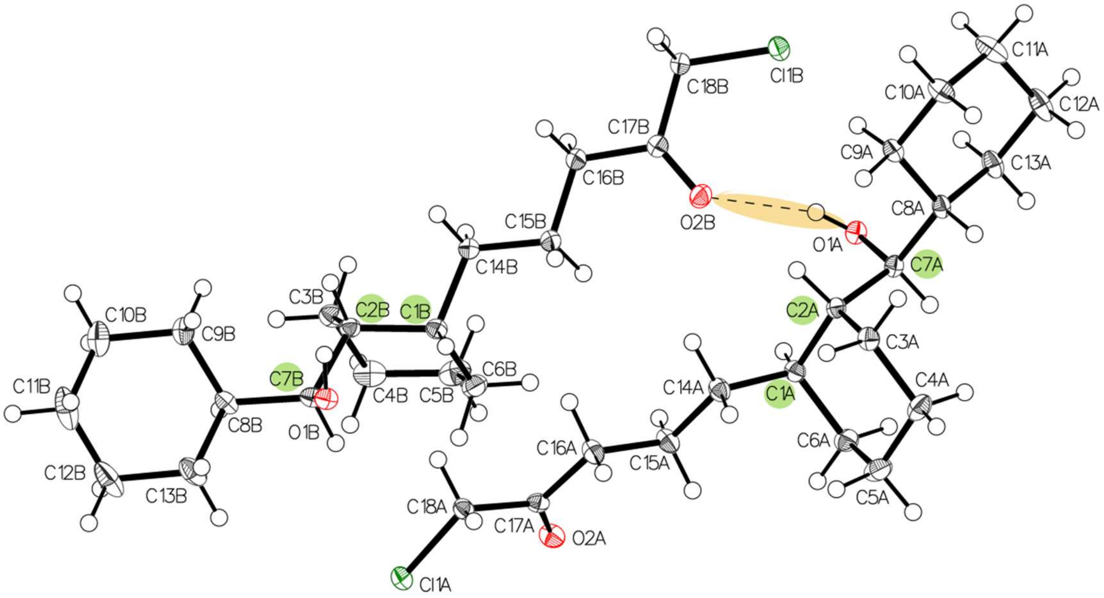


**Figure S-1** Asymmetric Unit of **6k** drawn with 50% displacement ellipsoid. Bond precision: C-C = 0.0022 Å. One intermolecular hydrogen bond (orange shaded) is part of the Asymmetric Unit (O1A – O2B: H-A=2.07 Å, D-A=2.8478(15) Å, Angle154.5°). Chiral centres (green shaded) are proofed with Flack=-0.030(8) and HOOFT=-0.032(8) Parameter. S: C1A, C1B, C7A, C7B; R: C2A,C2B.

Dissolving the sample in pentane, then slowly evaporating the solvent to obtain the crystal is suitable for X-Ray analysis. The *X*-ray intensity data were measured on Bruker D8 Venture and on a Bruker X8 Apex2 diffractometer equipped each with multilayer monochromator, Mo K/a INCOATEC micro focus sealed tube and Oxford Cryostream respectively Kryoflex cooling systems. The structures were solved by *direct methods* and refined by *full-matrix least-squares techniques*. Non-hydrogen atoms were refined with *anisotropic displacement parameters*. Hydrogen atoms were inserted at calculated positions and refined with riding model and as rotating groups. The following software was used: *Bruker SAINT software package*[i] using a narrow-frame algorithm for frame integration, *SADABS*[ii] for absorption correction, *OLEX2*[iii] for structure solution, refinement, molecular diagrams and graphical user-interface, *Shelxle*[iv] for refinement and

graphical user-interface *SHELXS-2013* [v] for structure solution, *SHELXL-2013* [vi] for refinement, *Platon*[vii] for symmetry check. Experimental data and CCDC-Codes can be found in Table S-3. Crystal data, data collection parameters, and structure refinement details are given in Tables S-4 to S-5. Crystal structures visualized in Figure S-1.

**Table S-3** Experimental parameter and CCDC-Code.

| Sample | Machine | Source | Temp. | Detector Distance | Time/ Frame | #Frames | Frame width | CCDC |
|---|---|---|---|---|---|---|---|---|
| | Bruker | | [K] | [mm] | [s] | | [°] | |
| JiLiCy2 | D8 | Mo | 100 | 40 | 15 | 2445 | 0.5 | 1813941 |

**Table S-41** Sample and crystal data of [**JiLiCy2**].

| | | | | |
|---|---|---|---|---|
| **Chemical formula** | C18H31ClO2 | **Crystal system** | monoclinic | |
| **Formula weight [g/mol]** | 314.88 | **Space group** | *C2* | |
| **Temperature [K]** | 100 | **Z** | 8 | |
| **Measurement method** | \f and \w scans | **Volume [Å$^3$]** | 3514.3(4) | |
| **Radiation (Wavelength [Å])** | MoKα (λ = 0.71073) | **Unit cell dimensions [Å] and [°]** | 30.5188(19) | 90 |
| **Crystal size / [mm$^3$]** | 0.99 × 0.199 × 0.126 | | 6.3177(4) | 101.588(2) |
| **Crystal habit** | clear colourless block | | 18.6060(12) | 90 |
| **Density (calculated) / [g/cm$^3$]** | 1.19 | **Absorption coefficient / [mm$^{-1}$]** | 0.221 | |
| **Abs. correction Tmin** | 0.6932 | **Abs. correction Tmax** | 0.746 | |
| **Abs. correction type** | multiscan | **F(000) [e$^-$]** | 1376 | |

**Table S-5** Data collection and structure refinement of [**JiLiCy2**].

| | | | | |
|---|---|---|---|---|
| **Index ranges** | -42 ≤ h ≤ 42, -8 ≤ k ≤ 8, -26 ≤ l ≤ 26 | **Theta range for data collection [°]** | 4.744 to 60.142 | |
| **Reflections number** | 77713 | **Data / restraints / parameters** | 10283/1/381 | |
| **Refinement method** | Least squares | **Final R indices** | all data | R1 = 0.0411, wR2 = 0.0753 |
| **Function minimized** | $\Sigma$ w($F_o^2$ - $F_c^2$)$^2$ | | I>2σ(I) | R1 = 0.0292, wR2 = 0.0691 |
| **Goodness-of-fit on F$^2$** | 1.031 | **Weighting scheme** | w=1/[$\sigma^2$($F_o^2$)+(0.0411P)$^2$+0.7599P] | |
| **Largest diff. peak and hole [e Å$^{-3}$]** | 0.27/-0.23 | | where P=($F_o^2$+2$F_c^2$)/3 | |

# 7. References


1. Souris, C., Misale, A., Chen, Y., Luparia, M. & Maulide, N. *Org. Lett.* **17**, 4486–4489 (2015)
2. Likhite, N. *et al. Org. Proc. Res. Dev.* **20**, 977–981 (2016)
3. Merschaert, A. *et al. Org. Proc. Res. Dev*. **10**, 776–783 (2006)
4. K. V. Chuang, C. Xu, S. E. Reisman *Science* **2016**, *353*, 912-915
5. S. K. Woo, M. S. Kwon, E. Lee, *Angew. Chem. Int. Ed.* **2008**, *47*, 3242–3244; *Angew. Chem.* **2008**, *120*, 3286–3288

# 8. NMR- and HPLC Spectra

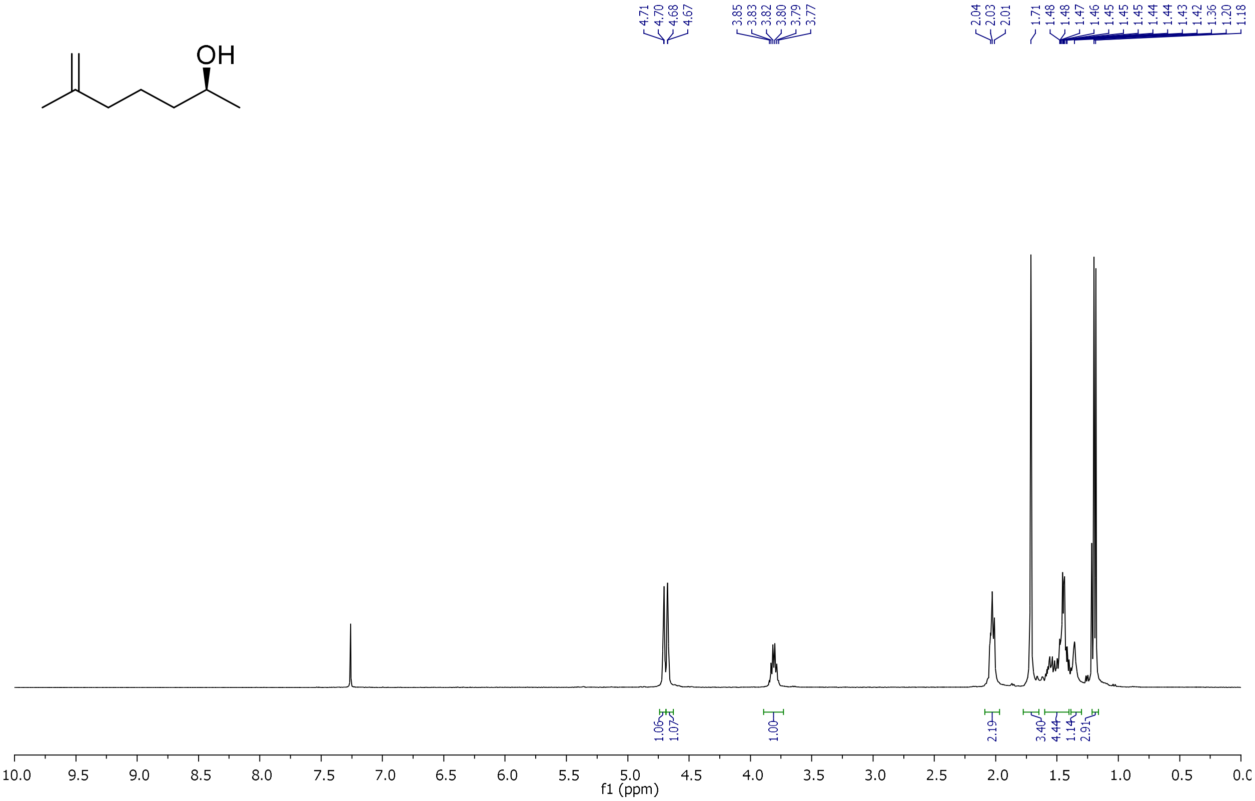


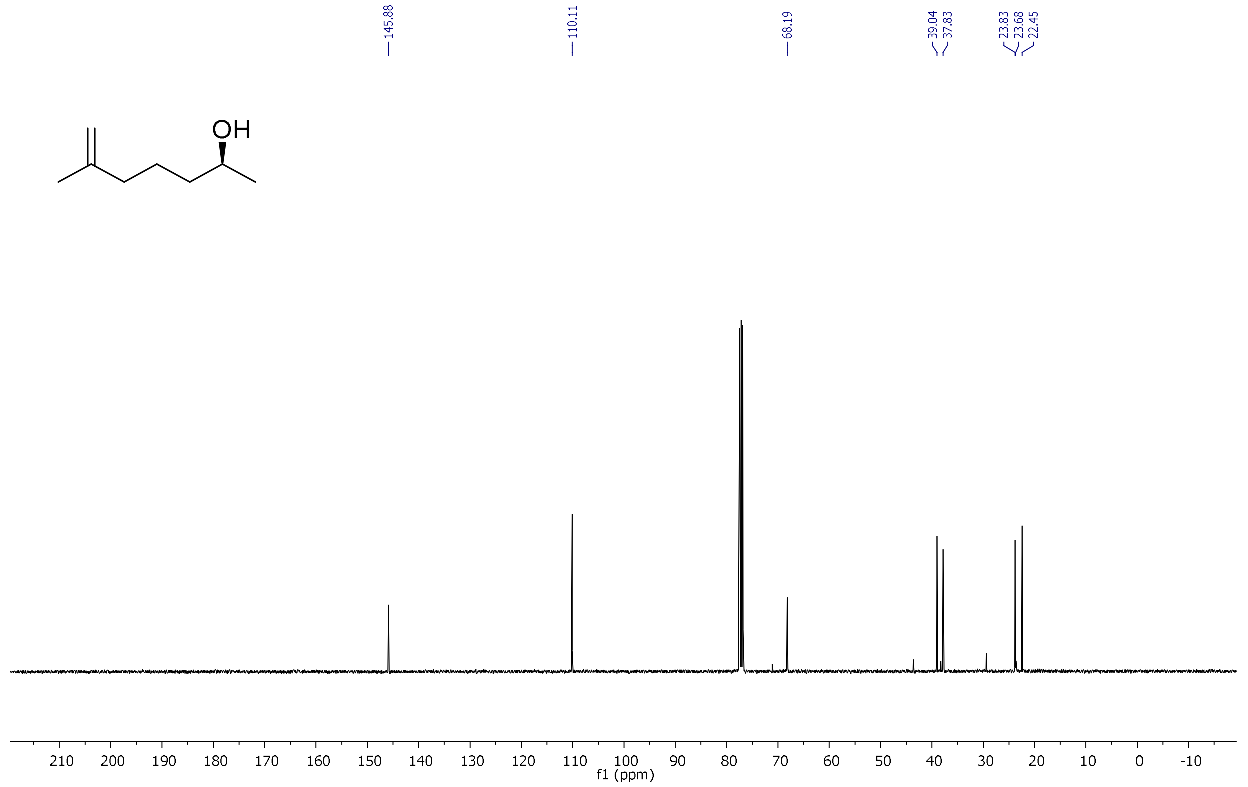

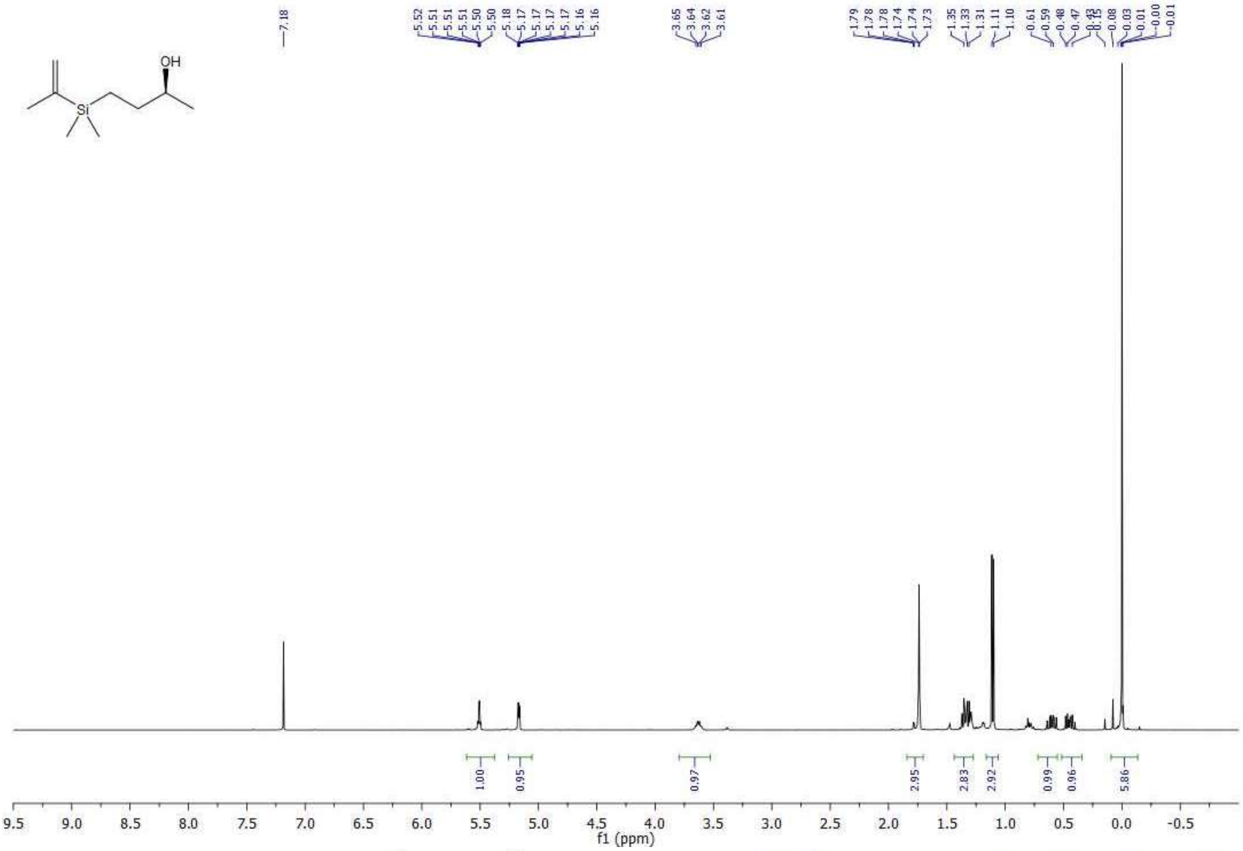
OH
Si
f1 (ppm)

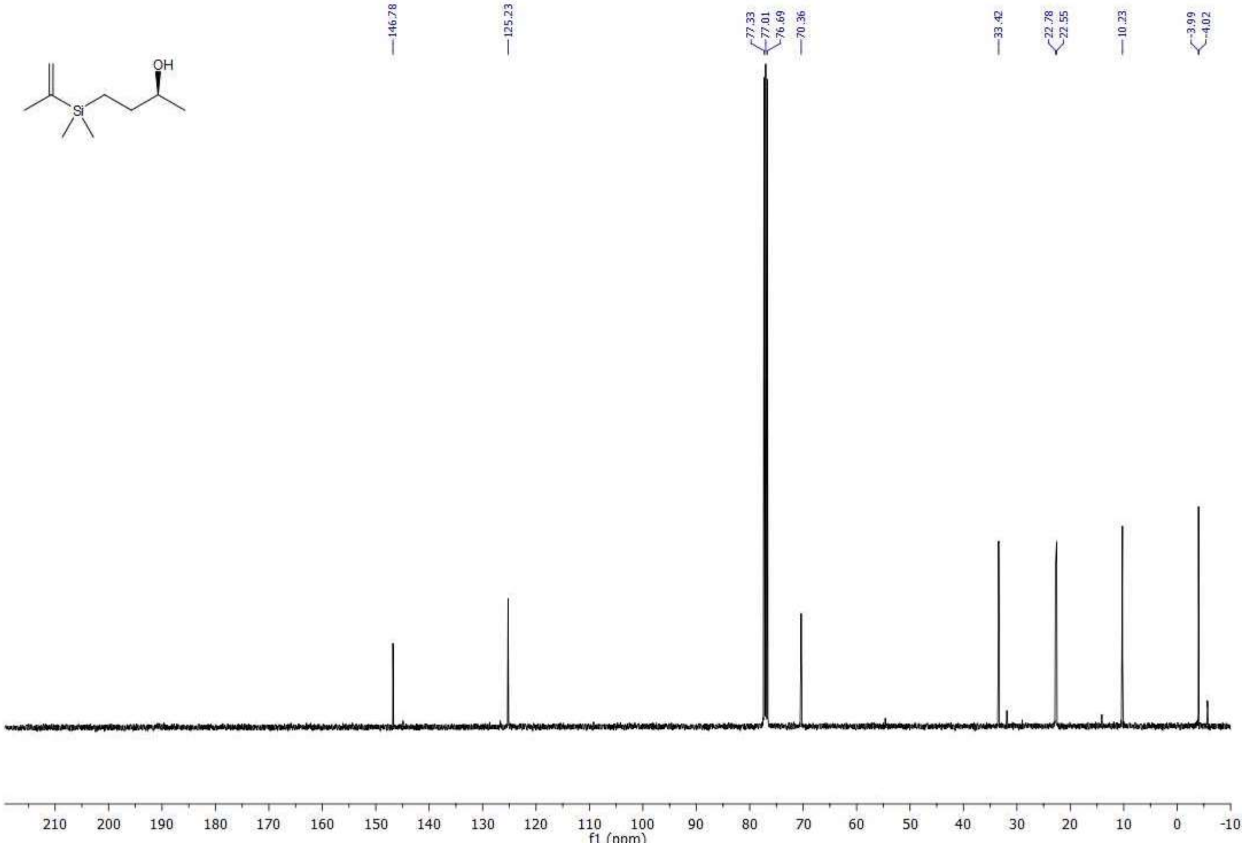
OH
Si
f1 (ppm)

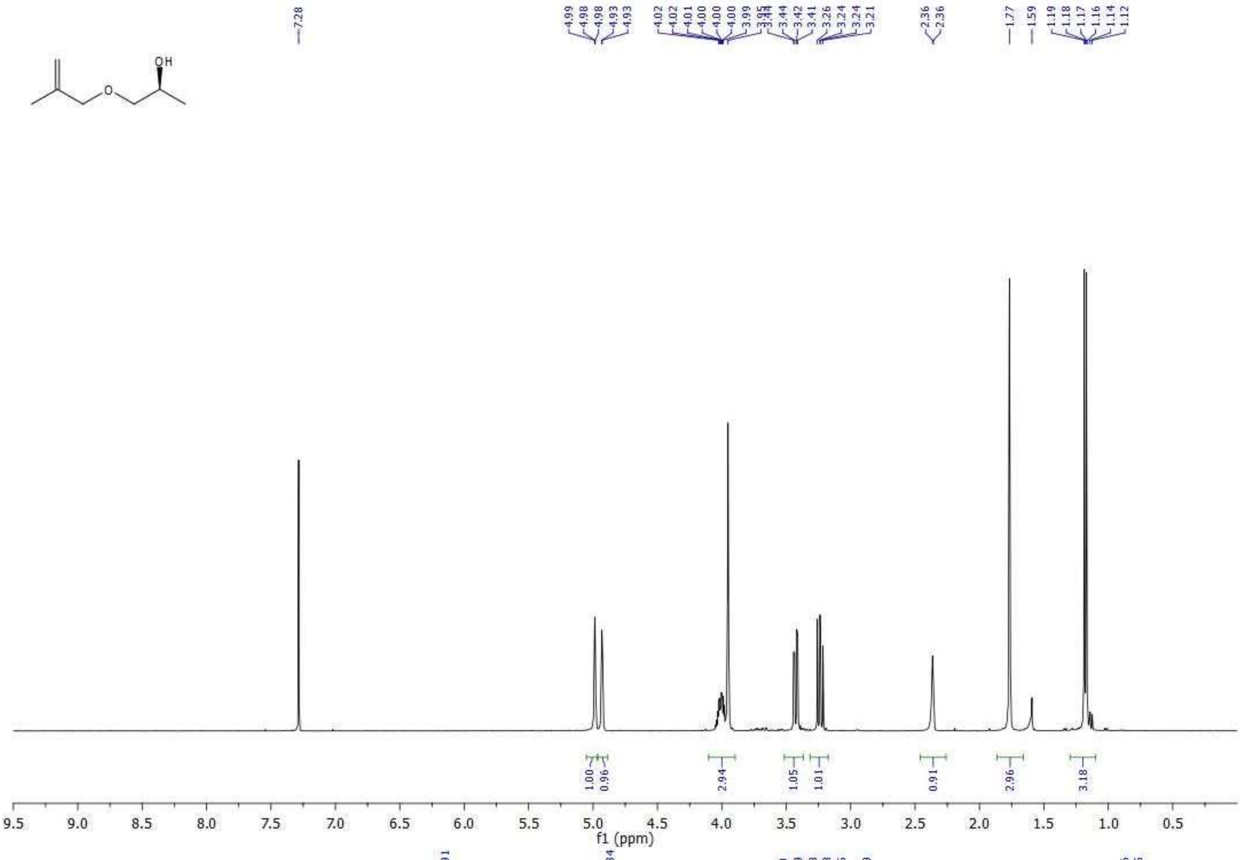
OH
7.28
4.99
4.98
4.98
4.93
4.93
4.02
4.02
4.01
4.00
4.00
4.00
3.99
3.95
3.44
3.42
3.41
3.26
3.24
3.21
2.36
2.36
1.77
1.59
1.19
1.18
1.17
1.16
1.14
1.12
1.00
0.96
2.94
1.05
1.01
0.91
2.96
3.18
f1 (ppm)

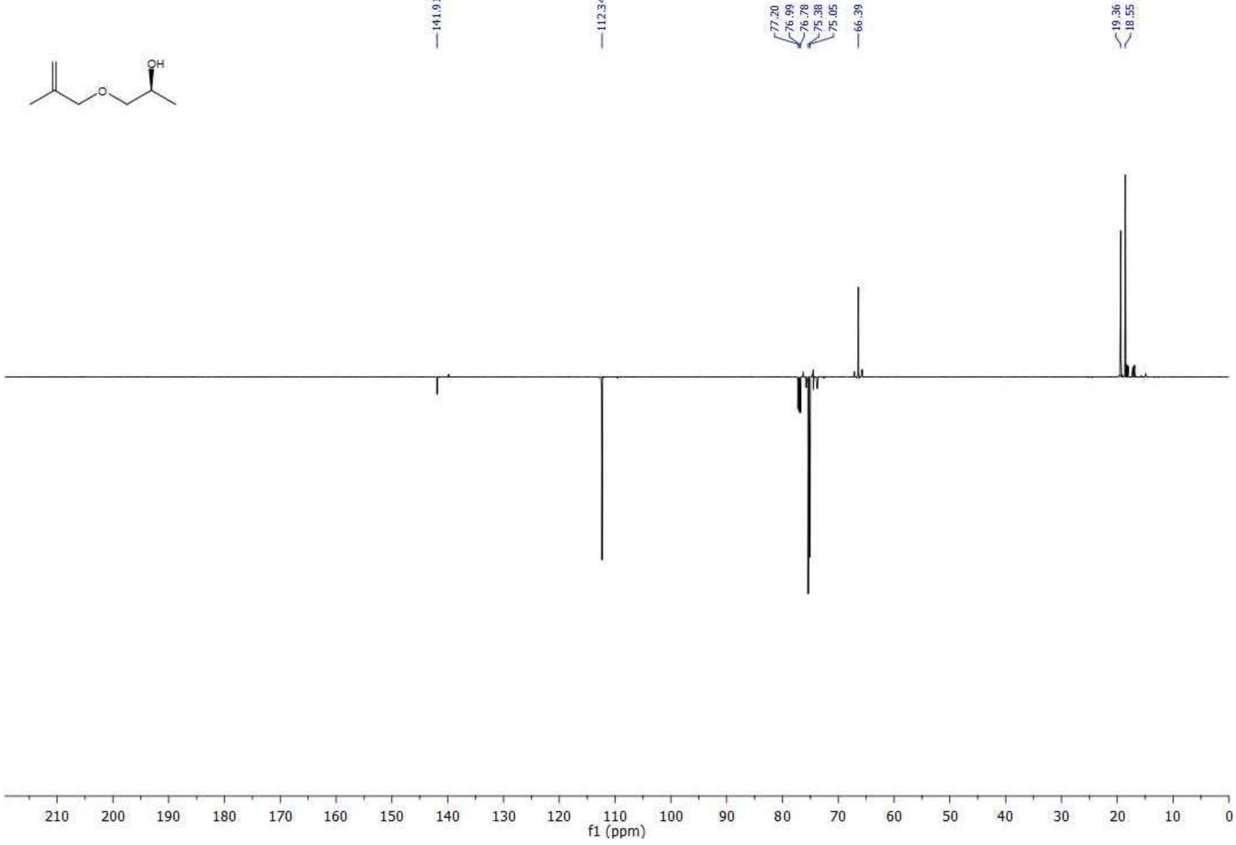
141.91
112.34
77.20
76.99
76.78
75.38
75.05
66.39
19.36
18.55
OH
f1 (ppm)

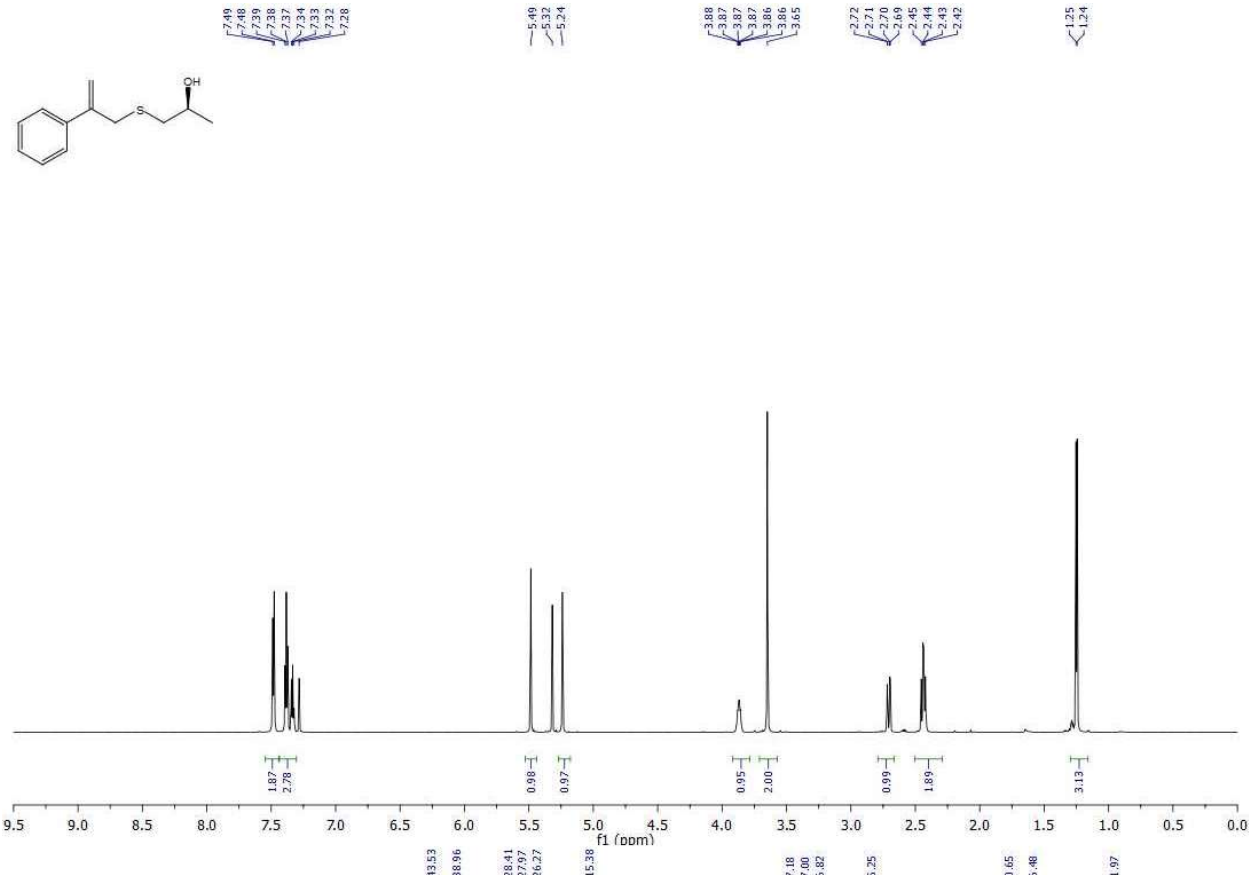
7.49 7.48 7.39 7.38 7.37 7.34 7.33 7.32 7.28
5.49 5.32 5.24
3.88 3.87 3.87 3.87 3.86 3.86 3.65
2.72 2.71 2.70 2.69 2.45 2.44 2.43 2.42
1.25 1.24
OH
S
1.87 2.78 0.98 0.97 0.95 2.00 0.99 1.89 3.13
9.5 9.0 8.5 8.0 7.5 7.0 6.5 6.0 5.5 5.0 4.5 4.0 3.5 3.0 2.5 2.0 1.5 1.0 0.5 0.0
f1 (ppm)

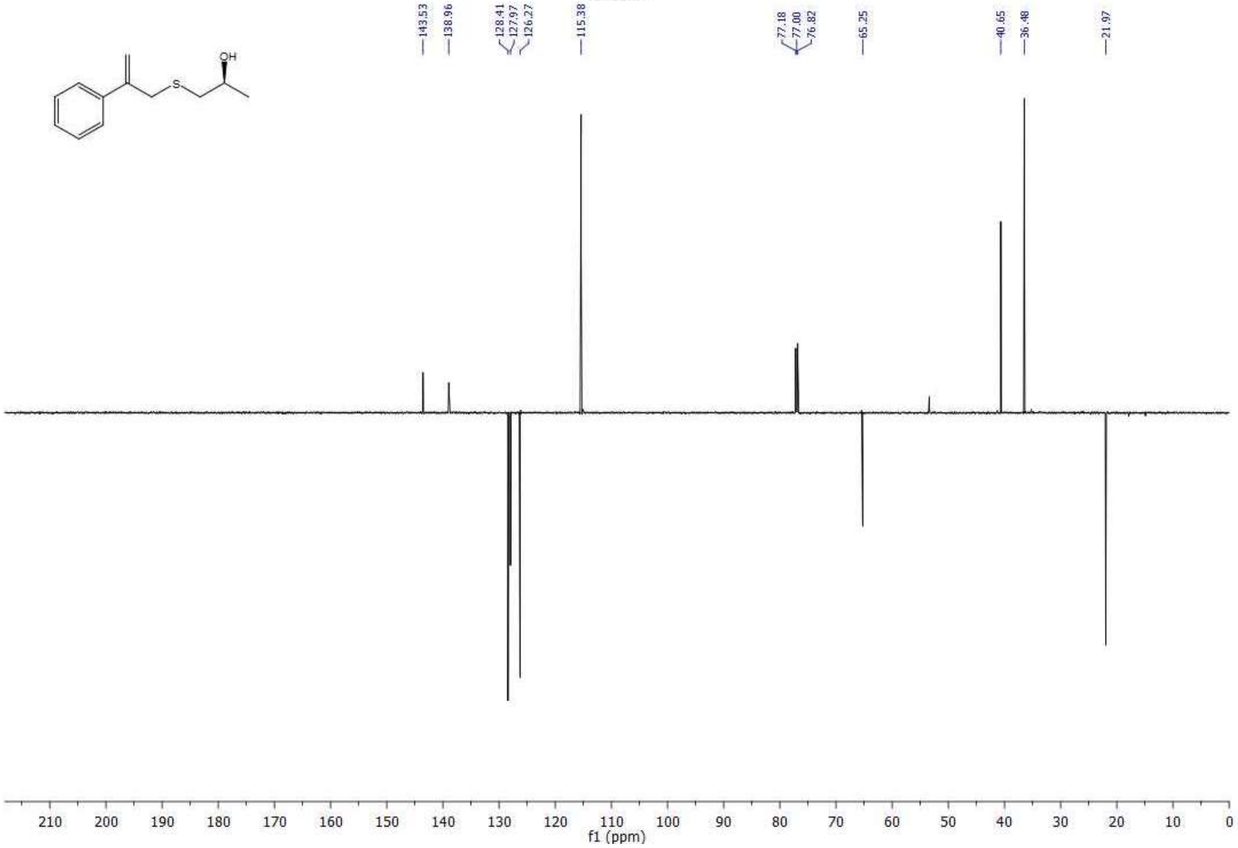
143.53 138.96 128.41 127.97 126.27 115.38 77.18 77.00 76.82 65.25 40.65 36.48 21.97
OH
S
210 200 190 180 170 160 150 140 130 120 110 100 90 80 70 60 50 40 30 20 10 0
f1 (ppm)

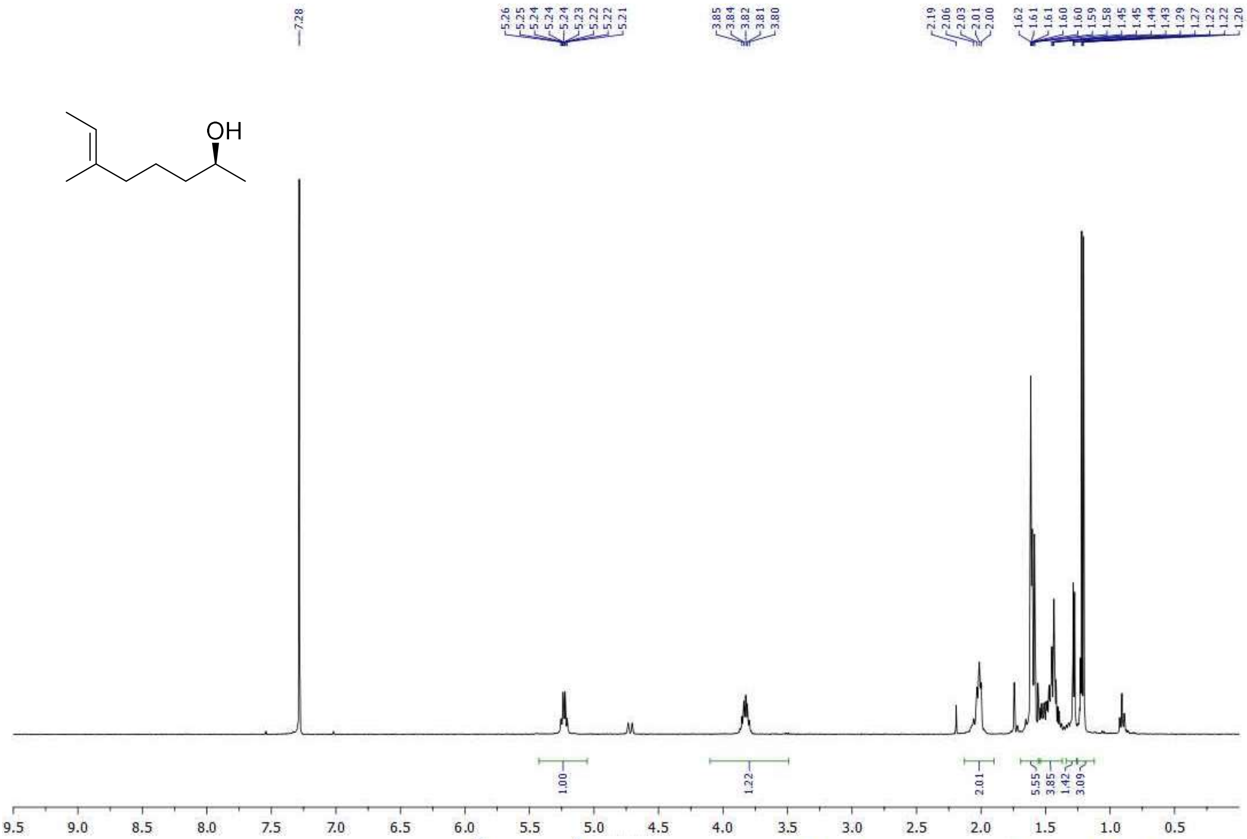
7.28
5.26
5.25
5.24
5.24
5.24
5.23
5.22
5.22
5.21
3.85
3.84
3.82
3.81
3.80
2.19
2.06
2.03
2.01
2.00
1.62
1.61
1.61
1.60
1.60
1.59
1.58
1.45
1.44
1.43
1.29
1.27
1.22
1.22
1.20
OH
1.00
1.22
2.01
5.55
3.85
1.42
3.09

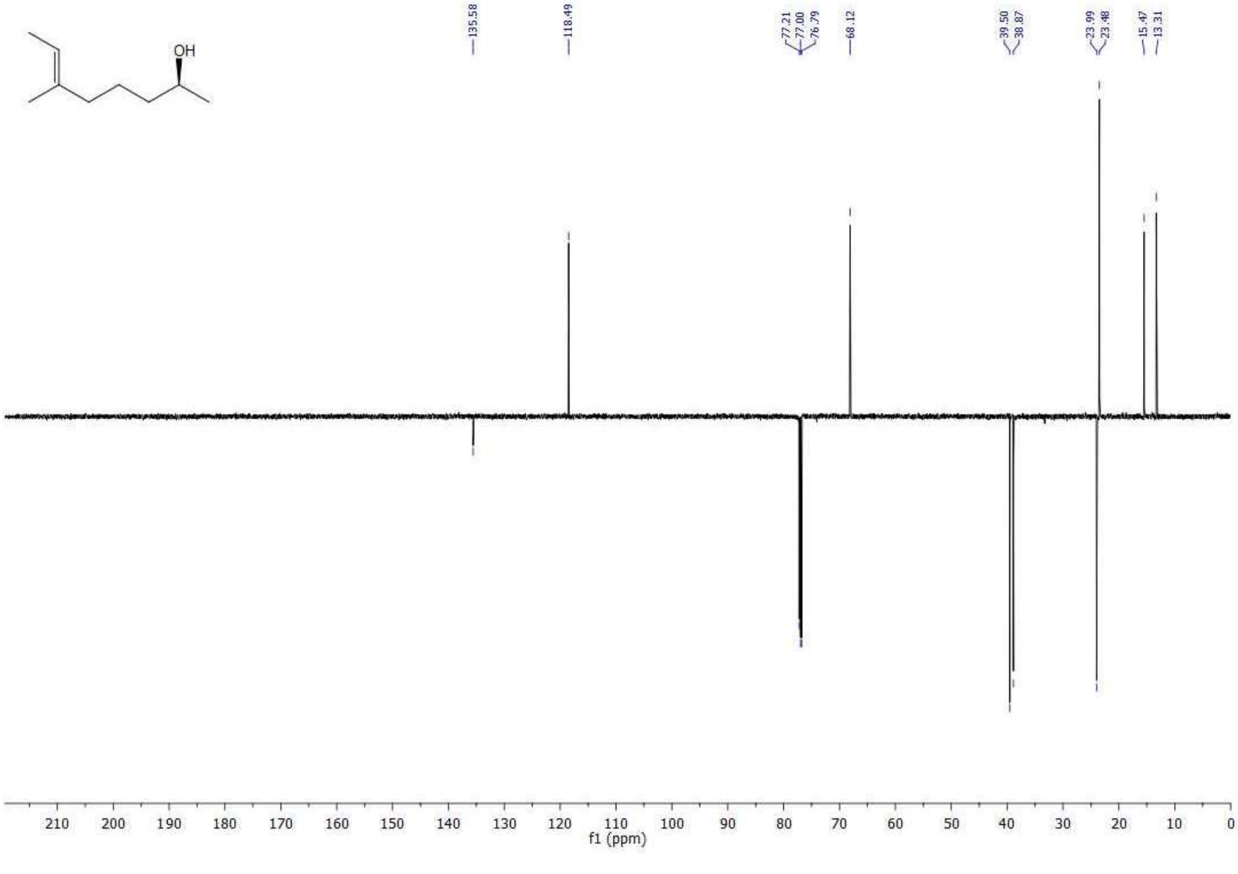
135.58
118.49
77.21
77.00
76.79
68.12
39.50
38.87
23.99
23.48
15.47
13.31
OH
f1 (ppm)

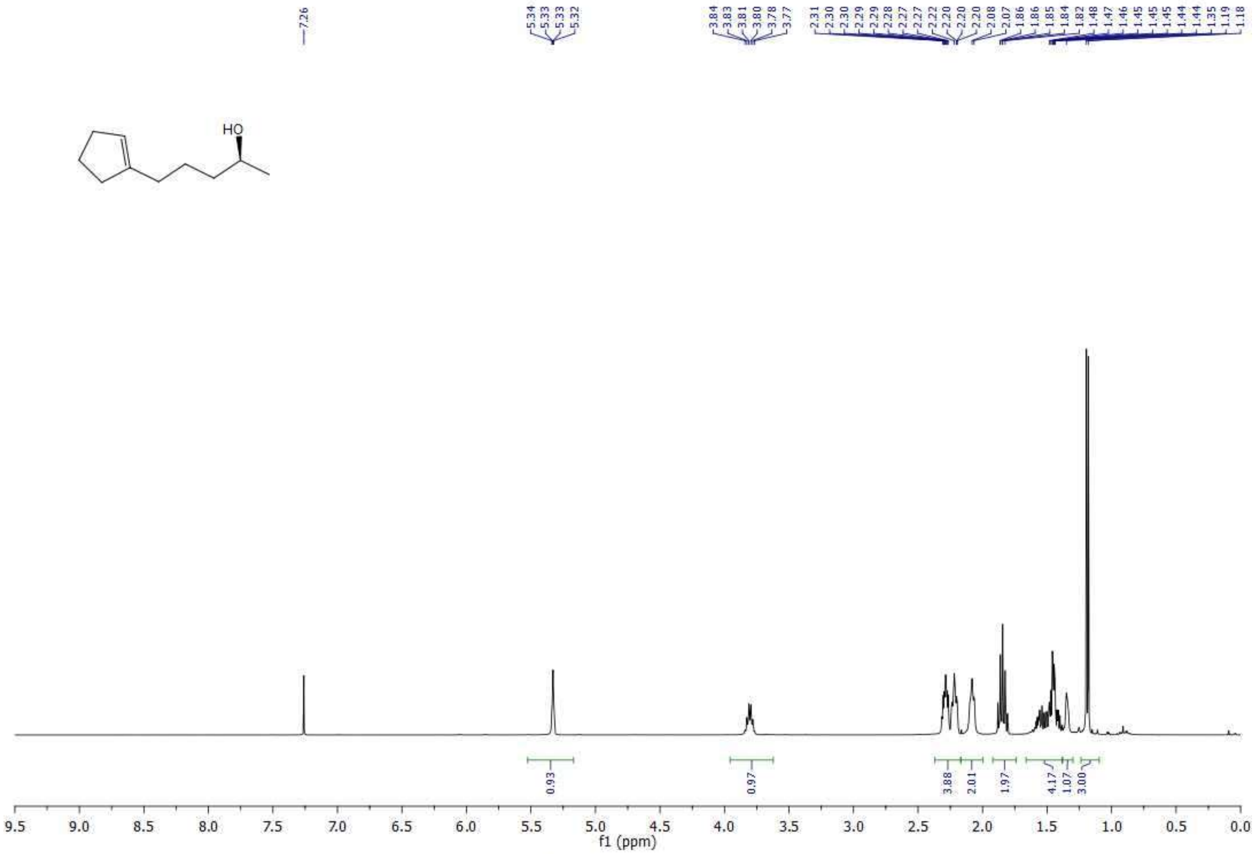

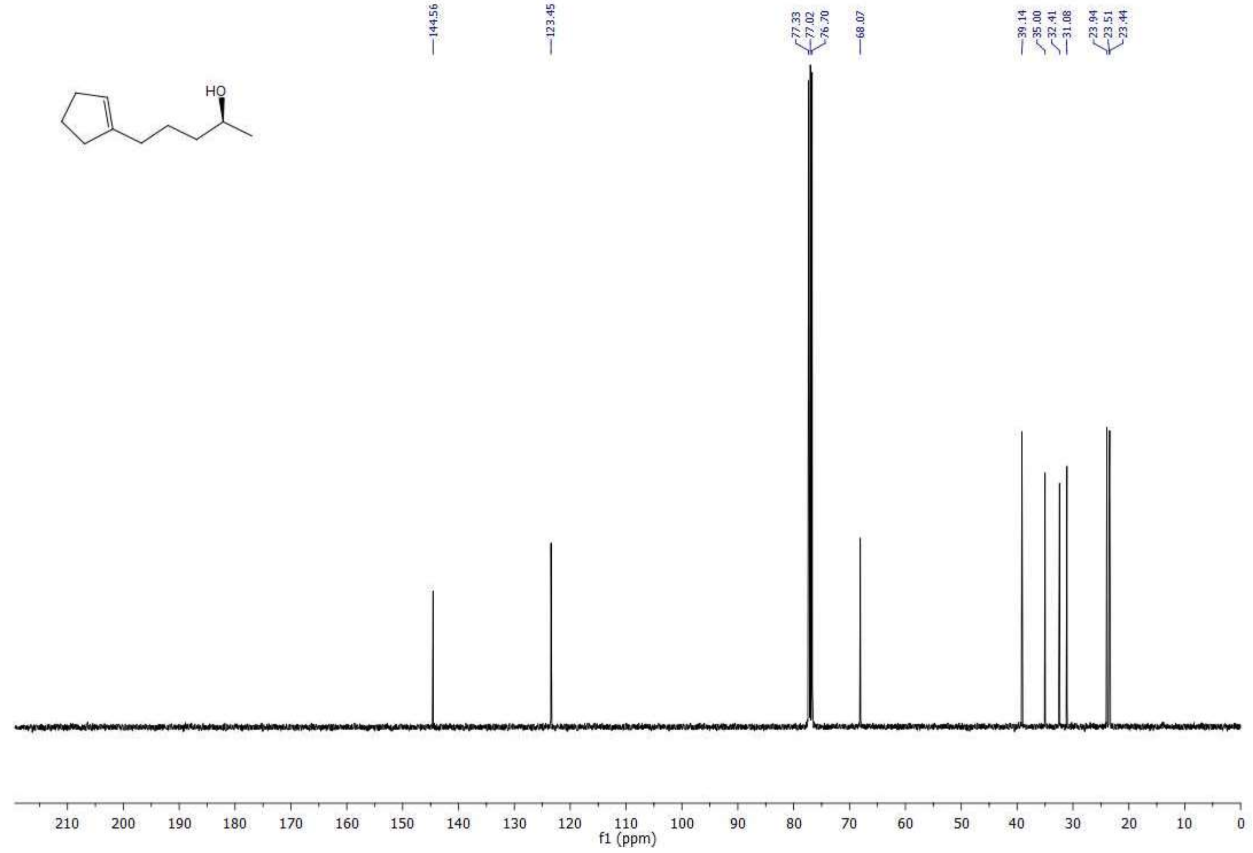

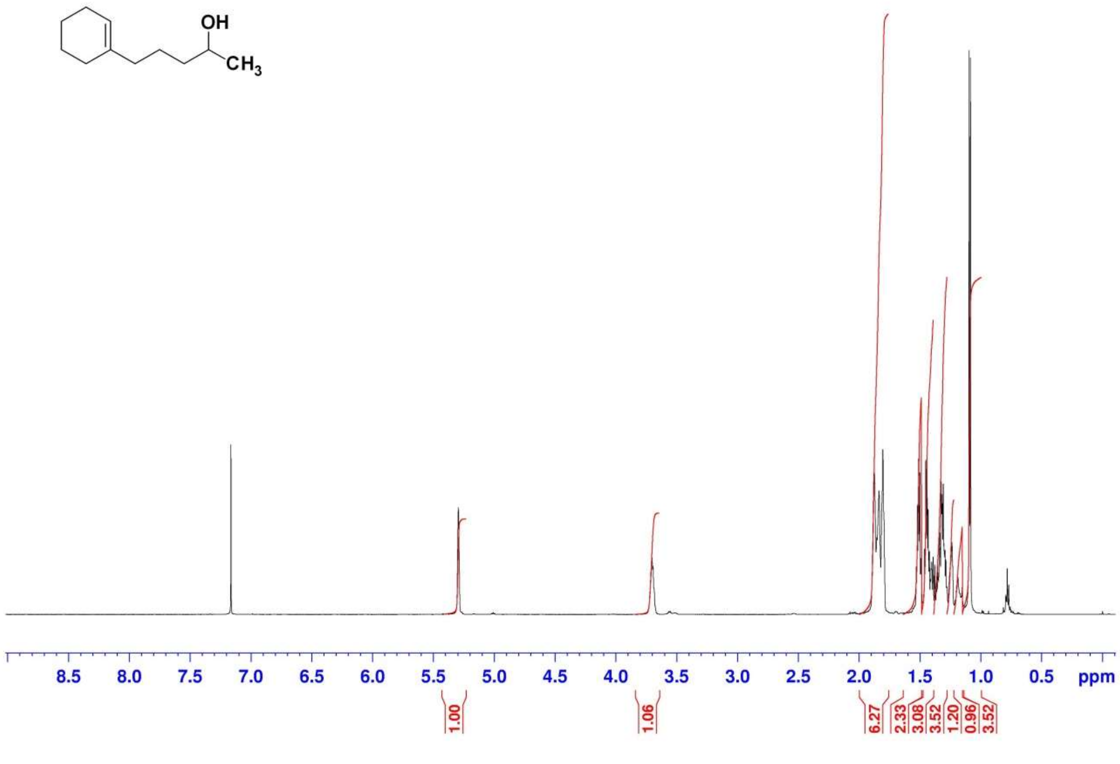
OH
CH3
8.5 8.0 7.5 7.0 6.5 6.0 5.5 5.0 4.5 4.0 3.5 3.0 2.5 2.0 1.5 1.0 0.5 ppm
1.00
1.06
6.27 2.33 3.08 3.52 1.20 0.96 3.52

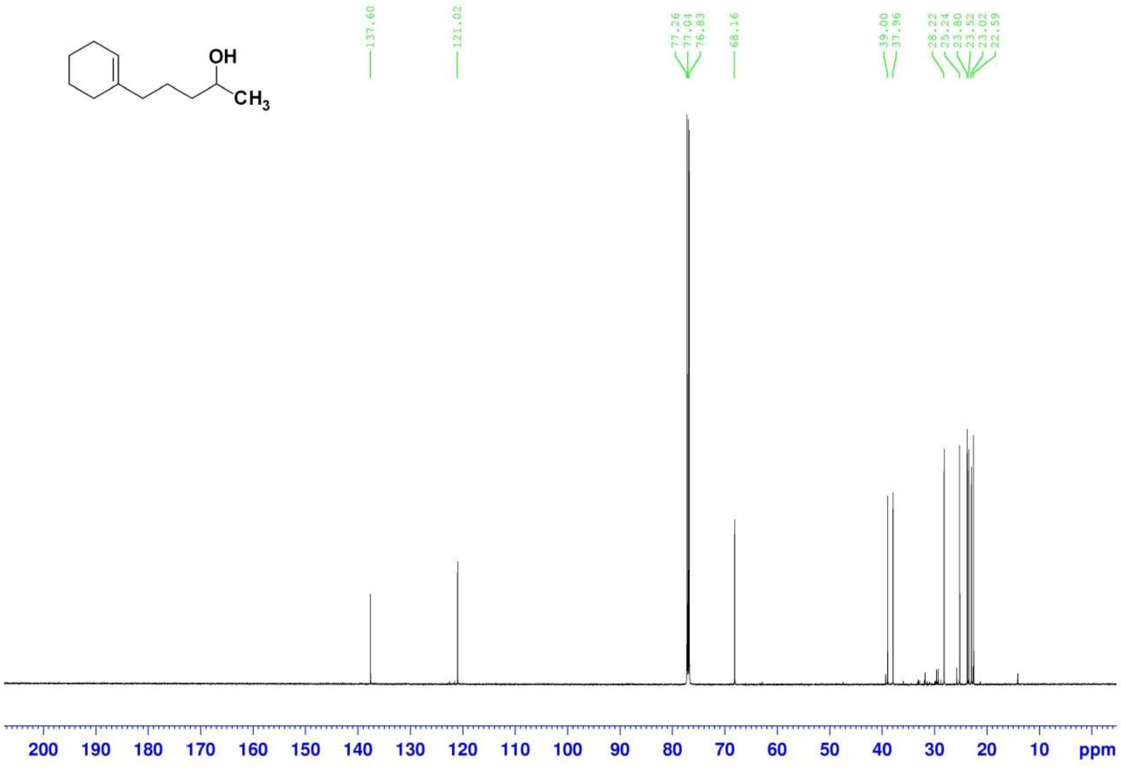
137.60
121.02
77.26 77.04 76.83
68.16
39.00 37.96
28.22 25.24 23.80 23.52 23.02 22.59
OH
CH3
200 190 180 170 160 150 140 130 120 110 100 90 80 70 60 50 40 30 20 10 ppm

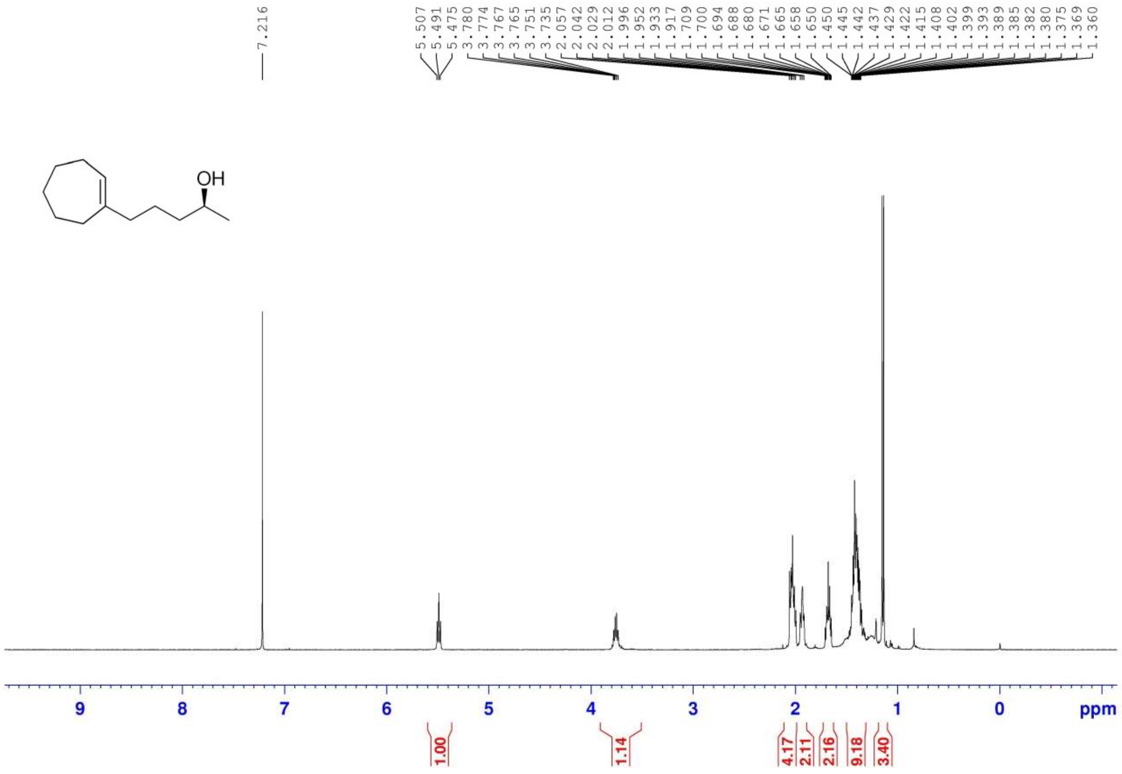
OH
9
8
7
6
5
4
3
2
1
0
ppm
1.00
1.14
4.17
2.11
2.16
9.18
3.40

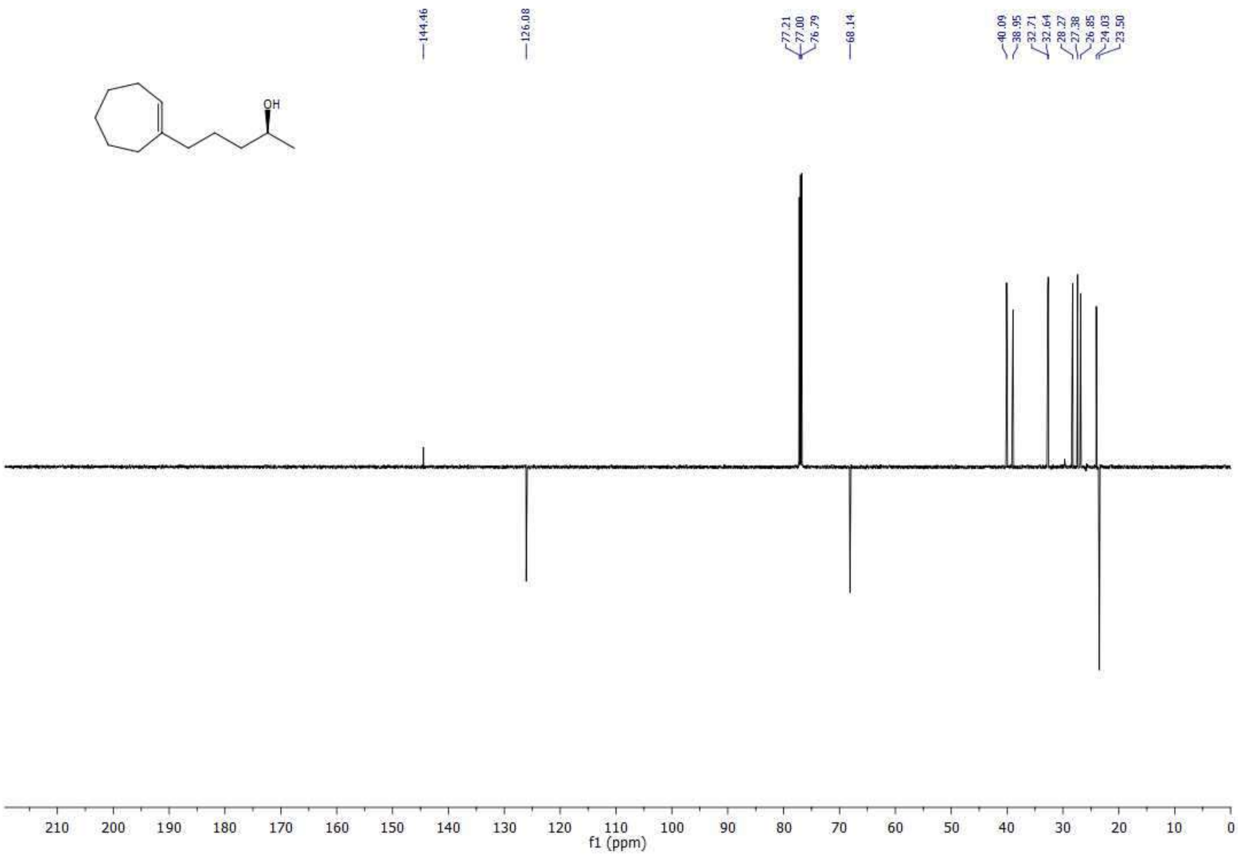
OH
144.46
126.08
77.21
77.00
76.79
68.14
40.09
38.95
32.71
32.64
28.27
27.38
26.85
24.03
23.50
f1 (ppm)

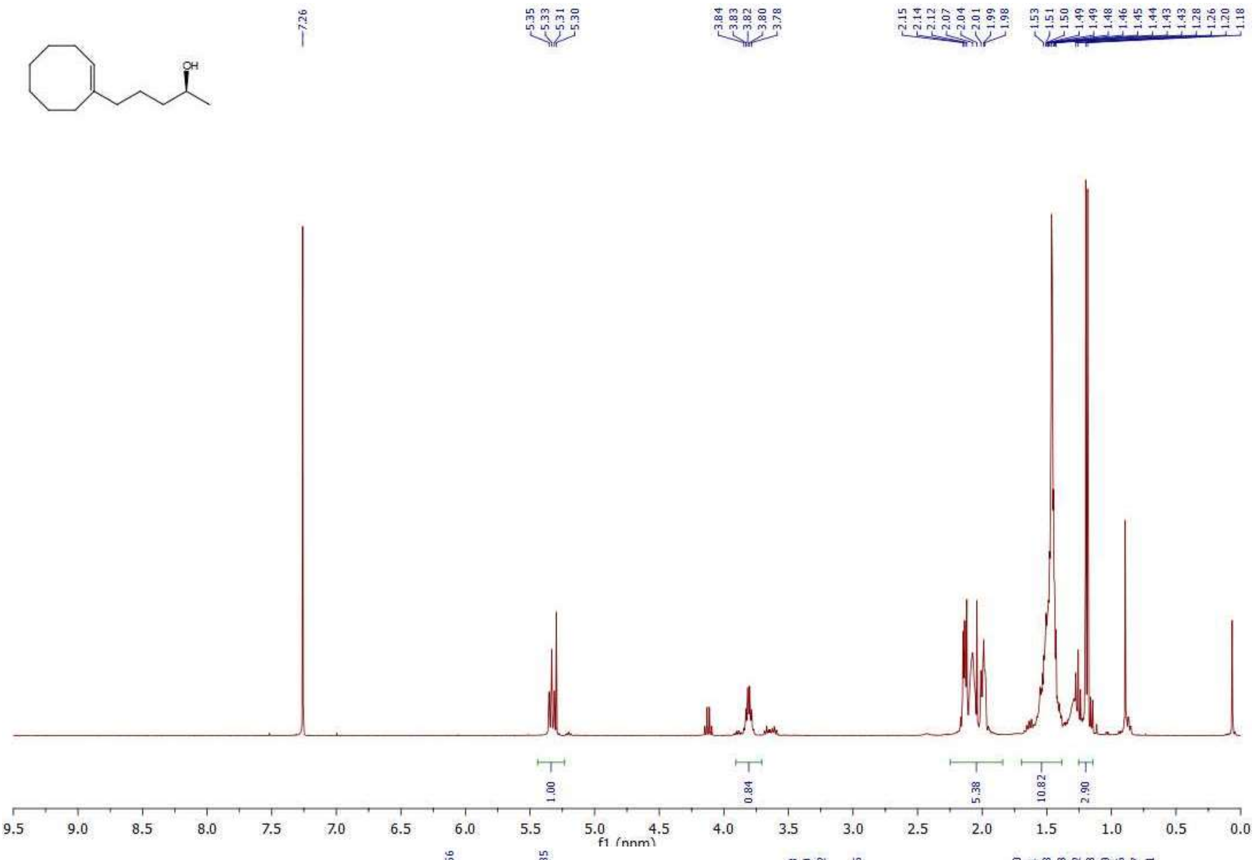
OH
7.26
5.35
5.33
5.31
5.30
3.84
3.83
3.82
3.80
3.78
2.15
2.14
2.12
2.07
2.04
2.01
1.99
1.98
1.53
1.51
1.50
1.49
1.49
1.48
1.46
1.45
1.44
1.43
1.28
1.26
1.20
1.18
1.00
0.84
5.38
10.82
2.90
f1 (ppm)

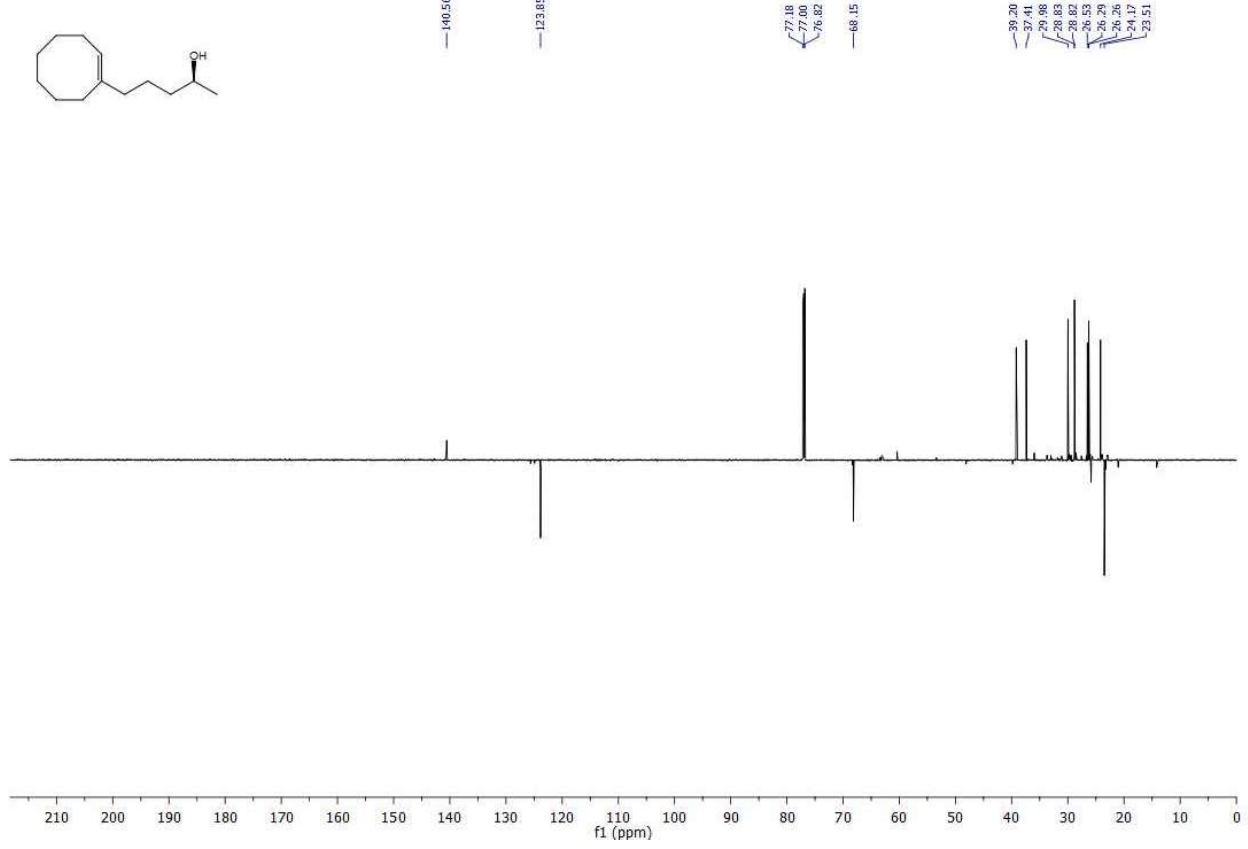
OH
140.56
123.85
77.18
77.00
76.82
68.15
39.20
37.41
29.98
28.83
28.82
26.53
26.29
26.26
24.17
23.51
f1 (ppm)

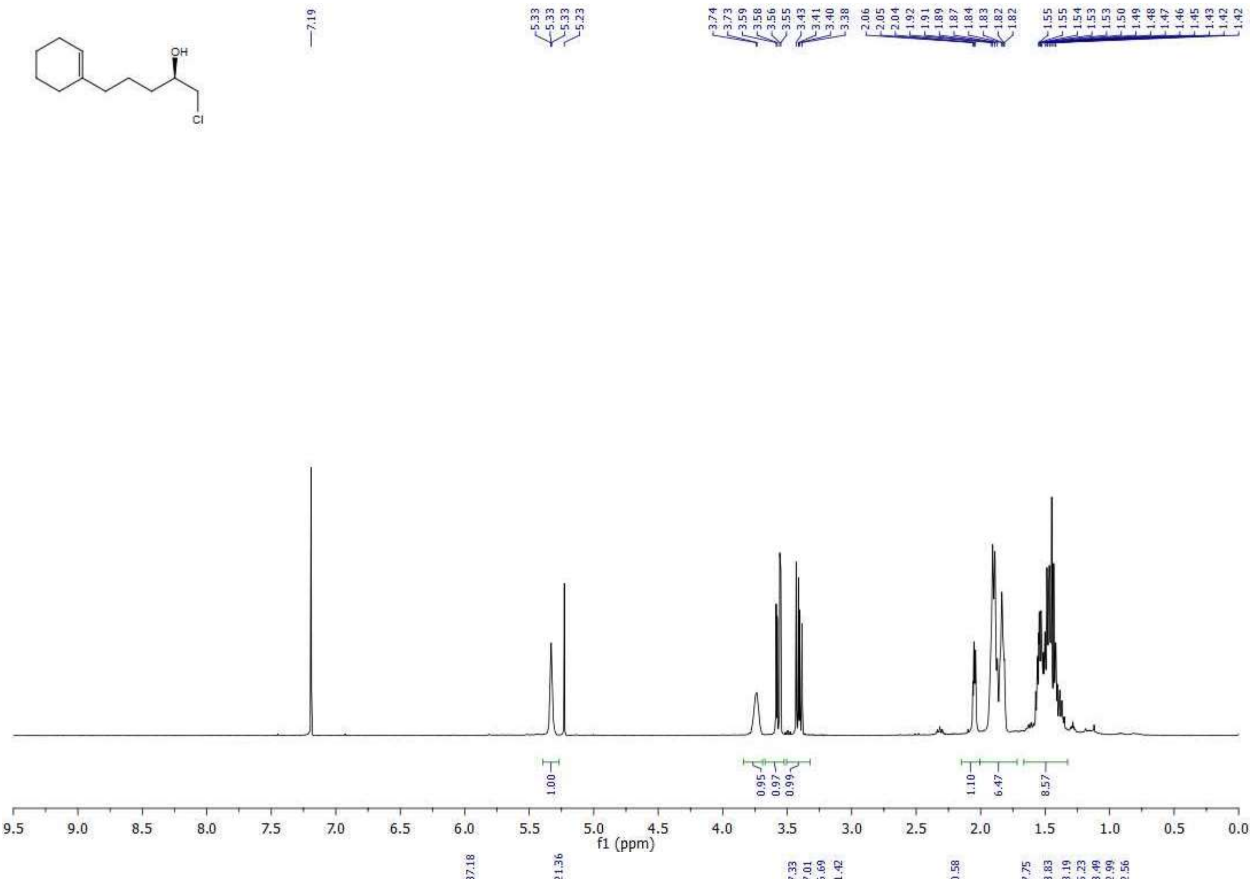

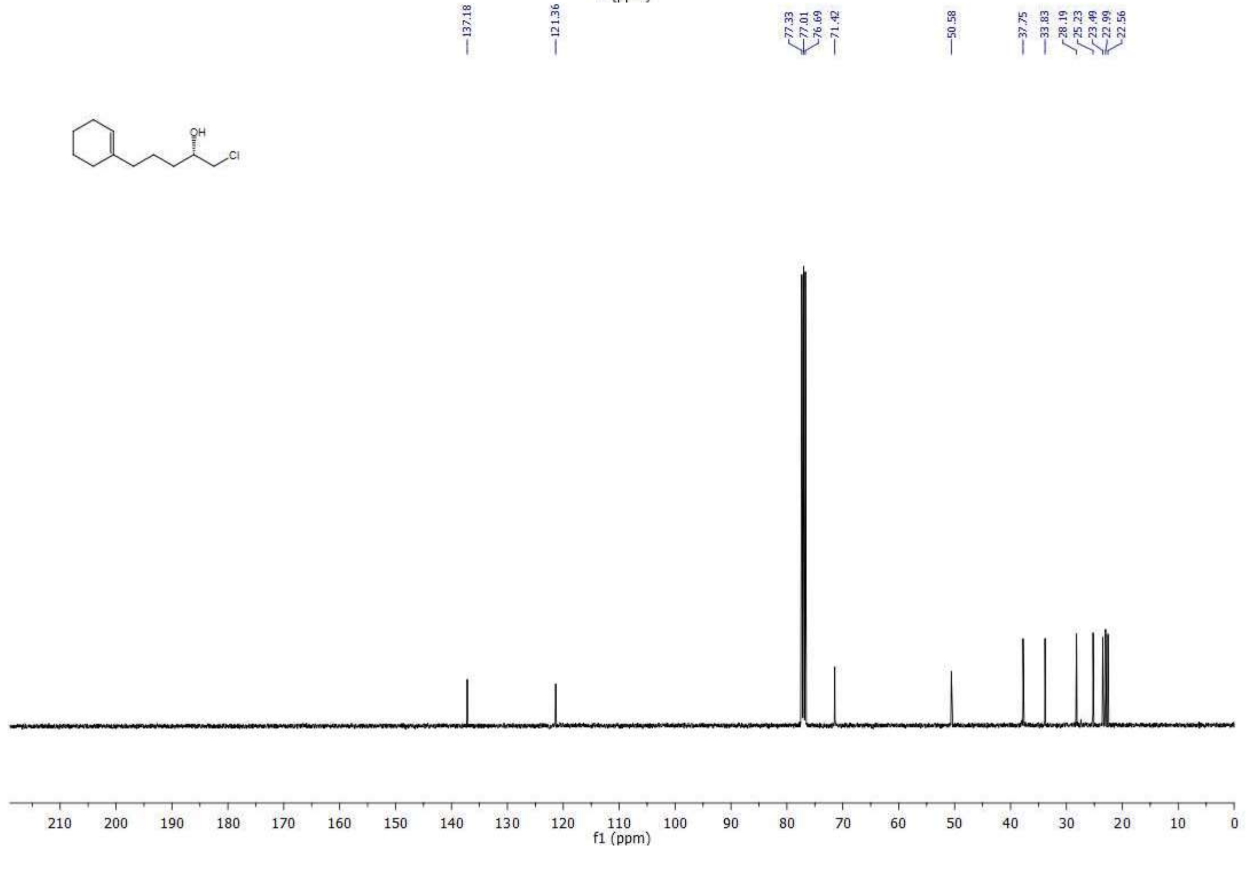

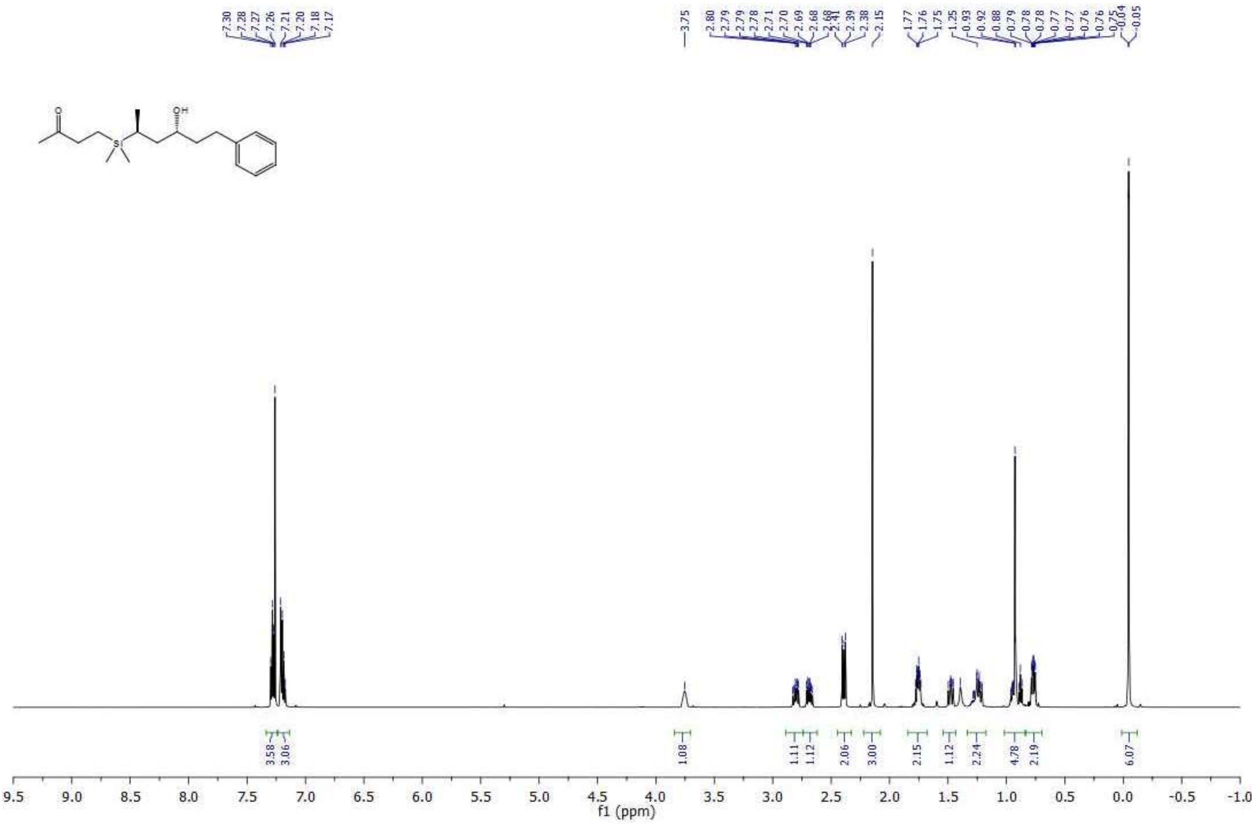

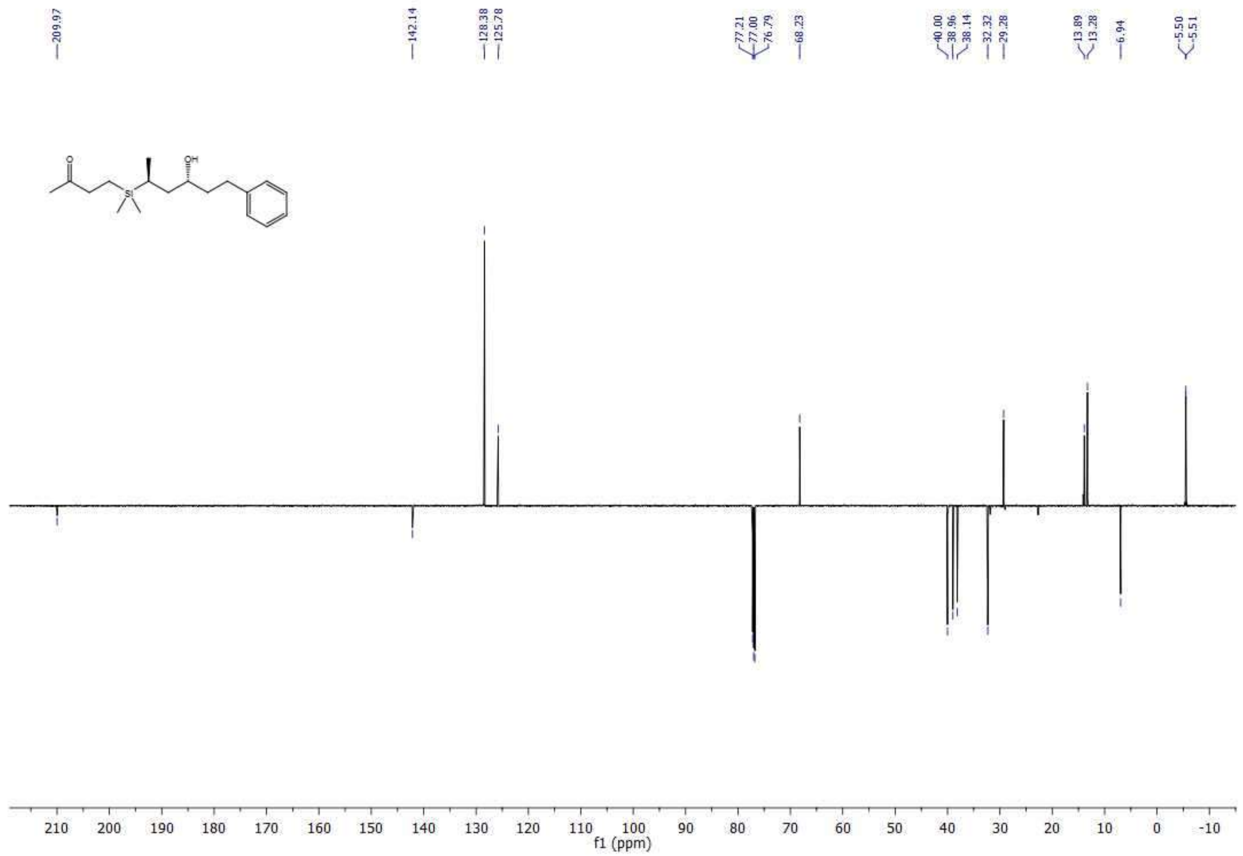

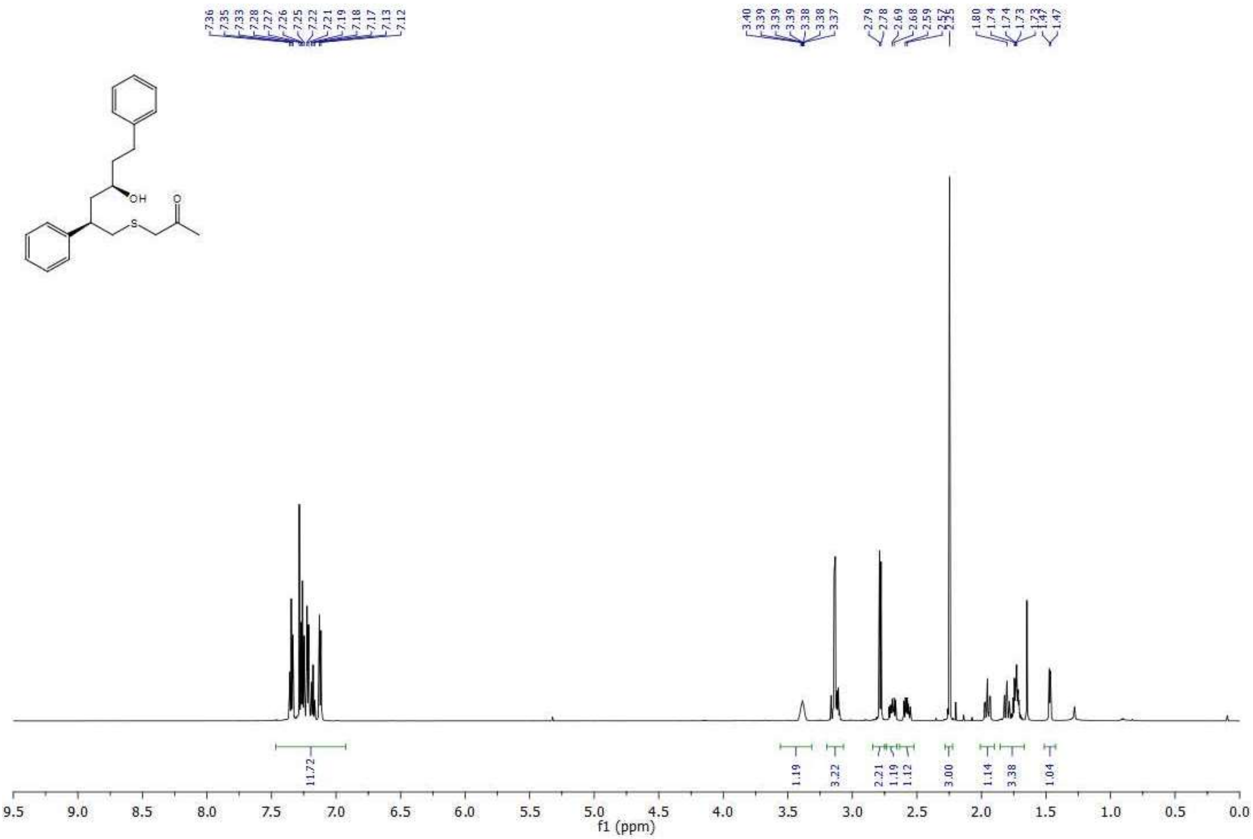
7.36
7.35
7.33
7.28
7.27
7.26
7.25
7.22
7.21
7.19
7.18
7.17
7.13
7.12
3.40
3.39
3.39
3.39
3.38
3.38
3.37
2.79
2.78
2.69
2.68
2.59
2.57
2.25
1.80
1.74
1.74
1.73
1.73
1.47
1.47
OH
S
O
11.72
1.19
3.22
2.21
1.19
1.12
3.00
1.14
3.38
1.04
f1 (ppm)

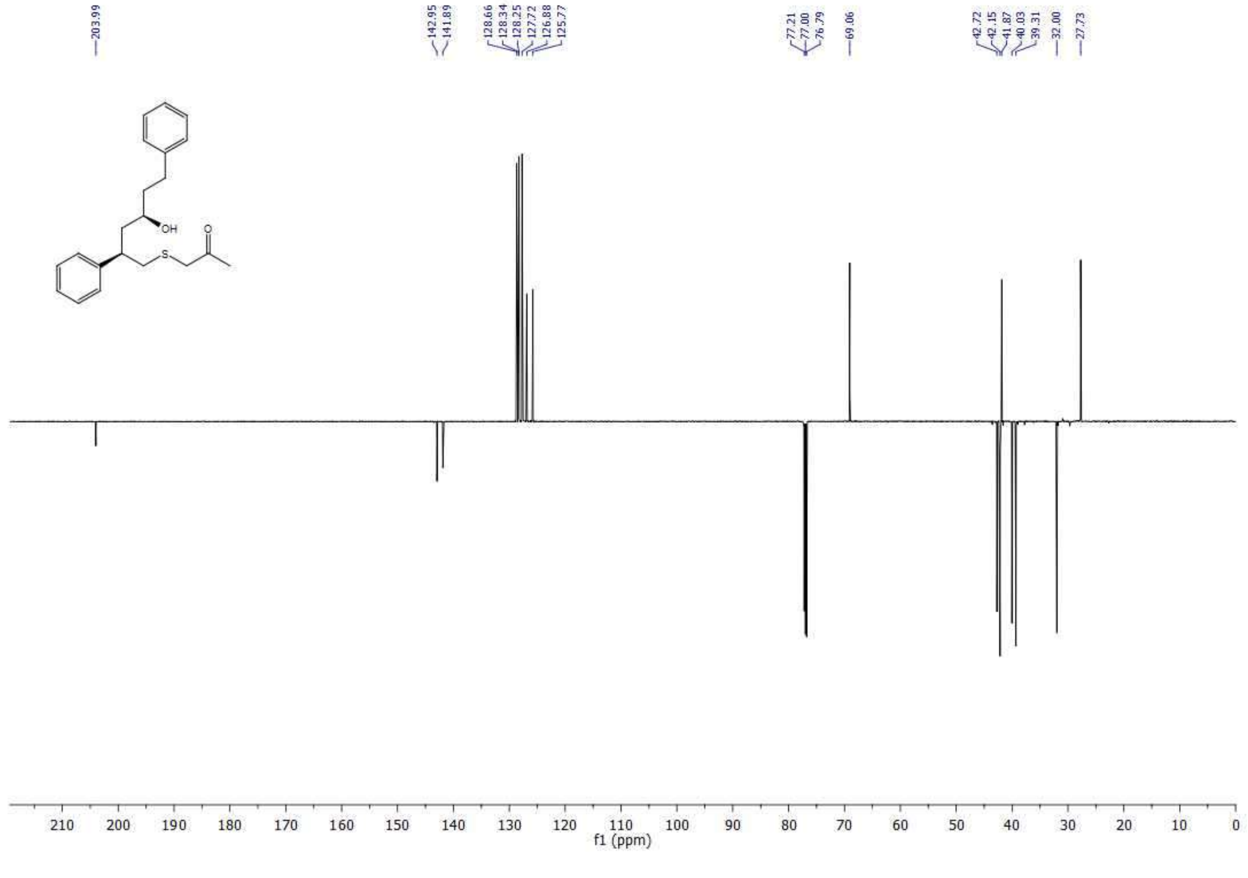
203.99
142.95
141.89
128.66
128.34
128.25
127.72
126.88
125.77
77.21
77.00
76.79
69.06
42.72
42.15
41.87
40.03
39.31
32.00
27.73
OH
S
O
f1 (ppm)

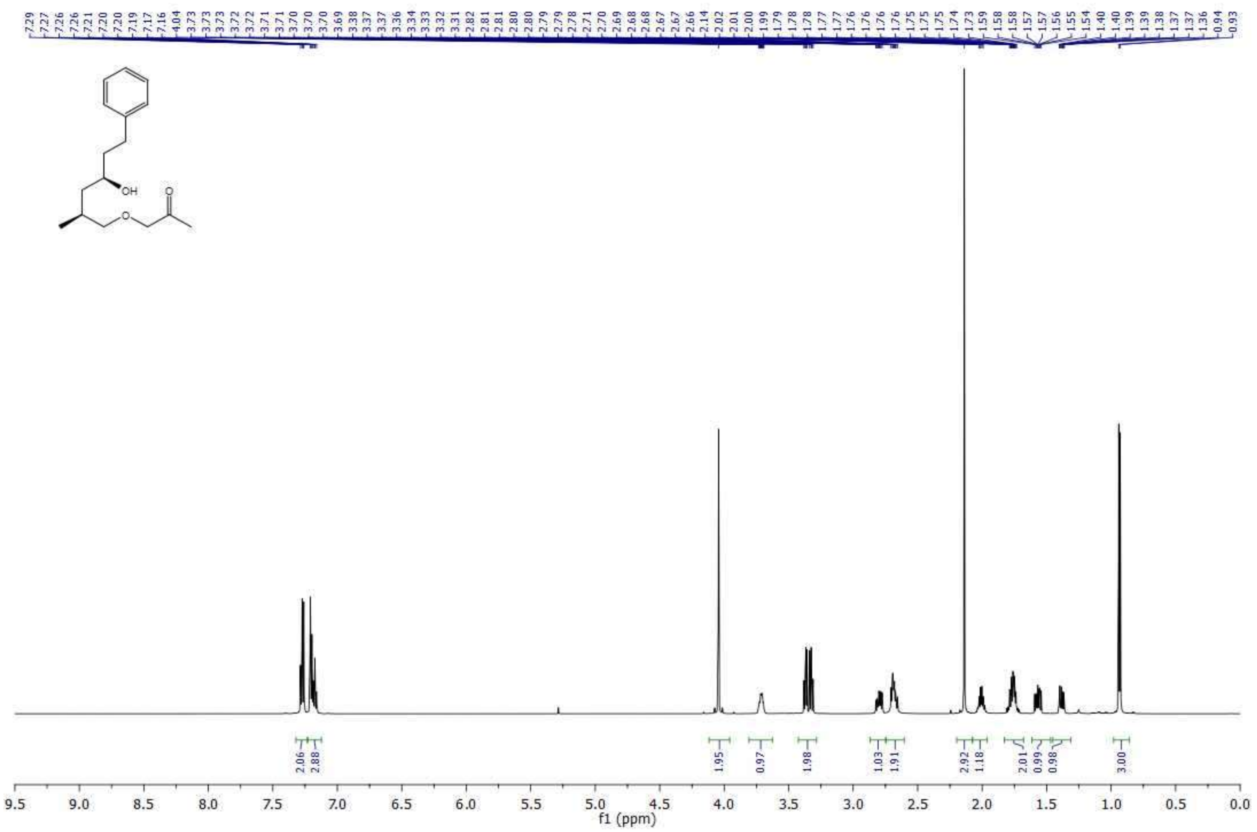

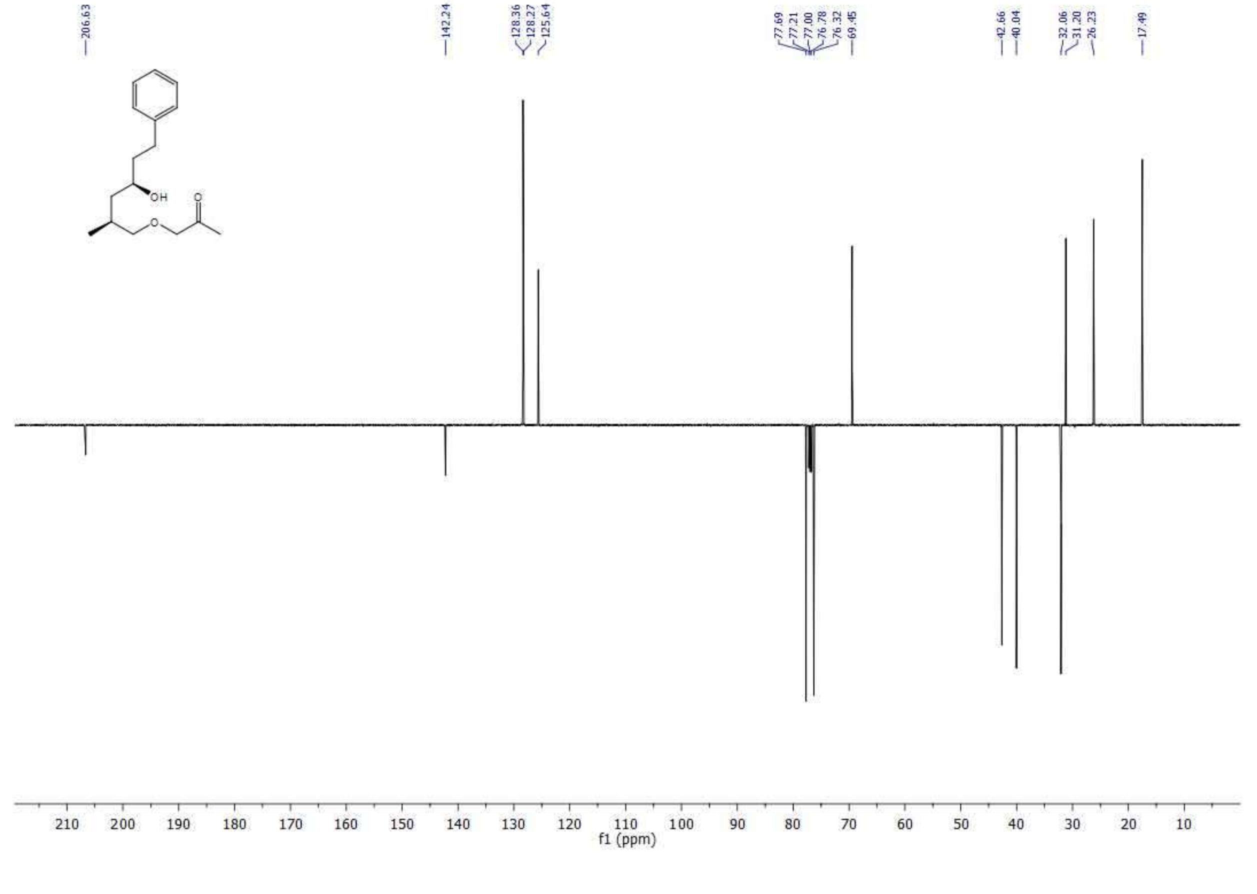

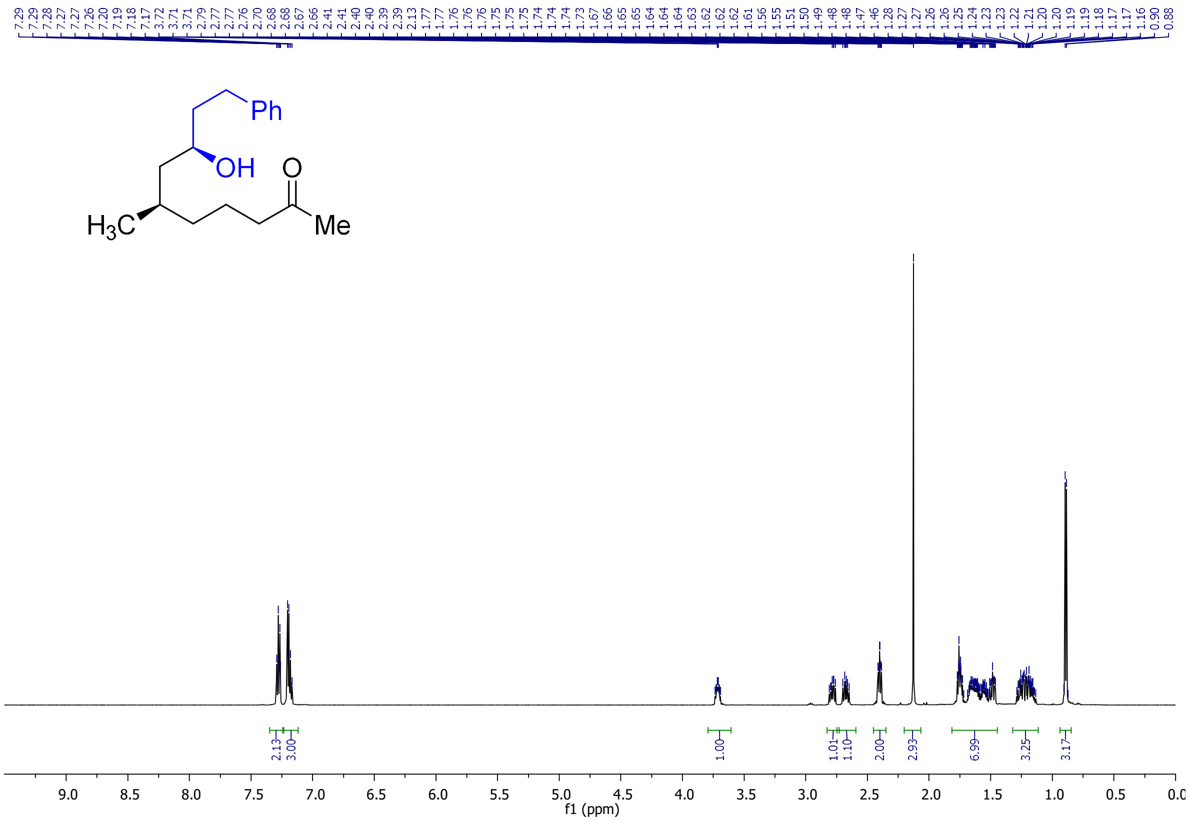

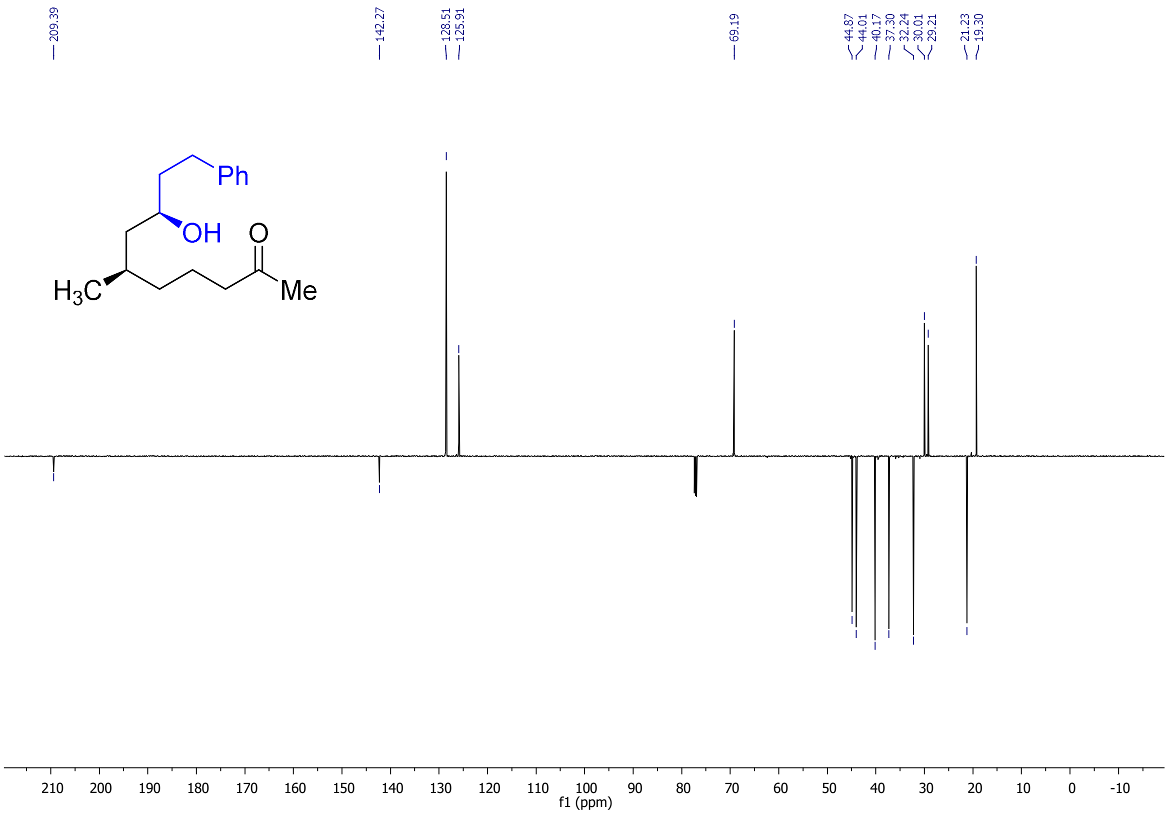

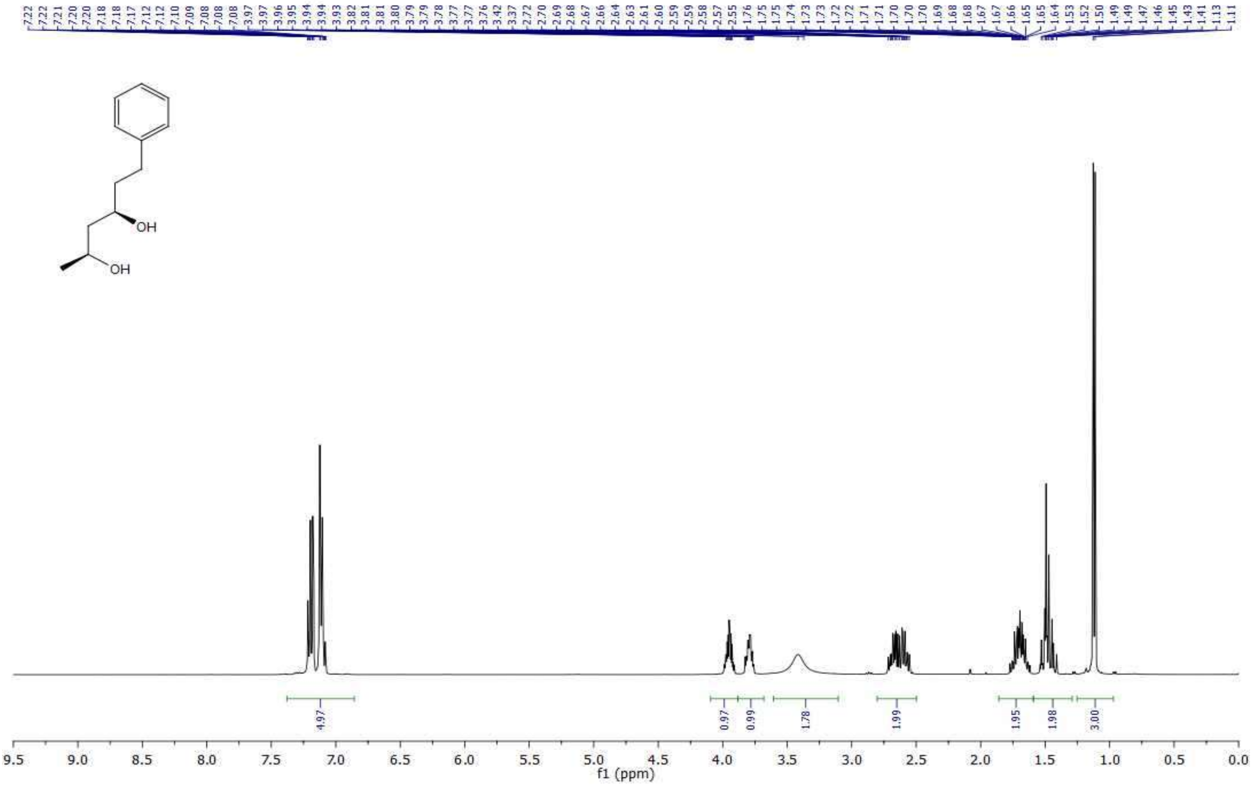
7.22
7.22
7.21
7.20
7.20
7.18
7.18
7.17
7.12
7.12
7.10
7.09
7.08
7.08
7.08
3.97
3.97
3.96
3.95
3.94
3.94
3.93
3.82
3.81
3.81
3.80
3.79
3.79
3.78
3.77
3.77
3.76
3.42
3.37
2.72
2.70
2.69
2.68
2.67
2.66
2.64
2.63
2.61
2.60
2.59
2.59
2.58
2.57
2.55
1.76
1.75
1.75
1.74
1.73
1.73
1.72
1.72
1.71
1.71
1.70
1.70
1.70
1.69
1.68
1.68
1.67
1.67
1.66
1.65
1.65
1.64
1.53
1.52
1.50
1.49
1.49
1.47
1.46
1.45
1.43
1.41
1.13
1.11
OH
OH
4.97
0.97
0.99
1.78
1.99
1.95
1.98
3.00
9.5
9.0
8.5
8.0
7.5
7.0
6.5
6.0
5.5
5.0
4.5
4.0
3.5
3.0
2.5
2.0
1.5
1.0
0.5
0.0
f1 (ppm)

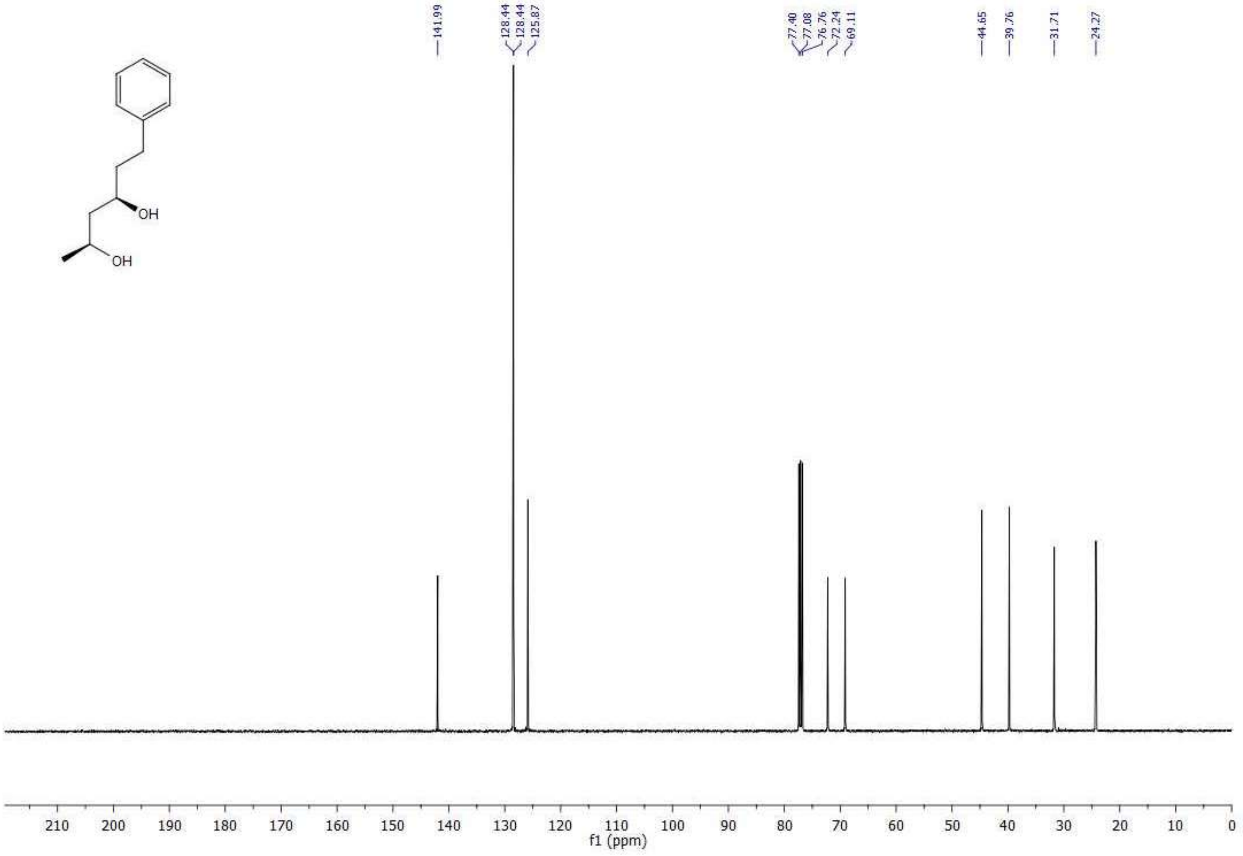
141.99
128.44
128.44
125.87
77.40
77.08
76.76
72.24
69.11
44.65
39.76
31.71
24.27
OH
OH
210
200
190
180
170
160
150
140
130
120
110
100
90
80
70
60
50
40
30
20
10
0
f1 (ppm)

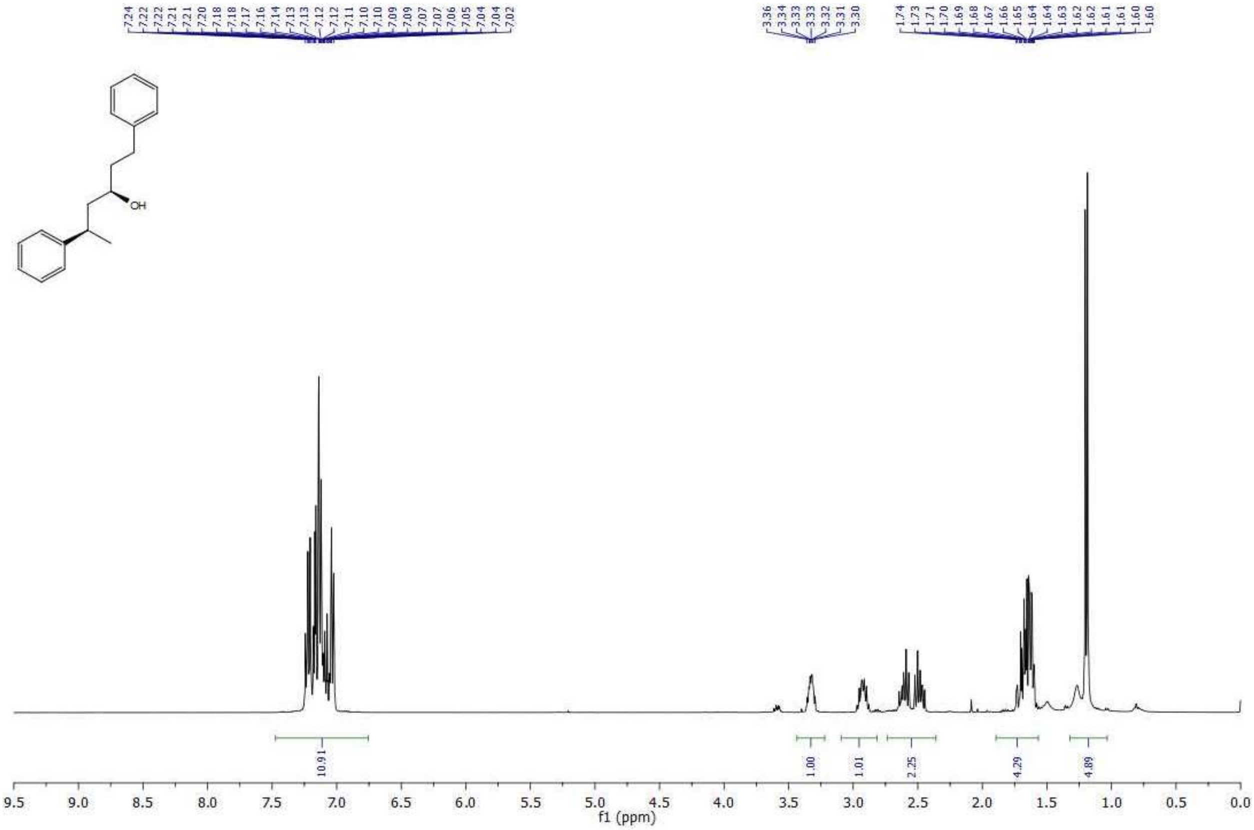

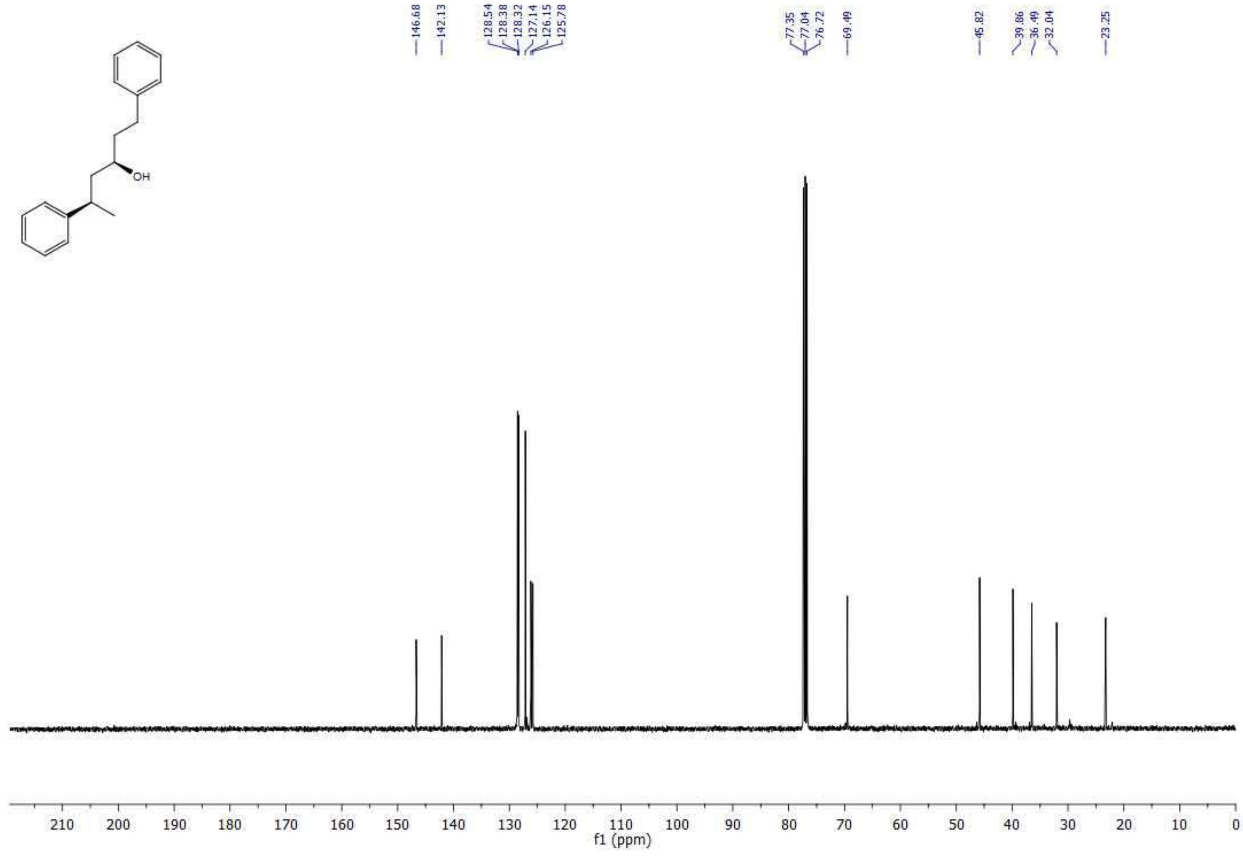

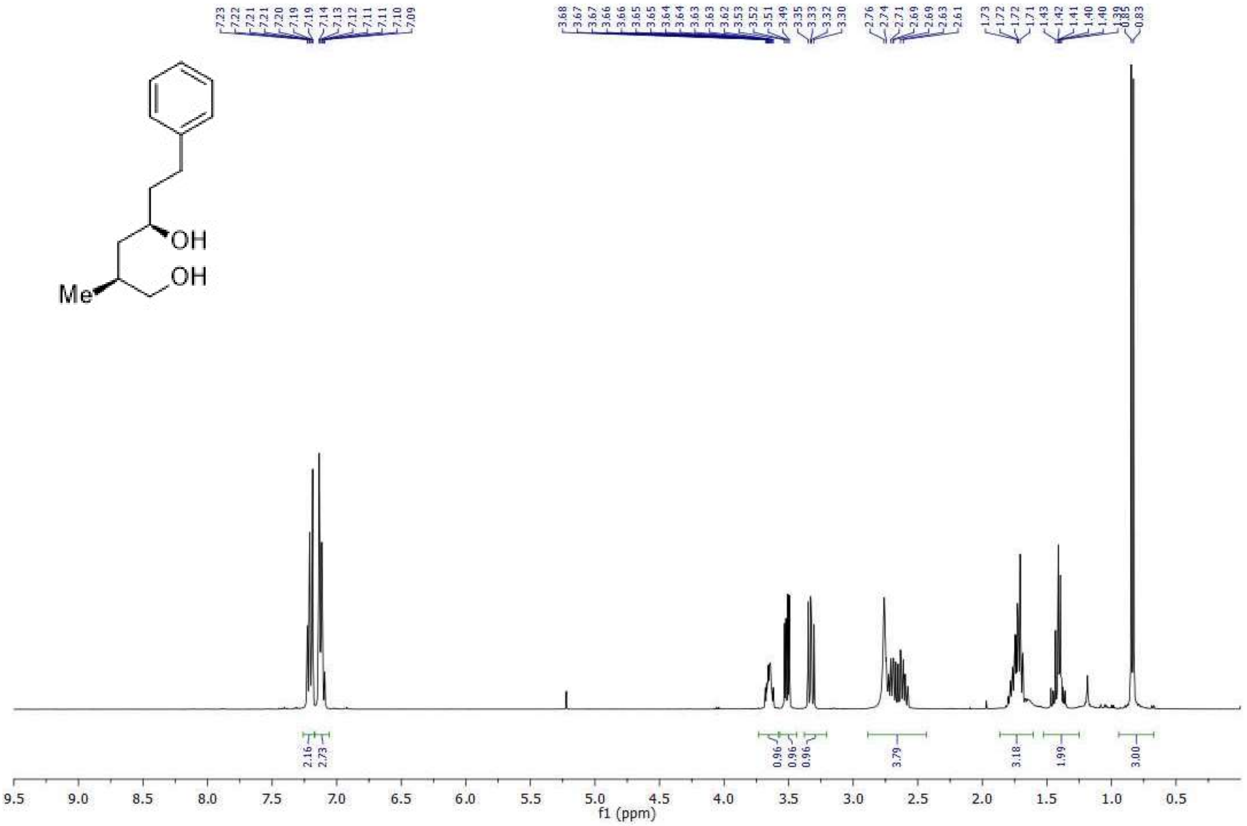
OH
OH
Me
f1 (ppm)

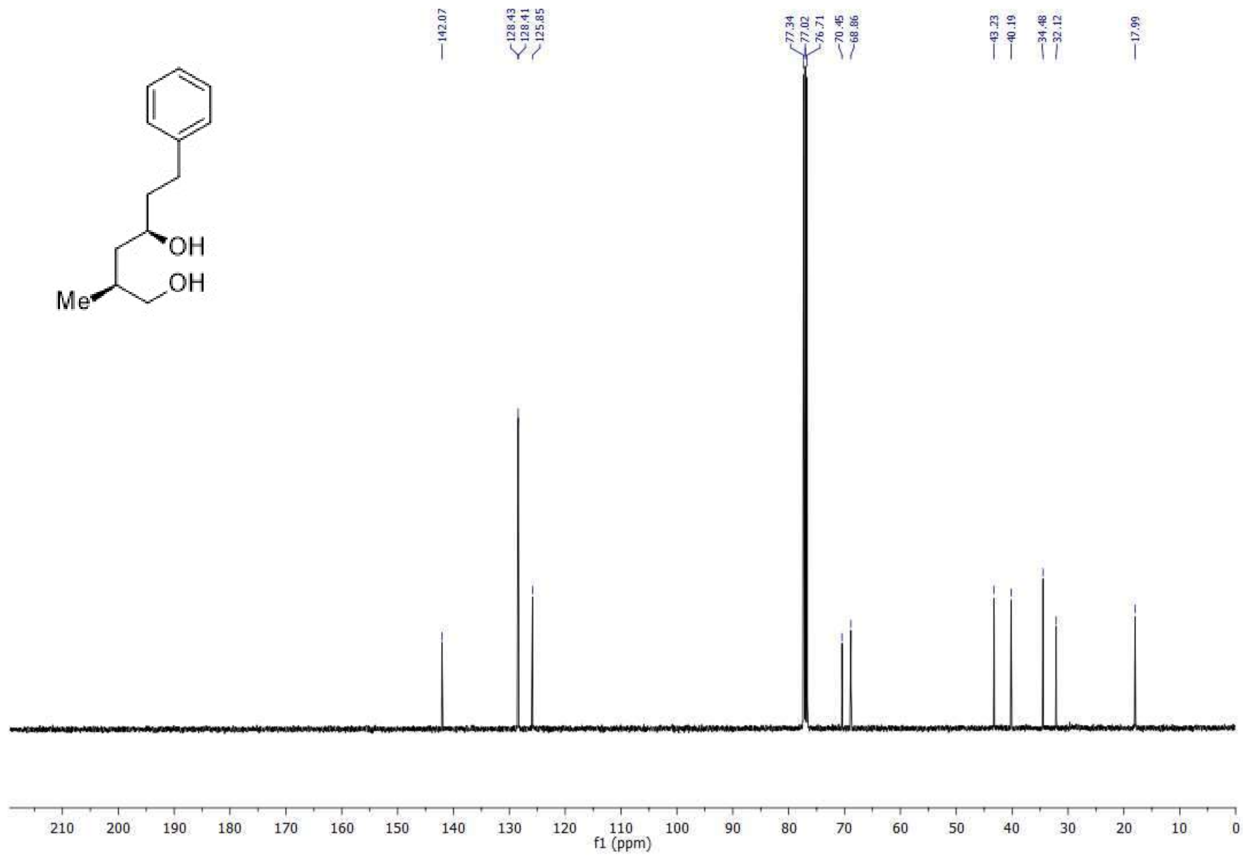
OH
OH
Me
f1 (ppm)

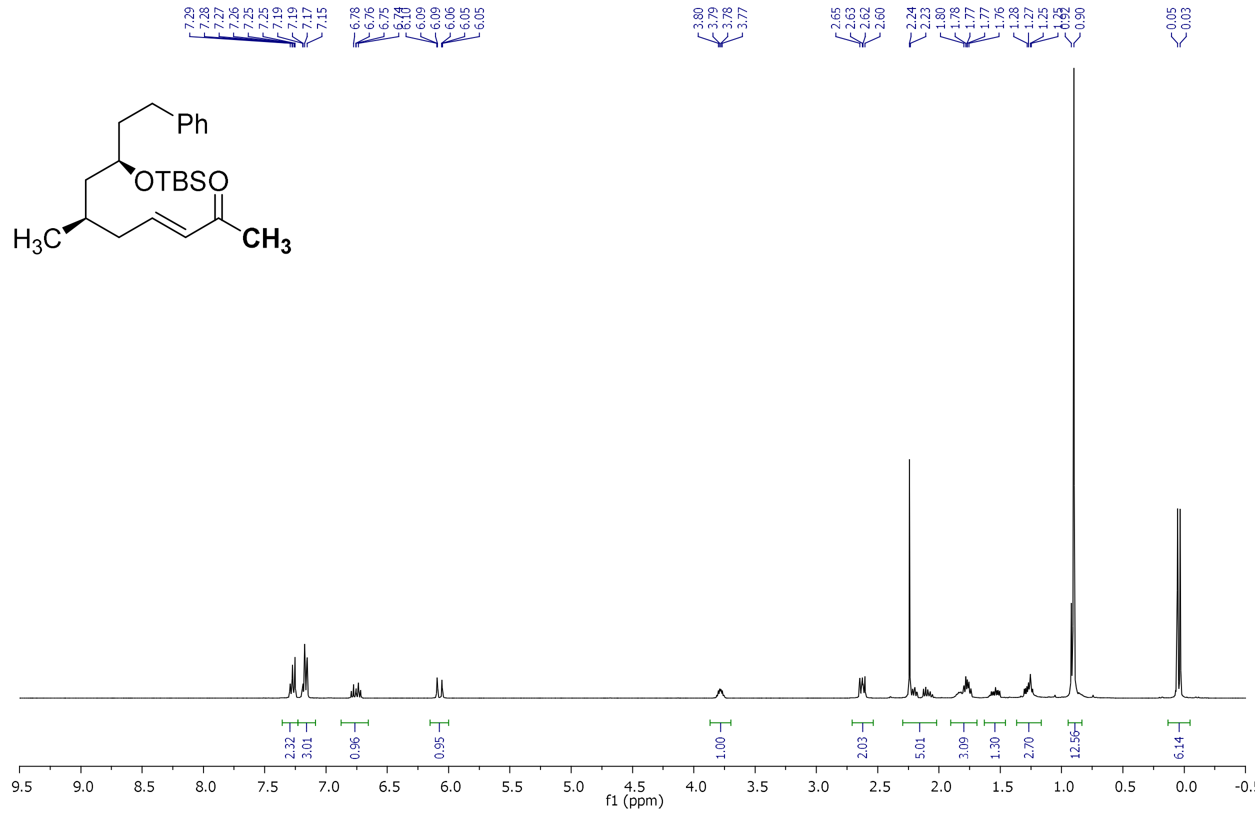

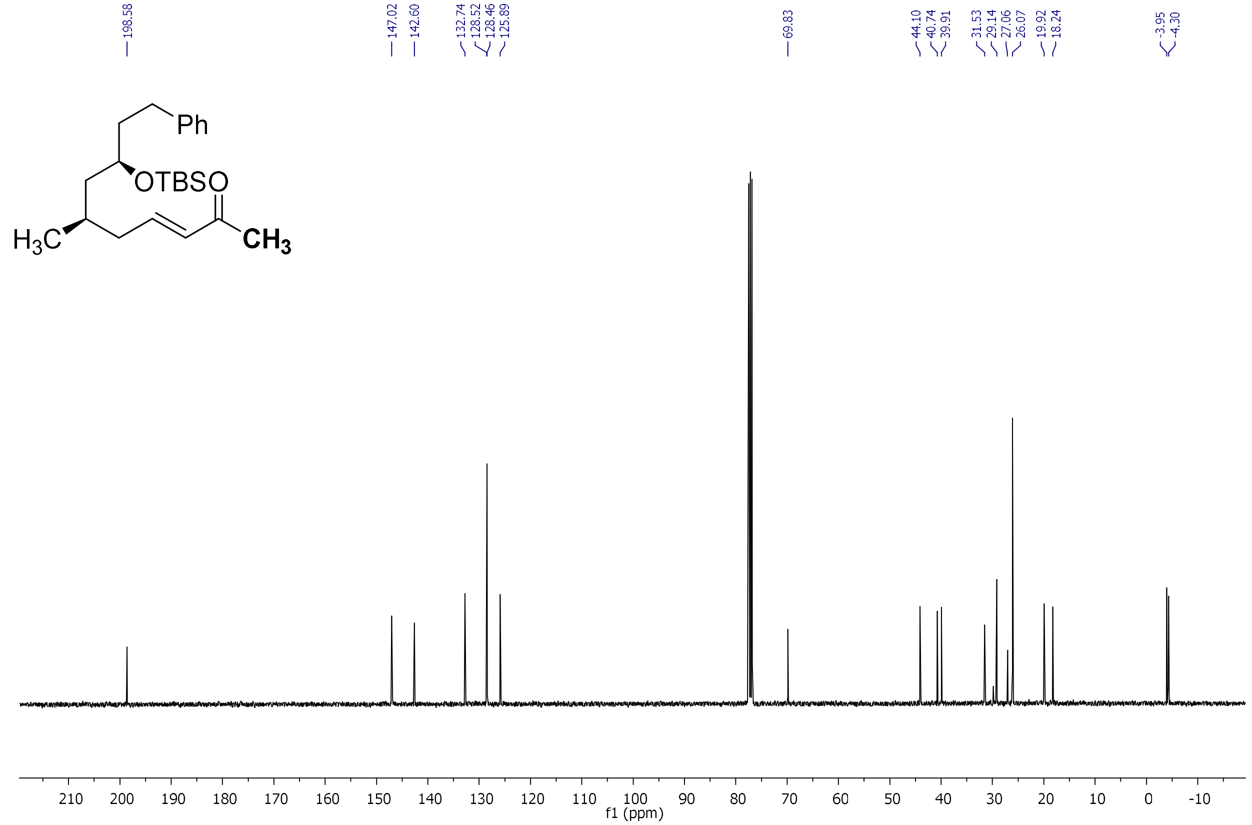

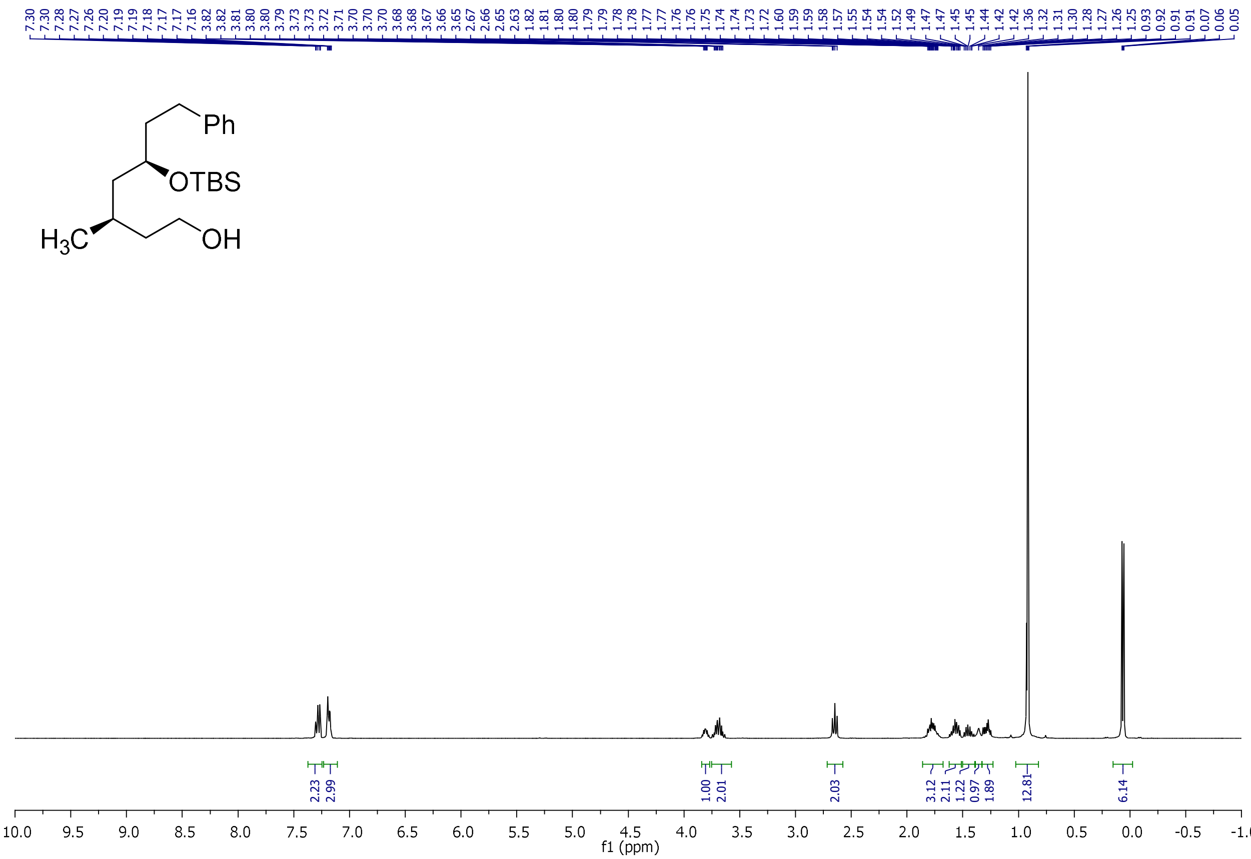
7.30 7.30 7.28 7.27 7.26 7.20 7.19 7.19 7.18 7.17 7.17 7.16 3.82 3.82 3.81 3.80 3.80 3.79 3.73 3.73 3.72 3.71 3.70 3.70 3.68 3.68 3.67 3.66 3.65 2.67 2.66 2.65 2.63 1.82 1.81 1.80 1.79 1.79 1.78 1.78 1.77 1.77 1.76 1.76 1.75 1.74 1.74 1.73 1.72 1.60 1.59 1.59 1.58 1.57 1.55 1.54 1.54 1.52 1.49 1.47 1.45 1.45 1.44 1.42 1.42 1.36 1.32 1.31 1.30 1.28 1.27 1.26 1.25 0.93 0.92 0.92 0.91 0.91 0.07 0.06 0.05
Ph
OTBS
H3C
OH
2.23
2.99
1.00
2.01
2.03
3.12
2.11
1.22
0.97
1.89
12.81
6.14
10.0 9.5 9.0 8.5 8.0 7.5 7.0 6.5 6.0 5.5 5.0 4.5 4.0 3.5 3.0 2.5 2.0 1.5 1.0 0.5 0.0 -0.5 -1.0
f1 (ppm)

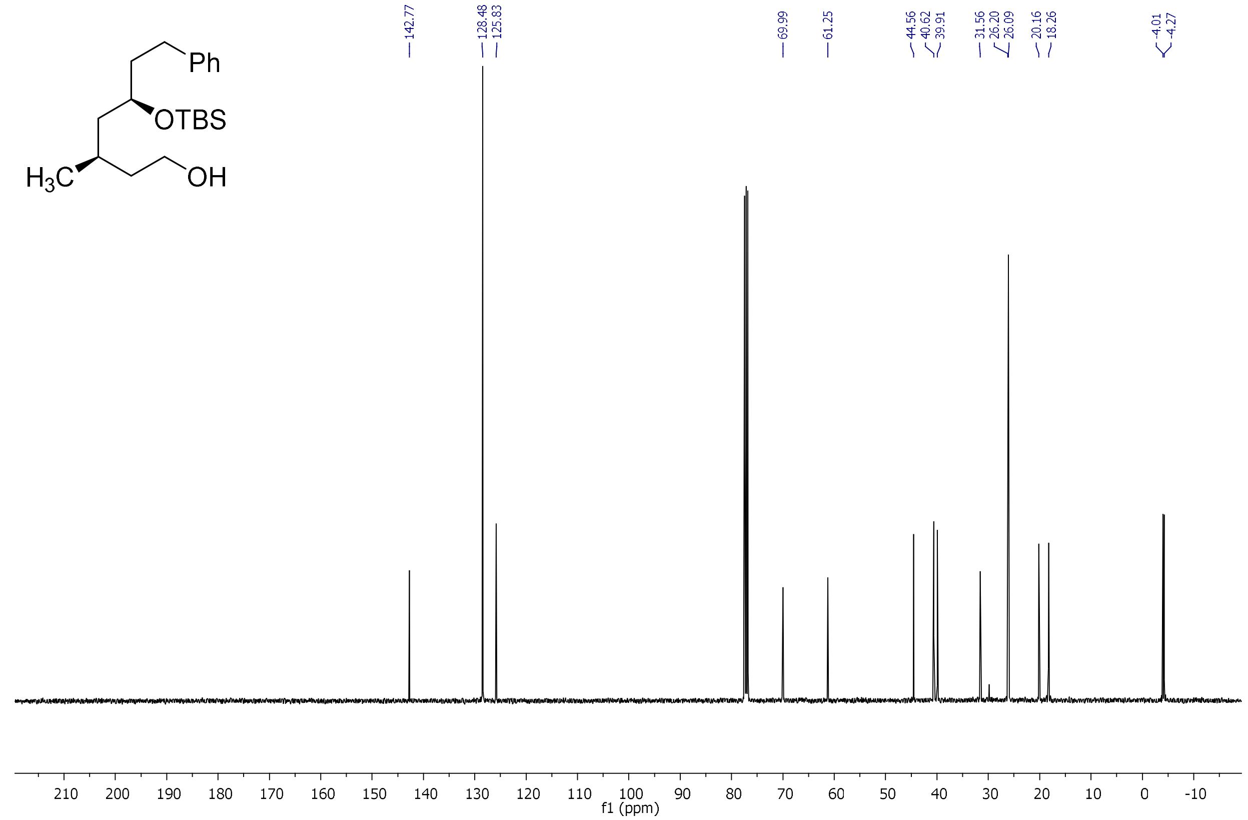
142.77
128.48
125.83
69.99
61.25
44.56
40.62
39.91
31.56
26.20
26.09
20.16
18.26
-4.01
-4.27
Ph
OTBS
H3C
OH
210 200 190 180 170 160 150 140 130 120 110 100 90 80 70 60 50 40 30 20 10 0 -10
f1 (ppm)

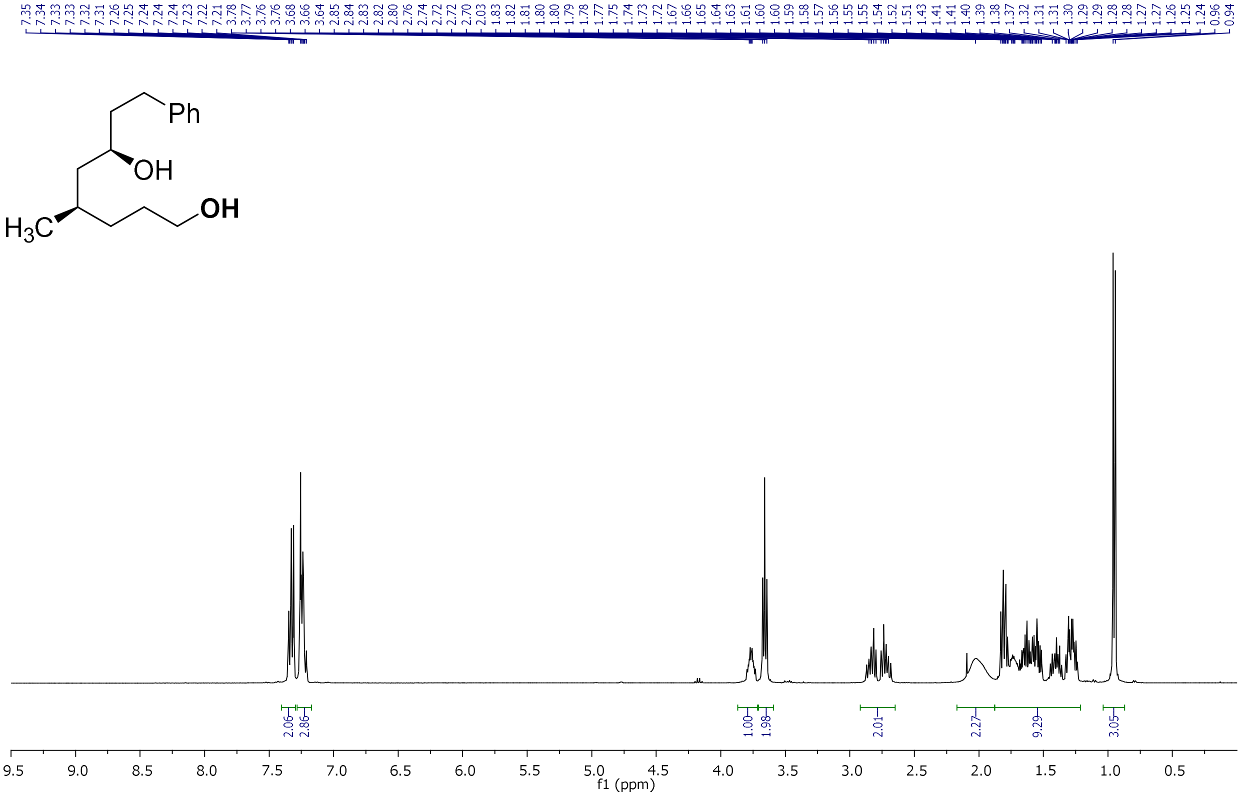
Ph
OH
H3C
OH
2.06
2.86
1.00
1.98
2.01
2.27
9.29
3.05
9.5 9.0 8.5 8.0 7.5 7.0 6.5 6.0 5.5 5.0 4.5 4.0 3.5 3.0 2.5 2.0 1.5 1.0 0.5
f1 (ppm)

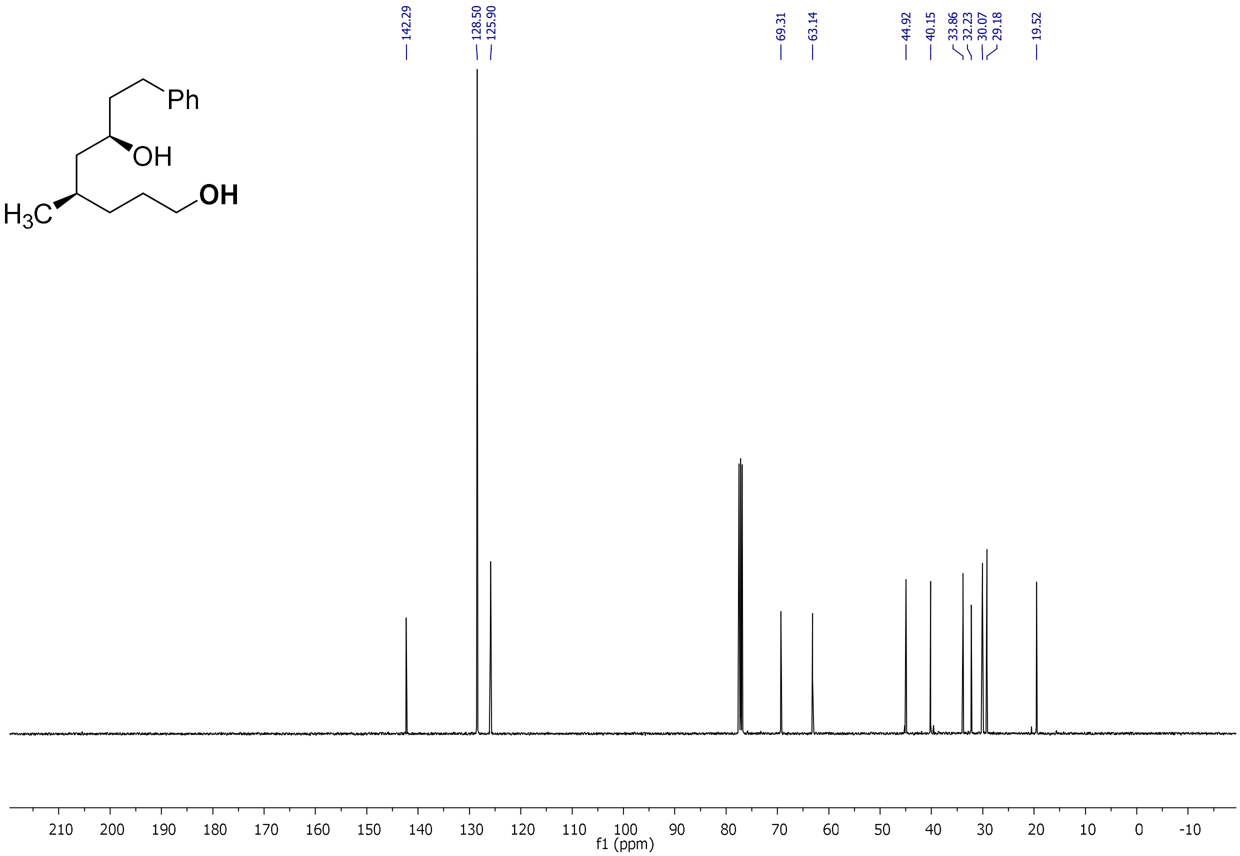
142.29
128.50
125.90
69.31
63.14
44.92
40.15
33.86
32.23
30.07
29.18
19.52
Ph
OH
H3C
OH
210 200 190 180 170 160 150 140 130 120 110 100 90 80 70 60 50 40 30 20 10 0 -10
f1 (ppm)

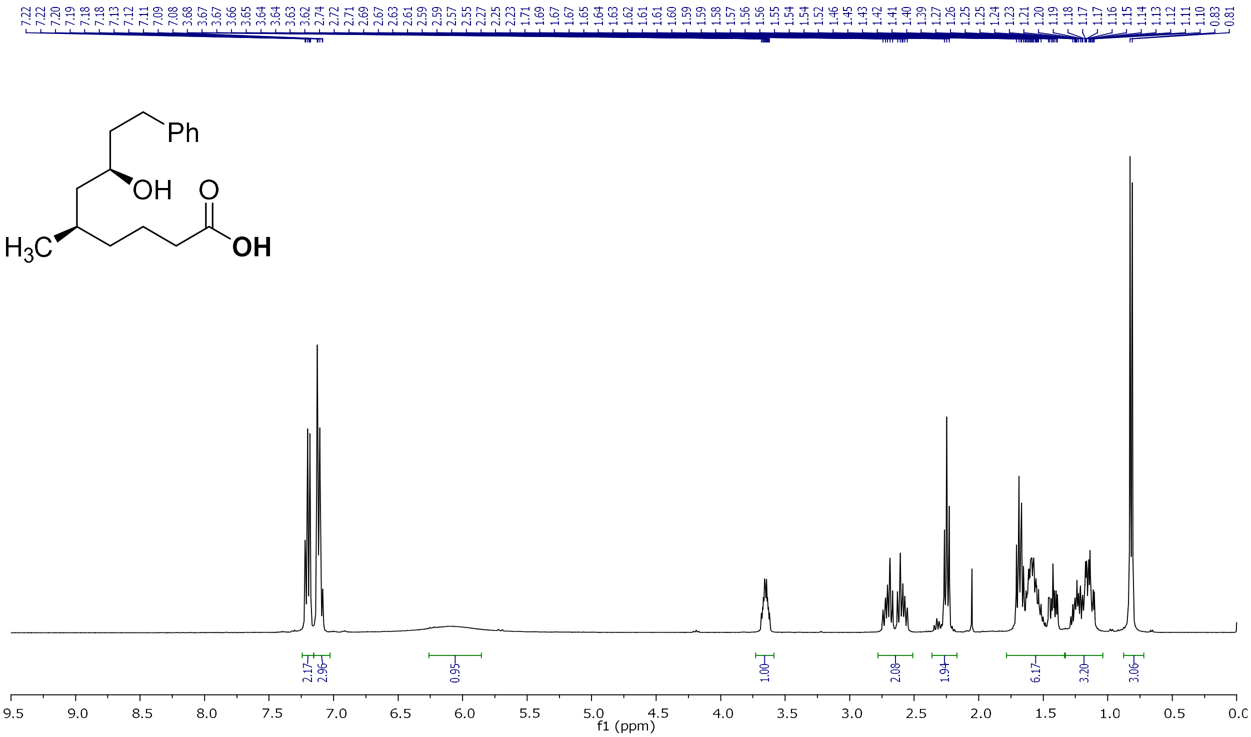
7.22 7.22 7.20 7.19 7.18 7.18 7.13 7.12 7.11 7.09 7.08 3.68 3.67 3.67 3.66 3.65 3.64 3.64 3.63 3.62 2.74 2.72 2.71 2.69 2.67 2.63 2.61 2.59 2.59 2.57 2.55 2.27 2.25 2.23 1.71 1.69 1.67 1.65 1.64 1.63 1.62 1.61 1.61 1.60 1.59 1.59 1.58 1.57 1.56 1.56 1.55 1.54 1.54 1.52 1.46 1.45 1.43 1.42 1.41 1.40 1.39 1.27 1.26 1.25 1.25 1.24 1.23 1.21 1.20 1.19 1.18 1.17 1.17 1.16 1.15 1.14 1.13 1.12 1.11 1.10 0.83 0.81
Ph
OH
O
$H_3C$
OH
2.17
2.96
0.95
1.00
2.08
1.94
6.17
3.20
3.06
9.5 9.0 8.5 8.0 7.5 7.0 6.5 6.0 5.5 5.0 4.5 4.0 3.5 3.0 2.5 2.0 1.5 1.0 0.5 0.0
f1 (ppm)

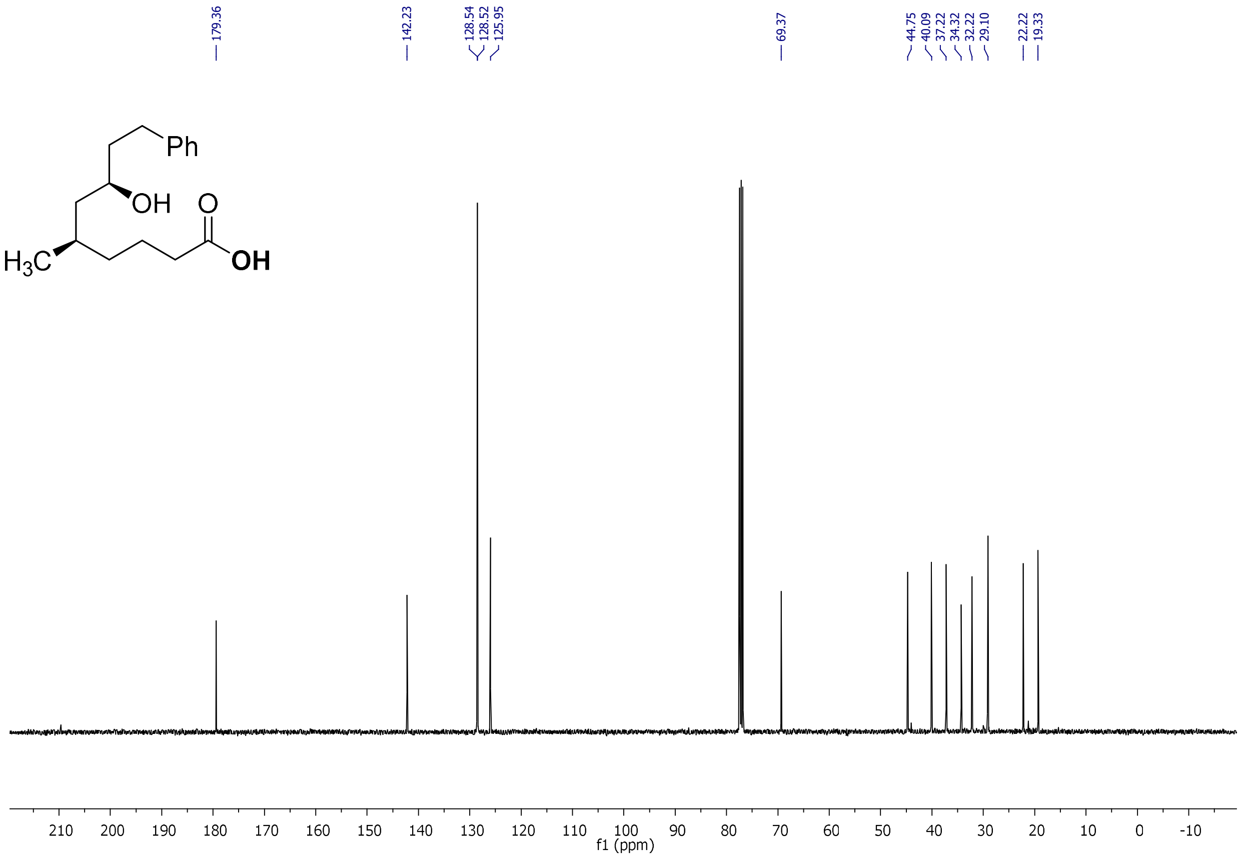
179.36
142.23
128.54 128.52 125.95
69.37
44.75 40.09 37.22 34.32 32.22 29.10
22.22 19.33
Ph
OH
O
$H_3C$
OH
210 200 190 180 170 160 150 140 130 120 110 100 90 80 70 60 50 40 30 20 10 0 -10
f1 (ppm)

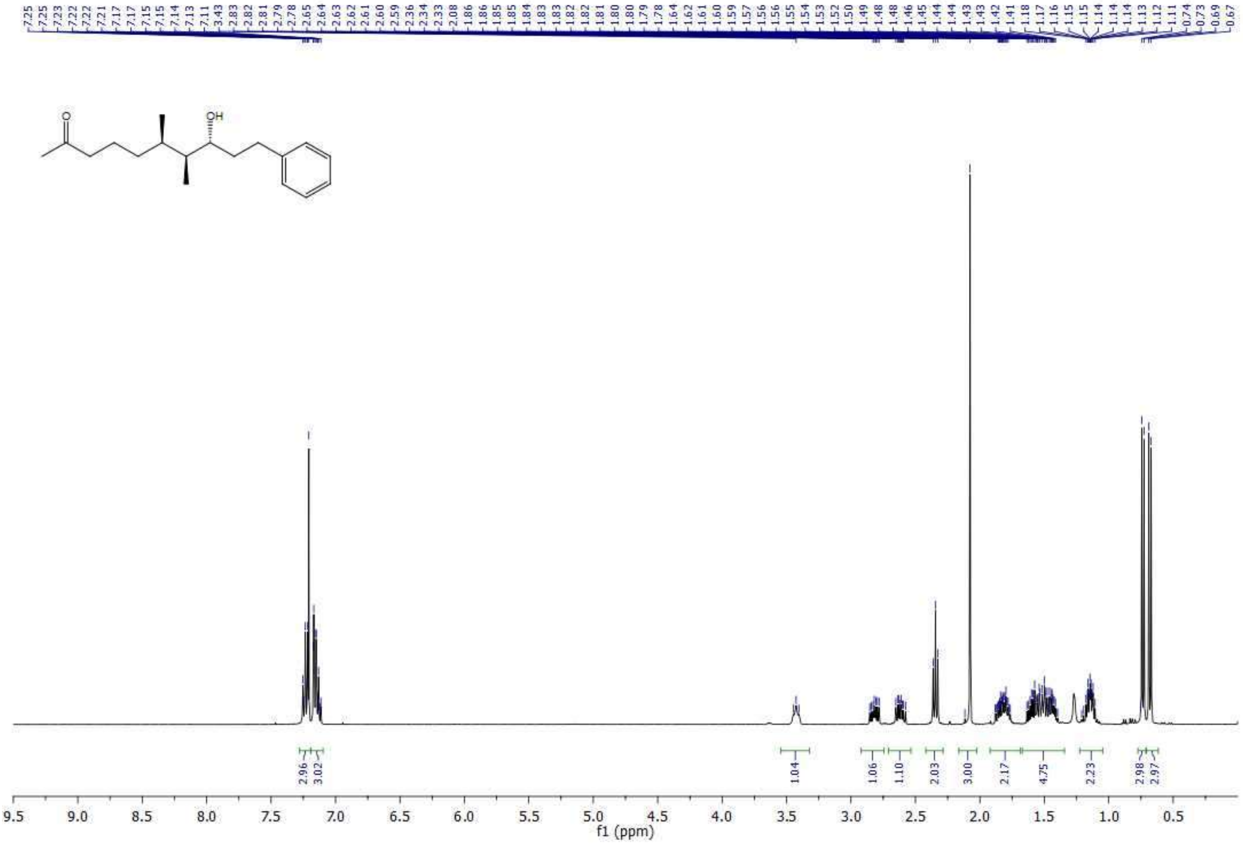
f1 (ppm)

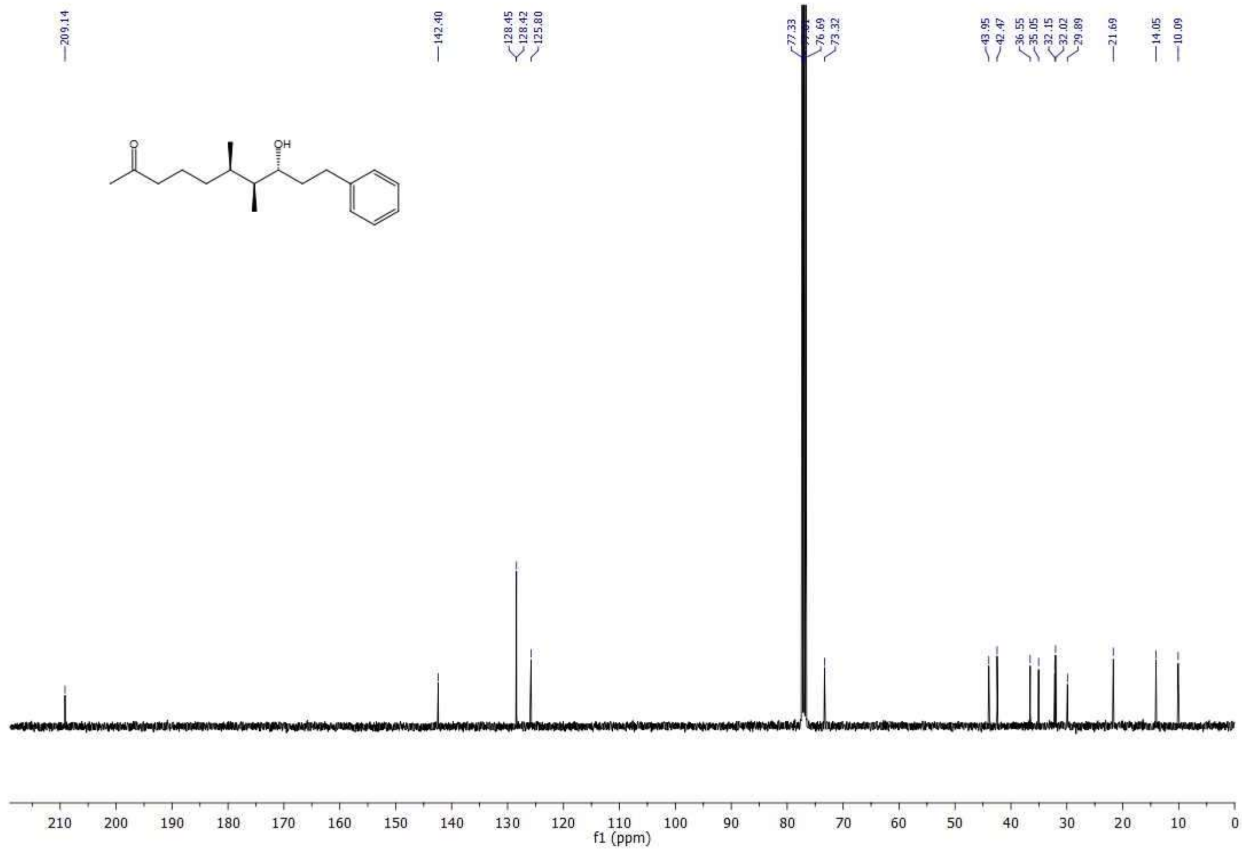
209.14
142.40
128.45
128.42
125.80
77.33
76.69
73.32
43.95
42.47
36.55
35.05
32.15
32.02
29.89
21.69
14.05
10.09
f1 (ppm)

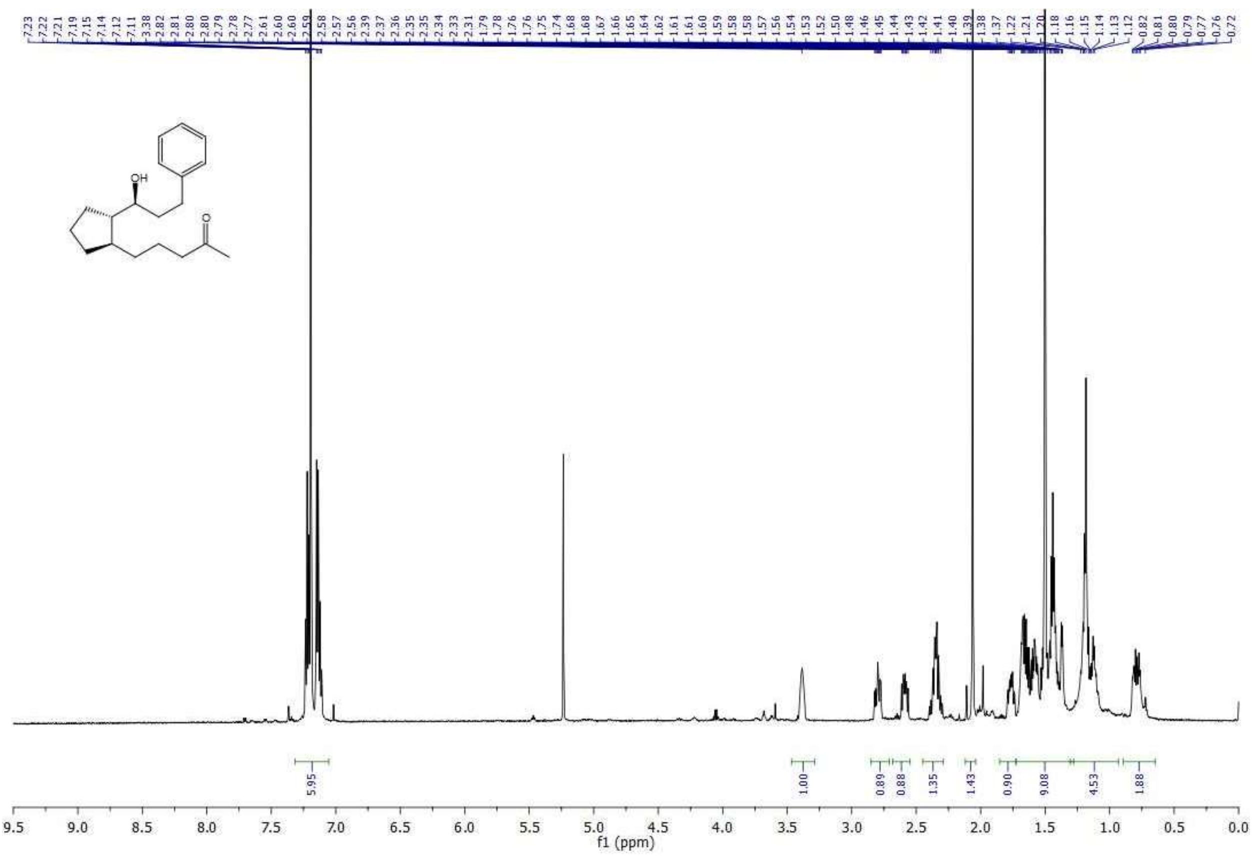
7.23 7.22 7.21 7.19 7.15 7.14 7.12 7.11 3.38 2.82 2.81 2.80 2.79 2.78 2.77 2.61 2.60 2.60 2.59 2.58 2.57 2.56 2.39 2.37 2.36 2.35 2.35 2.34 2.33 2.31 1.79 1.78 1.76 1.76 1.75 1.74 1.68 1.68 1.67 1.66 1.65 1.64 1.62 1.61 1.61 1.60 1.59 1.58 1.58 1.57 1.56 1.54 1.53 1.52 1.50 1.48 1.46 1.45 1.44 1.43 1.42 1.41 1.40 1.39 1.38 1.37 1.22 1.21 1.20 1.18 1.16 1.15 1.14 1.13 1.12 0.82 0.81 0.80 0.79 0.77 0.76 0.72
OH
O
5.95
1.00
0.89
0.88
1.35
1.43
0.90
9.08
4.53
1.88
9.5 9.0 8.5 8.0 7.5 7.0 6.5 6.0 5.5 5.0 4.5 4.0 3.5 3.0 2.5 2.0 1.5 1.0 0.5 0.0
f1 (ppm)

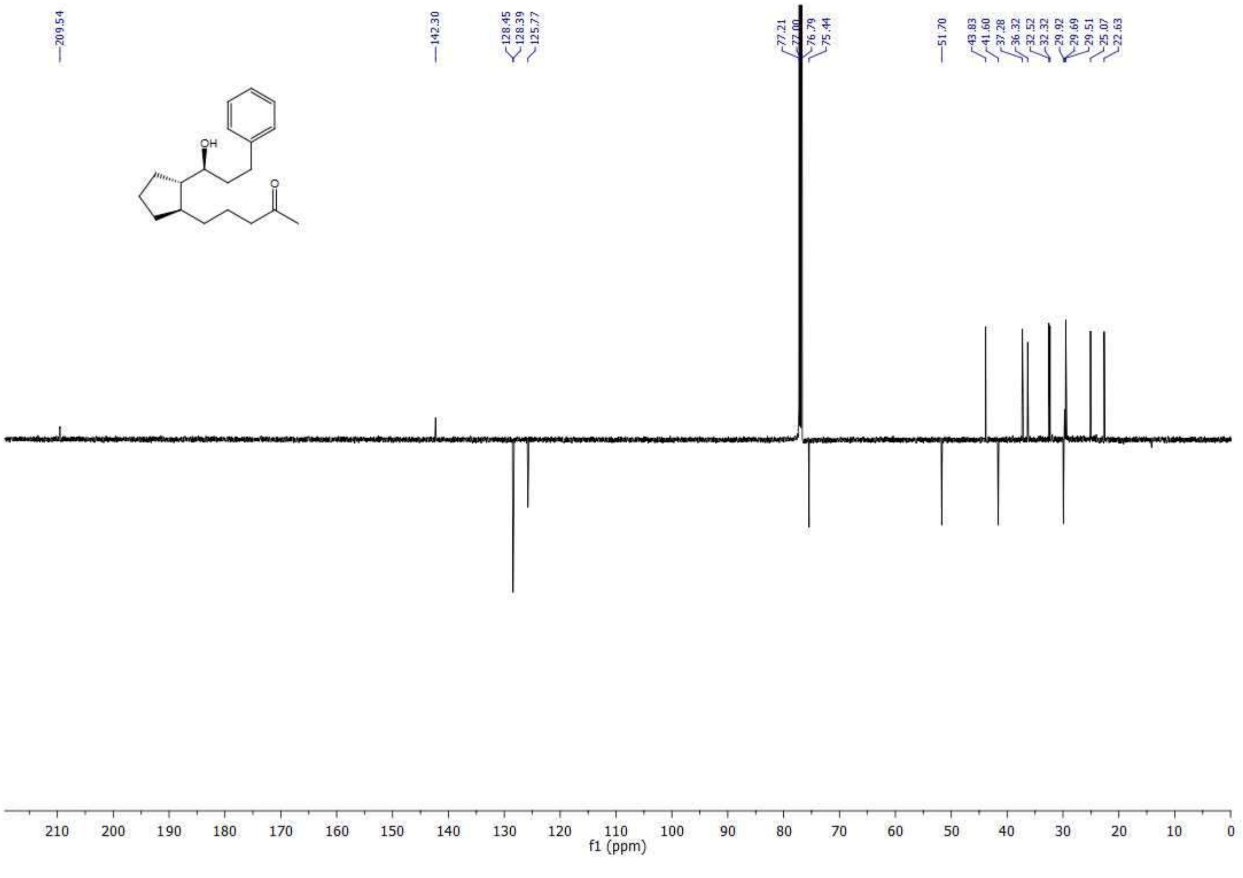
209.54
142.30
128.45 128.39 125.77
77.21 77.00 76.79 75.44
51.70
43.83 41.60 37.28 36.32 32.52 32.32 29.92 29.69 29.51 25.07 22.63
OH
O
210 200 190 180 170 160 150 140 130 120 110 100 90 80 70 60 50 40 30 20 10 0
f1 (ppm)

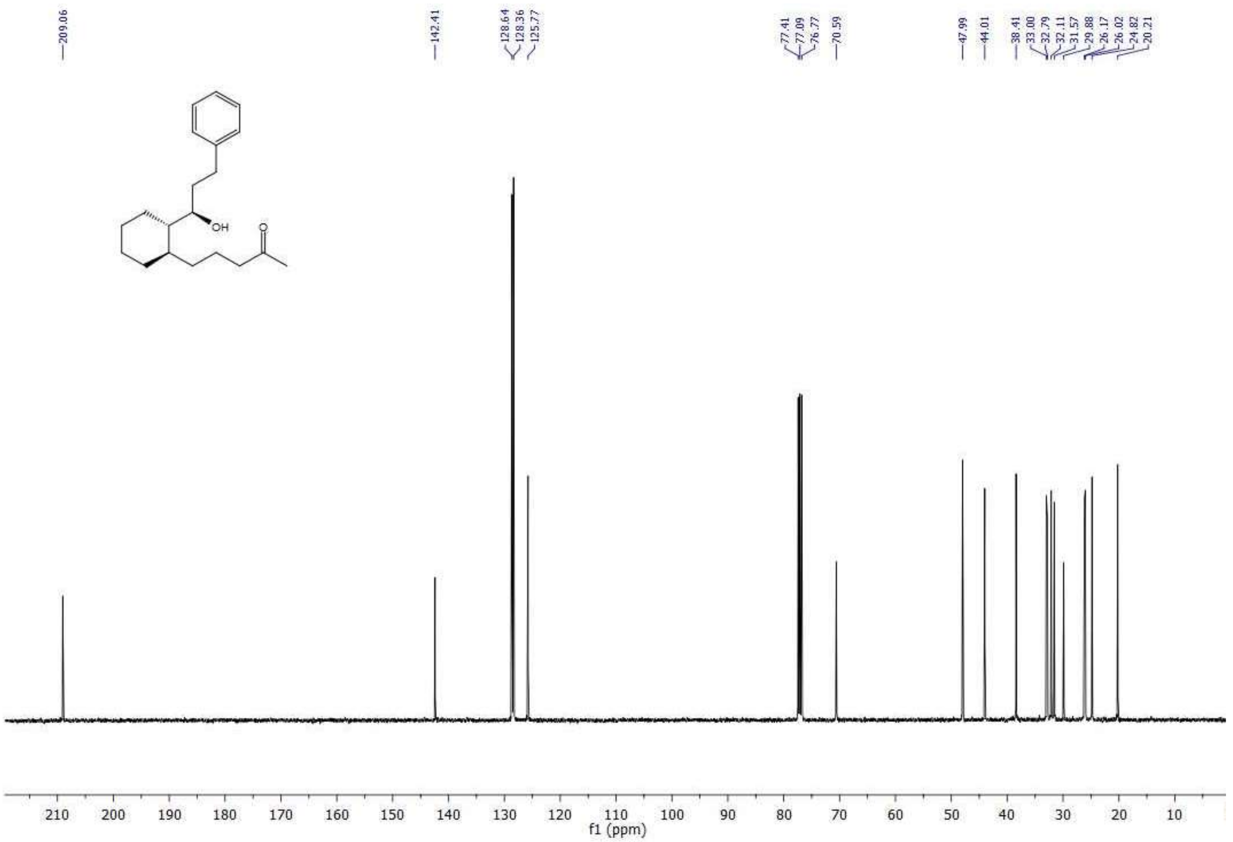
209.06
142.41
128.64
128.36
125.77
77.41
77.09
76.77
70.59
47.99
44.01
38.41
33.00
32.79
32.11
31.57
29.88
26.17
26.02
24.82
20.21
OH
O
210 200 190 180 170 160 150 140 130 120 110 100 90 80 70 60 50 40 30 20 10
f1 (ppm)

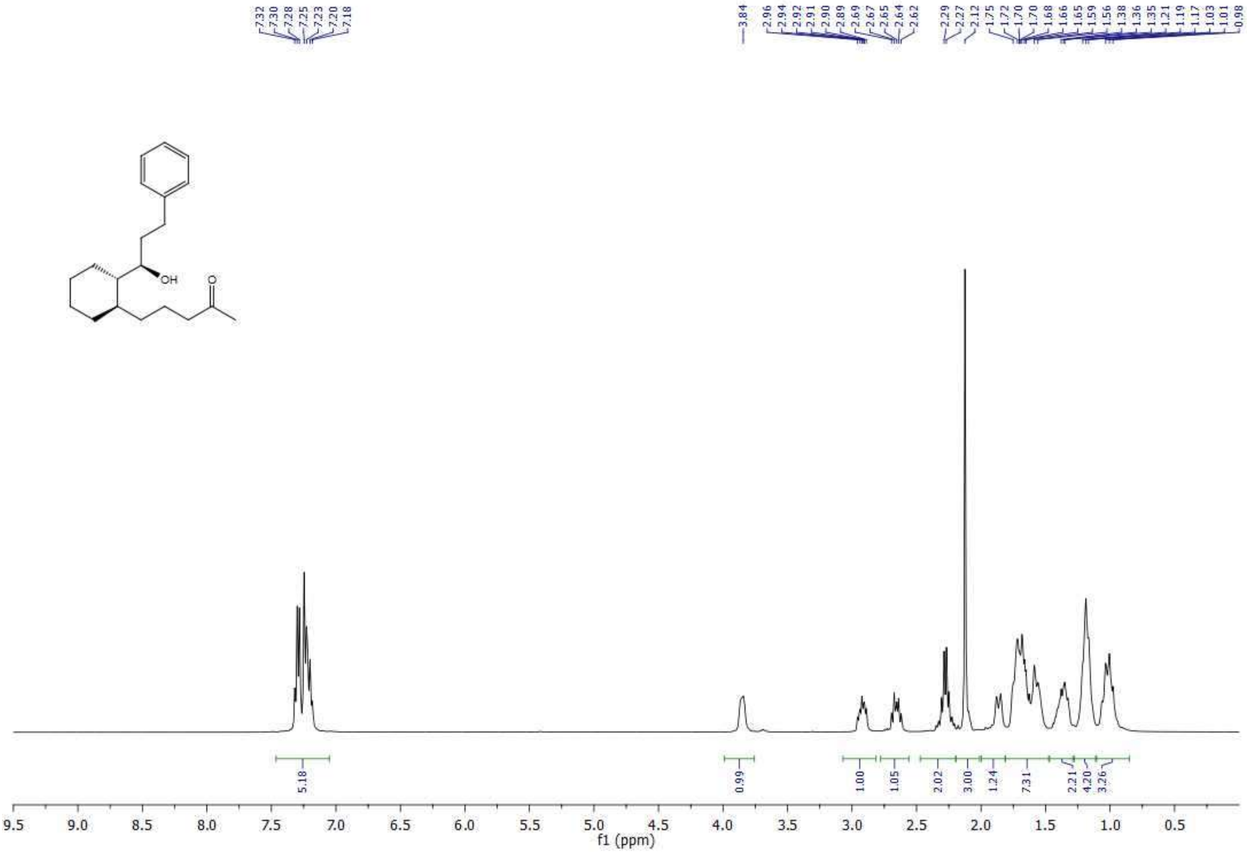
7.32
7.30
7.28
7.25
7.23
7.20
7.18
3.84
2.96
2.94
2.92
2.91
2.90
2.89
2.69
2.67
2.65
2.64
2.62
2.29
2.27
2.12
1.75
1.72
1.70
1.68
1.66
1.65
1.59
1.56
1.38
1.36
1.35
1.21
1.19
1.17
1.03
1.01
0.98
OH
O
5.18
0.99
1.00
1.05
2.02
3.00
1.24
7.31
2.21
4.20
3.26
9.5 9.0 8.5 8.0 7.5 7.0 6.5 6.0 5.5 5.0 4.5 4.0 3.5 3.0 2.5 2.0 1.5 1.0 0.5
f1 (ppm)

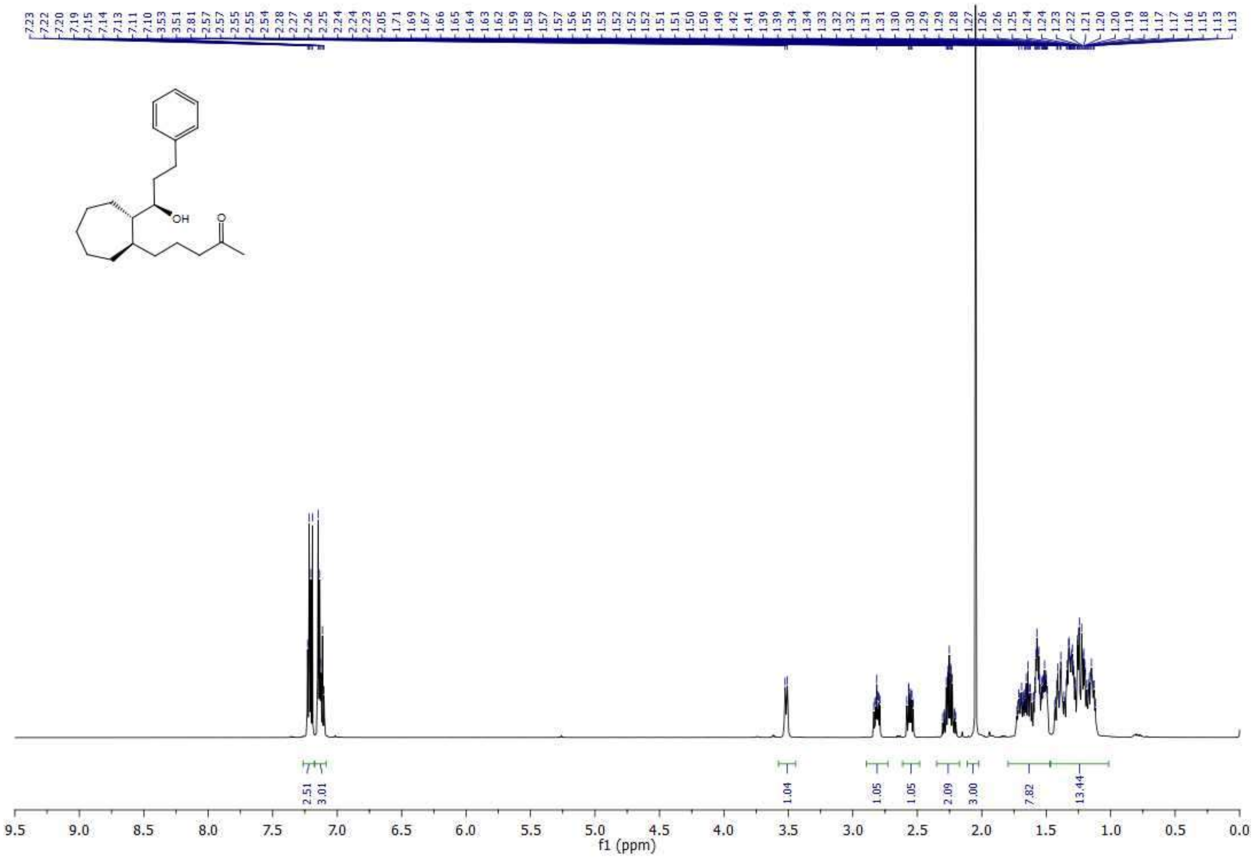

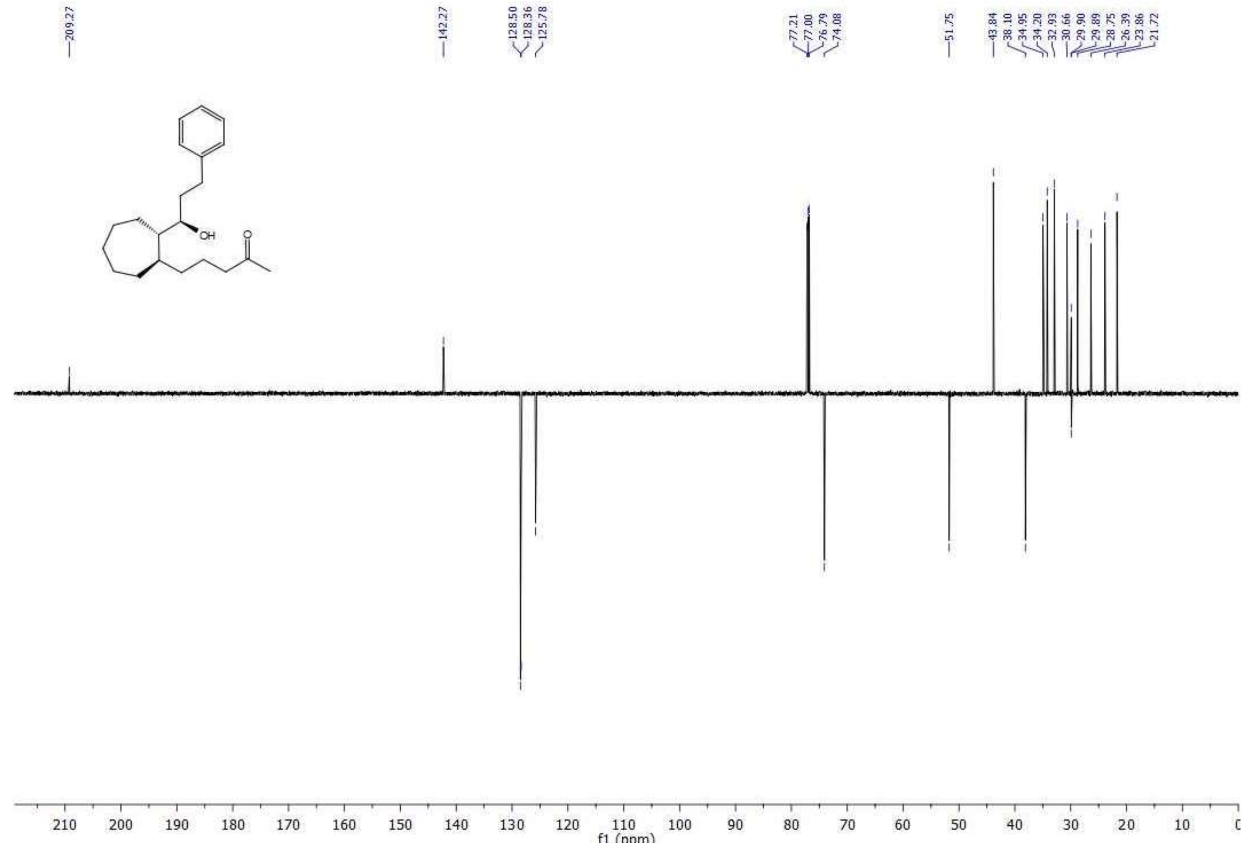

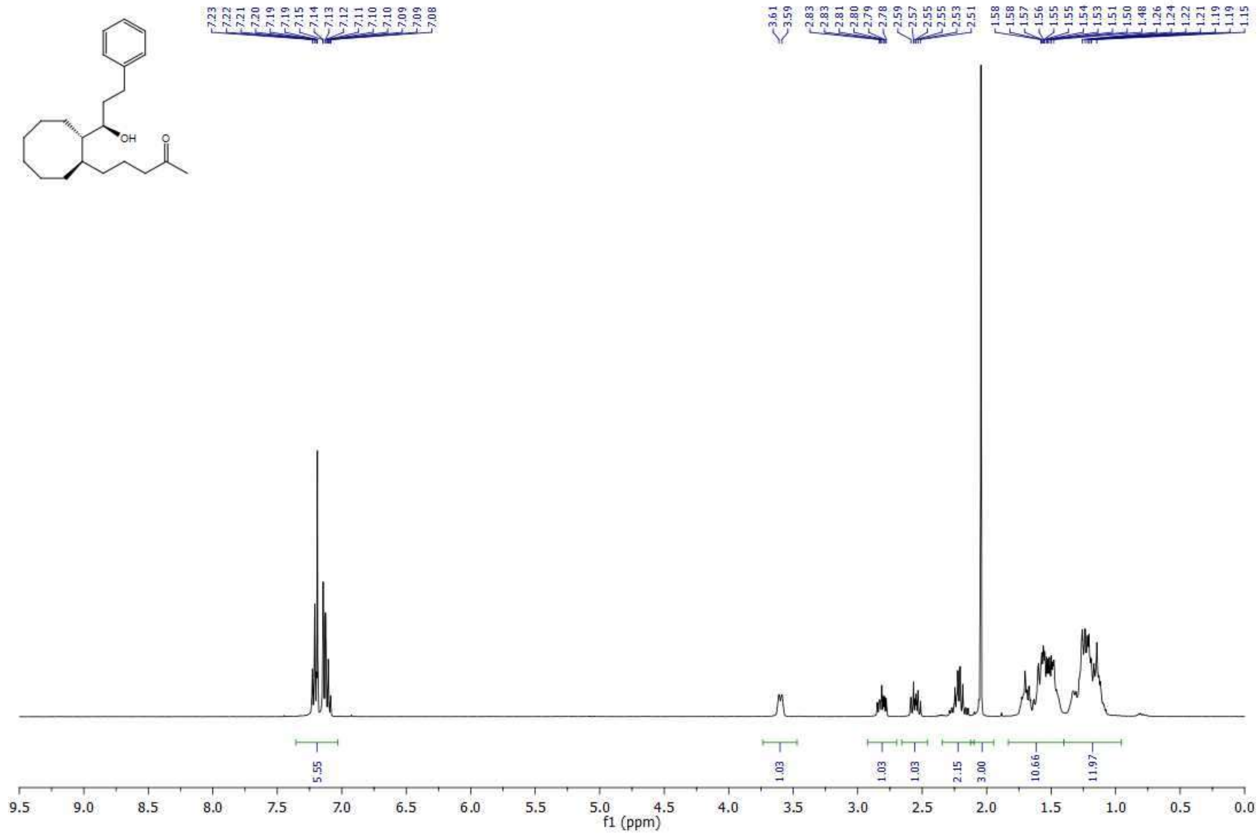
7.23 7.22 7.21 7.20 7.19 7.19 7.15 7.14 7.13 7.12 7.11 7.10 7.10 7.09 7.09 7.08
3.61 3.59
2.83 2.83 2.81 2.80 2.79 2.78 2.59 2.57 2.55 2.55 2.53 2.51
1.58 1.58 1.57 1.56 1.55 1.55 1.55 1.54 1.53 1.51 1.50 1.48 1.26 1.24 1.22 1.21 1.19 1.19 1.15
OH
O
5.55
1.03
1.03
1.03
2.15
3.00
10.66
11.97
9.5 9.0 8.5 8.0 7.5 7.0 6.5 6.0 5.5 5.0 4.5 4.0 3.5 3.0 2.5 2.0 1.5 1.0 0.5 0.0
f1 (ppm)
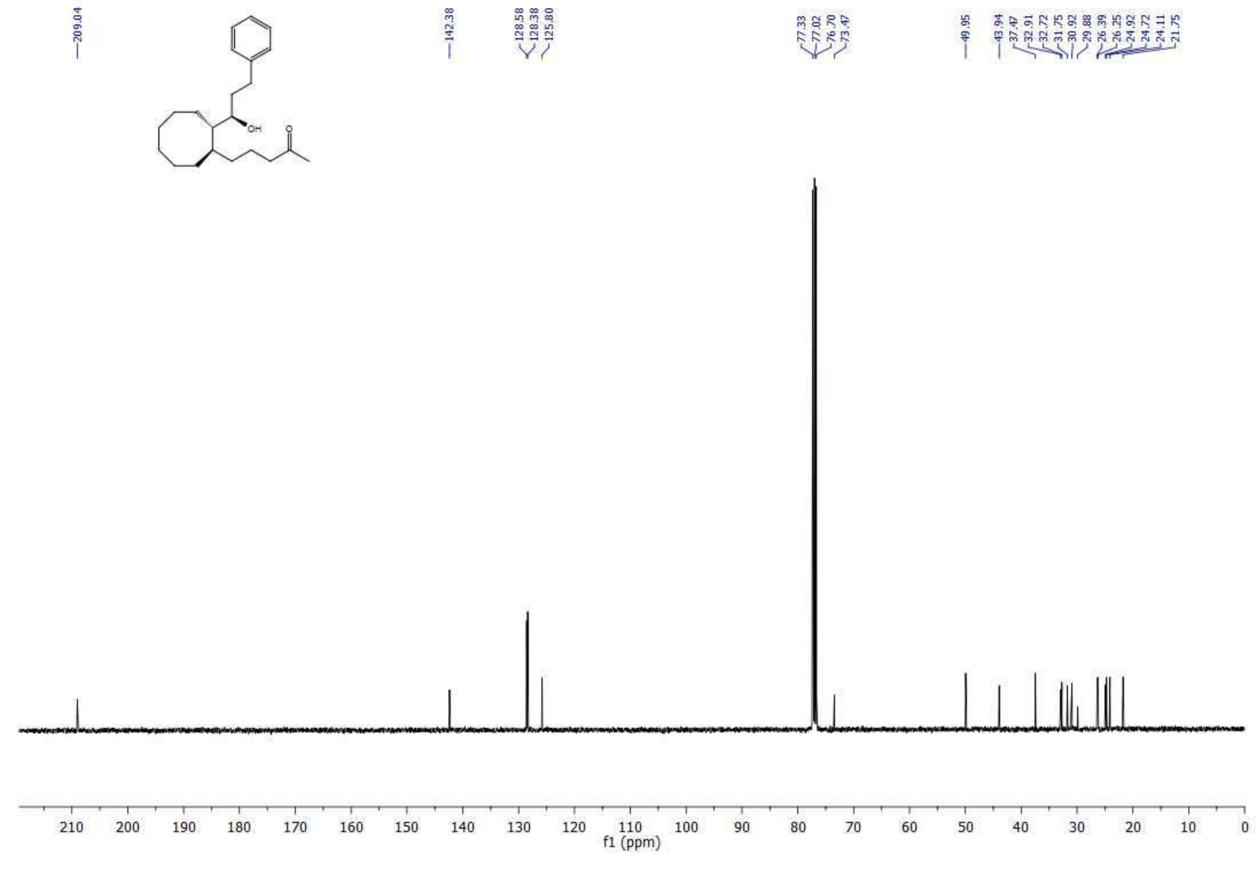
209.04
142.38
128.58 128.38 125.80
77.33 77.02 76.70 73.47
49.95
43.94 37.47 32.91 32.72 31.75 30.92 29.88 26.39 26.25 24.92 24.72 24.11 21.75
OH
O
210 200 190 180 170 160 150 140 130 120 110 100 90 80 70 60 50 40 30 20 10 0
f1 (ppm)

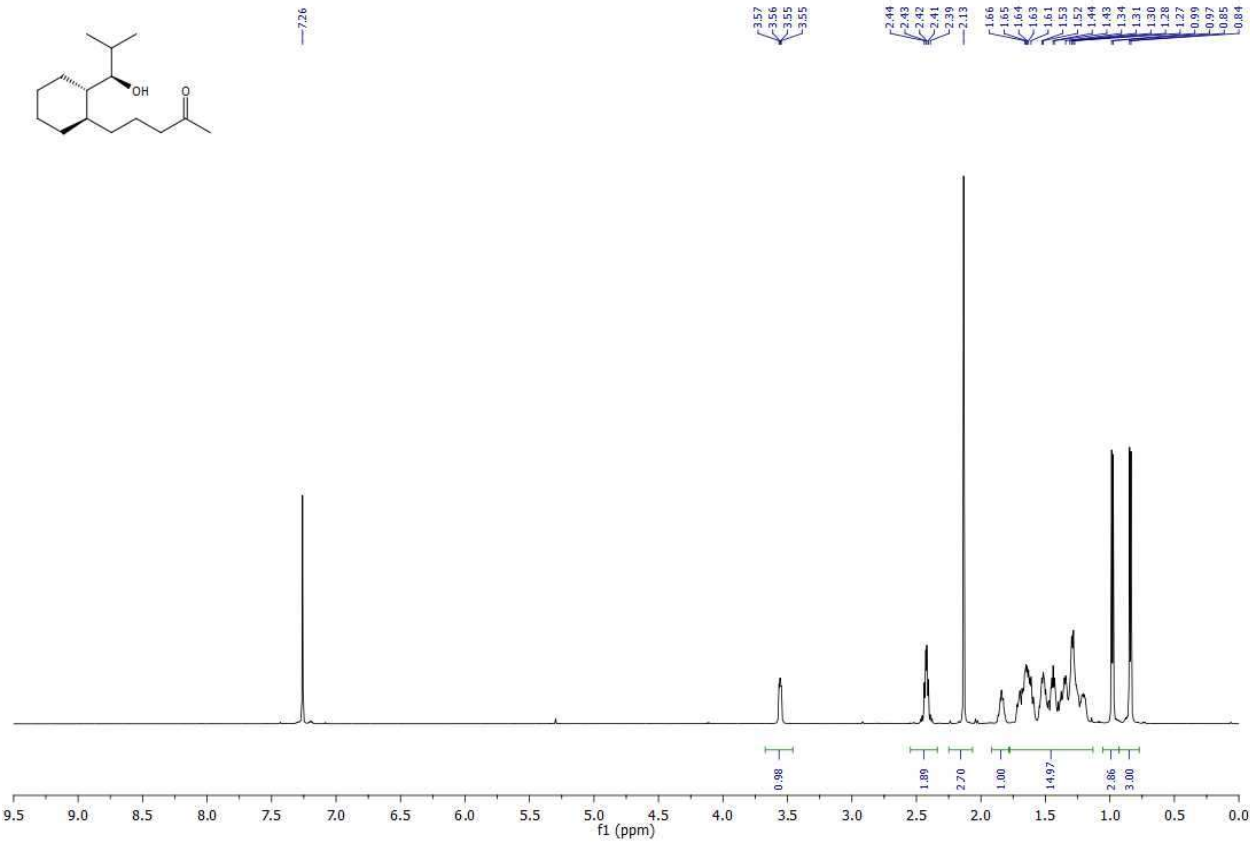
7.26
3.57
3.56
3.55
3.55
2.44
2.43
2.42
2.41
2.39
2.13
1.66
1.65
1.64
1.63
1.61
1.53
1.52
1.44
1.43
1.34
1.31
1.30
1.28
1.27
0.99
0.97
0.85
0.84
OH
O
0.98
1.89
2.70
1.00
14.97
2.86
3.00
f1 (ppm)

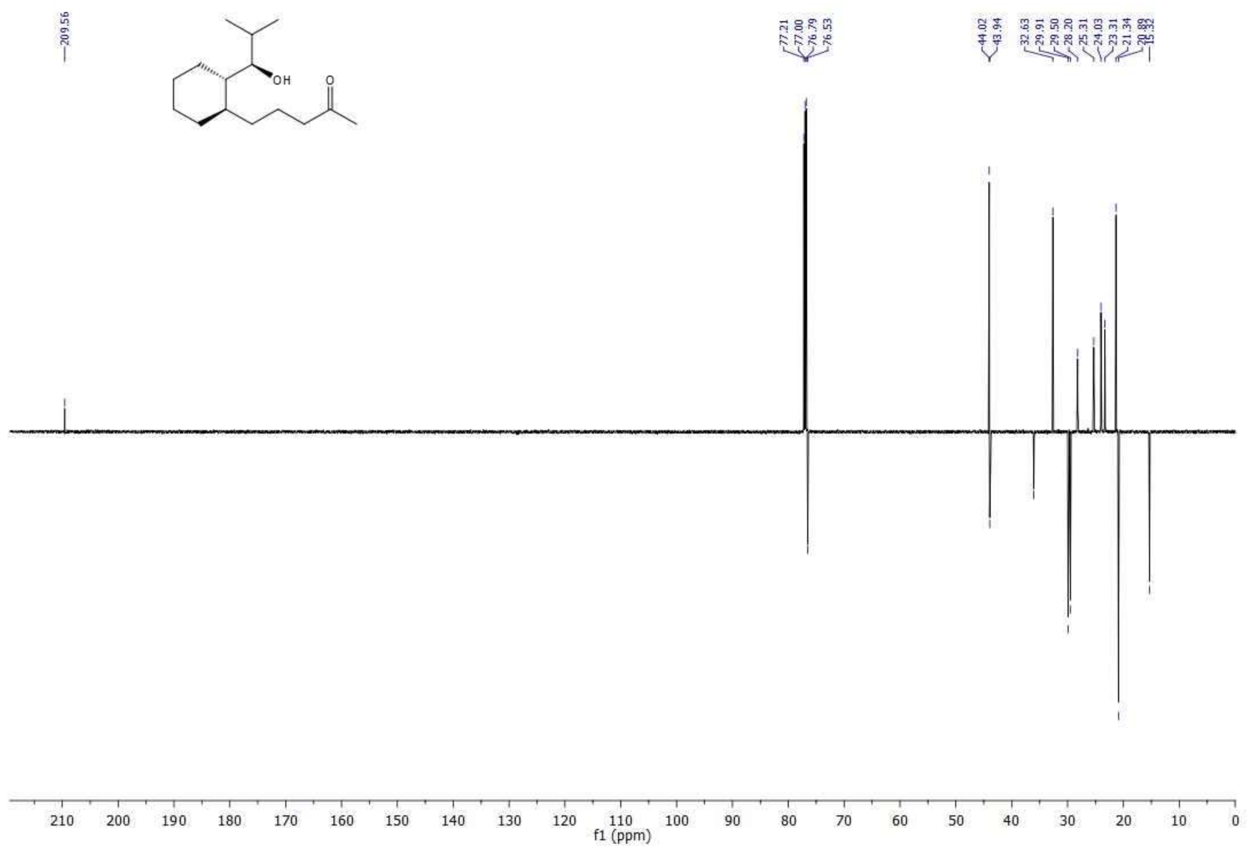
209.56
77.21
77.00
76.79
76.53
44.02
43.94
32.63
29.91
29.50
28.20
25.31
24.03
23.31
21.34
OH
O
f1 (ppm)

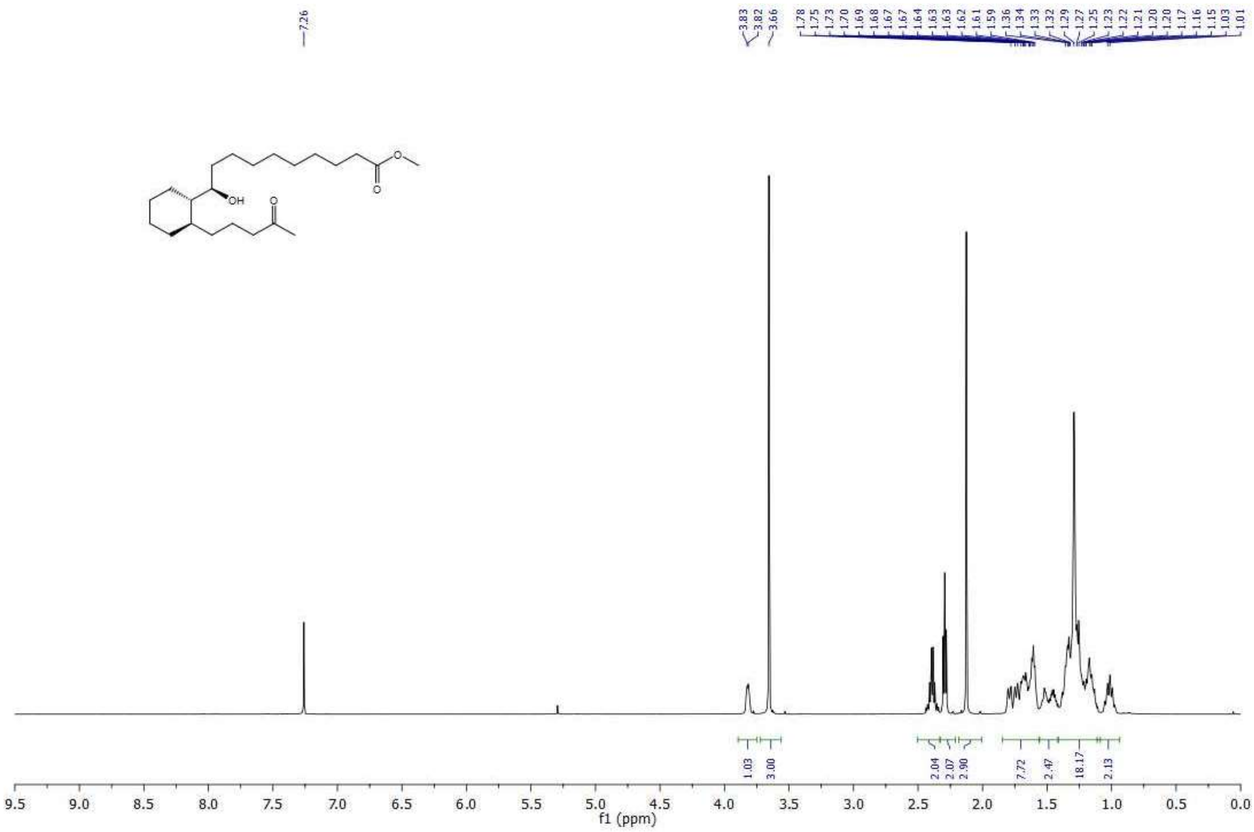

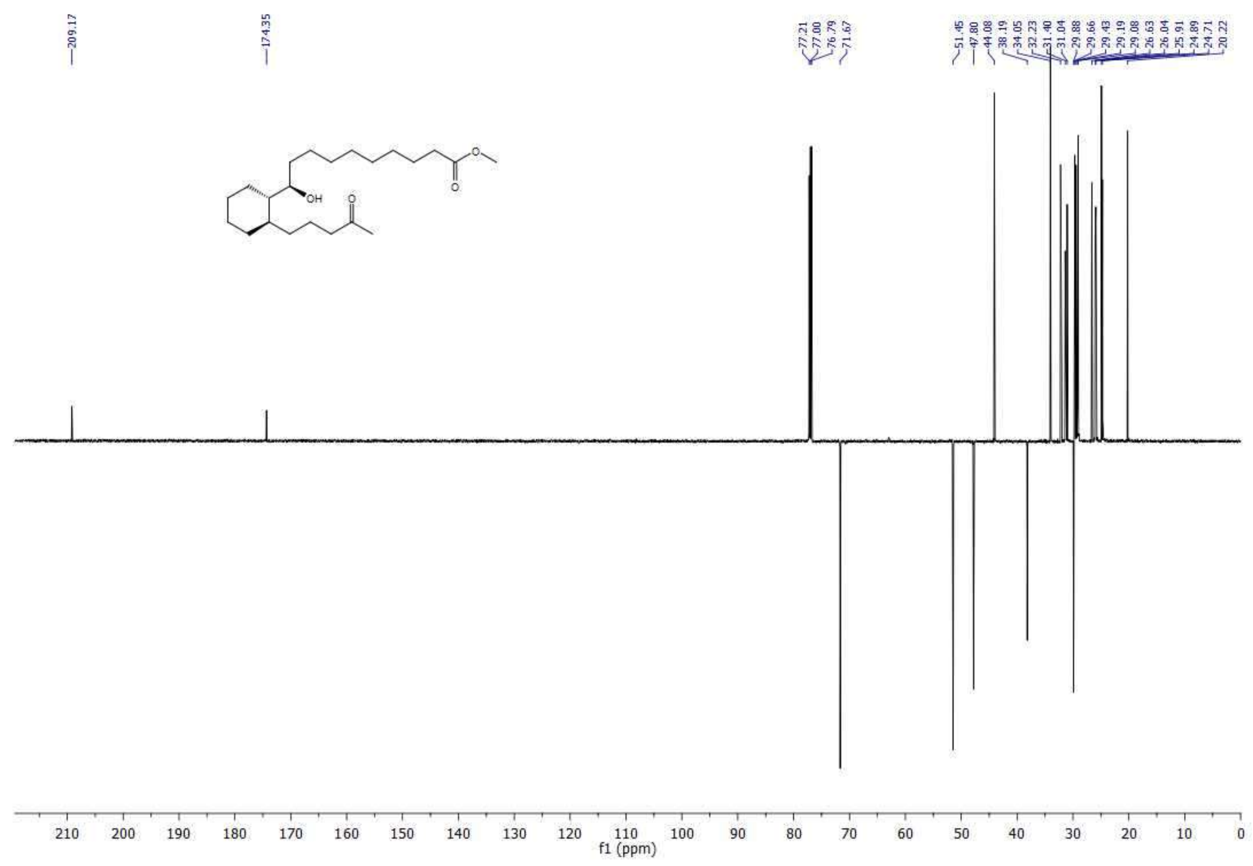

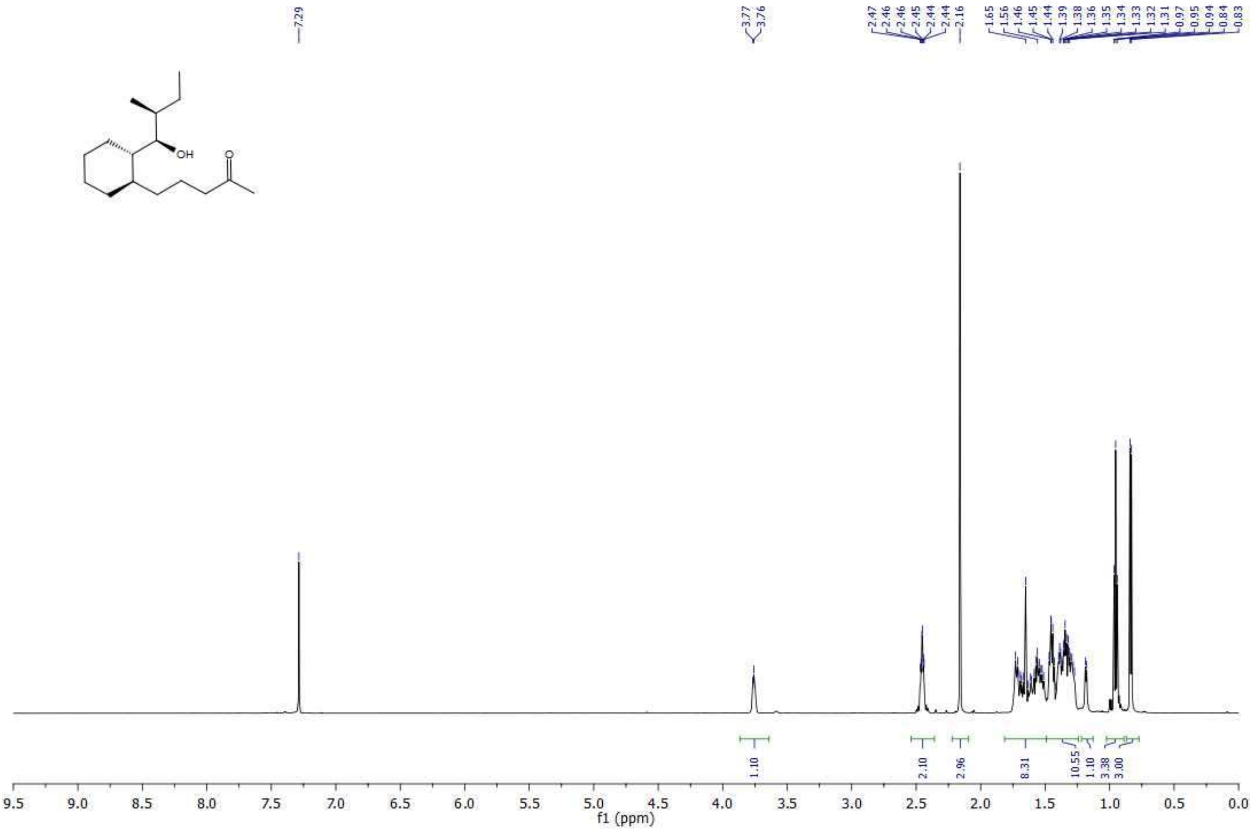
7.29
3.77
3.76
2.47
2.46
2.46
2.45
2.44
2.44
2.16
1.65
1.56
1.46
1.45
1.44
1.39
1.38
1.36
1.35
1.34
1.33
1.32
1.31
0.97
0.95
0.94
0.84
0.83
OH
O
1.10
2.10
2.96
8.31
10.55
1.10
3.38
3.00
f1 (ppm)

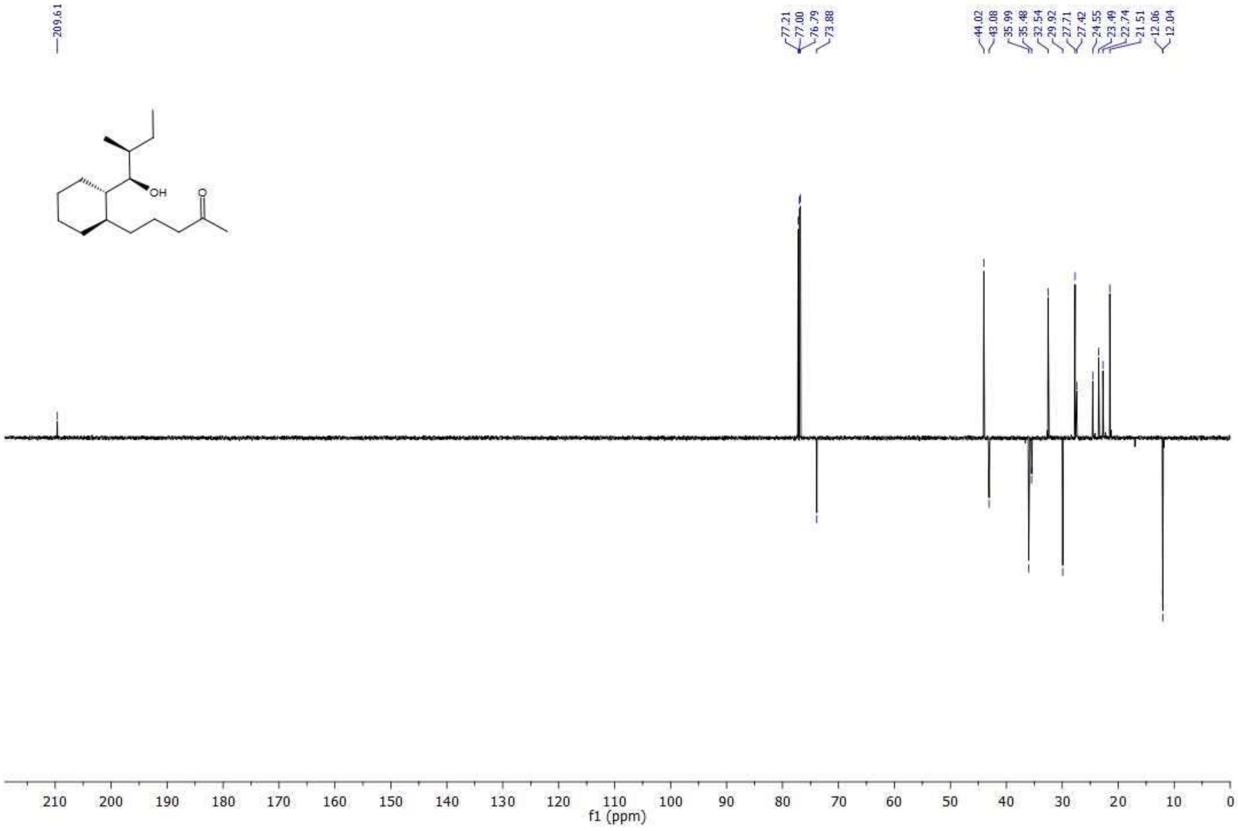
209.61
77.21
77.00
76.79
73.88
44.02
43.08
35.99
35.48
32.54
29.92
27.71
27.42
24.55
23.49
22.74
21.51
12.06
12.04
OH
O
f1 (ppm)

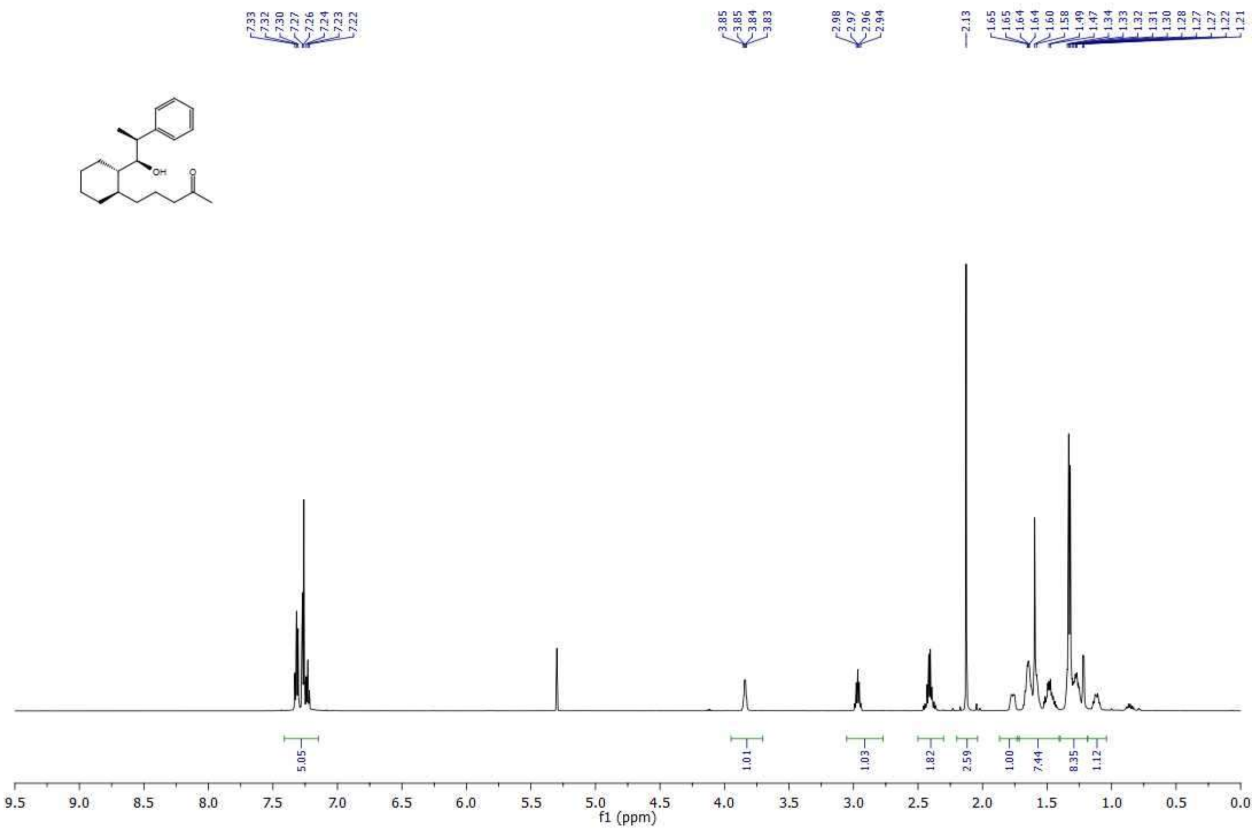

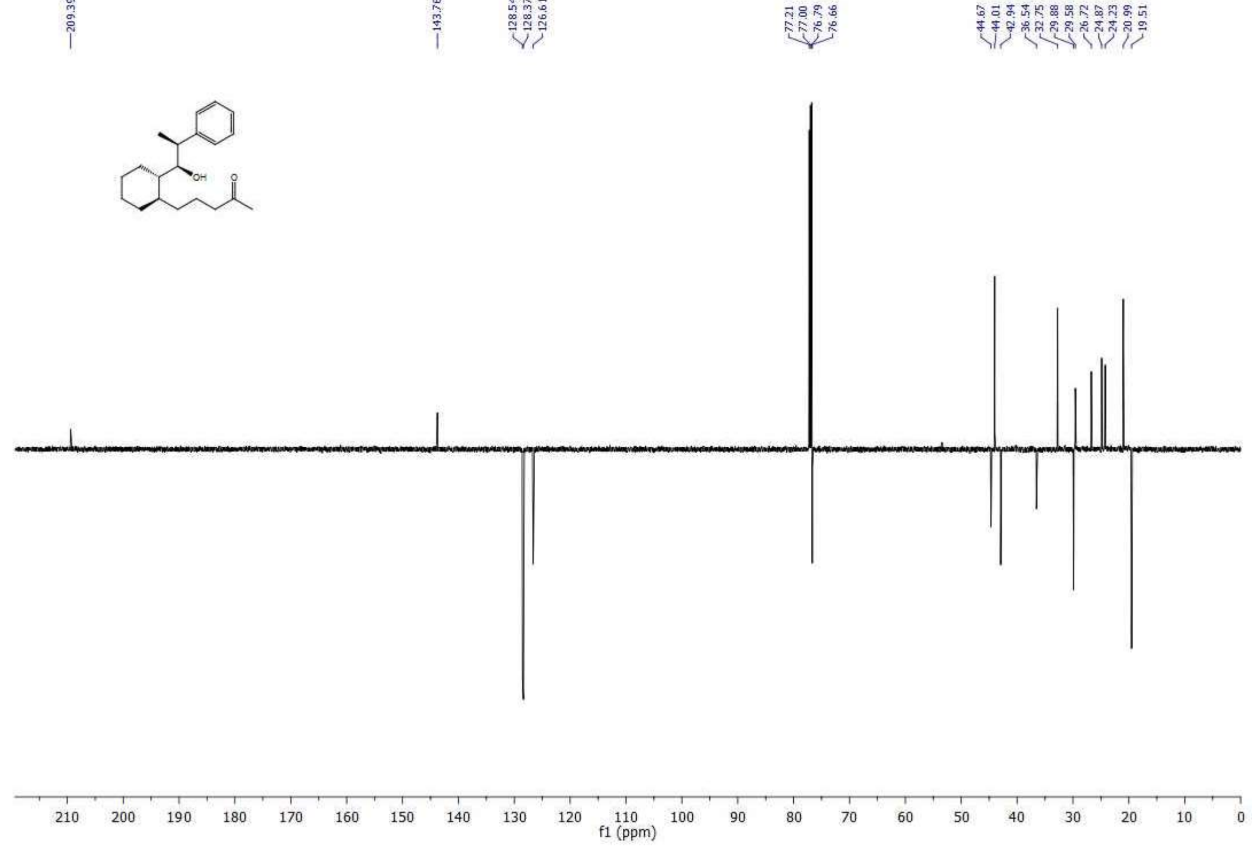

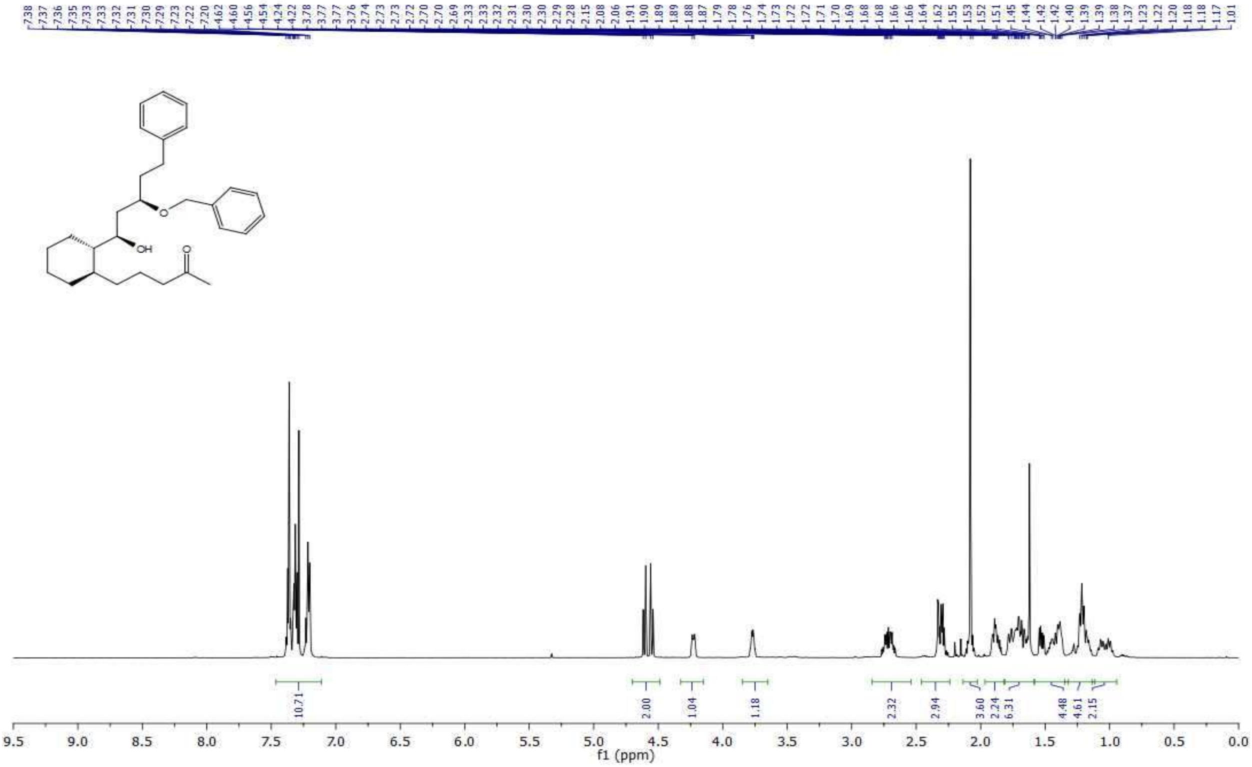

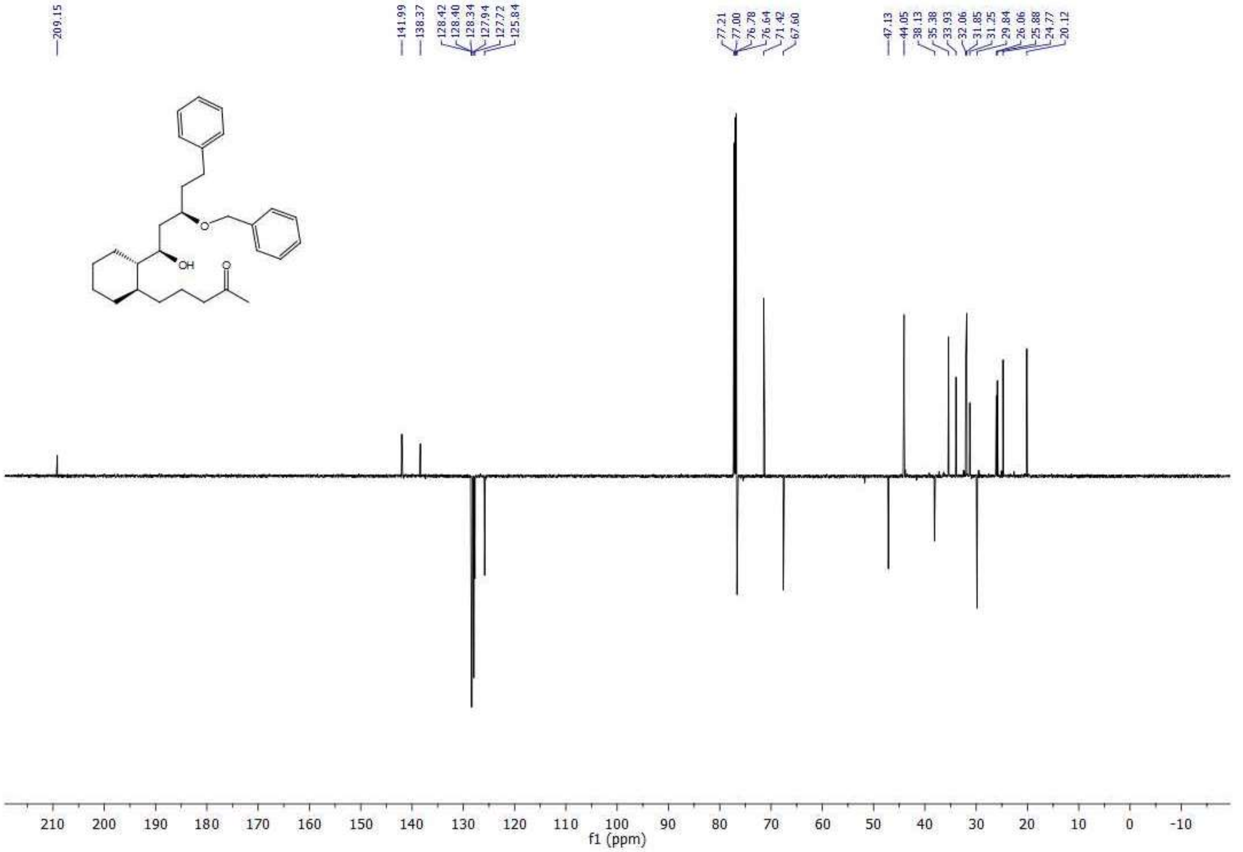

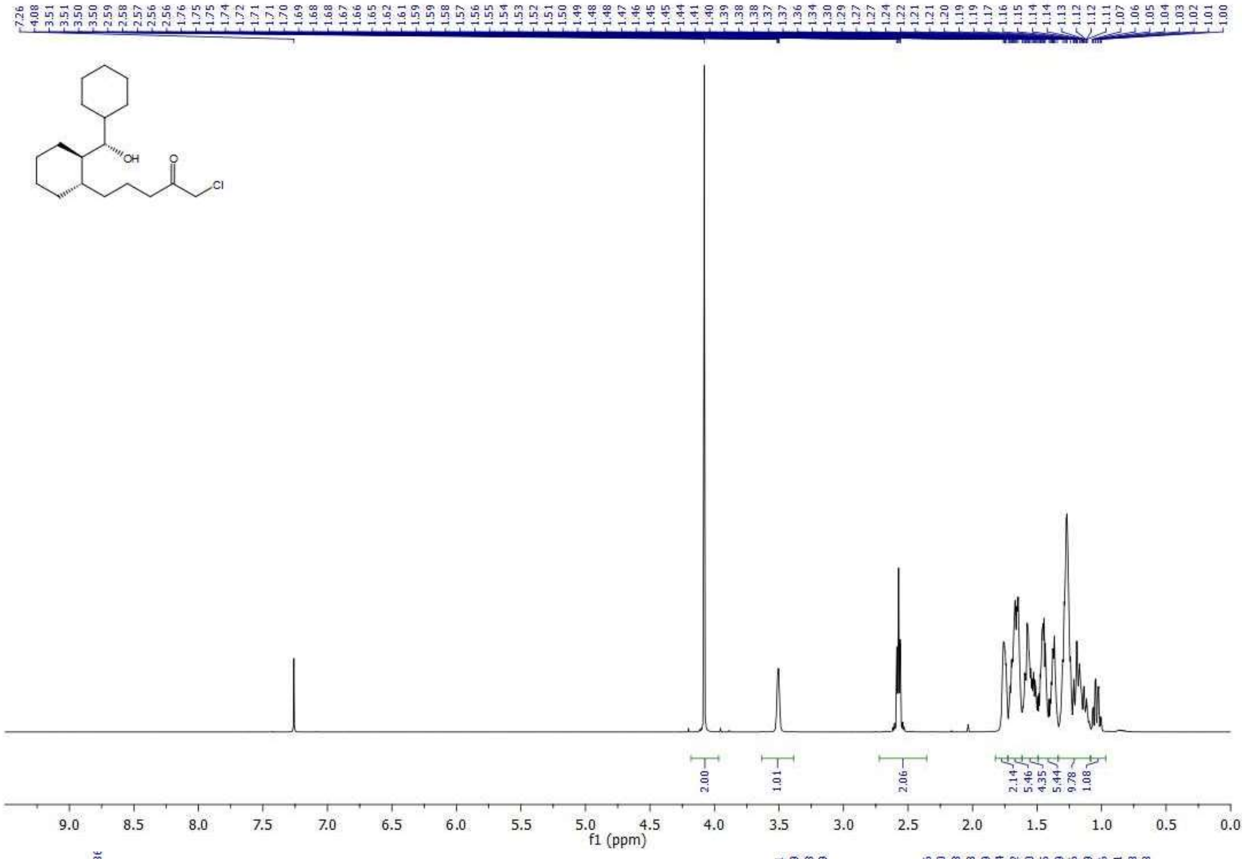
7.26
4.08
3.51
3.51
3.50
3.50
2.59
2.58
2.57
2.56
2.56
1.76
1.75
1.75
1.74
1.72
1.71
1.71
1.70
1.69
1.68
1.68
1.67
1.66
1.65
1.62
1.61
1.59
1.59
1.58
1.57
1.56
1.55
1.54
1.53
1.52
1.51
1.50
1.49
1.48
1.48
1.47
1.46
1.45
1.45
1.44
1.41
1.40
1.39
1.38
1.38
1.37
1.37
1.36
1.34
1.30
1.29
1.27
1.27
1.24
1.22
1.21
1.21
1.20
1.19
1.19
1.17
1.16
1.15
1.14
1.14
1.13
1.12
1.12
1.11
1.07
1.06
1.05
1.04
1.03
1.02
1.01
1.00
OH
O
Cl
2.00
1.01
2.06
2.14
5.46
4.35
5.44
9.78
1.08
9.0 8.5 8.0 7.5 7.0 6.5 6.0 5.5 5.0 4.5 4.0 3.5 3.0 2.5 2.0 1.5 1.0 0.5 0.0
f1 (ppm)

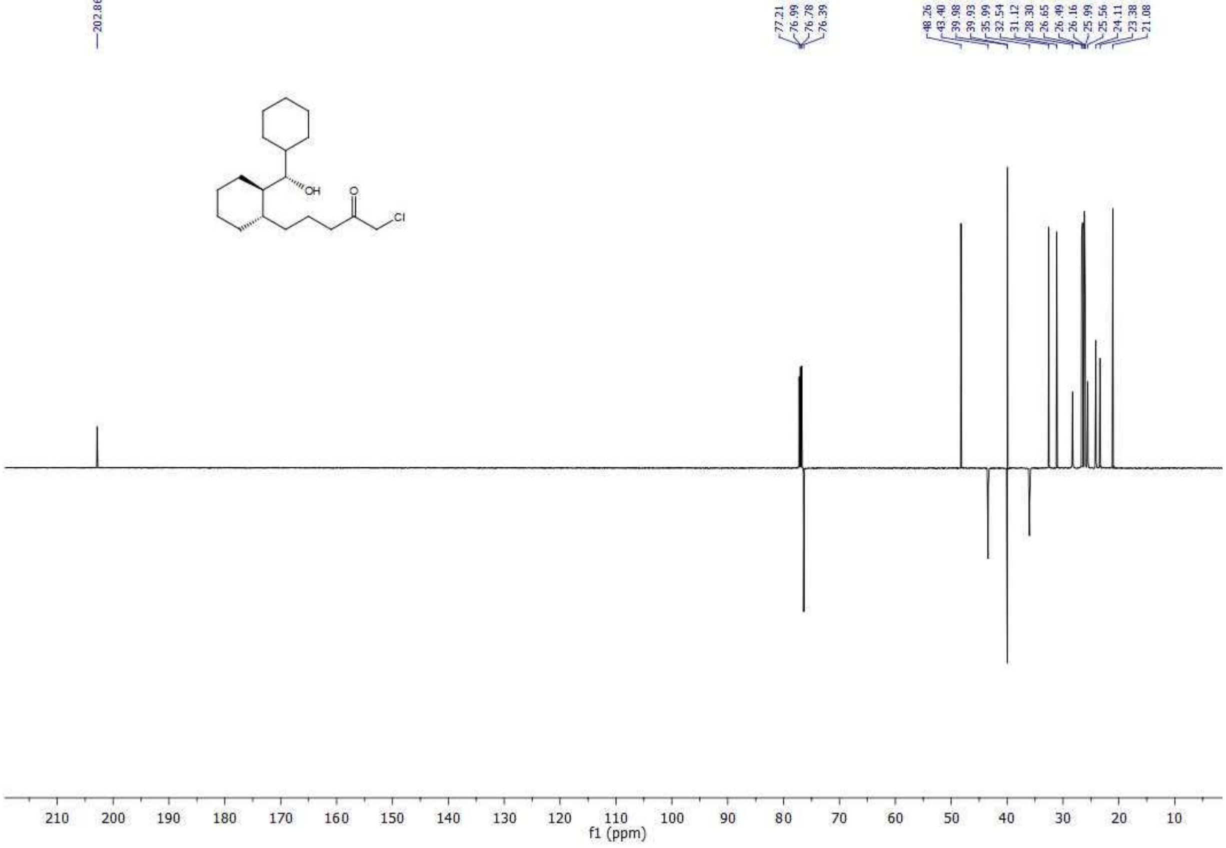
202.8
77.21
76.99
76.78
76.39
48.26
43.40
39.98
39.93
35.99
32.54
31.12
28.30
26.65
26.49
26.16
25.99
25.56
24.11
23.38
21.08
OH
O
Cl
210 200 190 180 170 160 150 140 130 120 110 100 90 80 70 60 50 40 30 20 10
f1 (ppm)

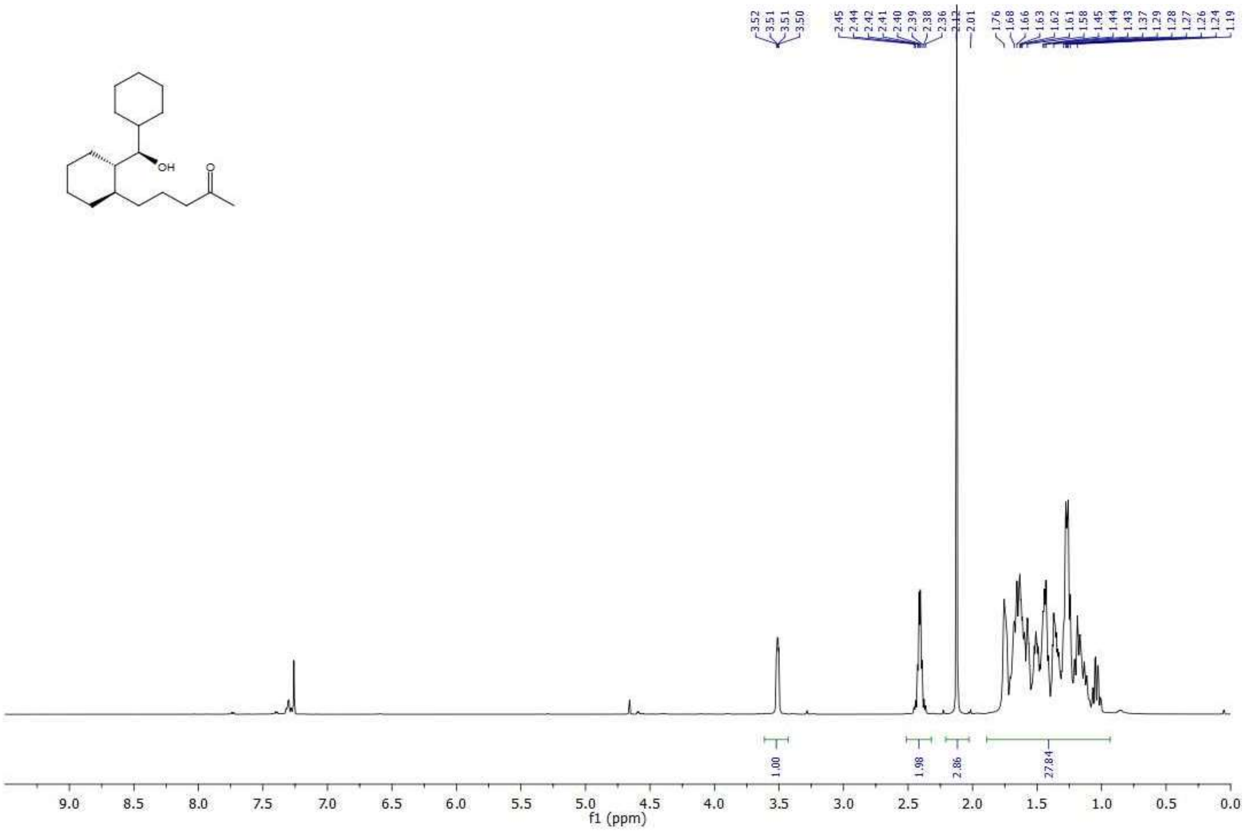
OH
O
1.00
1.98
2.86
27.84
f1 (ppm)

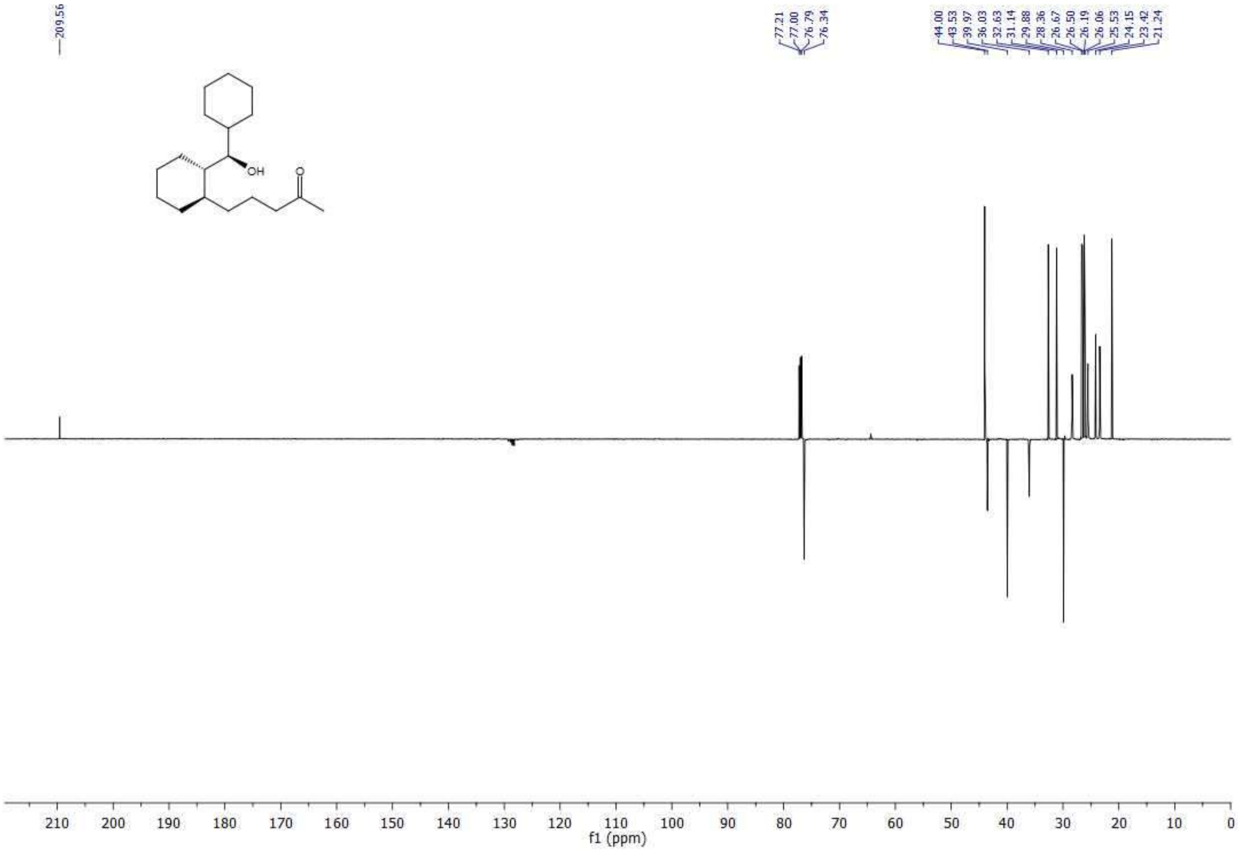
OH
O
f1 (ppm)

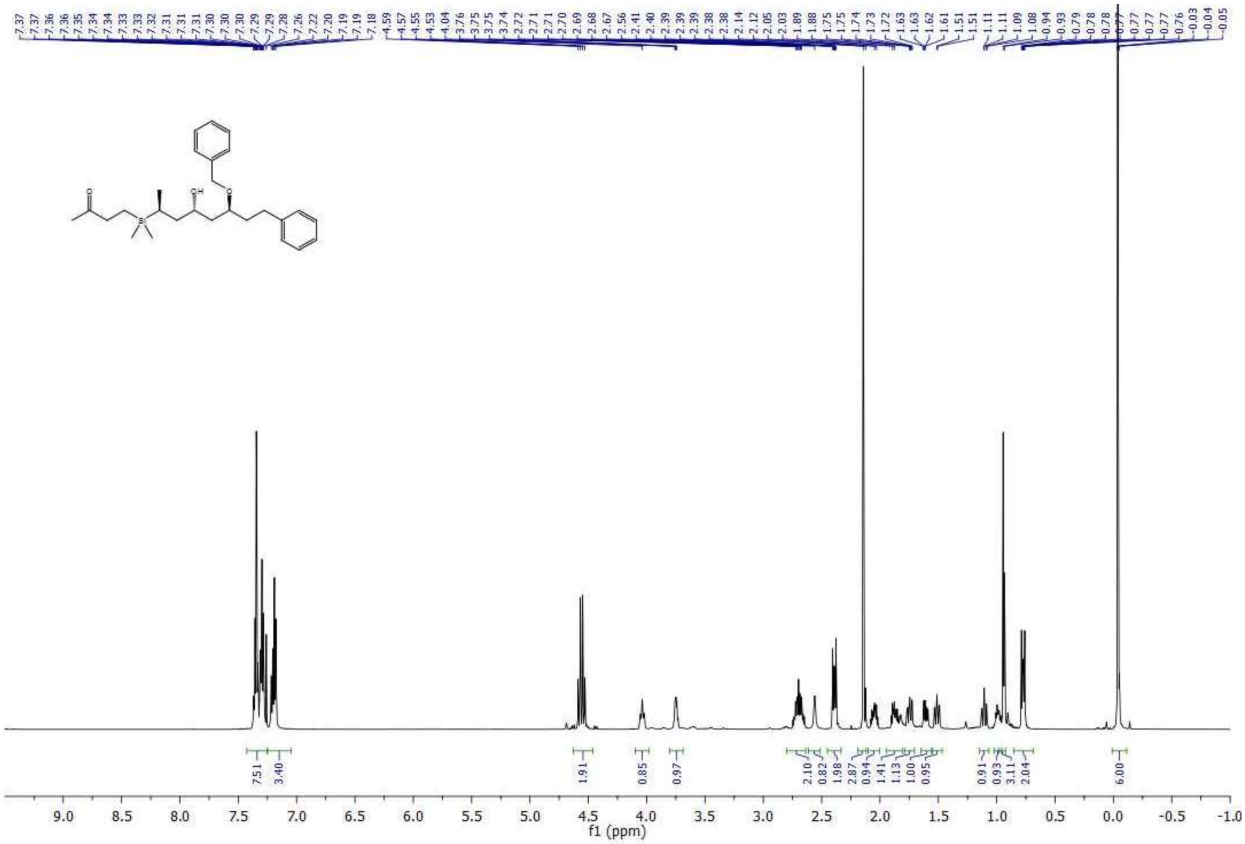

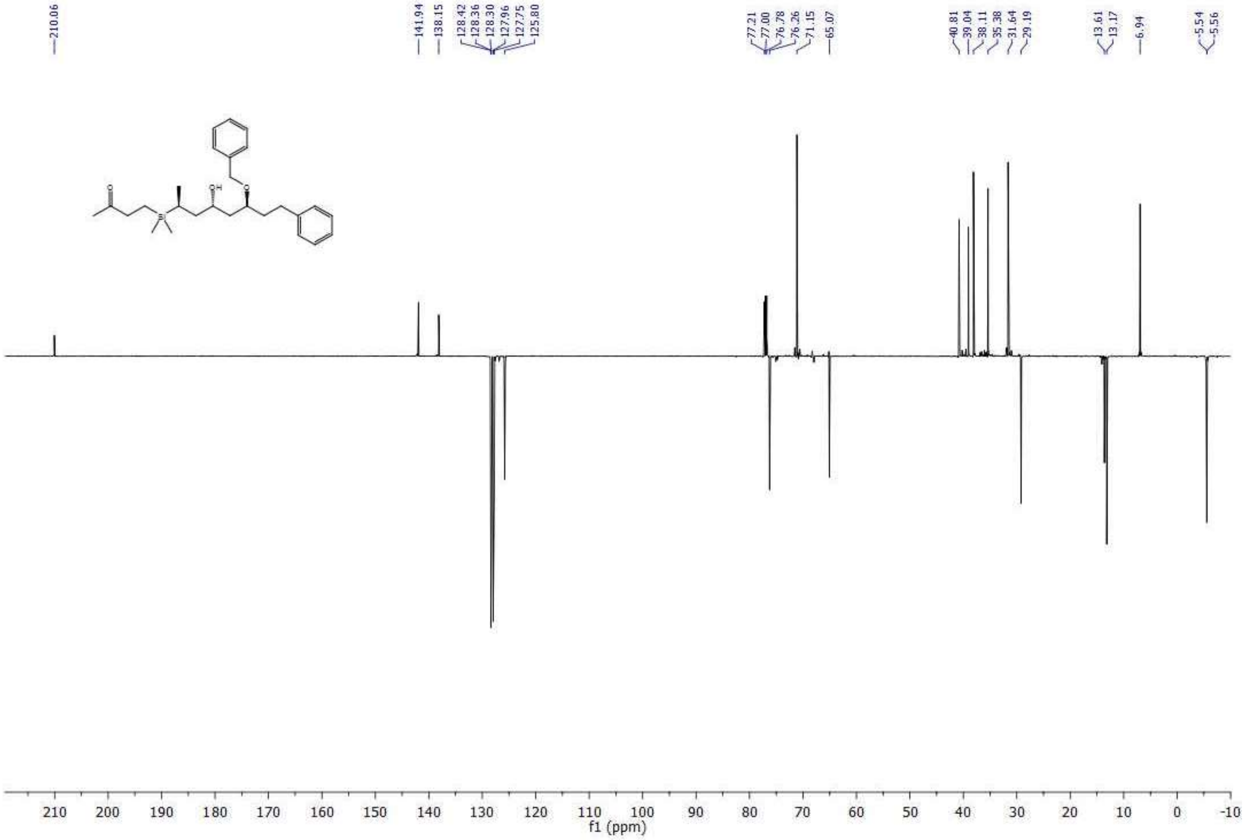

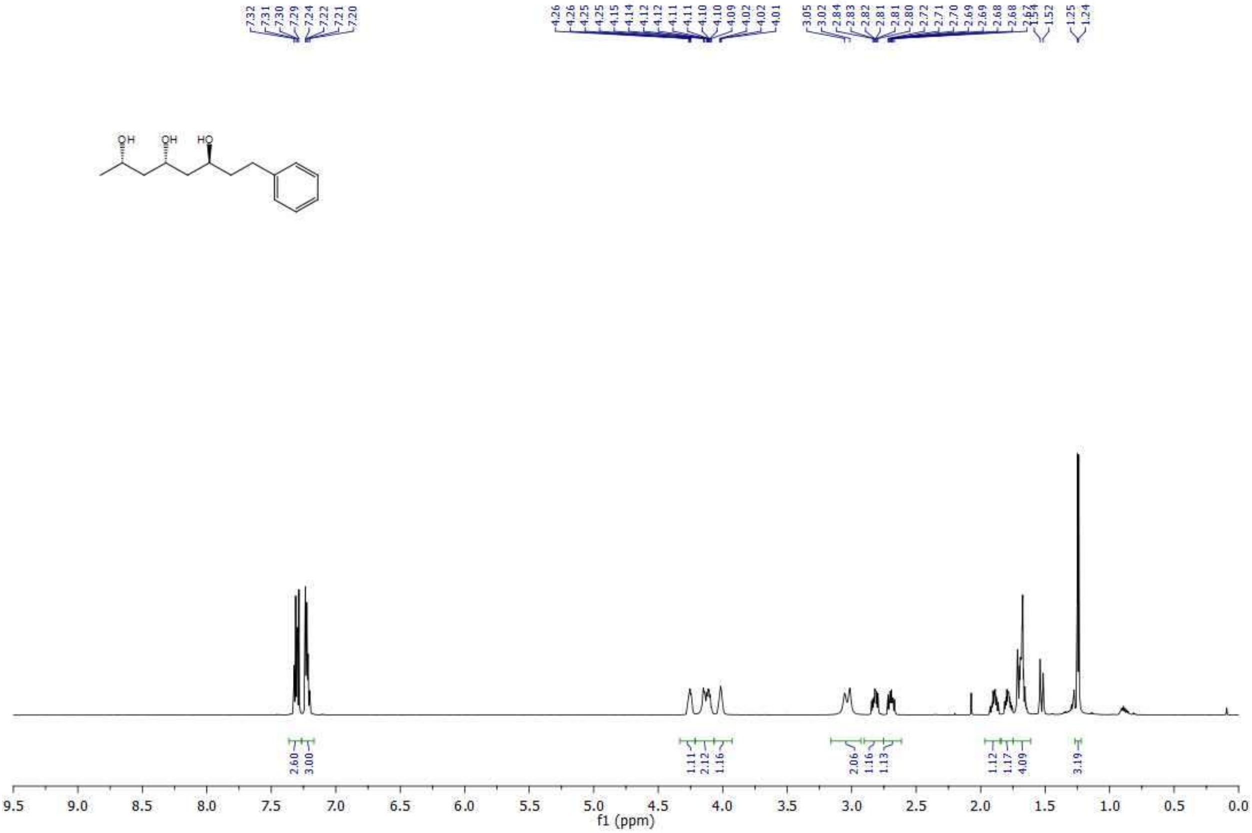

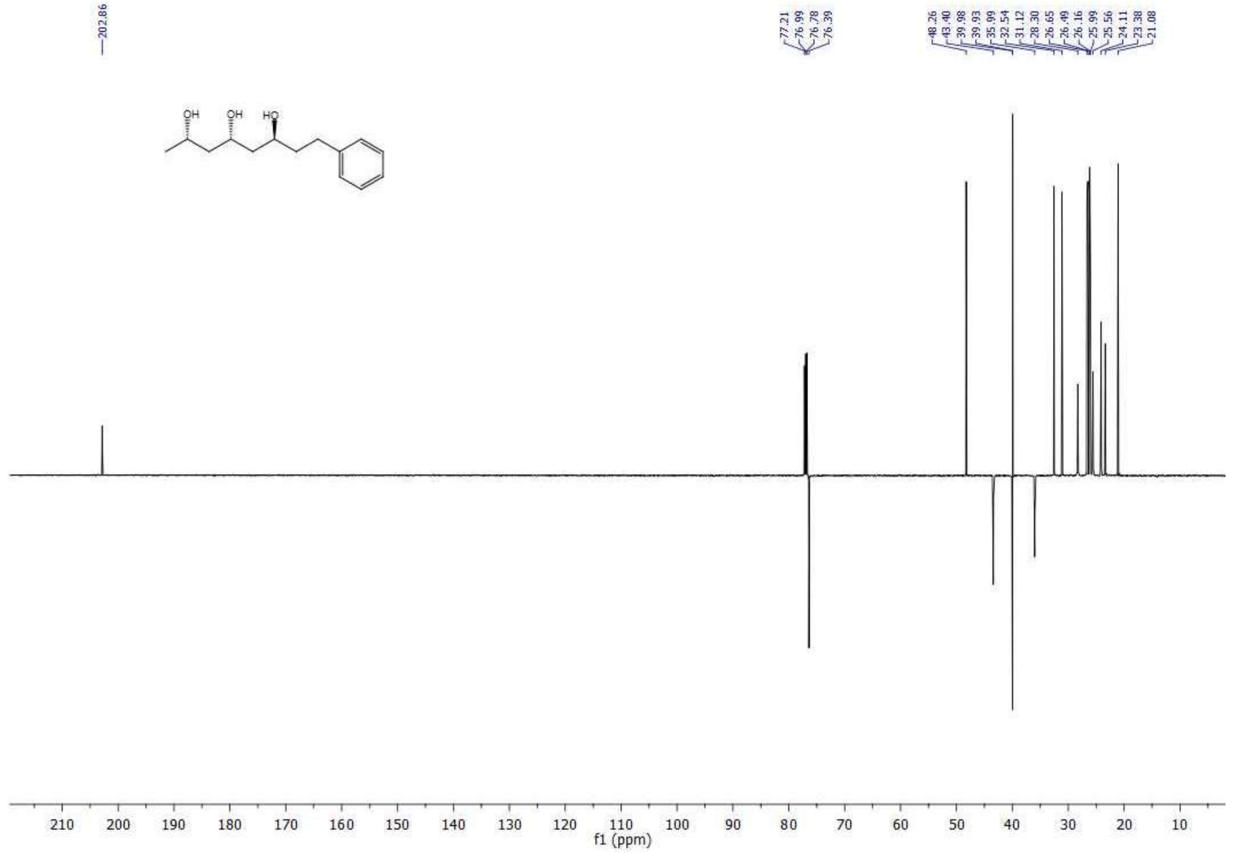

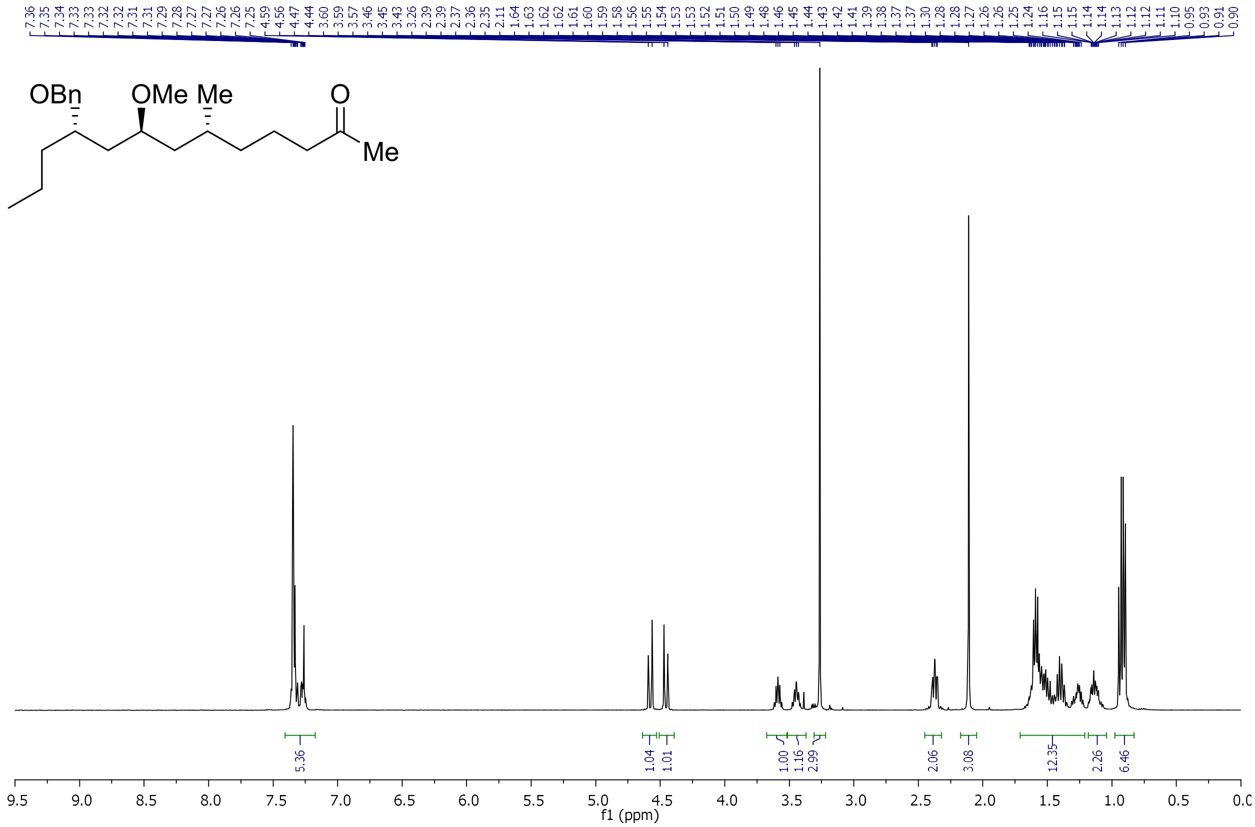
OBn
OMe
Me
O
Me
7.36 7.35 7.34 7.33 7.33 7.32 7.32 7.31 7.29 7.28 7.27 7.27 7.26 7.26 7.25 4.59 4.56 4.47 4.44 3.60 3.59 3.57 3.46 3.45 3.43 3.26 2.39 2.39 2.37 2.36 2.35 2.11 1.64 1.63 1.62 1.62 1.61 1.60 1.59 1.58 1.56 1.55 1.54 1.53 1.53 1.52 1.51 1.50 1.49 1.48 1.46 1.45 1.44 1.43 1.42 1.41 1.39 1.38 1.37 1.37 1.30 1.28 1.28 1.27 1.26 1.26 1.25 1.24 1.16 1.15 1.15 1.14 1.14 1.13 1.12 1.12 1.11 1.10 0.95 0.93 0.91 0.90
5.36
1.04
1.01
1.00
1.16
2.99
2.06
3.08
12.35
2.26
6.46
9.5 9.0 8.5 8.0 7.5 7.0 6.5 6.0 5.5 5.0 4.5 4.0 3.5 3.0 2.5 2.0 1.5 1.0 0.5 0.0
f1 (ppm)

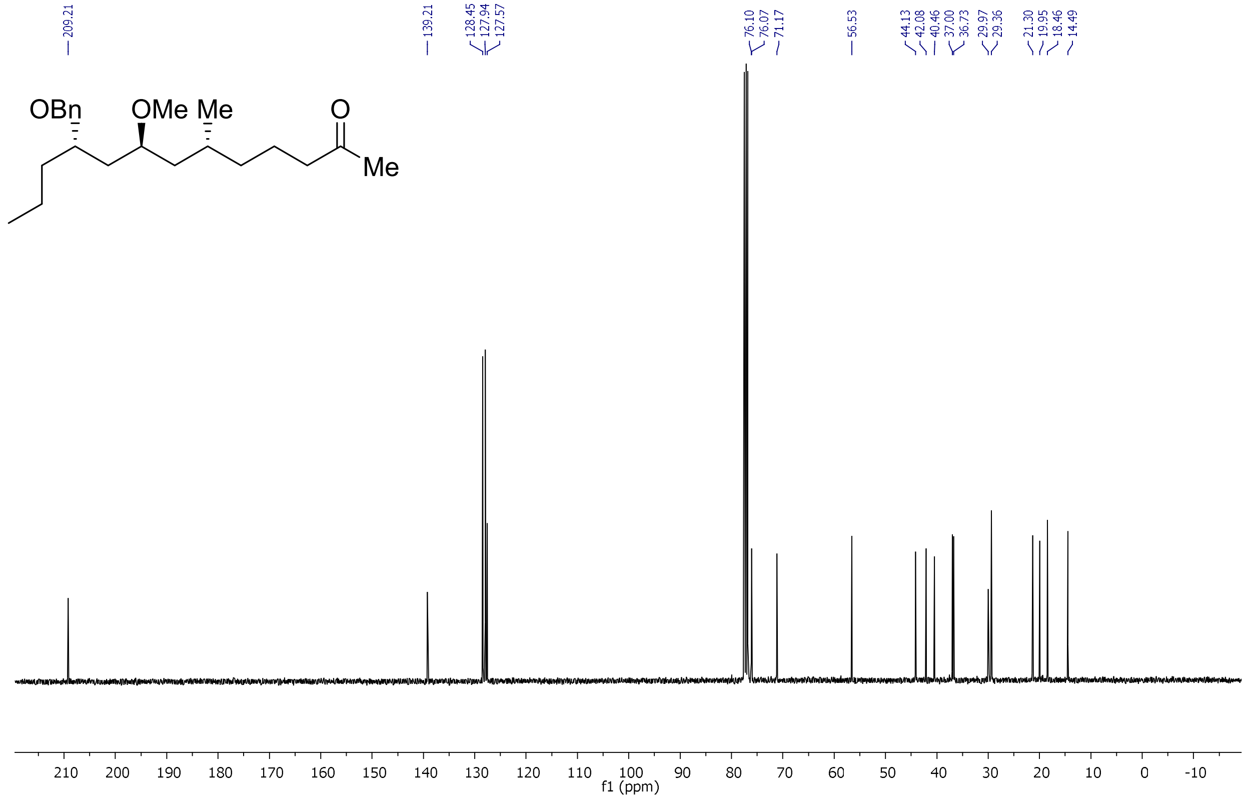
209.21
139.21
128.45 127.94 127.57
76.10 76.07 71.17
56.53
44.13 42.08 40.46 37.00 36.73 29.97 29.36
21.30 19.95 18.46 14.49
OBn
OMe
Me
O
Me
210 200 190 180 170 160 150 140 130 120 110 100 90 80 70 60 50 40 30 20 10 0 -10
f1 (ppm)

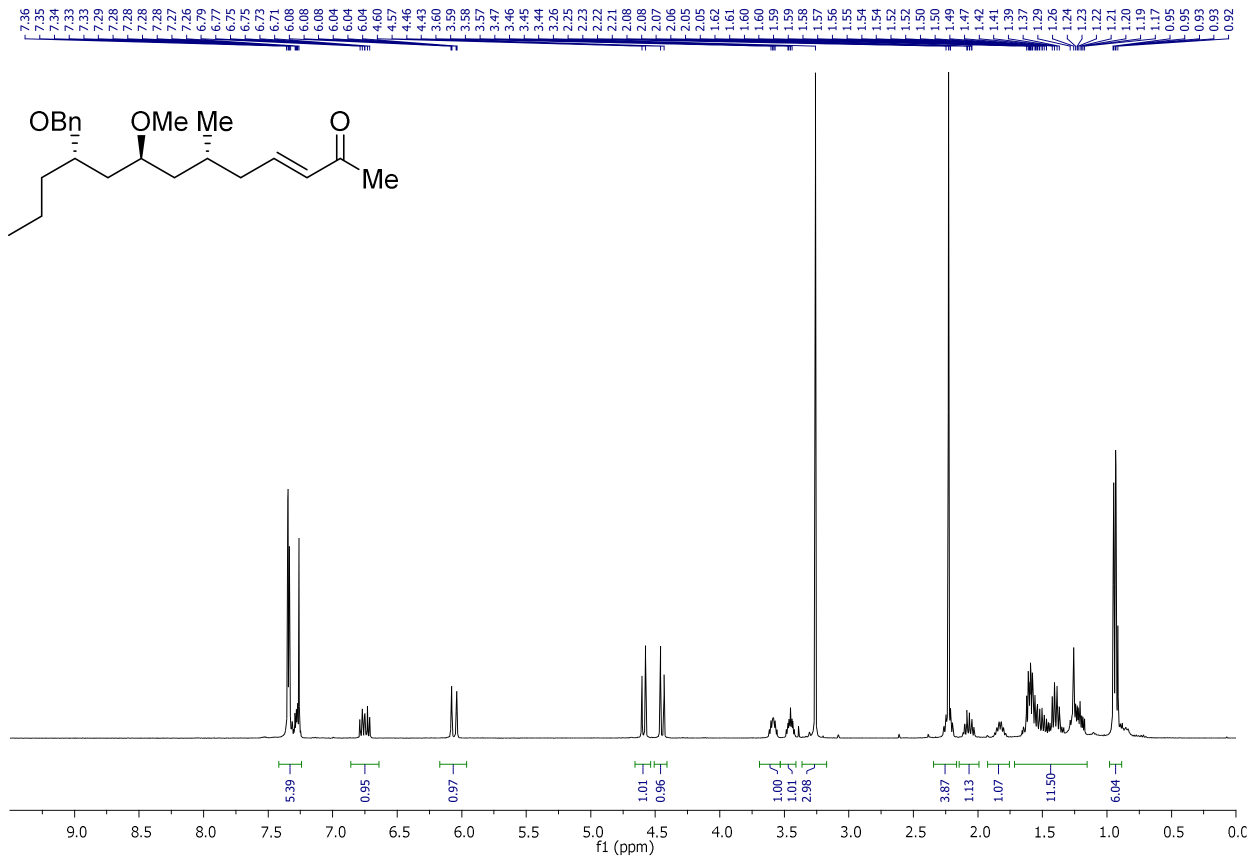

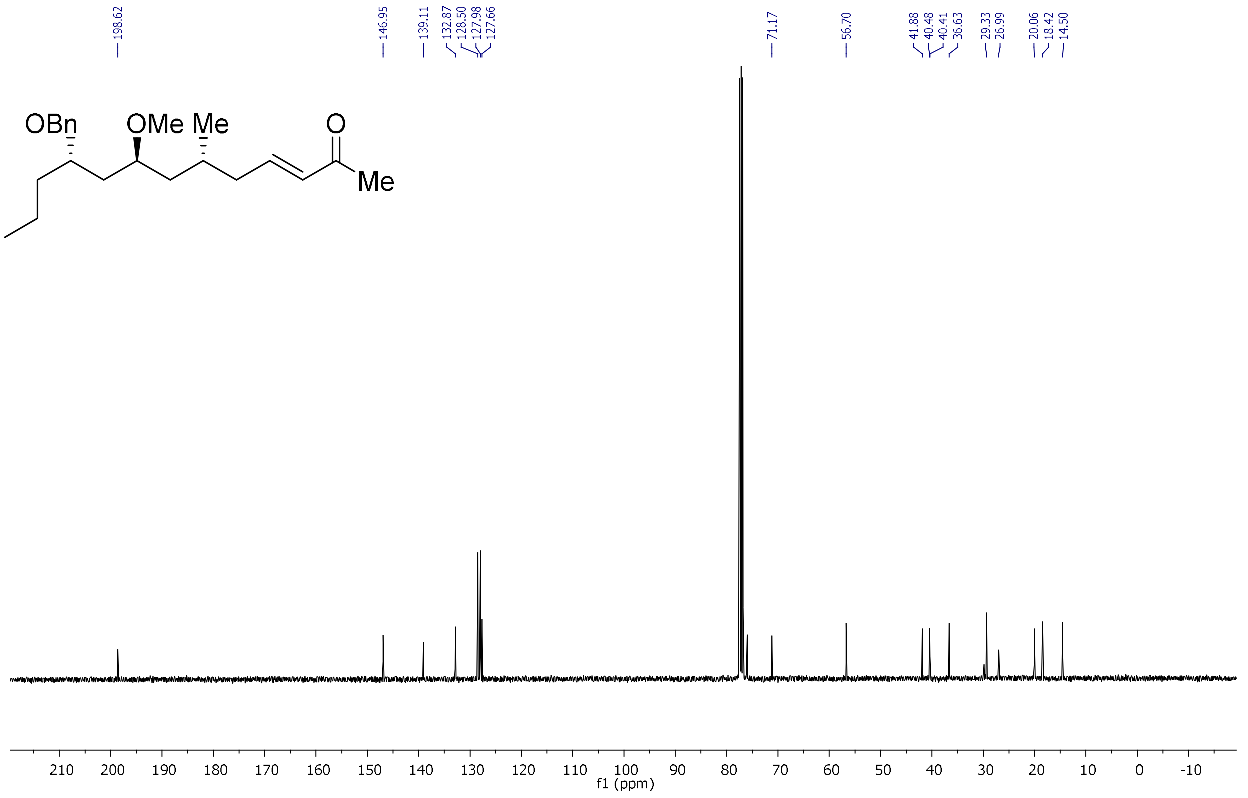

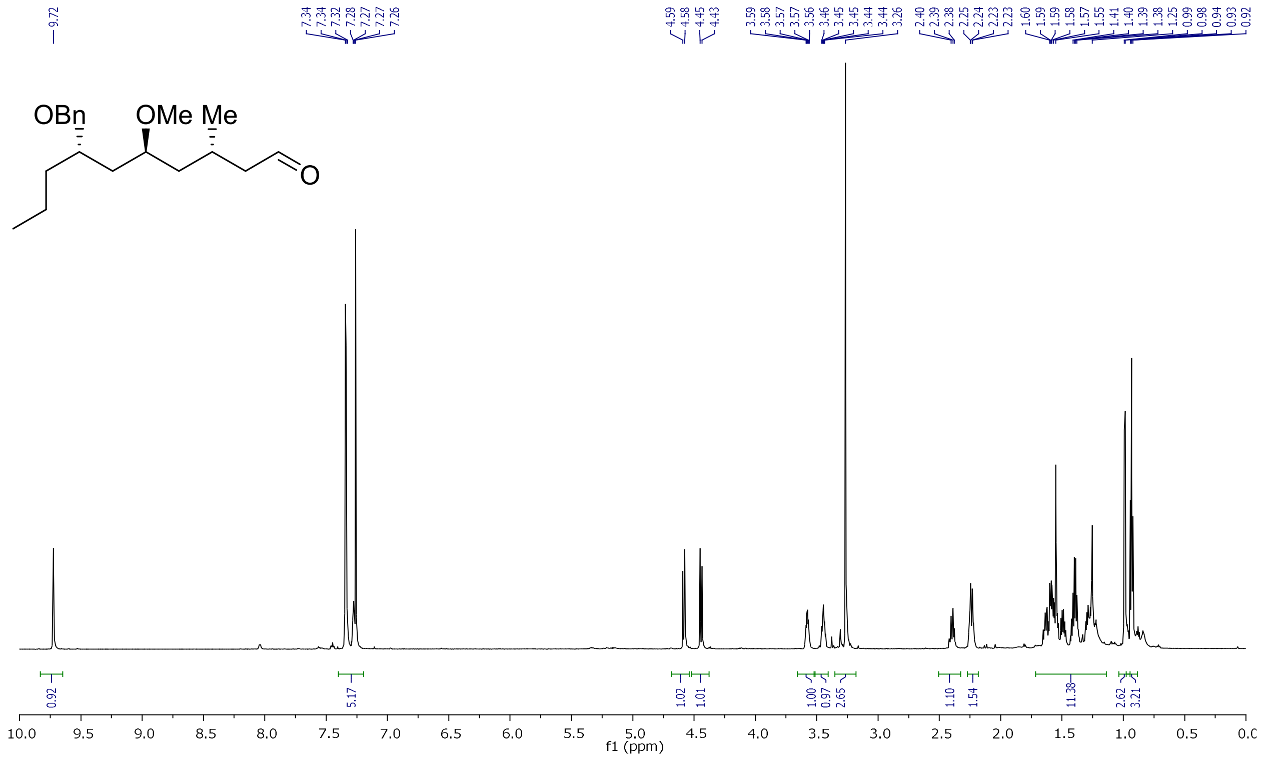
OBn
OMe Me
O
f1 (ppm)

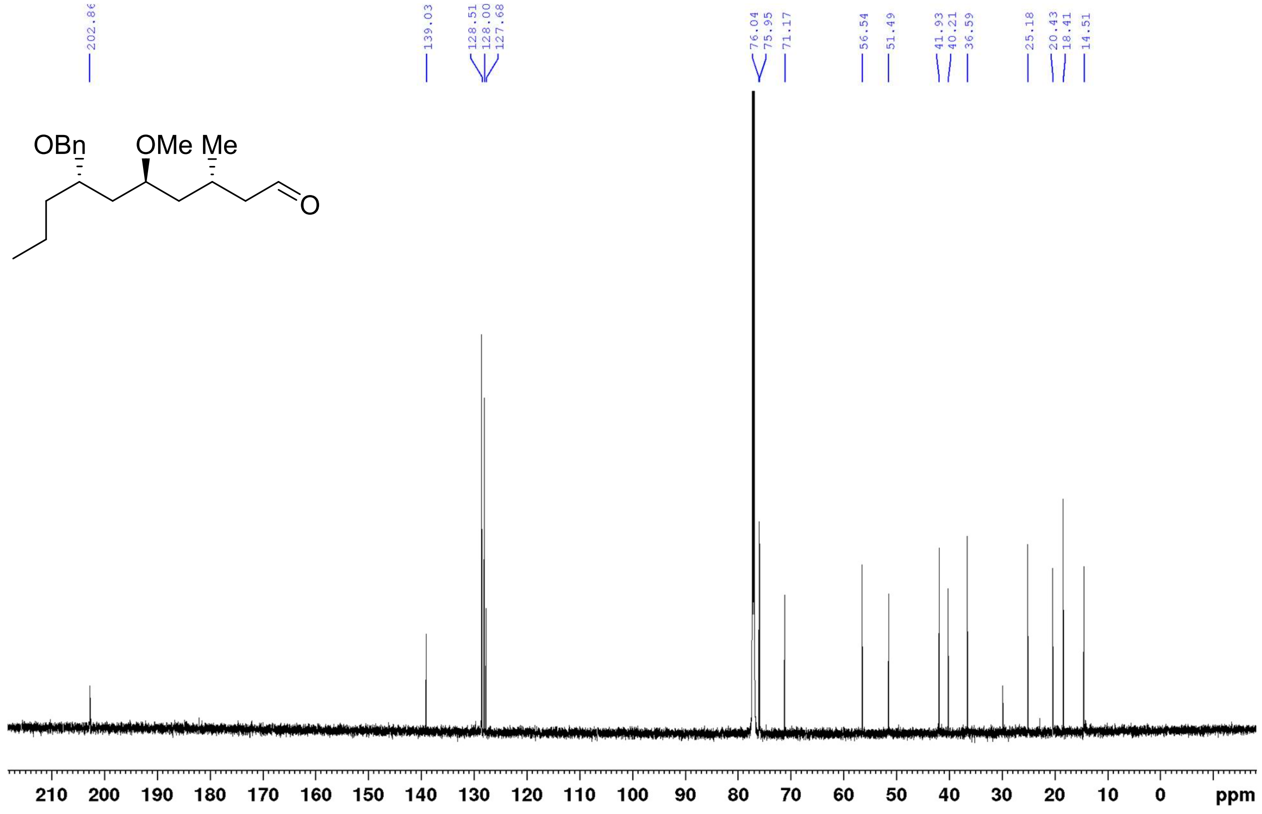
OBn
OMe Me
O
ppm

# HPLC

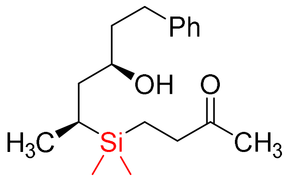


Method Description:
Column:Lux-Cellulose1 (Chiralcel OD-H) 250x4,6mm
Solvent System: n-Heptan+0,1%IPA/EtOH 95:5
Flow:0,7 ml/min

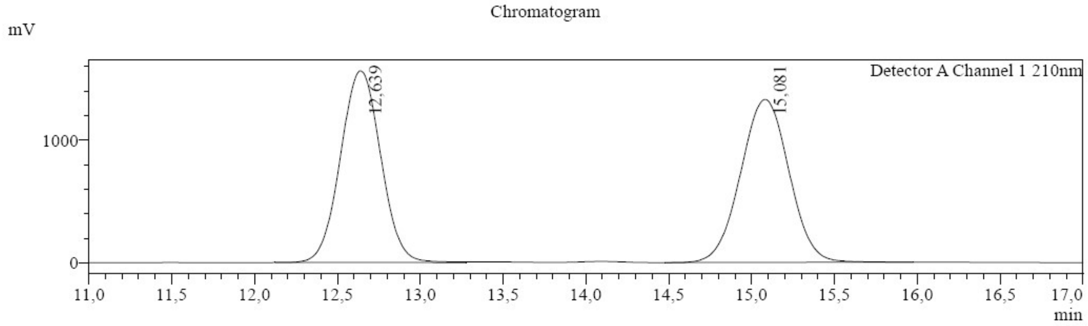


Detector A Channel 1 210nm

| Peak# | Ret. Time | Area | Area% |
|---|---|---|---|
| 1 | 12,639 | 26485892 | 49,523 |
| 2 | 15,081 | 26996573 | 50,477 |
| Total | | 53482465 | 100,000 |

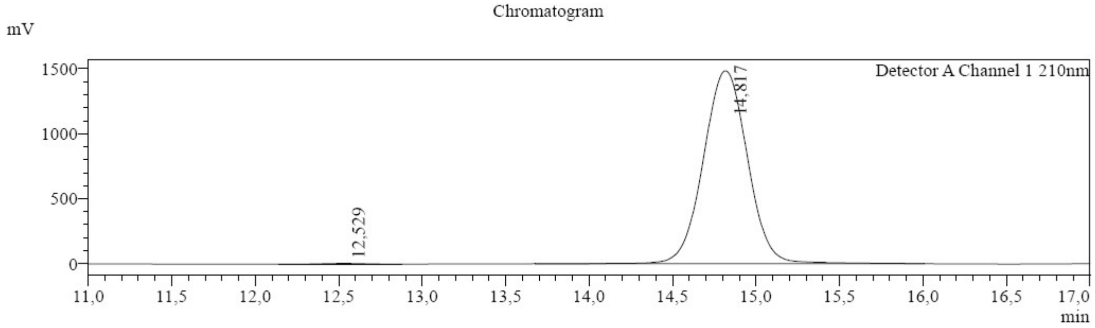


Detector A Channel 1 210nm

| Peak# | Ret. Time | Area | Area% |
|---|---|---|---|
| 1 | 12,529 | 101485 | 0,370 |
| 2 | 14,817 | 27295456 | 99,630 |
| Total | | 27396941 | 100,000 |

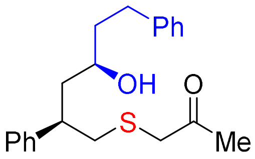


Method Description:
Column:Lux-Cellulose1 (Chiralcel OD-H) 250x4,6mm
Solvent System: n-Heptan+0,1%IPA/IPA 8:2
Flow:1 ml/min

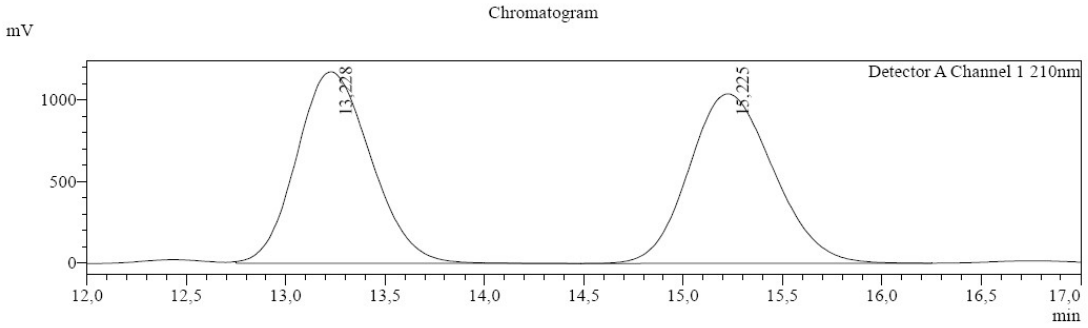


Detector A Channel 1 210nm

| Peak# | Ret. Time | Area | Area% |
|---|---|---|---|
| 1 | 13,228 | 30577577 | 49,906 |
| 2 | 15,225 | 30692497 | 50,094 |
| Total | | 61270074 | 100,000 |

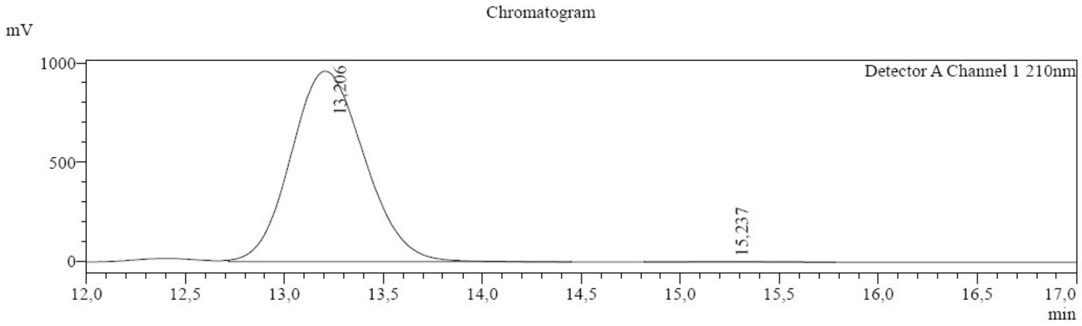


Detector A Channel 1 210nm

| Peak# | Ret. Time | Area | Area% |
|---|---|---|---|
| 1 | 13,206 | 24756036 | 99,755 |
| 2 | 15,237 | 60870 | 0,245 |
| Total | | 24816906 | 100,000 |

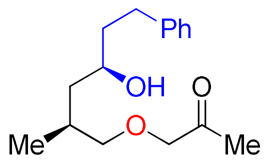


Method Description:
Column:Lux-Cellulose1 (Chiralcel OD-H) 250x4,6mm
Solvent System: n-Heptan+0,1%IPA/EtOH 95:5
Flow:0,7 ml/min

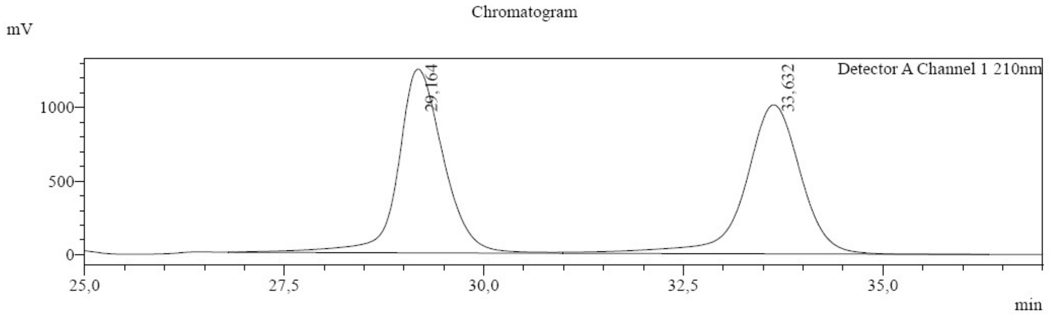


Detector A Channel 1 210nm

| Peak# | Ret. Time | Area | Area% |
|---|---|---|---|
| 1 | 29,164 | 49609429 | 50,239 |
| 2 | 33,632 | 49136513 | 49,761 |
| Total | | 98745942 | 100,000 |

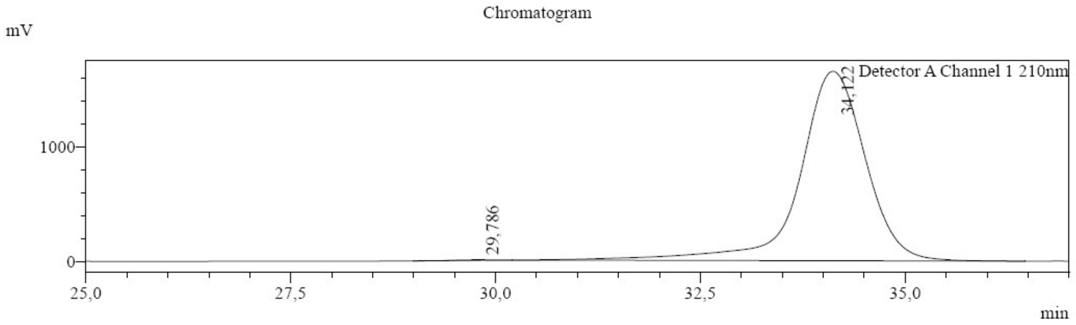


Detector A Channel 1 210nm

| Peak# | Ret. Time | Area | Area% |
|---|---|---|---|
| 1 | 29,786 | 133739 | 0,144 |
| 2 | 34,122 | 92650914 | 99,856 |
| Total | | 92784653 | 100,000 |

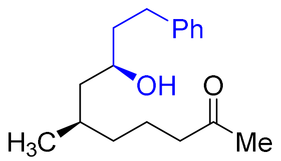


**3a**

Method Description:
Column:Chiralcel OD-H 250x4,6mm
Solvent System: n-Heptan+0,1%IPA/EtOH 95:5
Flow:0,7 ml/min

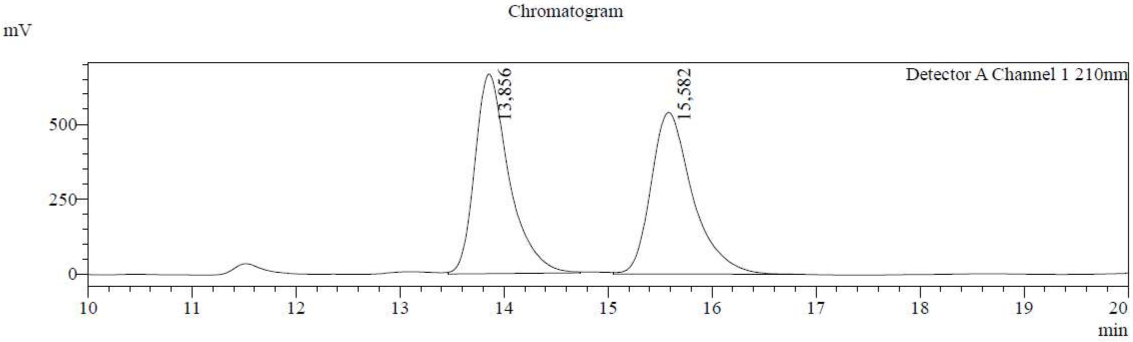


Detector A Channel 1 210nm

| Peak# | Ret. Time | Area | Area% |
|---|---|---|---|
| 1 | 13,856 | 15227375 | 50,300 |
| 2 | 15,582 | 15045760 | 49,700 |
| Total | | 30273135 | 100,000 |

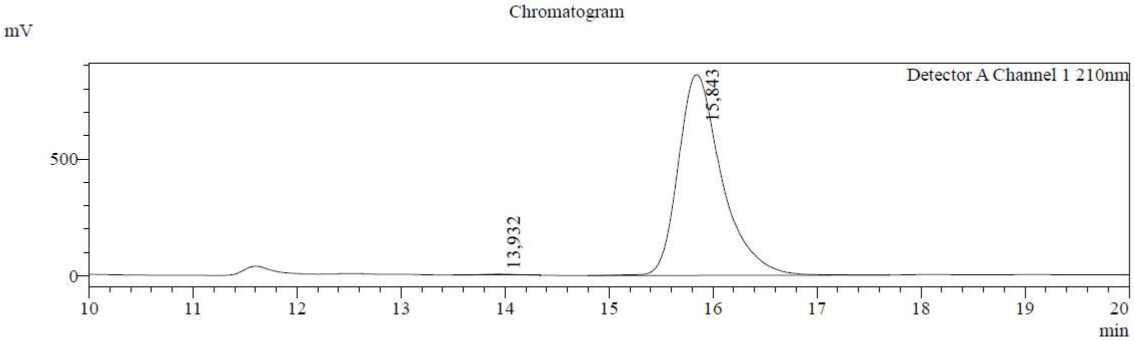


Detector A Channel 1 210nm

| Peak# | Ret. Time | Area | Area% |
|---|---|---|---|
| 1 | 13,932 | 100742 | 0,400 |
| 2 | 15,843 | 25086410 | 99,600 |
| Total | | 25187153 | 100,000 |

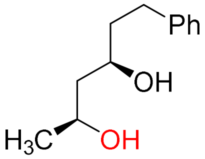


Method Description:
Column:Lux-Cellulose1 (Chiralcel OD-H) 250x4,6mm
Solvent System: n-Heptan+0,1%IPA/IPA 9:1
Flow:1 ml/min

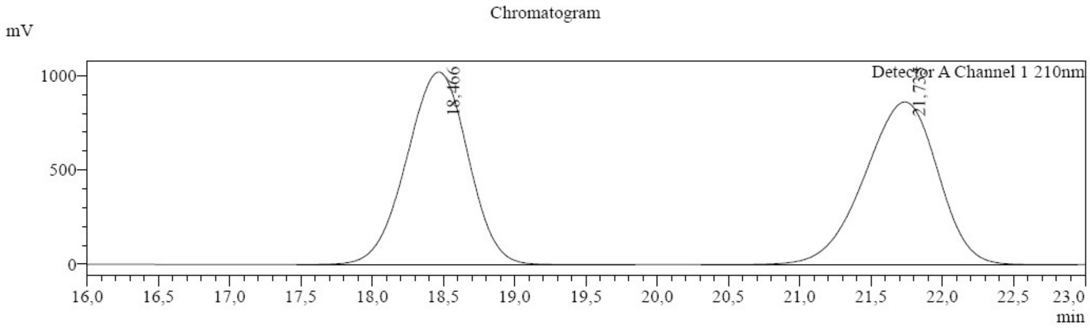


Detector A Channel 1 210nm

| Peak# | Ret. Time | Area | Area% |
|---|---|---|---|
| 1 | 18,466 | 30596960 | 49,762 |
| 2 | 21,735 | 30889482 | 50,238 |
| Total | | 61486442 | 100,000 |

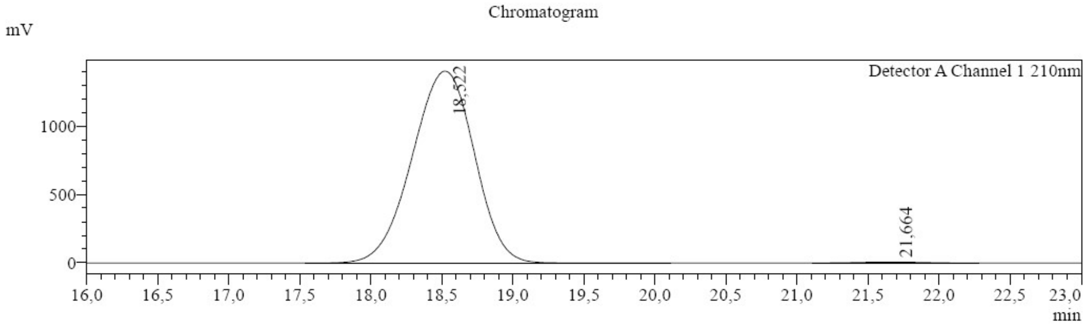


Detector A Channel 1 210nm

| Peak# | Ret. Time | Area | Area% |
|---|---|---|---|
| 1 | 18,522 | 42807899 | 99,490 |
| 2 | 21,664 | 219420 | 0,510 |
| Total | | 43027319 | 100,000 |

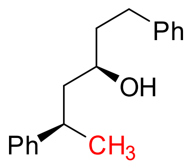


Method Description:
Column:Chiralpak IC 250x4,6mm Particle Size 5 micrometer
Solvent System: n-Heptan/IPA 99:1
Flow:0,7 ml/min T=25°C

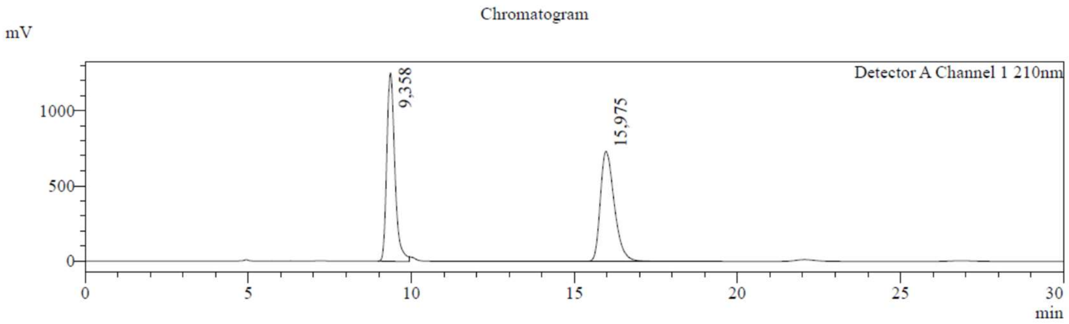


Detector A Channel 1 210nm

| Peak# | Ret. Time | Area | Area% |
|---|---|---|---|
| 1 | 9,358 | 21355893 | 49,451 |
| 2 | 15,975 | 21830131 | 50,549 |
| Total | | 43186025 | 100,000 |

Method Description:
Column:Chiralpak IC 250x4,6mm Particle Size 5 micrometer
Solvent System: n-Heptan/IPA 99:1
Flow:0,7 ml/min T=25°C

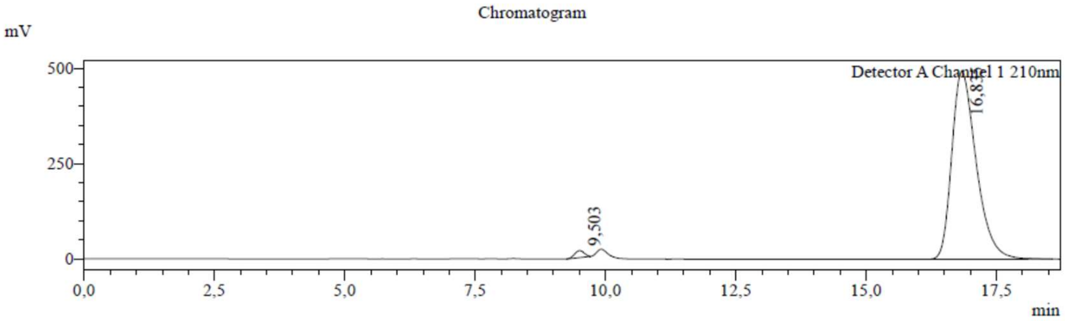


Detector A Channel 1 210nm

| Peak# | Ret. Time | Area | Area% |
|---|---|---|---|
| 1 | 9,503 | 229536 | 1,408 |
| 2 | 16,835 | 16071866 | 98,592 |
| Total | | 16301402 | 100,000 |

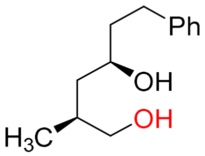

Method Description:
Column:Chiralpak IC 250x4,6mm Particle Size 5 micrometer
Solvent System: n-Heptan+0,1%IPA/IPA 9,5:0,5
Flow:1 ml/min T=25°C

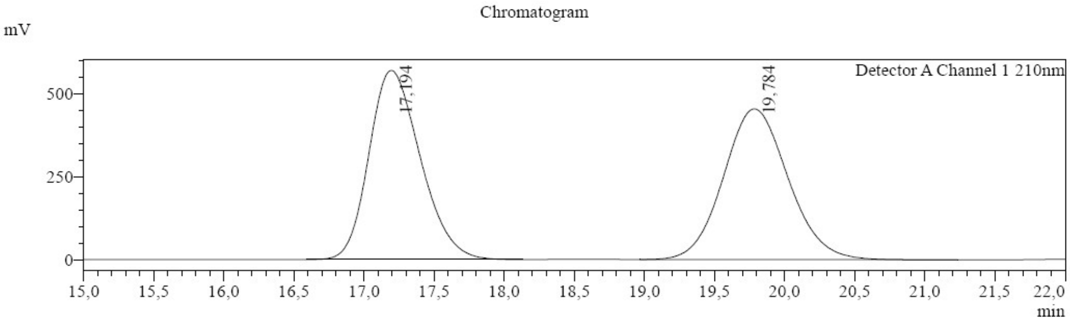


Detector A Channel 1 210nm

| Peak# | Ret. Time | Area | Area% |
|---|---|---|---|
| 1 | 17,194 | 14500596 | 49,833 |
| 2 | 19,784 | 14597535 | 50,167 |
| Total | | 29098131 | 100,000 |

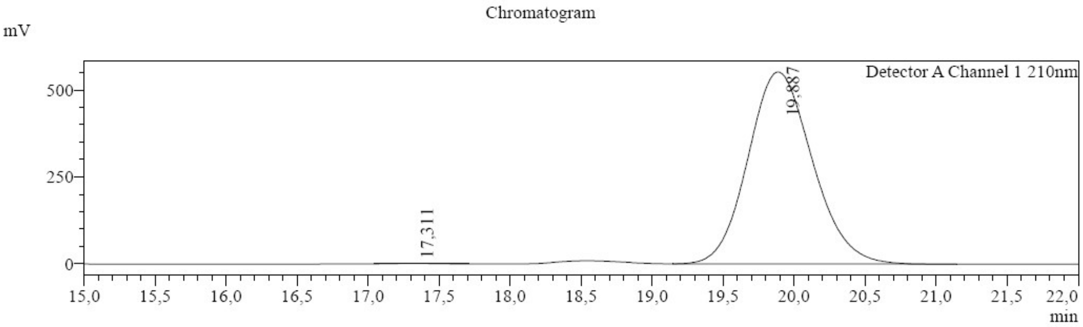


Detector A Channel 1 210nm

| Peak# | Ret. Time | Area | Area% |
|---|---|---|---|
| 1 | 17,311 | 26946 | 0,155 |
| 2 | 19,887 | 17402122 | 99,845 |
| Total | | 17429067 | 100,000 |

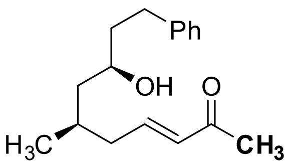


Method Description:
Column:Chiralpak IA 250x4,6mm Particle Size 5 micrometer
Solvent System: n-Heptan+0,1%IPA/IPA 98,5:1,5
Flow:0,7 ml/min T=25°C

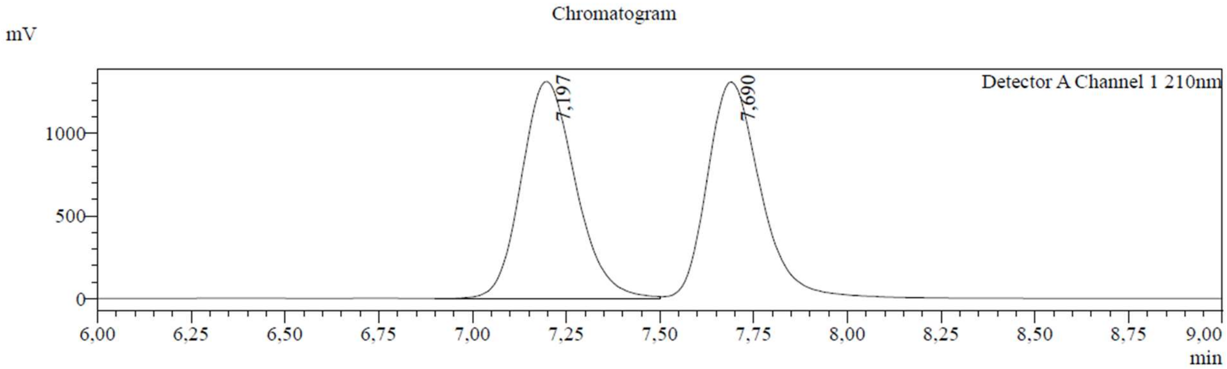


Detector A Channel 1 210nm

| Peak# | Ret. Time | Area | Area% |
|---|---|---|---|
| 1 | 7,197 | 13230578 | 50,412 |
| 2 | 7,690 | 13014542 | 49,588 |
| Total | | 26245120 | 100,000 |

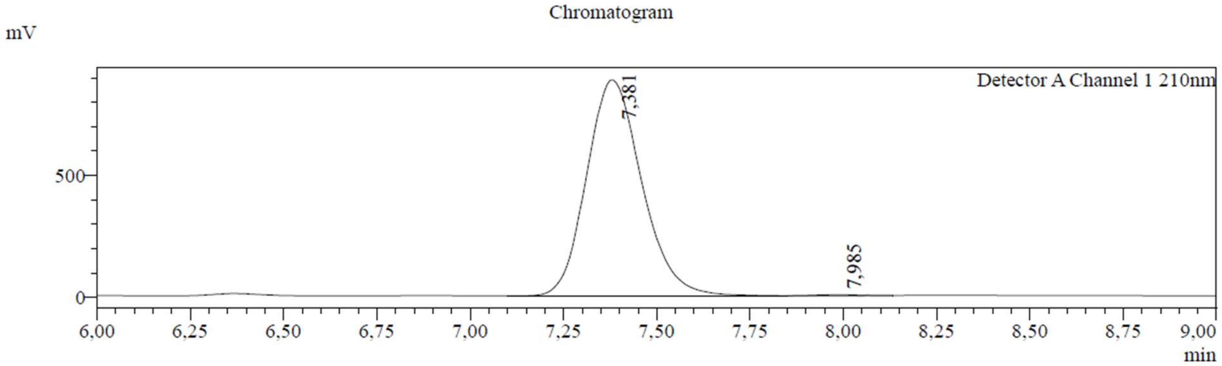


Detector A Channel 1 210nm

| Peak# | Ret. Time | Area | Area% |
|---|---|---|---|
| 1 | 7,381 | 9125615 | 99,697 |
| 2 | 7,985 | 27769 | 0,303 |
| Total | | 9153385 | 100,000 |

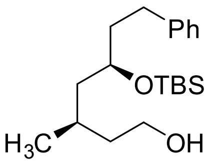


Method Description:
Column:Lux-Cellulose1 (Chiralcel OD-H) 250x4,6mm
Solvent System: n-Heptan+0,1%IPA/IPA 92:8
Flow:0,7 ml/min

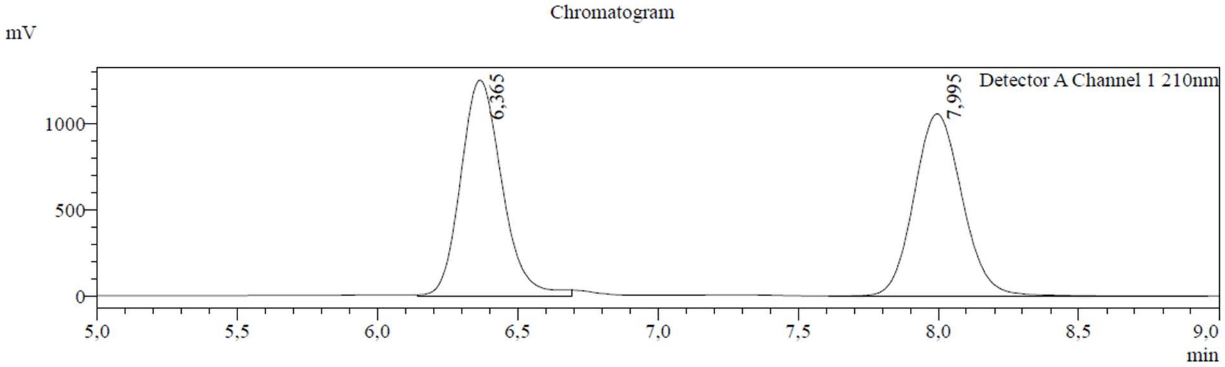


Detector A Channel 1 210nm

| Peak# | Ret. Time | Area | Area% |
|---|---|---|---|
| 1 | 6,365 | 12765643 | 50,368 |
| 2 | 7,995 | 12578918 | 49,632 |
| Total | | 25344561 | 100,000 |

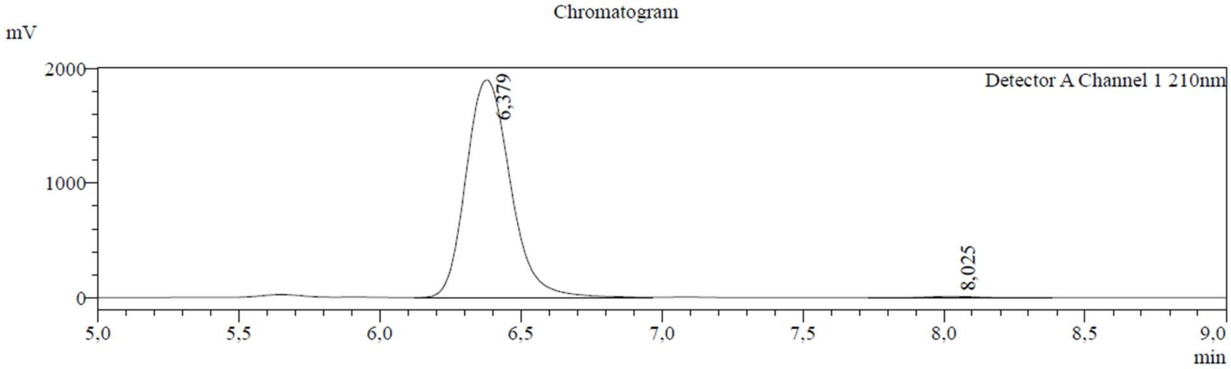


Detector A Channel 1 210nm

| Peak# | Ret. Time | Area | Area% |
|---|---|---|---|
| 1 | 6,379 | 20878529 | 99,398 |
| 2 | 8,025 | 126432 | 0,602 |
| Total | | 21004961 | 100,000 |

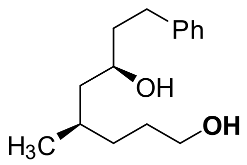


Method Description:
Column:Lux-Cellulose1 (Chiralcel OD-H) 250x4,6mm
Solvent System: n-Heptan+0,1%IPA/IPA 85:15
Flow:0,7 ml/min

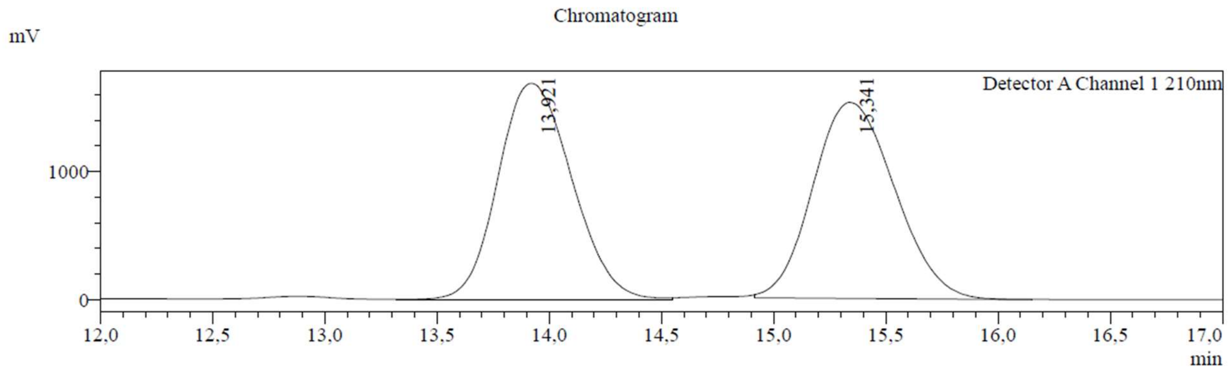


Detector A Channel 1 210nm

| Peak# | Ret. Time | Area | Area% |
|---|---|---|---|
| 1 | 13,921 | 38563857 | 49,881 |
| 2 | 15,341 | 38747412 | 50,119 |
| Total | | 77311268 | 100,000 |

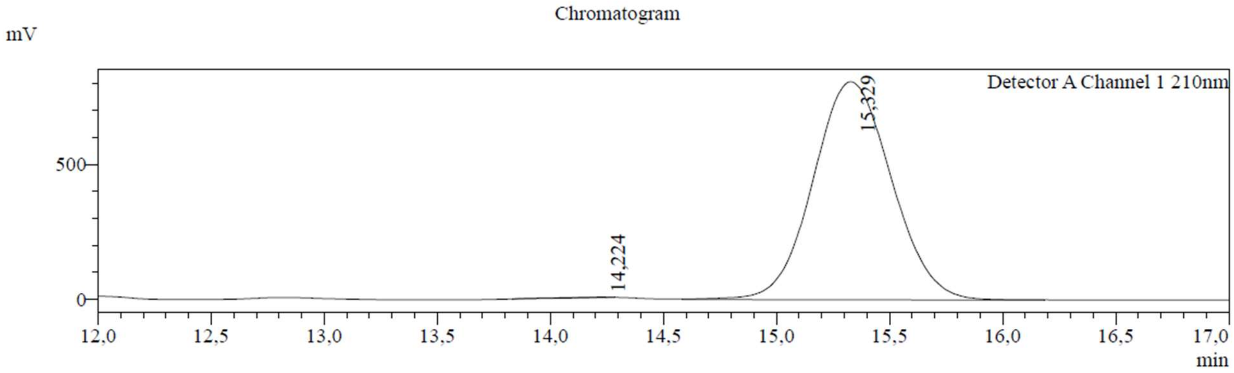


Detector A Channel 1 210nm

| Peak# | Ret. Time | Area | Area% |
|---|---|---|---|
| 1 | 14,224 | 33120 | 0,170 |
| 2 | 15,329 | 19505985 | 99,830 |
| Total | | 19539105 | 100,000 |

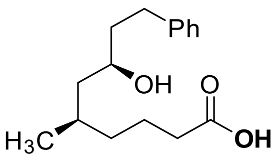


Method Description:
Column:Chiralpak IA 250x4,6mm Particle Size 5 micrometer
Solvent System: n-Heptan+1%EtOH+5%IPA+0,1%TFA
Flow:0,7 ml/min

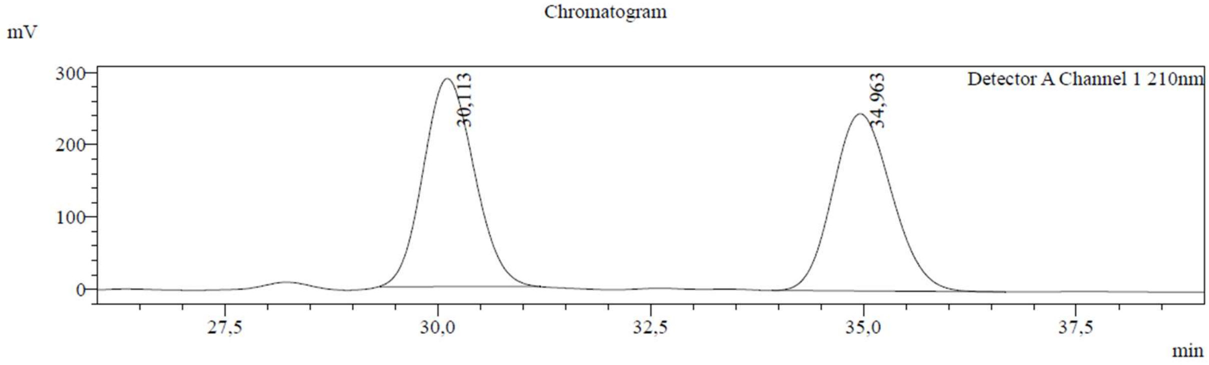


Detector A Channel 1 210nm

| Peak# | Ret. Time | Area | Area% |
|---|---|---|---|
| 1 | 30,113 | 12027285 | 50,391 |
| 2 | 34,963 | 11840586 | 49,609 |
| Total | | 23867871 | 100,000 |

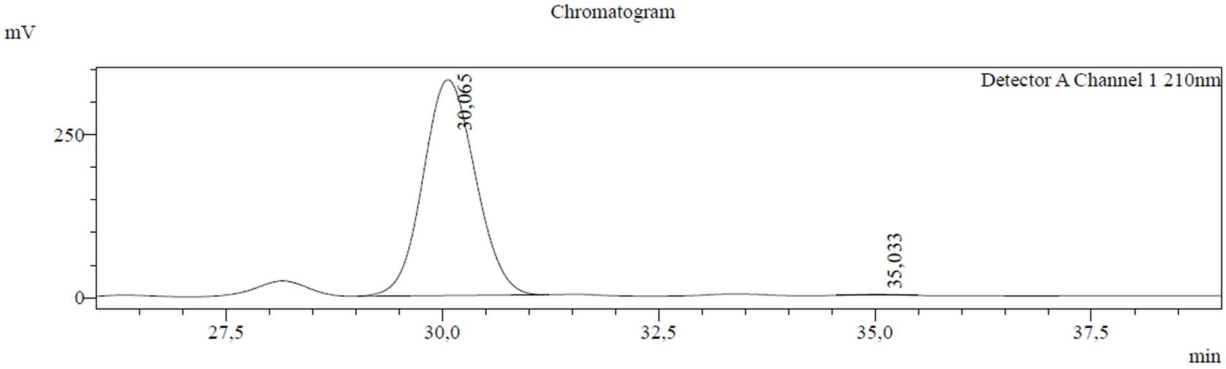


Detector A Channel 1 210nm

| Peak# | Ret. Time | Area | Area% |
|---|---|---|---|
| 1 | 30,065 | 13902395 | 99,698 |
| 2 | 35,033 | 42104 | 0,302 |
| Total | | 13944499 | 100,000 |

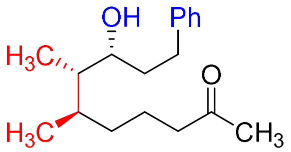


Method Description:
Column:Chiralcel OD-H 250x4,6mm
Solvent System: n-Heptan+0,1%IPA/IPA 95:5
Flow:1 ml/min

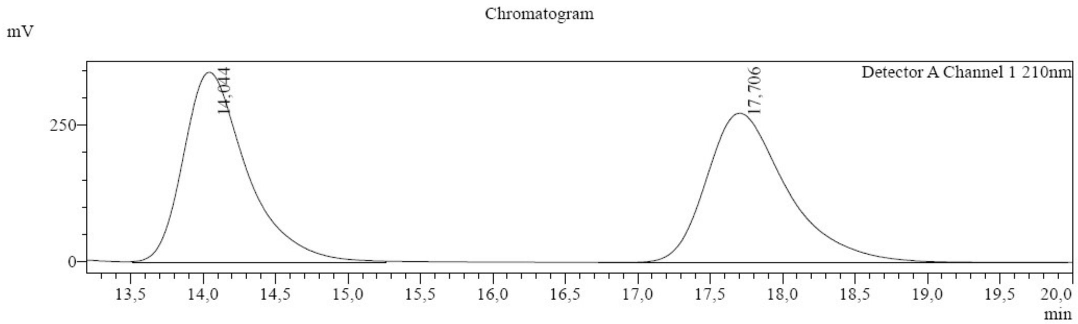


Detector A Channel 1 210nm

| Peak# | Ret. Time | Area | Area% |
|---|---|---|---|
| 1 | 14,044 | 10614896 | 50,171 |
| 2 | 17,706 | 10542618 | 49,829 |
| Total | | 21157513 | 100,000 |

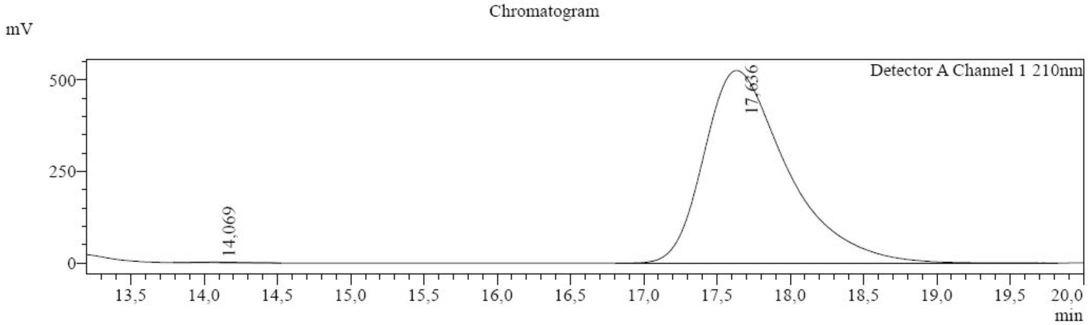


Detector A Channel 1 210nm

| Peak# | Ret. Time | Area | Area% |
|---|---|---|---|
| 1 | 14,069 | 33663 | 0,162 |
| 2 | 17,636 | 20750071 | 99,838 |
| Total | | 20783734 | 100,000 |

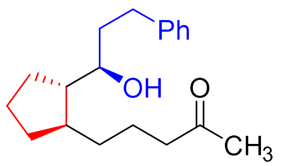


Method Description:
Column:Chiralpak IA 250x4,6mm Particle Size 5 micrometer
Solvent System: n-Heptan+0,1%IPA/IPA 92:8
Flow:0,7 ml/min

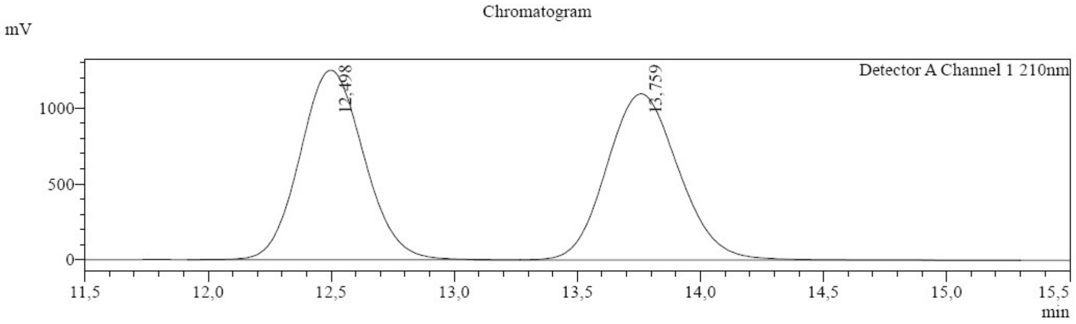


Detector A Channel 1 210nm

| Peak# | Ret. Time | Area | Area% |
|---|---|---|---|
| 1 | 12,498 | 22575856 | 50,246 |
| 2 | 13,759 | 22354851 | 49,754 |
| Total | | 44930707 | 100,000 |

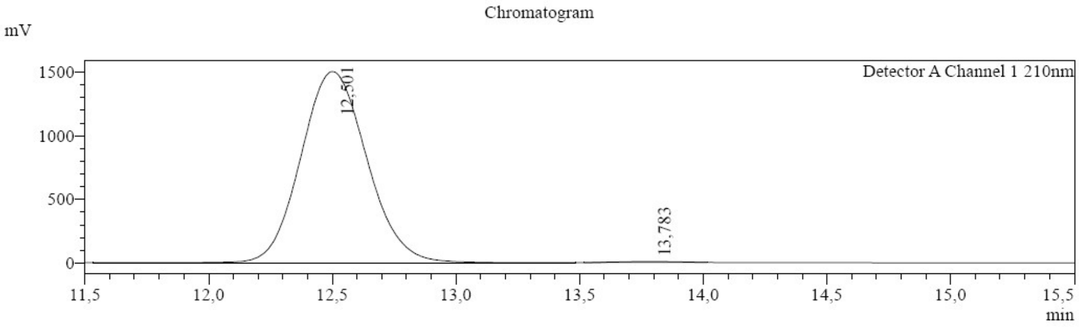


Detector A Channel 1 210nm

| Peak# | Ret. Time | Area | Area% |
|---|---|---|---|
| 1 | 12,501 | 28067987 | 99,553 |
| 2 | 13,783 | 126022 | 0,447 |
| Total | | 28194009 | 100,000 |

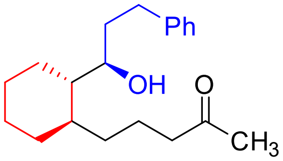


Method Description:
Column:Chiralpak IB 250x4,6 mm ID
Solvent System: n-Heptan+0,1%IPA/IPA 95:5
Flow:0,7 ml/min
T=25°C

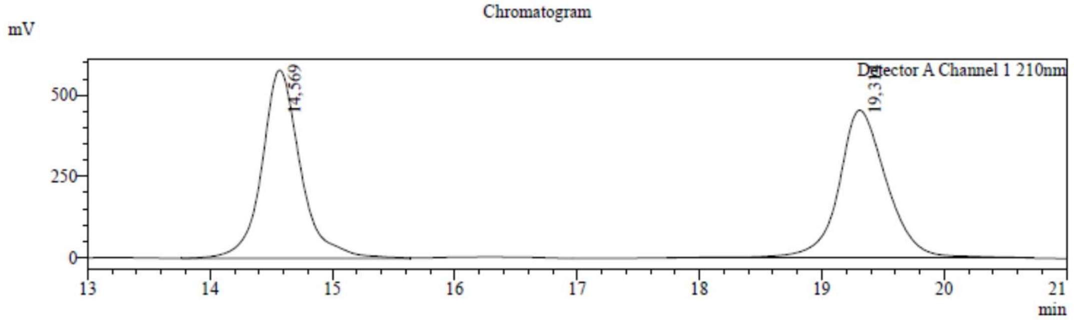


Detector A Channel 1 210nm

| Peak# | Ret. Time | Area | Area% |
|---|---|---|---|
| 1 | 14,569 | 12689608 | 50,492 |
| 2 | 19,314 | 12442491 | 49,508 |
| Total | | 25132099 | 100,000 |

Method Description:
Column:Chiralpak IB 250x4,6 mm ID
Solvent System: n-Heptan+0,1%IPA/IPA 95:5
Flow:0,7 ml/min
T=25°C

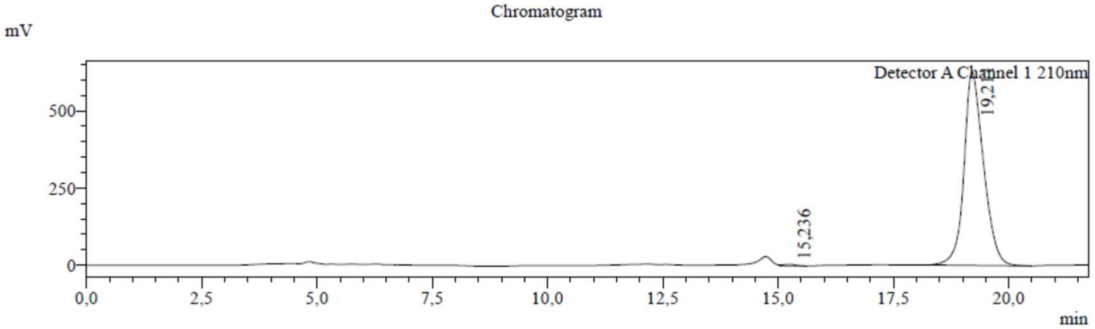


Detector A Channel 1 210nm

| Peak# | Ret. Time | Area | Area% |
|---|---|---|---|
| 1 | 15,236 | 97205 | 0,521 |
| 2 | 19,211 | 18552092 | 99,479 |
| Total | | 18649297 | 100,000 |

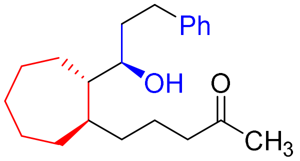


Method Description:
Column:Chiralpak IC 250x4,6mm Particle Size 5 micrometer
Solvent System: n-Heptan+0,1%IPA/EtOH 8:2
Flow:0,7 ml/min T=25°C

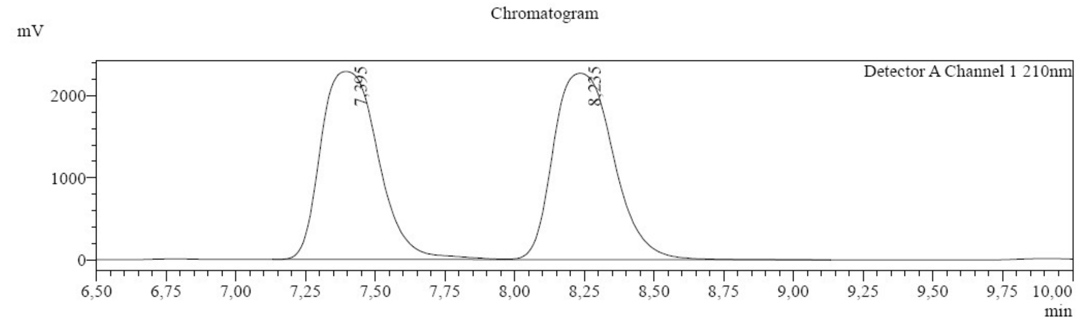


Detector A Channel 1 210nm

| Peak# | Ret. Time | Area | Area% |
|---|---|---|---|
| 1 | 7,395 | 32742906 | 49,156 |
| 2 | 8,235 | 33867381 | 50,844 |
| Total | | 66610287 | 100,000 |

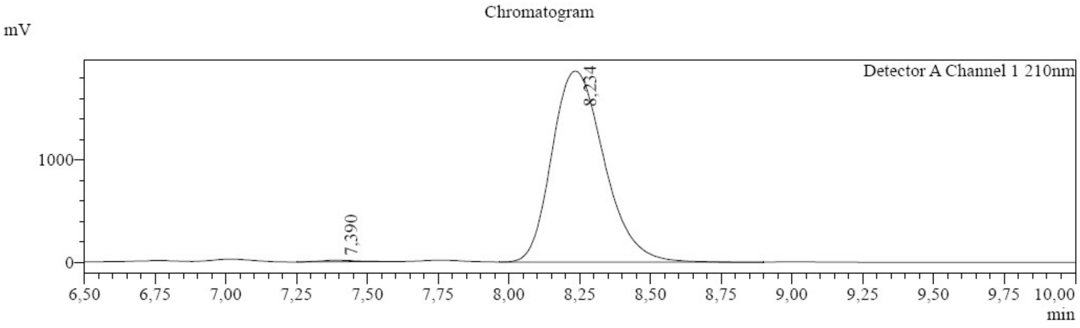


Detector A Channel 1 210nm

| Peak# | Ret. Time | Area | Area% |
|---|---|---|---|
| 1 | 7,390 | 113588 | 0,472 |
| 2 | 8,234 | 23965433 | 99,528 |
| Total | | 24079021 | 100,000 |

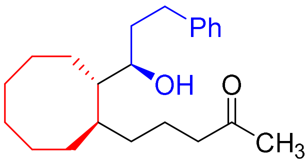


Method Description:
Column:Chiralpak IC 250x4,6mm Particle Size 5 micrometer
Solvent System:( n-Heptan+0,1%IPA)/EtOH 9:1
Flow=1ml/min T=25°C

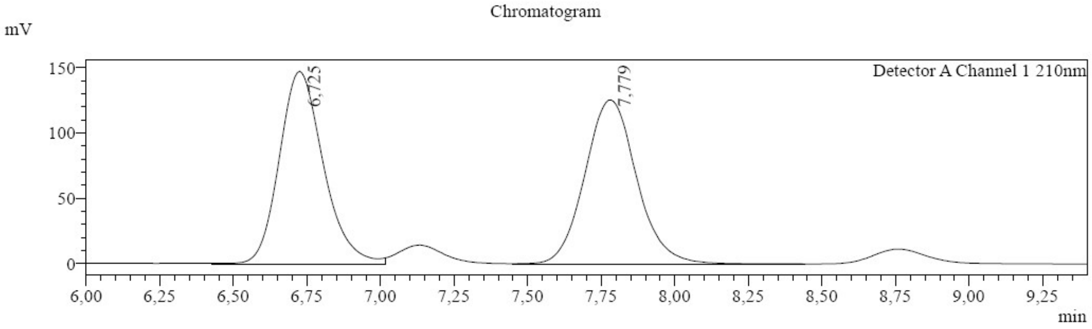


Detector A Channel 1 210nm

| Peak# | Ret. Time | Area | Area% |
|---|---|---|---|
| 1 | 6,725 | 1539172 | 50,085 |
| 2 | 7,779 | 1533924 | 49,915 |
| Total | | 3073096 | 100,000 |

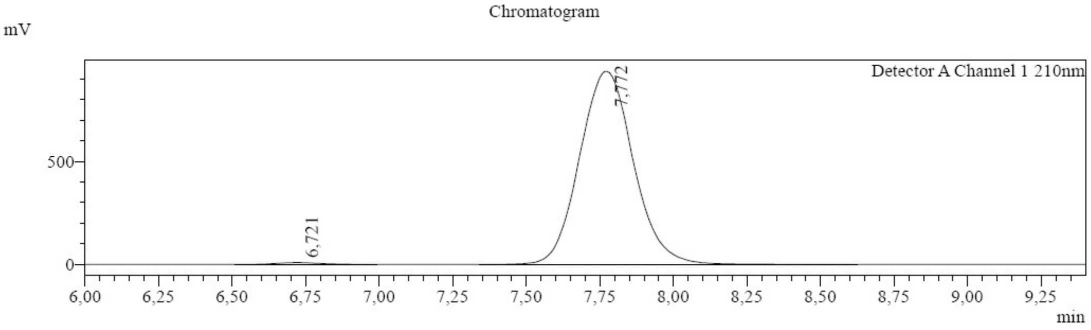


Detector A Channel 1 210nm

| Peak# | Ret. Time | Area | Area% |
|---|---|---|---|
| 1 | 6,721 | 90336 | 0,750 |
| 2 | 7,772 | 11949593 | 99,250 |
| Total | | 12039929 | 100,000 |

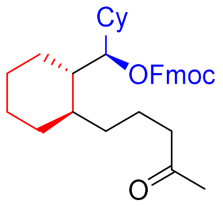


Method Description:
Column:Lux-Cellulose1 (Chiralcel OD-H) 250x4,6mm
Solvent System: n-Heptan+0,1%IPA/IPA 95:5
Flow:1ml/min

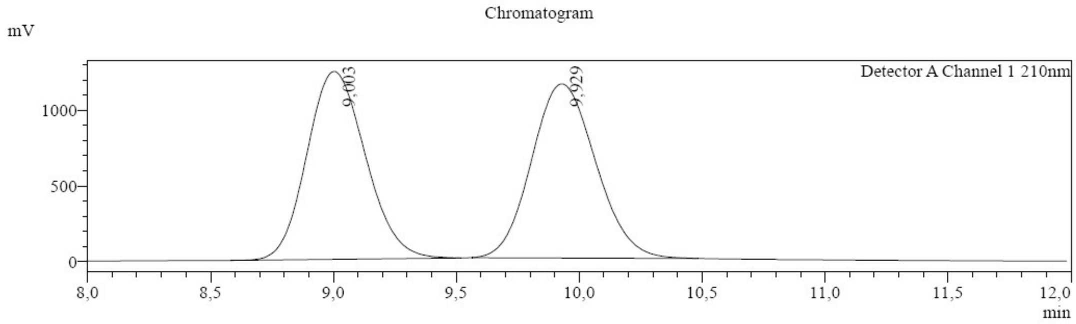


Detector A Channel 1 210nm

| Peak# | Ret. Time | Area | Area% |
|---|---|---|---|
| 1 | 9,003 | 20564374 | 49,634 |
| 2 | 9,929 | 20867465 | 50,366 |
| Total | | 41431839 | 100,000 |

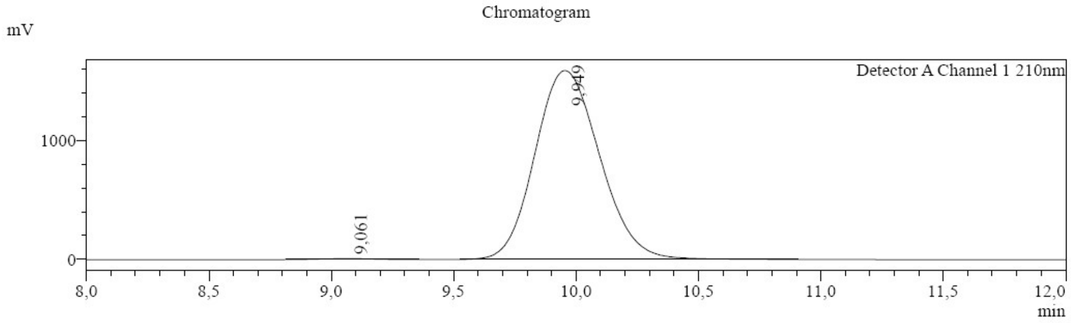


Detector A Channel 1 210nm

| Peak# | Ret. Time | Area | Area% |
|---|---|---|---|
| 1 | 9,061 | 108656 | 0,371 |
| 2 | 9,949 | 29216586 | 99,629 |
| Total | | 29325241 | 100,000 |

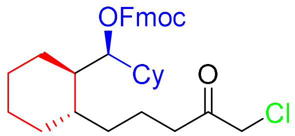


Method Description:
Column:Lux-Cellulose1 (Chiralcel OD-H) 250x4,6mm
Solvent System: n-Heptan+0,1%IPA/IPA 95:5
Flow:1ml/min

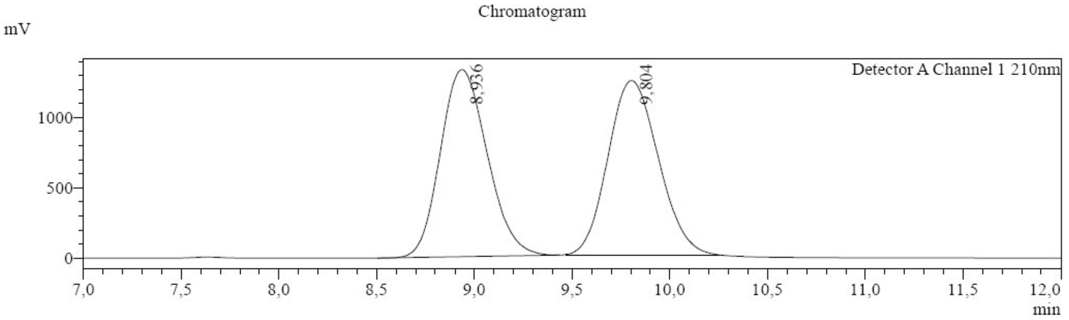


Detector A Channel 1 210nm

| Peak# | Ret. Time | Area | Area% |
|---|---|---|---|
| 1 | 8,936 | 22205019 | 49,986 |
| 2 | 9,804 | 22217337 | 50,014 |
| Total | | 44422356 | 100,000 |

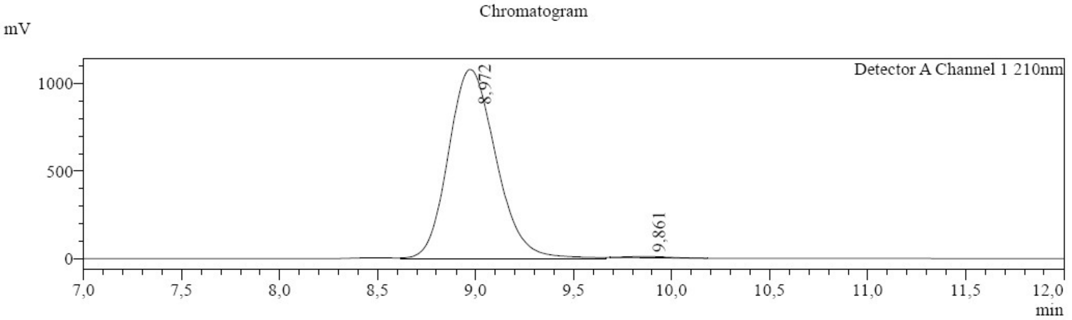


Detector A Channel 1 210nm

| Peak# | Ret. Time | Area | Area% |
|---|---|---|---|
| 1 | 8,972 | 18318735 | 99,551 |
| 2 | 9,861 | 82637 | 0,449 |
| Total | | 18401371 | 100,000 |

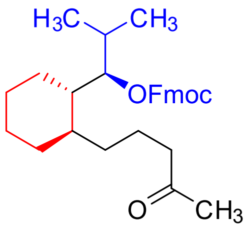


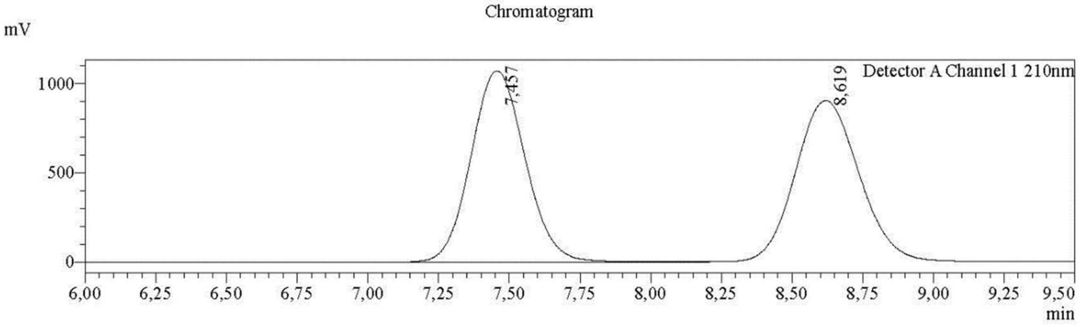


Detector A Channel 1 210nm

| Peak# | Ret. Time | Area | Area% |
|---|---|---|---|
| 1 | 7,457 | 14186911 | 50,073 |
| 2 | 8,619 | 14145389 | 49,927 |
| Total | | 28332300 | 100,000 |

Method Description:
Column:Lux-Cellulose1 (Chiralcel OD-H) 250x4,6mm
Solvent System: n-Heptan+0,1%IPA/IPA 9:1
Flow:1 ml/min

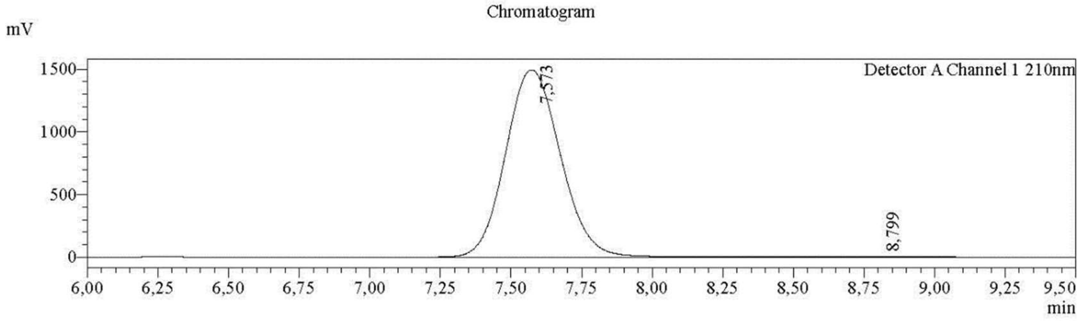


Detector A Channel 1 210nm

| Peak# | Ret. Time | Area | Area% |
|---|---|---|---|
| 1 | 7,573 | 20185322 | 99,588 |
| 2 | 8,799 | 83449 | 0,412 |
| Total | | 20268771 | 100,000 |

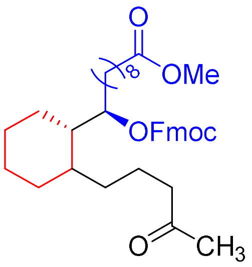


Method Description:
Column:Chiralpak IA 250x4,6mm Particle Size 5 micrometer
Solvent System: n-Heptan+0,1%IPA/IPA 92:8
Flow:0,7 ml/min

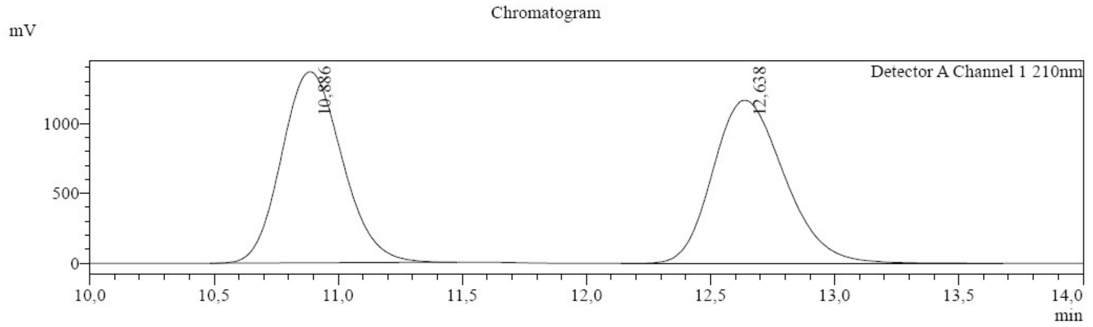


Detector A Channel 1 210nm

| Peak# | Ret. Time | Area | Area% |
|---|---|---|---|
| 1 | 10,886 | 23197684 | 49,804 |
| 2 | 12,638 | 23380160 | 50,196 |
| Total | | 46577844 | 100,000 |

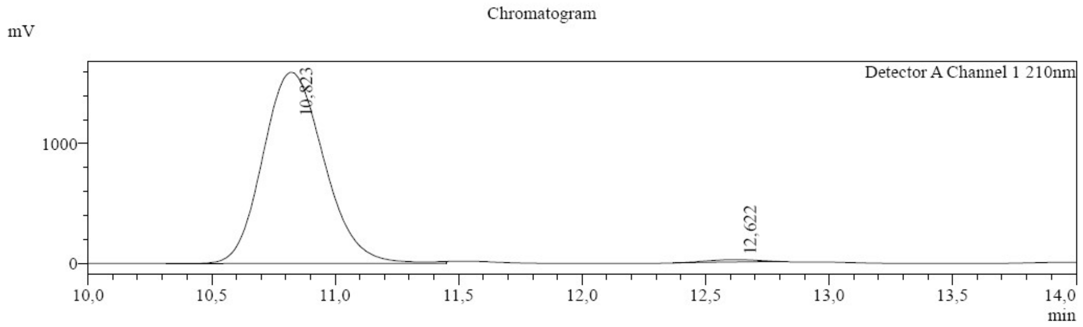


Detector A Channel 1 210nm

| Peak# | Ret. Time | Area | Area% |
|---|---|---|---|
| 1 | 10,823 | 27537174 | 99,018 |
| 2 | 12,622 | 273157 | 0,982 |
| Total | | 27810331 | 100,000 |
